# Unravelling the complex nature of resilience factors and their changes between early and later adolescence

**DOI:** 10.1186/s12916-019-1430-6

**Published:** 2019-11-14

**Authors:** J. Fritz, J. Stochl, E. I. Fried, I. M. Goodyer, C. D. van Borkulo, P. O. Wilkinson, A.-L. van Harmelen

**Affiliations:** 1 Department of Psychiatry, University of Cambridge, Cambridge, UK; 2 Department of Kinanthropology, Charles University, Prague, Czech Republic; 3 Department of Clinical Psychology, Leiden University, Leiden, the Netherlands; 4 Department of Psychological Methods, University of Amsterdam, Amsterdam, the Netherlands

**Keywords:** Resilience factors, Childhood adversity, Mental health, Adolescence

## Abstract

**Background:**

Childhood adversity (CA) is strongly associated with mental health problems. Resilience factors (RFs) reduce mental health problems following CA. Yet, knowledge on the nature of RFs is scarce. Therefore, we examined RF mean levels, RF interrelations, RF-distress pathways, and their changes between early (age 14) and later adolescence (age 17).

**Methods:**

We studied 10 empirically supported RFs in adolescents with (CA+; *n* = 631) and without CA (CA−; *n* = 499), using network psychometrics.

**Results:**

All inter-personal RFs (e.g. friendships) showed stable mean levels between age 14 and 17, and three of seven intra-personal RFs (e.g. distress tolerance) changed in a similar manner in the two groups. The CA+ group had lower RFs and higher distress at both ages. Thus, CA does not seem to inhibit RF changes, but to increase the risk of persistently lower RFs. At age 14, but not 17, the RF network of the CA+ group was less positively connected, suggesting that RFs are less likely to enhance each other than in the CA− group. Those findings underpin the notion that CA has a predominantly strong proximal effect. RF-distress pathways did not differ in strength between the CA+ and the CA− group, which suggests that RFs have a similarly protective strength in the two groups. Yet, as RFs are lower and distress is higher, RF-distress pathways may overall be less advantageous in the CA+ group. Most RF interrelations and RF-distress pathways were stable between age 14 and 17, which may help explain why exposure to CA is frequently found to have a lasting impact on mental health.

**Conclusions:**

Our findings not only shed light on the nature and changes of RFs between early and later adolescence, but also offer some accounts for why exposure to CA has stronger proximal effects and is often found to have a lasting impact on mental health.

## Background

Adolescents who have been exposed to adversity in childhood (CA), such as traumatic and/or severely stressful events, have a higher risk of developing mental health problems [1–3]. Moreover, approximately one in two children and adolescents worldwide experience adverse events before the age of 18 [1–4]. Therefore, it is imperative that the deleterious mental health consequences following CA are addressed in research, therapy, and mental health policy. This notion has not only been noticed in science [3, 5], but has also led to a discussion in public media questioning whether “… childhood trauma [should] be treated as a public health crisis?” (NPR: National Public Radio, 09 November 2018) [6] and whether “… people [can] be saved from a terrible childhood?” (The Guardian, 07 November 2018) [7]. One way to understand better how we can reduce the deleterious consequences of CA is to study the complex nature of resilience factors (RFs), i.e. factors that are empirically found to reduce the risk of mental health problems following CA [8, 9]. To this end, we here aim to shed light on the longitudinal nature of RFs between two time points, respectively marking early and later adolescence.

RFs operate on various intertwined functioning levels encompassing biological (e.g. genes or hormones), intra-personal (e.g. distress tolerance), and inter-personal levels (e.g. peer support) [8, 10, 11]. We will focus on the latter two categories as those RFs can be targeted in psychosocial interventions and may therefore be particularly relevant in informing translational research and thus eventually prevention and therapy.

Despite the fact that RFs do not function in isolation, most studies have investigated single RFs [8, 12]. Recently, researchers have argued that to improve our understanding of resilience mechanisms, it is necessary to move from relatively simple reductionist towards more holistic, complex models [12–14]. In several research fields, complex system models have been applied to describe risk and resilience processes, as for instance for financial markets or ecosystems [13, 15, 16]. Complex system models promise to fit the complexity of resilience research well, as they enable the exploration of multiple interconnected factors that are assumed to reinforce each other. Recently, we took the first step in bridging this gap for resilience research focussing on mental health in the face of adversity. We showed that RFs function as a complex interrelated network in both adolescents with and without CA, at age 14 [17]. We found that the group of adolescents with CA had lower RF mean levels and the RFs were less positively interrelated, suggesting that the RFs may not enhance each other to the same extent as in adolescents without CA [17].

Mental health levels can change over time, particularly during the process of dealing with adversity [18–21]. This suggests that RFs and/or their interrelations may also change over time. Individuals with CA often have lower levels of RFs [17, 22], which are suggested to be transferred forward across development [3, 23]. Hence, it is crucial to determine how RFs change over time in adolescents with and without CA, as this firstly unravels whether RFs change similarly or differently in the two groups, and secondly reveals which RFs improve, deteriorate, or stay stable during adolescence. Such RF changing patterns can inform translational research which in turn can shed light on the RFs that should be targeted and promoted to aid successful development after CA [3, 23]. However, research on RF changes is surprisingly scarce, and results are mixed: Some intra- and inter-personal RFs are found to increase (e.g. ruminative worrying, prosocial involvement), whereas others have been reported to stay stable between early and later adolescence (e.g. family involvement, expressive suppression, dysfunctional rumination) [23–25]. Here, we therefore examined whether RFs change between early (age 14) and later (age 17) adolescence, through investigating (a) RF mean levels, (b) RF interrelations, and (c) the way RFs are interrelated with distress (directly and/or indirectly via other RFs). Importantly, we specifically examined whether RFs change differentially in groups of adolescents with (CA+) and without CA (CA−).

## Methods

### Design

In 2005 and 2006, 1238 14-year-old adolescents were recruited from schools in Cambridgeshire to take part in the longitudinal ROOTS study. Follow-up took place around age 17 [26]. Consent was provided by the adolescents and one parent [26]. ROOTS was conducted following Good Clinical Practice guidelines and the Declaration of Helsinki and was approved by the Cambridgeshire Research Ethics Committee (03/302) [27].

### Sample

In the current study, we performed all main analyses on 1130 of the 1238 participants. We included all those who had data for potential CA experiences (CA+: *n* = 638; CA−: *n* = 501) and had less than 85% missingness on the analyses variables (*n* = 1188), resulting in 631 adolescents with and 499 adolescents without prior exposure to CA.

### Measures

#### Childhood adversity (CA)

CA was assessed with the semi-structured Cambridge Early Experience Interview (CAMEEI) that mainly measures intra-family-related adversity before the age of 14 [27]. The interview was conducted with the primary caregiver, which was in 96% of the cases the biological mother. All interviews were performed when the adolescents were 14 years old. The CAMEEI was designed to measure adverse events in three time windows (0–5, 5–11, and 11–14 years), to support recall accuracy. Several types of adverse experiences were measured: loss of a family member, family separations (> 6 months), divorce, death, adoption, discord within the family, absence of maternal affection/involvement, aberrant parenting style, significant medical illnesses within the family, psychopathology of family members, times of parental unemployment, financial hardship, physical abuse, sexual abuse, emotional abuse, criminality of family members, acute life events (e.g. environmental event with impact on the living situation), and chronic social hardship (e.g. demands of caring for extended family) [27]. Based on this information, Dunn and colleagues [27] performed a latent class analysis, which revealed four classes (no CA, moderate CA, severe CA, and aberrant parenting CA) for each of the three time windows. In line with previous reports [17], adolescents were assigned a “0” when they belonged for all three time windows to the “no CA” category (CA−), and were assigned a “1” when they belonged for at least one time window to a category other than “no CA” (CA+; see Table 1 for detailed numbers).

**Table 1:** Numbers CA exposure (CA+ = 638, CA− = 501)

0 to 5 years	5 to 11 years	11 to 14 years	CA variable	Cumulative number of participants with CA		
CA+ = 355	CA+ = 463	CA+ = 406	CA+ = 638	1 time window	2 time windows	3 time windows
CA− = 784	CA− = 676	CA− = 733	CA− = 501	*n* = 262	*n* = 166	*n* = 210

#### General distress

To compile a general distress index, we used the 13-item short form of the Mood and Feelings Questionnaire (MFQ) [31], measuring a broad range of depression-related symptoms, and the 28-item Revised Children’s Manifest Anxiety Scale (RCMAS) [32], measuring a wide range of anxiety-related symptoms. We used confirmatory factor analysis (CFA) based on polychoric correlations to estimate one underlying latent general distress factor for those 41 items. Brodbeck et al. [33], Stochl et al. [34], and St Clair et al. [35] used similar approaches and showed that a latent general distress factor replicates well in adolescent samples. Please note, for computational reasons, we have used fewer depression items for the general distress factor than in our previous report [17] (for a detailed rationale see Additional file 1).

#### Resilience factors (RFs)

Based on findings of our preregistered systematic review [8], we included 8 self-report (1–8 below) and 2 parent report RFs (9–10 below) that were assessed in our adolescent cohort. All RFs are scored in such a way that high values are protective, to which end five of the scales were reversed:

1. Friendship support was assessed with five items of the Cambridge Friendships Questionnaire [36].
2. Family support was assessed with five items of the McMaster Family Assessment Device [37].
3. Family cohesion was assessed with seven items of the McMaster Family Assessment Device [37].
4. Positive self-esteem was assessed with five items of the Rosenberg self-esteem scale [38].
5. Negative self-esteem was assessed with five items of the Rosenberg self-esteem scale [38]. We reversed the items so that high values of low negative self-esteem are protective.
6. Reflective rumination was assessed with five items of the Ruminative Response Scale (RRS) [39, 40]. We reversed the items so that high values of low reflective rumination are protective.
7. Ruminative brooding was assessed with five items of the RRS [39, 40]. Please note the ruminative brooding factor does not match the one used in our previous report [17], for a detailed rationale see Additional file 1 and Additional file 2. We reversed the items so that high values of low ruminative brooding are protective.
8. Aggression was assessed with four items of the Behaviour Checklist (11 questions based on the DSM-IV criteria for conduct problems) [41, 42]. We reversed the items so that high values of low aggression are protective.
9. Distress tolerance was assessed with five items of the Emotionality Activity Sociability Temperament Survey [43].
10. Expressive suppression was assessed with one item of the Antisocial Process Screening Device [44]. We reversed the item so that high values of low expressive suppression are protective.

Information regarding the psychometric properties of the RF measures is reported by Fritz and colleagues [17] (i.e. in Supplement XIV).

### Analysis

All analyses were conducted with R version 3.5.1 [45]. All used packages and the belonging version numbers can be found in Additional file 3.

#### Variable preparation

A minor subset of participants had incidentally missing items and some participants had missingness due to attrition, both detailed in Additional file 4: Table S2. The identified missingness patterns on most RFs and general distress could partially be accounted for by exposure to CA, being male, having a low mood, and having a psychiatric history prior to the age of 14 (see Additional file 4: Table S3). Accordingly, we used multivariate multiple imputation algorithms with chained equations to impute the missing data [46]. We computed 10 imputation data sets each with 100 iterations, using predictive mean matching algorithms for ordered categorical items and logistic regression for dichotomous items. The imputation models were based on seven descriptive variables (CA, gender, socio-economic status, prior psychiatric history at occasions 1 and 2, and age at occasions 1 and 2), as well as 50 RF, 33 depression-related, and 28 anxiety-related items for both occasions, resulting in a total of 229 items. In contrast to missingness on the RF or distress variables, we did not impute data for the CA variable. We made this decision as we felt that some forms of CA, such as a traumatizing car crash or being exposed to fire in the home, are in our opinion not sufficiently predictable to be imputed for missingness. The imputed data sets contained data for 1188 participants. To estimate the best fitting latent RF and distress indices, we used CFA models and extracted the resulting factor scores as RF and general distress variables. We decided to use factor scores instead of sum scores to reduce measurement error and to circumvent tau-equivalence (for a rational, see Additional file 5: Part A). As we aimed to compare two time points, we estimated longitudinal CFAs (LCFAs; separately for each RF and general distress). Given that all RF and general distress items were assessed with three to six answer categories, we computed categorical LCFAs [47], treated the items as ordinal, and used a weighted least square mean and variance adjusted (WLSMV) estimator (for details see Additional file 5: Part B). Distribution plots for the RFs and general distress are in Additional file 5: Figure S5. Hence, all main analyses were performed on 1130 participants (CA+ *n* = 631, CA− *n* = 499) who had data for potential CA experiences (*n* = 1139) and had less than 85% missingness on the analyses variables (*n* = 1188). In contrast to the analyses, all descriptive statistics are computed on the un-imputed data and may therefore contain slightly different sample sizes. The interested reader can find analysis results not being based on imputed data in Additional file 18.

#### Investigating RF mean level changes

To examine whether RFs (a) differ in their protective value between the CA+ and the CA− group and (b) change in their protective value between age 14 and 17, we conducted RF mean comparison analyses. More specifically, we compared the RF and general distress mean levels (a) between the CA+ and the CA− group (i.e. separately for age 14 and 17), and (b) between age 14 and age 17 (i.e. separately in the CA+ and CA− groups). To ensure latent mean comparability across ages, we estimated strongly invariant categorical LCFAs [47], for which the exact LCFA parameter specifications and model identification details are outlined in Additional file 5: Part B. All strongly invariant categorical LCFAs fitted satisfactorily (Additional file 5: Part B Table S5). We did not compute an LCFA for the expressive suppression RF, as this RF was measured with only one item. We binarized the aggression and expressive suppression RFs, as they showed a restricted range. To circumvent slight deviations from normality, we tested CA+ vs CA− mean level differences with independent sample Wilcoxon rank-sum tests (with continuity correction). Moreover, we compared age 14 and age 17 mean levels with paired sample Wilcoxon signed rank tests (with continuity correction). As sensitivity analyses, we re-ran the mean change analyses (a) with factor scores retrieved from the full invariance models (see Additional file 6) and (b) with sum scores (see Additional file 6). All mean comparisons were corrected for the false discovery rate [48]. Additionally, we explored whether CA moderates the relationship between age and RFs, to test whether the change patterns of the RFs differ between the two groups.

#### Investigating network structure changes

To examine (a) whether RFs interrelate differently in the CA+ and the CA− groups and (b) whether those RF interrelations change between age 14 and 17, we computed RF network models. More specifically, we used RF factor scores to estimate regularized partial correlation network models [49]. Those models were computed separately for adolescents with and without CA, as well as for age 14 and age 17. We compared the resulting models with each other using permutation tests (i.e. network comparison tests (NCTs)) [50]. To ensure that the exchangeability assumption of permutation tests was met (i.e. the joint distribution of the scores is invariant when permuting over time), we estimated fully invariant categorical LCFAs. The exact LCFA parameter specifications and details regarding the model identification can be found in Additional file 5: Part B. All fully invariant categorical LCFAs fitted satisfactorily (see Additional file 5: Part B Table S5). As above, we did not compute an LCFA for expressive suppression, and we again binarized the aggression and expressive suppression RFs. We estimated (a) networks only containing the 10 RFs, (b) networks containing both the 10 RFs and the general distress factor, and (c) networks containing the 10 RFs corrected for general distress levels. To ensure conciseness, we here discuss the RF network models being corrected for general distress levels, as those enable the comparison of the CA+ and the CA− groups when taking the putatively confounding effect of psychopathology levels into account. The other two models are discussed in Additional file 7.

For the comparisons of the four network models (i.e. CA+ vs CA− = independent sample permutation tests, and age 14 vs age 17 = paired sample permutation tests), we conducted three types of network comparison tests (two-tailed; we used an adjusted version of [50]). Firstly, we investigated whether the highest interrelation difference between the respective two networks differs from the highest interrelation differences of several (i.e. 5000 permutations) randomly permuted network model pairs, which indicates whether the two tested network structures are invariant [50]. Secondly, we investigated whether the relative connectivity, which is the sum of the positive interrelations after subtracting the sum of the negative interrelations, differed between the two respective networks. This test is also called “global network expected influence” comparison [17, 51] and indicates to which degree RFs are concurrently positively associated. This test is of particular interest here, as it suggests to which degree RFs can concurrently enhance each other. Thirdly, we explored which individual RF interrelations and/or interrelations between RFs and general distress differed between the respective two networks of interest (for details, see [50]). Hence, the first two tests examine *global* network structure differences, whereas the third test examines *local* network structure differences.

#### Investigating RF-general distress pathway changes

To examine the way RFs are interrelated with distress in the network models, we calculated two types of pathways between the RFs and general distress. First, we examined the direct pathways between the RFs and general distress, regardless of whether those pathways are the strongest or “quickest” ways to traverse the network from the RFs to general distress [52]. Second, we examined the shortest pathways (or “shortest path lengths”) between the RFs and general distress, regardless of whether the RFs have direct pathways with general distress. More specifically, we explored whether the shortest pathway to traverse the network from a given RF to the general distress variable is direct or indirect via other RFs [53]. Moreover, we conducted permutation tests to compare the two types of pathways between the CA+ and the CA− group, for both age 14 and age 17. Lastly, we examined whether the two types of pathways changed between age 14 and 17 (i.e. separately for the CA+ and the CA− groups), again using permutation tests. Correlations and regularized partial correlations between the RFs and the general distress variable, for both CA+ and CA− as well as for age 14 and age 17, are discussed in Additional file 8.

#### Network stability, accuracy, and inference

To test the robustness of our network model parameters, we estimated the stability of expected influence (EI) coefficients and the accuracy of all interrelations. We tested the stability of the EI coefficients by applying a subset bootstrap (2000 bootstraps) to identify the maximum sample percentage that can be dropped to reveal (with a 95% chance) a relationship of ≥ 0.7 between the subset and the original EI coefficients [54]. Moreover, we tested the accuracy of the network models by bootstrapping all interrelations (2000 bootstraps) and investigated their bootstrapped confidence intervals (CIs) [54]. Those analyses are reported in Additional file 9. We further explored the node expected influence coefficients for individual RFs (i.e. the sum of all positive interrelations of the respective RF, after subtracting the sum of the negative interrelations of that RF) [55, 56], which are reported in Additional file 10.

#### Network sensitivity analyses

To establish whether our results would hold if the RFs were computed differently, we re-estimated the network models (a) based on factor scores of the configural LCFAs, which do not constrain parameters across time points but estimate the best fitting time point specific latent factor, and (b) based on sum scores. Results were overall similar and are discussed in Additional file 11 and Additional file 12.

#### Data availability

Data for this specific paper has been uploaded to the Cambridge Data Repository 10.17863/CAM.36708 and is password protected. Our participants did not give informed consent for their measures to be made publicly available, and it is possible that they could be identified from this data set. Access to the data supporting the analyses presented in this paper will be made available to researchers with a reasonable request to openNSPN@medschl.cam.ac.uk.

#### Code availability

Analysis code is available from http://jessica-fritz.com/.

## Results

### Sample

The CA+ and the CA− groups did not differ with regard to age or gender, but the CA+ group had a lower socio-economic status (see Table 2). In addition, adolescents in the CA+ group were more likely to have a psychiatric history and had higher levels of depression and anxiety symptoms, at both age 14 and 17.

**Table 2:** Sample comparisons: CA+ (*n* = 638) versus CA− (*n* = 501) groups

	CA+	CA−	t^*1^/z^*2^/*X*^2*3^ (*DF*)	95% CI^*4^	*p*
Gender	*n* girls = 358	*n* girls = 262	1.50 (1)		.22
	*n* boys = 280	*n* boys = 239			
SES^*5^	*n* hard pressed = 77	*n* hard pressed = 30	5.45		< .001
	*n* moderate means = 36	*n* moderate means = 11			
	*n* comfortably off = 170	*n* comfortably off = 105			
	*n* urban prosperity = 37	*n* urban prosperity = 41			
	*n* wealthy achievers = 318	*n* wealthy achievers = 314			
Age 14					
Age	*M* = 14.49, *SD* = 0.28	*M* = 14.48, *SD* = 0.28	− 0.43 (1049.3)	− .04 to .03	.67
Psychiatric history (PH)^*6^	*n* PH = 201	*n* PH = 74	42 (1)		< .001
	*n* no-PH = 437	*n* no-PH = 427			
Depression symptoms	*M* = 17.42, *SD* = 11.61	*M* = 14.03, *SD* = 10.46	− 5.10 (1088.5)	− 4.69 to − 2.09	< .001
Anxiety symptoms	*M* = 16.92, *SD* = 12.61	*M* = 13.92, *SD* = 11.28	− 4.17 (1089.2)	− 4.42 to − 1.59	< .001
Age 17					
Age	*M* = 17.49, *SD* = 0.34	*M* = 17.48, *SD* = 0.32	− 0.56 (1017.5)	−.05 to .03	.58
PH^*6^	*n* PH = 268	*n* PH = 122	48.48 (1)		< .001
	*n* no-PH = 297	*n* no-PH = 345			
Depression symptoms	*M* = 16.36, *SD* = 12.27	*M* = 12.38, *SD* = 10.19	− 5.51 (967.61)	− 5.39 to − 2.56	< .001
Anxiety symptoms	*M* = 15.02, *SD* = 12.72	*M* = 11.53, *SD* = 10.96	− 4.58 (967.76)	−4.98 to − 1.99	< .001

*Note. CA* childhood adversity, *SES* socio-economic status. ^*1^We applied Welsh’s two-tailed independent sample *t* test to account for potentially unequal variances across groups. ^*2^As SES was split in five ordered categories, we applied the two-tailed Asymptotic Cochran-Armitage test [28]. ^*3^We applied two-tailed Pearson’s chi-square tests. ^*4^The confidence interval (CI) for the difference in location estimates, corresponding to the alternative hypothesis. ^*5^SES was assessed with the ACORN classification system (http://www.caci.co.uk) [29]. ^*6^Psychiatric history was assessed with the Schedule for Affective Disorders and Schizophrenia for School-Age Children (Present and Lifetime Version), at age 14 additionally including learning disabilities, clinical sub-threshold diagnoses, and deliberate self-harm, and at age 17 additionally including clinical sub-threshold diagnoses and deliberate self-harm [30]

### RF mean level changes

#### Group comparisons

At both age 14 and 17, distress was significantly higher and nine of the ten RFs were significantly lower in the CA+ group (please note, RFs are scored in such a way that higher levels are more protective; see Table 3). The tenth RF, reflective rumination, was also significantly lower in the CA+ group, but only at age 17, not at 14. The general pattern clearly indicates that RFs are lower and distress is higher in the CA+ than in the CA− group, during both early and later adolescence.

**Table 3:** RF and general distress comparisons: CA+ (*n* = 631) versus CA− (*n* = 499) groups

	Age	CA+	CA−	*W/χ*^2^*(df)*	95% CI^*1^	p^*2^
Friendship support (high)	14	0.09	0.23	173,600	.04 to .22	< .01
	17	0.07	0.30	180,700	.12 to .33	< .001
Family support (high)	14	− 0.02	0.17	178,690	.09 to .29	< .001
	17	− 0.07	0.14	180,780	.12 to .33	< .001
Family cohesion (high)	14	− 0.10	0.29	198,690	.30 to .51	< .001
	17	− 0.18	0.29	198,080	.37 to .63	< .001
Negative self-esteem (low)	14	0.06	0.29	182,270	.11 to .31	< .001
	17	0.10	0.55	187,900	.25 to .58	< .001
Positive self-esteem (high)	14	− 0.08	0.21	188,440	.20 to .41	< .001
	17	− 0.14	0.22	192,880	.26 to .50	< .001
Ruminative brooding (low)	14	0.03	0.19	175,000	.07 to .28	< .01
	17	− 0.07	0.12	182,540	.11 to .28	< .001
Reflective rumination (low)	14	0.10	0.20	167,440	− .00 to .19	.066
	17	− 0.08	0.00	170,430	.01 to .15	< .05
Distress tolerance (high)	14	− 0.06	0.25	188,300	.21 to .43	< .001
	17	0.02	0.42	195,600	.30 to .53	< .001
Aggression (low)	14	Low: 498 (s = 1)	Low: 440 (s = 1)	16.27 (1)		< .001
		High: 133 (s = 0)	High: 59 (s = 0)			
	17	Low: 491 (s = 1)	Low: 425 (s = 1)	09.35 (1)		< .01
		High: 140 (s = 0)	High: 74 (s = 0)			
Expressive suppression (low)	14	Low: 418 (s = 1)	Low: 371 (s = 1)	08.31 (1)		< .01
		High: 213 (s = 0)	High: 128 (s = 0)			
	17	Low: 396 (s = 1)	Low: 355 (s = 1)	08.42 (1)		< .01
		High: 235 (s = 0)	High: 144 (s = 0)			
General distress	14	− 0.09	− 0.40	130,950	− .43 to − .18	< .001
	17	− 0.09	− 0.68	125,400	− .75 to − .38	< .001

*Note. CA* childhood adversity. All RFs are scored in such a way that high values are protective (e.g. high levels of high friendship support or high levels of low negative self-esteem) and low values are harmful (e.g. low levels of high friendship support or low levels of low negative self-esteem). The continuous general distress variable is scored in such a way that the higher the value the higher the level of general distress. ^*1^The confidence interval (CI) for the difference in location estimates, corresponding to the alternative hypothesis. ^*2^Please note the *p* values are corrected for the false discovery rate, which is why the CIs do not have to contain 0 for the *p* value to be nonsignificant

#### Temporal comparisons

**Fig. 1:**
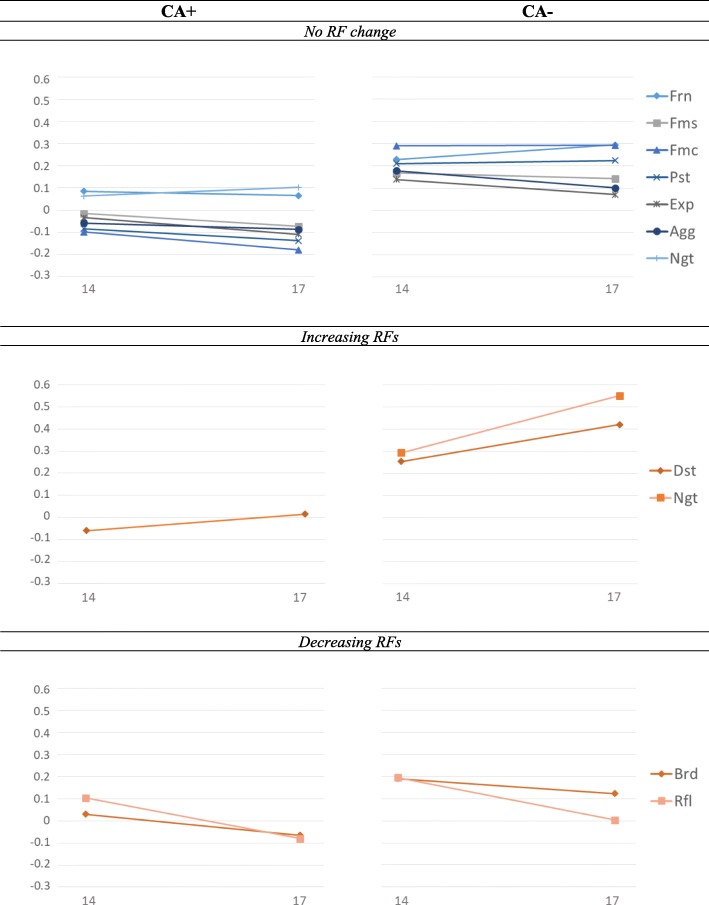
RF mean level comparisons. CA = childhood adversity. All scores are derived from strongly invariant confirmatory factor analyses. All RFs are scored in such a way that high values are protective (e.g. high levels of high friendship support or high levels of low negative self-esteem) and low values are harmful (e.g. low levels of high friendship support or low levels of low negative self-esteem). Legend: Frn = friend support, Fms = family support, Fmc = family cohesion, Ngt = negative self-esteem, Pst = positive self-esteem, Rfl = reflection, Brd = brooding, Dst = distress tolerance, Agg = aggression, Exp = expressive suppression

In both groups, two RFs had lower mean levels at age 17 than at age 14: ruminative brooding and reflection. In the CA− group, distress tolerance and negative self-esteem had higher mean levels at age 17 than at age 14. In the CA+ group, only distress tolerance had higher mean levels at age 17 than at age 14. All other RFs did not change significantly over time (see Fig. 1). Importantly, age-CA interaction effects did not predict the RFs and general distress (see Table 4). Therefore, all RFs that changed between age 14 and 17 changed similarly in the two groups.

**Table 4:** RF and general distress comparisons: age 14 versus age 17

	CA	Age 14	Age 17	*V*	95% CI^*1^	p^*2^	age_*x*_CA^*3^	age_*x*_CA *p*
Friendship support (high)	Yes	0.09	0.07	102,800	− .04 to .08	.55	− .09	.63
	No	0.23	0.30	55,837	− .13 to − .00	.08		
Family support (high)	Yes	− 0.02	− 0.07	109,330	.00 to .12	.07	− .03	.81
	No	0.17	0.14	64,965	− .03 to .09	.49		
Family cohesion (high)	Yes	− 0.10	− 0.18	110,280	.01 to .14	.06	− .08	.63
	No	0.29	0.29	61,400	− .08 to .06	.76		
Negative self-esteem (low)	Yes	0.06	0.10	90,292	− .19 to − .01	.07	− .22	.13
	No	0.29	0.55	41,185	− .43 to − .24	< .001		
Positive self-esteem (high)	Yes	− 0.08	− 0.14	108,460	− .00 to .11	.09	− .07	.63
	No	0.21	0.23	59,923	− .09 to .04	.49		
Ruminative brooding (low)	Yes	0.03	− 0.07	116,300	.05 to .16	< .01	− .03	.81
	No	0.19	0.12	71,074	.02 to .14	< .05		
Reflective rumination (low)	Yes	0.10	− 0.08	130,350	.14 to .26	< .001	.01	.96
	No	0.20	0.00	82,603	.14 to .27	< .001		
Distress tolerance (high)	Yes	− 0.06	0.02	81,643	− .11 to − .04	< .001	− .09	.63
	No	0.25	0.42	36,790	− .20 to − .13	< .001		
Aggression (low)	Yes	Low: 498 (=1)	Low: 491 (=1)	7138		.59	1.22	.63
		High: 133 (=0)	High: 140 (=0)					
	No	Low: 440 (=1)	Low: 425 (=1)	2438		.18		
		High: 59 (=0)	High: 74 (=0)					
Expressive suppression (low)	Yes	Low: 418 (=1)	Low: 396 (=1)	9333		.14	1.01	.96
		High: 213 (=0)	High: 235 (=0)					
	No	Low: 371 (=1)	Low: 355 (=1)	4375		.21		
		High: 128 (=0)	High: 144 (=0)					
General distress	Yes	− 0.09	− 0.09	106,940	− .02 to .22	.14	.27	.13
	No	− 0.40	− 0.68	79,608	.22 to .46	< .001		

*Note. CA* childhood adversity. All RFs are scored in such a way that high values are protective (e.g. high levels of high friendship support or high levels of low negative self-esteem) and low values are harmful (e.g. low levels of high friendship support or low levels of low negative self-esteem). The continuous general distress variable is scored in such a way that the higher the value the higher the level of general distress. ^*1^The confidence interval (CI) for the difference in location estimates, corresponding to the alternative hypothesis. ^*2^Please note the *p* values are corrected for the false discovery rate, which is why the CIs do not have to contain 0 for the *p* value to be nonsignificant. ^*3^For linear models the interaction is reported as *b* value and for binomial logit models as odds ratio

### RF interrelation changes

#### Group comparisons

**Fig. 2:**
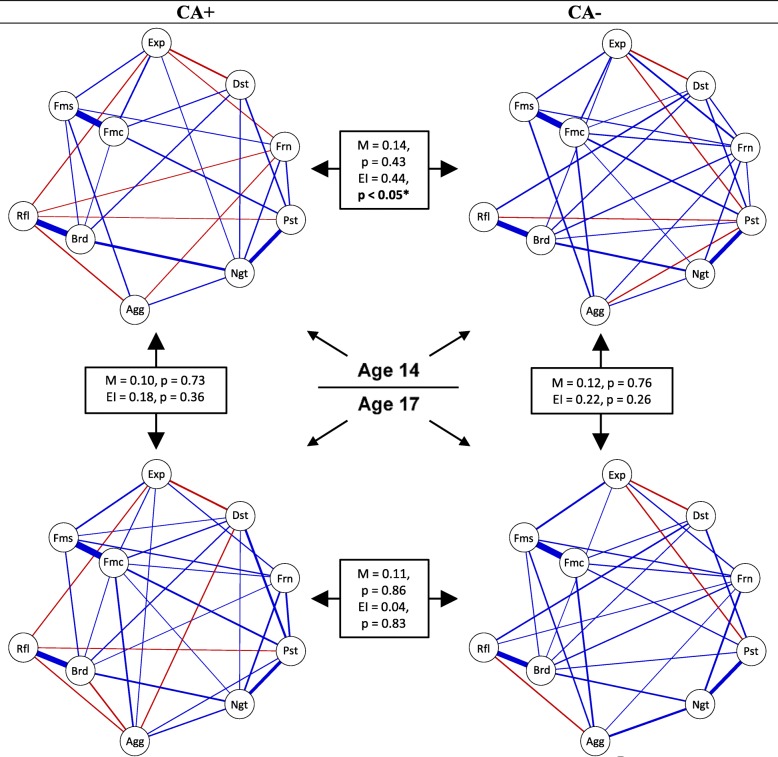
CA+ (*n* = 631) and CA− (*n* = 499) resilience factor networks for age 14 (upper panel) and age 17 (lower panel) corrected for the general distress variable. Width of the lines = association strength. Positive interrelations = blue, negative interrelations = red. Legend: Frn = friend support, Fms = family support, Fmc = family cohesion, Ngt = negative self-esteem, Pst = positive self-esteem, Rfl = reflection, Brd = brooding, Dst = distress tolerance, Agg = aggression, Exp = expressive suppression, GD = general distress. The boxes depict the maximal interrelation difference between the respective two networks (M), the difference in global network expected influence (EI) between the respective two networks (EI), and the corresponding *p* values (5000 comparison samples). The above networks with faded interrelations can be found in Additional file 13. Please note, the upper panel of the figure is similar to a figure in a previous report on this sample (see [17] in Scientific Reports; can be retrieved from 10.1038/s41598-018-34130-2; information regarding the publishing license of the original figure, and information regarding differences with the above figure can be found in Additional file 14)

Figure 2 depicts the RF networks that are corrected for general distress for the CA+ and the CA− group, as well as for age 14 and 17 (for additional information see Additional files 13 and 14). For age 14, the CA+ and CA− networks were invariant (*M* = .14, *p* = .43). However, the global network expected influence, which indicates the degree to which RFs are positively interrelated, was significantly lower in the CA+ network (EI_CA+_ = 2.27, EI_CA−_ = 2.71, EI = 0.44, *p* = .02). This suggests that in the CA+ network RFs are less likely to enhance each other than in the CA− network. Four individual RF interrelations differed between the CA+ and the CA− networks (see Additional file 15: Table S9). For age 17, both the global network structure invariance and the expected influence comparison tests were not significant (*M* = .11, *p* = .86; EI_CA+_ = 2.45, EI_CA−_ = 2.49, EI = 0.04, *p* = .83). Moreover, only one individual RF interrelation differed between the CA+ and the CA− networks (see Additional file 15: Table S9).

#### Temporal comparisons

When we compared the networks between age 14 and 17, the networks were invariant and did not differ in global network expected influence, in both the CA+ (*M* = .10, *p* = .73; EI_14_ = 2.27, EI_17_ = 2.45, EI = 0.18, *p* = .36) and the CA− group (*M* = .12, *p* = .76; EI_14_ = 2.71, EI_17_ = 2.49, EI = 0.22, *p* = .26). In the CA+ network, two individual RF interrelations changed significantly between age 14 and 17, whereas none changed in the CA− network, see Additional file 15: Table S10.

### Changes in pathways between RFs and general distress

#### Group comparisons

**Fig. 3:**
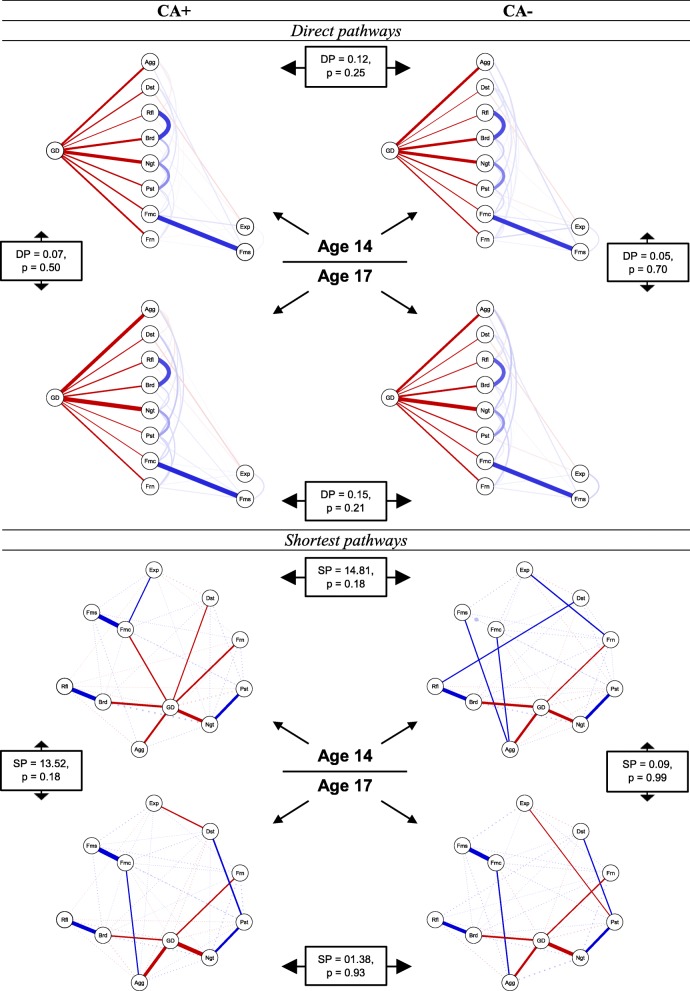
Direct (DP) and shortest pathways (SP) between the resilience factors (RFs) and the general distress variable, for the CA+ (*n* = 631) and the CA− (*n* = 499) group. The upper panel depicts direct and the lower panel the shortest pathways between the RFs and general distress. Within the panels, the upper part depicts the networks for age 14 and the lower part the networks for age 17. Non-transparent lines = direct/shortest pathway of interest. Transparent/dotted lines = all remaining partial regularized correlation relationships. Positive interrelations = blue, negative interrelations = red. Legend: Frn = friend support, Fms = family support, Fmc = family cohesion, Ngt = negative self-esteem, Pst = positive self-esteem, Rfl = reflection, Brd = brooding, Dst = distress tolerance, Agg = aggression, Exp = expressive suppression. Please note, the upper part of the lower panel is similar to a figure in a previous report on this sample (see [17] Scientific Reports; can be retrieved from 10.1038/s41598-018-34130-2; information regarding the publishing license of the original figure, and information regarding differences with the above figure can be found in Additional file 14)

First, we explored the *direct pathways* between the RFs and general distress (Fig. 3 upper panel). At age 14, most RFs had negative direct pathways, in both the CA+ and the CA− group, indicating that high RFs go together with low distress (or vice versa). Yet, those *negative direct pathways* to distress did overall not differ in strength between the CA+ and the CA− group (DP_CA+_ = − 1.40, DP_CA−_ = − 1.28, DP = 0.12, *p* = .25, i.e. a more negative DP value indicates a stronger (negative) direct pathway and a less negative DP value indicates a weaker (negative) direct pathway). At age 17, the results were similar as the strength of the *direct pathways* did not differ between the two groups (DP_CA+_ = − 1.47, DP_CA−_ = − 1.33, DP = 0.15, *p* = .21). Importantly, the *direct pathway* results do not consider that some RFs have stronger *indirect* than *direct* effects on distress, i.e. via other RFs. To this end, we next calculated *shortest pathways* between RFs and distress, which indicate the quickest way to traverse the network from the RF to distress (Fig. 3 lower panel). At age 14, the majority of RFs in the CA+ group had a *direct shortest pathway* with general distress (i.e. 6 out of 10), whereas the majority of RFs in the CA− group had an *indirect shortest pathway* with distress (i.e. 6 out of 10). However, the overall strength of the *shortest pathways* did not differ between the two groups (SP_CA+_ = 78.62, SP_CA−_ = 93.42, SP = 14.81, *p* = .18, i.e. a lower SP value indicates a stronger (and thus shorter) shortest pathway and a higher SP value indicates a weaker (and thus longer) shortest pathway). At age 17, the two groups no longer differed in the number of *negative shortest pathways* and neither in the strength of the *shortest pathways* (SP_CA+_ = 92.13, SP_CA−_ = 93.51, SP = 1.38, *p* = .93).

#### Temporal comparisons

When comparing the *direct pathways* between the RFs and general distress between age 14 and age 17, no significant temporal differences were found in the CA+ (CA+: DP_14_ = − 1.40, DP_17_ = − 1.47, DP = 0.07, *p* = 0.50) and the CA− group (DP_14_ = − 1.28, DP_17_ = − 1.33, DP = 0.05, *p* = 0.70). Similarly, when comparing the *shortest pathways* between age 14 and age 17, we again did not find significant temporal differences in the CA+ (SP_14_ = 78.62, SP_17_ = 92.13, SP = 13.52, *p* = 0.18) and the CA− group (SP_14_ = 93.42, SP_17_ = 93.51, SP = 0.09, *p* = 0.99).

## Discussion

We aimed to shed light on RF changes between age 14 and age 17 and investigated (a) RF mean levels, (b) RF interrelations, and (c) pathways from the RFs to general distress, in adolescents with and without CA. Regarding RF mean levels (a), we found that although inter-personal RFs (e.g. friendships) seemed to stay stable, some intra-personal RFs (e.g. distress tolerance) changed between age 14 and 17. Interestingly, all RFs that in- or decreased between age 14 and 17 changed similarly in the two groups. Moreover, the CA+ group had lower RFs and higher distress at both ages. Regarding RF interrelations (b), we found that at age 14, but not at age 17, RFs were less positively interrelated in the CA+ group. This suggests that the RFs are less likely to enhance each other in the CA+ compared to the CA− network. Regarding RF-distress pathways (c), our results indicate that the strength of the pathways did neither differ between the CA+ and the CA− group, nor over time, suggesting that RFs may be similarly protective in both groups and at both ages. Below we will outline how our findings inform about the complex nature of RFs and will discuss tentative accounts for why CA not only has strong proximal effects, but is often found to have a lasting impact on mental health.

### RF mean level changes

All inter-personal RFs (i.e. friendship support, family support, and family cohesion) seemed to stay stable between age 14 and 17, showing that, in this cohort, adolescents perceive their social support environment to be similar during early and later adolescence. The mean levels of some intra-personal RFs changed however between age 14 and 17 (i.e. distress tolerance, brooding, and reflection in both groups, as well as negative self-esteem in the CA− group). Adolescents reported a higher level of distress tolerance at age 17 than at age 14, which potentially may be explained by the improvement of executive functions and emotion regulation strategies. Previous literature has shown that executive functions, such as inhibitory control which facilitates the regulation of cognition and behaviour, develop and improve until adulthood [57, 58]. Similarly, the use of emotion regulation strategies is found to be significantly lower in mid-adolescence (age 15) than in young adulthood (age 19) [25].

In the literature, findings regarding changes in rumination are mixed. For example, Zimmerman and Iwanski [25] did not find a significant difference in rumination between age 13 and 17, whereas Frydenberg and Lewis [24] showed that ruminative worrying is higher at age 16 than at age 14. In line with Frydenberg and Lewis [24], our sample reported higher (more harmful) levels of reflective rumination and ruminative brooding at age 17 than at age 14. Besides the increase in rumination, our CA− group reported a decrease in negative self-esteem between age 14 and 17. Those results together suggest that although CA− adolescents may worry and reflect more about their experiences and behaviours during later adolescence, they may not attach those negative thoughts and evaluations to their self-image. Despite the fact that there was no significant decrease in negative self-esteem in the CA+ group, the change in negative self-esteem from age 14 to 17 did not differ significantly between the two groups. While further replication of our results is required, we suggest that between early and later adolescence mechanisms emerge that alter the perception of the self (e.g. negative self-esteem, rumination) and self-regulation (e.g. distress tolerance, rumination) [23–25, 57, 58].

Our results further showed that all changes in RF mean levels between early and later adolescence were similar in the CA+ and the CA− groups. Crucially, however, the CA+ group had lower RFs at both ages, which is in line with previous research [22]. Hence, CA does not seem to inhibit RF changes, but seems to increase the risk of persistently lower RFs. Those findings support the hypothesis that lower and therefore possibly disadvantageous RF levels after CA are transferred forward from early to later adolescence [3, 23], which underpins the importance of revealing which factors and processes lend themselves best to aid optimal development after CA [3, 23].

In sum, our findings show that individual RFs change differently between early and later adolescence, but that the change pattern is similar in groups of CA+ and CA− adolescents. Based on those results, we cautiously suggest implications for future research, while reminding the reader that our findings only allow for group-level not individual-level conclusions. The main questions that arise from our mean-level findings are threefold. Firstly, one could ask whether RFs that seem to increase naturally during adolescence (e.g. distress tolerance) are particularly amenable and therefore more efficient intervention targets for reducing distress. Similarly, one may wonder whether it may be as advantageous to intervene on worsening RFs (e.g. rumination), to reduce or prevent such a decline. Regarding RFs that stay stable (e.g. friendships, family support and family cohesion), the arising question seems different. Stable RF levels may be advantageous for adolescents with a high level of those RFs, but may be disadvantageous for adolescents with a persistently low level of those RFs. Speculatively, stable RFs may function as a “vulnerability marker” when being persistently low, and early detection may be beneficial. Replication studies and translational research are crucially needed to answer these important questions, as such knowledge may eventually shed light on which RFs should be targeted in order to aid successful mental health development in adolescents with and without CA.

### RF interrelation changes

Despite the fact that the RF levels differed between the CA+ and the CA− group at both age 14 and 17, RF interrelations differed between the two groups only at age 14, not at age 17. This suggests that CA may have a more pronounced effect at age 14, as it then goes together with both differential RF levels and differential RF interrelations. One account could be proximity of CA, as CA was measured up to the age of 14. This would be in line with previous work suggesting that although CA has deleterious effects on mental health across the life course, it has a particularly strong effect on a shorter term and accordingly a decreasing effect on affective and behaviour disorders from childhood to young adulthood [2, 59].

Interestingly, on a *global* network structure level, taking the overall pattern of RF interrelations into account, both the CA+ and the CA− network were invariant between early and later adolescence. Moreover, neither the CA+ nor the CA− network changed in the degree to which RFs are expected to enhance each other (i.e. expected influence) between early and later adolescence. We believe that the lack of temporal changes on the *global* network level is unlikely to be explained by power, as we did detect a difference in expected influence in other comparisons (see example in the next paragraph). Moreover, on the *local* network structure level, we also identified only minor changes between early and later adolescence. In the CA+ network, one out of 45 possible RF interrelations turned more positive and one turned less positive between age 14 and 17 (see Additional file 15: Table S10), which may have cancelled each other out and thus may help explain why there was little change in the expected influence of the CA+ network. In the CA− network, none of the 45 RF interrelations changed significantly between age 14 and 17 (see Additional file 15: Table S10). Hence, those findings point towards a general stability of RF interrelations between early and later adolescence, in both the CA+ and the CA− network. If this would generalize to other cohorts, it may offer one account for the finding that CA often has lasting effects on mental health [1, 60].

Of note, those findings were slightly different for the RF networks which are not corrected for general distress (see Additional file 7), as those networks differed in positive connectivity between age 14 and age 17 in the CA+ group. At age 17, the CA+ network was significantly more positively interrelated than at age 14. This finding suggests that in the CA+ (not the CA−) group there is some improvement in the degree to which RFs can potentially enhance each other, between early and later adolescence. Yet, as this finding does not hold when we take general distress into account, the effect should be considered with caution.

For both the CA+ and the CA− network, at both age 14 and age 17, the family, brooding, and negative self-esteem RFs were most positively connected with the other RFs (for more details see Additional file 10). Hence, those RFs are potentially important in driving the positive connectivity of the RF networks and in underpinning the degree to which RFs can enhance each other. Interestingly, in terms of mean levels, the family RFs stayed stable in both groups, the brooding RF decreased in both groups and the negative self-esteem RF increased in the CA− group between age 14 and age 17. This suggests that (changes in) mean levels of RFs may not, or at least not directly, impact the degree to which the RFs can enhance other RFs. Thus, our RF mean level and RF network model analyses provide independent but complementary insights. To further improve knowledge about the clinical relevance of those indicators, future research needs to examine whether RF mean levels or RF interrelations characteristics (such as *expected influence* coefficients) are better predictors for subsequent mental health. Such knowledge needs to be obtained before our network findings can inform clinical research, as knowledge on the prediction magnitude is essential for picking promising RF targets for translational studies.

### Changes in pathways between RFs and general distress

Our findings showed that most RFs had direct negative pathways with distress, in both the CA+ and the CA− group, indicating that high RFs decrease distress, high distress decreases RFs, or both mutually influence each other. As all investigated RFs have empirically been shown to significantly decrease subsequent distress [8], it seems plausible that RF-distress pathways may not only over time, but also concurrently, operate as protective pathways. In the same vein, it is however also plausible that high distress reduces the protective effects of RFs (concurrently and/or over time). Such mutualistic coupling effects [61] need to be examined in future research. At both age 14 and 17, those potentially protective pathways appeared to be similarly strong in the two groups, regardless of solely investigating *direct* or also *indirect* pathways (i.e. via other RFs). Moreover, we did not detect differences between age 14 and 17, suggesting that RF-distress pathways seem stable between age 14 and 17.

Importantly, however, when taking our mean level findings into account—i.e. that the CA+ group had lower RFs and higher distress than the CA− group—a more elaborate interpretation emerges. That is, despite the fact that RF-distress pathways seem on the first glance to be similarly protective in the two groups, the combination of lower RFs and higher distress in the CA+ group supports the notion that RF-distress pathways operate on a different, and presumably more disadvantageous, mean level than in the CA− group. As lower RFs, higher distress, and potentially disadvantageous RF-distress pathways seemed to be rather stable from early to later adolescence, this may be another account for why exposure to CA is frequently found to not only have a short-term but also a longer-lasting impact on mental health [1, 60].

The four RFs that were most strongly interrelated with distress, in both the direct and the shortest pathway models, were negative self-esteem, brooding, aggression, and friendship support. Interestingly, the first two of those RFs were also among the RFs being most positively connected with the other RFs, in both groups and at both ages. Hence, if replication of our findings would hold, the negative self-esteem and brooding RFs may be of particular interest for future prediction studies, as they not only seem to have the highest potential of increasing other RFs, but also seem to have the highest potential in reducing distress, and therefore may also have a high potential in reducing subsequent mental health problems.

### Limitations

Our research has several limitations. First, CA was assessed with retrospective caregiver report, which may be inaccurate due to for example limited recall, limited knowledge, or embarrassment. To enhance recall, caregivers were encouraged to use assisting material (e.g. photo albums) [27], and an event timeline (with the following time windows: 0–5, 5–11, 11–14) was established. Second, the family support and family cohesion RFs were derived from one questionnaire, which may have resulted in more similar response patterns in those RFs. The same argument goes for rumination (reflection and brooding) and self-esteem (high positive and low negative self-esteem) RFs. Third, to enable RF comparisons over time, we had to equate multiple LCFA parameters between age 14 and age 17. This may disadvantage the model accuracy and therefore potentially increase bias in the resulting factor scores. To circumvent this limitation as best we could, we used the least restricted models possible to still meet the assumptions of the respective network and mean change analyses. However, this meant that we could not use the exact same factor scores for the network and the mean change analyses. For completeness, we re-ran the mean change analyses with factor scores derived from the LCFAs that we used for the network analyses (see Additional file 6). Fourth, we interpret negative interrelations between RFs in networks that take general distress into account as disadvantageous. However, as our models are undirected, we cannot disentangle whether the general distress variable behaved as intended as a confounder, or against our expectation as a collider [62], falsely inducing or enhancing these interrelations (for a detailed discussion see Supplement XIII in [17]). Fifth, we performed the network models with regularized partial correlations, which currently is the default method. However, recently, other approaches have been suggested such as non-regularized methods [63]. Future research will need to show which methods tend to be most suitable for psychometric network models. Sixth, as our study contains two time points, we cannot draw conclusions with regard to tipping points or specifically sensitive periods. Likewise, we cannot examine how RFs change from prior to post CA, as we did not assess the RFs prior to CA. Seventh, we used imputation methods to include participants with missing information. Yet, when we pooled the factor model results for the imputed data sets together, we revealed for some models a negative pooled chi-square. As relative fit indices cannot be calculated based on a negative chi-square, the chi-squares had to be set to zero, resulting in arbitrary chi-square-dependent (“relative”) pooled fit indices. To enable the reader to judge the various models (i.e. being based on the different imputed data sets), we provide a chi-square-independent (“absolute”) fit index pooled over the separate models (i.e. the standardized root mean residual) and provide chi-square-dependent (“relative”) fit indices separately for the models. Eighth, it would have been valuable to explore gender effects (e.g. as in [64]); however, for many of the analyses, we may not have had enough power to split the sample additionally with regard to gender. Ninth, the ROOTS participants had on average a slightly higher SES than the average UK population and generalizations may therefore be most valid for above average SES populations [26].

Regarding the question whether resilience and risk factors are opposing sides of the same coin, the quick, but insufficient, answer for our study is probably that many (or most) of the investigated RFs are indeed the flip side of risk factors. For example, self-esteem (or a positive self-concept) is commonly defined as RF and has been discussed as such by many of the seminal resilience researchers, including Michael Rutter, Emmy Werner, Ann Masten, and Michael Ungar (for a review see, e.g. [65]). Yet, at the same time, a low level of self-esteem or self-worth is part of the DSM V criteria for depression (“Feelings of worthlessness”; American Psychiatric Association [66]). Hence, whereas a high level of self-esteem may protect against low mood levels, low self-esteem is assumed to contribute to or reflect low mood. As doing this question fully justice is out of the scope of this discussion, we added a more detailed debate on the question to Additional files 16 and 17. Importantly however, regardless of whether resilience and risk factors operate on the same continuum or are inversely correlated but not identical, understanding the nature of RFs seems to have universal appeal as it focuses on what promotes good mental health rather than on what increases mental health problems.

## Conclusion

Our results support several prior conjectures regarding changes in RF mean levels, for example that lower and therefore disadvantageous levels of RFs are likely to be carried forward over time in adolescents with prior exposure to CA. Our findings also contribute novel hypotheses: for example, they suggest that RF changes are similar in adolescents with and without CA and that inter-personal mean levels may stay stable, whereas some intra-personal RFs change between early and later adolescence. On a network level, CA seemed to have a stronger proximal effect, as RF interrelations differed between the two groups at age 14, but not at age 17. RF-distress pathways seemed to have similarly protective strengths in both groups, during early and later adolescence. Yet, as RFs are lower and distress is higher in the CA+ group, we cautiously suggest that RF-distress pathways may overall be less advantageous than in the CA− group. As lower RFs, higher distress, and potentially disadvantaged pathways between RFs and distress seemed to be carried forward from early to later adolescence, our findings may help explain why exposure to CA is frequently found to have a lasting impact on mental health. To pinpoint the clinical relevance of our findings, we commend future research to examine whether (a) RF mean levels, (b) RF interrelations coefficients, or (c) RFs that score high on both indicators offer the best prediction for subsequent mental health and thus lend themselves best for formulating translational hypotheses. In sum, our study not only sheds light on the complex nature and changes of ten empirically supported RFs between early and later adolescence, but also offers tentative accounts for why CA has strong proximal effects and is often found to have a lasting impact on mental health.

## Supplementary information

**Additional file 1.** Rationale for changes in variables since the previous report.

**Additional file 2.** Network models this time excluding the brooding variable.

**Additional file 3.** Overview of used R packages, including their version number and reference.

**Additional file 4.** Missing data patterns and missingness predictors.

**Additional file 5.** Part A: Rationale for using factor scores, instead of sum scores. Part B: Model specifications and model fit for the three estimated invariance levels of the categorical longitudinal confirmatory factor analyses for the resilience factors and the distress index, as well as box-and-whisker plots with individual data points for the resulting factor scores.

**Additional file 6.** Mean change analyses with (a) fully invariant factor scores and (b) sum scores.

**Additional file 7.** RF network results without the general distress variable as well as RF network results with the general distress variable.

**Additional file 8.** Correlations and regularized partial correlations between the RFs and the general distress factor.

**Additional file 9.** The stability of the expected influence (EI) coefficients and the accuracy of the ‘RF-RF’ and ‘RF-general distress’ interrelations.

**Additional file 10.** Expected influence (EI) for RFs in networks corrected for general distress.

**Additional file 11.** Network analysis results conducted with factor scores derived from the configurable LCFA models.

**Additional file 12.** Network analysis results conducted with sum scores.

**Additional file 13.** Network models presented in the main manuscript and in Additional file 7 with faded interrelations.

**Additional file 14.** Similarity and differences to Figures in a previous report on this sample.

**Additional file 15.** Significant RF-RF interrelation differences (a) between the CA+ (n = 631) and the CA- (n = 499) networks, as well as (b) between age 14 and age 17 networks.

**Additional file 16.** Debate: Are resilience and risk factors opposing sides of the same coin?

**Additional file 17.** References for the additional files.

**Additional file 18.** Supplementary materials: Analysis results based on imputed data.

## Data Availability

*Data availability*: Data for this specific paper has been uploaded to the Cambridge Data Repository 10.17863/CAM.36708 and is password protected. Our participants did not give informed consent for their measures to be made publicly available, and it is possible that they could be identified from this data set. Access to the data supporting the analyses presented in this paper will be made available to researchers with a reasonable request to openNSPN@medschl.cam.ac.uk. *Code availability*: Analysis code is available from http://jessica-fritz.com/.

## Abbreviations

RFs: Resilience factors
CA: Childhood adversity
