# Supplementary material for: Unravelling the complex nature of resilience factors and their changes between early and later adolescence
Source: BMC Med. 2019 Nov 14;17:203. doi: 10.1186/s12916-019-1430-6 (PMC6854636; doi:10.1186/s12916-019-1430-6)
Supplement: Supplementary file 1 — Additional file 1. Rationale for changes in variables since the previous report. [file 12916_2019_1430_MOESM1_ESM.pdf]

Additional file I

In our previous report[1] we used a distress index computed by Brodbeck and colleagues (2011)[2]. This index was however only computed for age 14. As we here wanted to compare two occasions, we decided to compute a separate general distress factor including information for both occasions, so that we could apply invariance constraints over the two time points. This was for example necessary to compare the latent means. Moreover, in comparison to Brodbeck and colleagues (2011)[2] we simplified the index (e.g. through only using the short form rather than the complete version of the used depression scale), to ensure feasibility of the computation of the invariance models (particularly because of the increased computation time with regard to the multiple imputed data sets).

As the rumination questionnaire underwent revision between our two occasions, different versions of the questionnaire were used at the two occasions. The ruminative reflection factor stayed the same for both time points, however, we had to use another ruminative brooding factor than in Fritz, Fried et al. (2018)[1] as only two of the five originally used items were available for both occasions. Here we report results based on a ruminative brooding approach that has been established by Burwell and Shirk (2007; 6 items)[3] for a version of the rumination questionnaire that matches the one used at our second occasion, as five of the six identified items of that approach were available for both of our occasions. We also analysed the network models excluding the brooding variable, which revealed similar results, see Additional file II.
