# Supplementary material for: Unravelling the complex nature of resilience factors and their changes between early and later adolescence
Source: BMC Med. 2019 Nov 14;17:203. doi: 10.1186/s12916-019-1430-6 (PMC6854636; doi:10.1186/s12916-019-1430-6)
Supplement: Supplementary file 2 — Additional file 2. Network models this time excluding the brooding variable. [file 12916_2019_1430_MOESM2_ESM.pdf]

Additional file II

The following three figures depict CA+ and CA- networks as presented in the main manuscript, however this time excluding the brooding variable. The models were estimated separately for age 14 and 17, as well as (1) once without the general distress variable, (2) once with the general distress variable, and (3) once corrected for the general distress variable. At age 14, the network invariance test was not significant for the networks without the general distress variable (M = .14, p = .41; see Figure 1), but the global network expected influence differed between the CA+ and the CA- RF networks (EI_{CA+} = 2.59, EI_{CA-} = 2.96, EI = 0.37, p = .02). More specifically, the CA+ network was less positively connected than the CA- network. Those findings were only partially similar in the networks for age 17, as neither of the two tests revealed significant differences between the CA+ and the CA- group (M = .12, p = .64; EI_{CA+} = 2.93, EI_{CA+} = 2.79, EI = 0.14, p = .31). When we compared the RF networks for age 14 and age 17, the two CA+ network were not invariant over time, in other words, they did vary over time (M = .23, p = .002). Moreover, the RFs in the age 14 network were less positively interrelated than in the age 17 network (EI_{14} = 2.59, EI_{17} = 2.93, EI = 0.34, p = .003). The age 14 and age 17 CA- networks did however not differ with regard to their global network structure (M = .15, p = .45; EI_{14} = 2.96, EI_{17} = 2.79, EI = 0.17, p = .26).

For the networks with the general distress variable, the network invariance test (M = .19, p = .12; see Figure 2) was not significant at age 14. However, the global network expected influence differed significantly between the CA+ and the CA- networks (EI_{CA+} = -0.19, EI_{CA-} = 0.75, EI = 0.94, p < .01). Those findings were only partially similar in the networks for age 17, as neither of the two tests revealed significant differences between the CA+ and the CA- group (M = .15, p = .51; EI_{CA+} = 0.13, EI_{CA-} = 0.55, EI = 0.42, p = .19). When we compared the networks for age 14 and age 17, we did not find any significant global network structure differences; neither for adolescents with (M = .15, p = .19; EI_{14} = -0.19, EI_{17} = 0.13, EI = 0.32, p = .35) nor for adolescents without a history of adversity (M = .14, p = .62; EI_{14} = 0.75, EI_{17} = 0.55, EI = 0.20, p = .50).

For the networks corrected for the general distress variable, the network invariance test for the CA+ and the CA- group (M = .19, p = .10; see Figure 3) was not (or only very marginally) significant, at age 14. In contrast, the global network expected influence differed between the CA+ and the CA- networks (EI_{CA+} = 1.36, EI_{CA-} = 2.09, EI = 0.73, p = .001). Those findings were again only partially similar in the networks for age 17, as neither of the two tests revealed significant differences between the CA+ and the CA- group (M = .12, p = .82; EI_{CA+} = 1.69, EI_{CA-} = 1.92, EI = 0.24, p = .27). When we compared the networks for age 14 and age 17, we once more did not find any

significant global network structure differences; neither for adolescents with (M = .12, p = .53; EI_{14} = 1.36, EI_{17} = 1.69, EI = 0.33, p = .17) nor for adolescents without a history of adversity (M = .14, p = .58; EI_{14} = 2.09, EI_{17} = 1.92, EI = 0.17, p = .41).

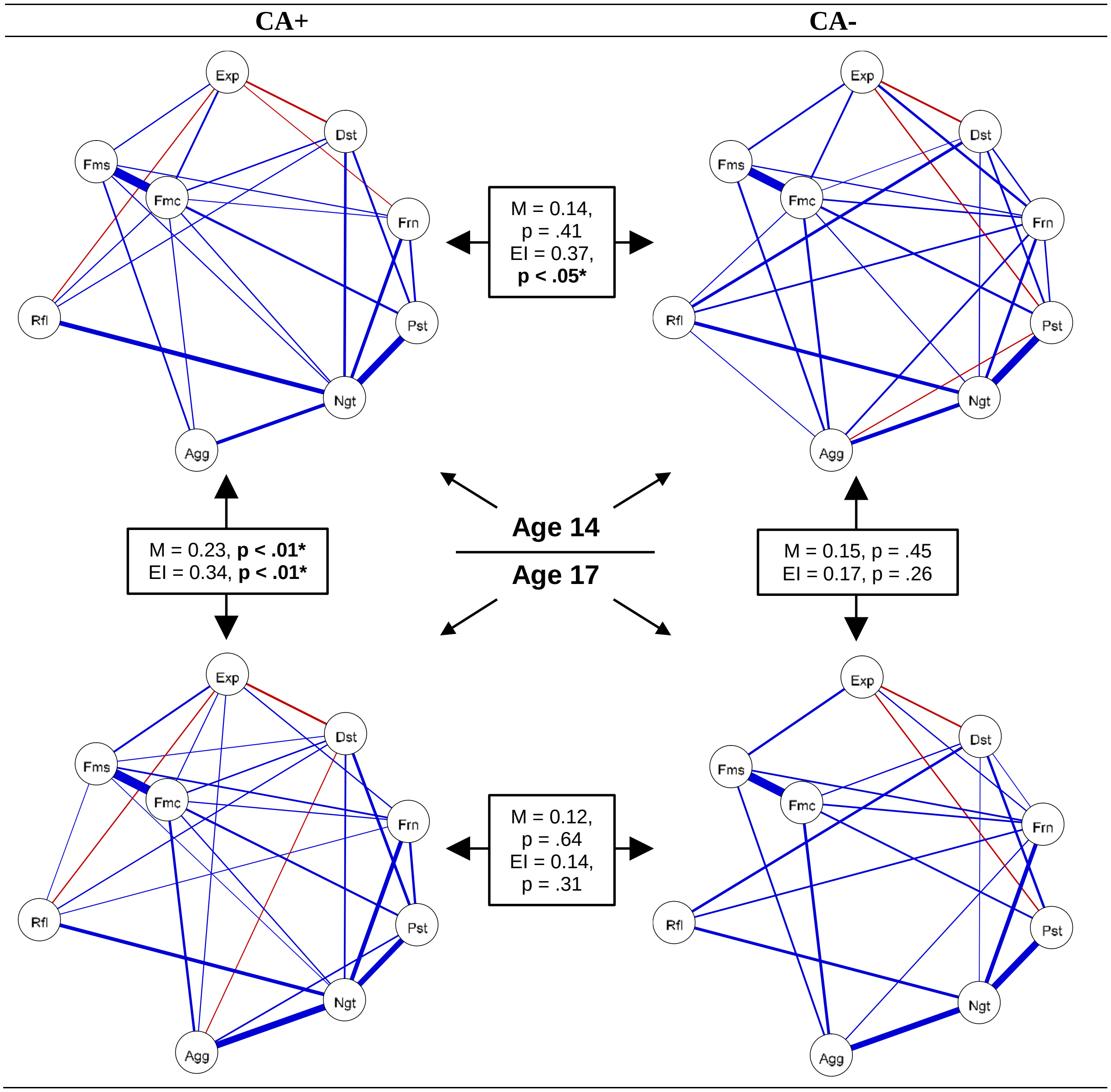

Figure 1. CA+ (*n* = 631) and CA- (*n* = 499) resilience factor networks for age 14 (upper panel) and age 17 (lower panel) without the brooding and the general distress variable. Width of the lines = association strength. Positive interrelations = blue, negative interrelations = red. **Legend:** Frn = friend support, fms = family support, fmc = family cohesion, ngt = negative self-esteem, pst = positive self-esteem, rfl = reflection, dst = distress tolerance, agg = aggression, exp = expressive suppression. The boxes depict the maximal interrelation difference between the respective two networks (M), the difference in global network expected influence (EI) between the respective two networks (EI), and the corresponding p-values (5000 comparison samples).

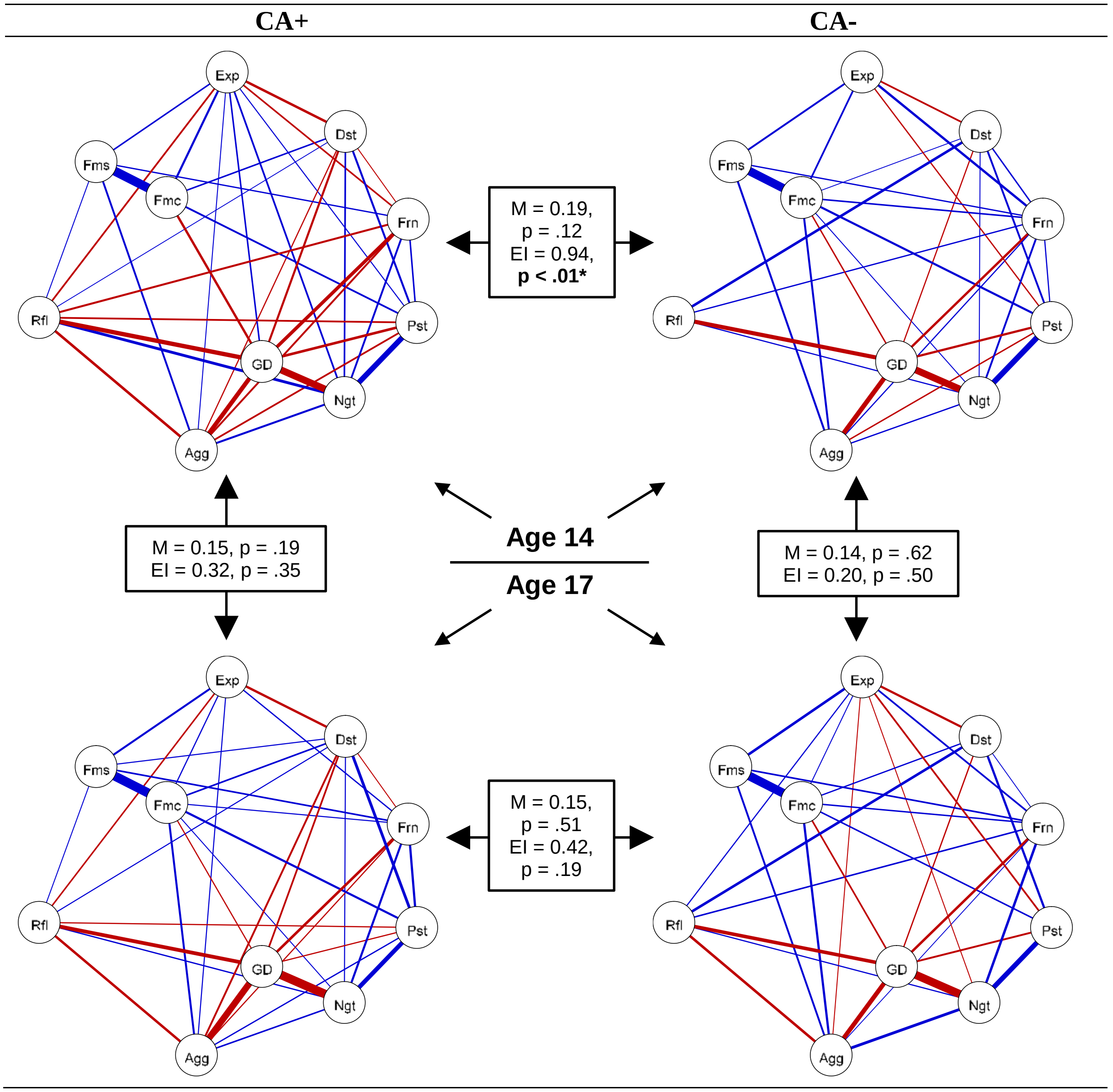

Figure 2. CA+ (*n* = 631) and CA- (*n* = 499) resilience factor networks for age 14 (upper panel) and age 17 (lower panel) without the brooding variable, but with the general distress variable. Width of the lines = association strength. Positive interrelations = blue, negative interrelations = red. **Legend:** Frn = friend support, fms = family support, fmc = family cohesion, ngt = negative self-esteem, pst = positive self-esteem, rfl = reflection, dst = distress tolerance, agg = aggression, exp = expressive suppression, GD = general distress. The boxes depict the maximal interrelation difference between the respective two networks (M), the difference in global network expected influence (EI) between the respective two networks (EI), and the corresponding p-values (5000 comparison samples).

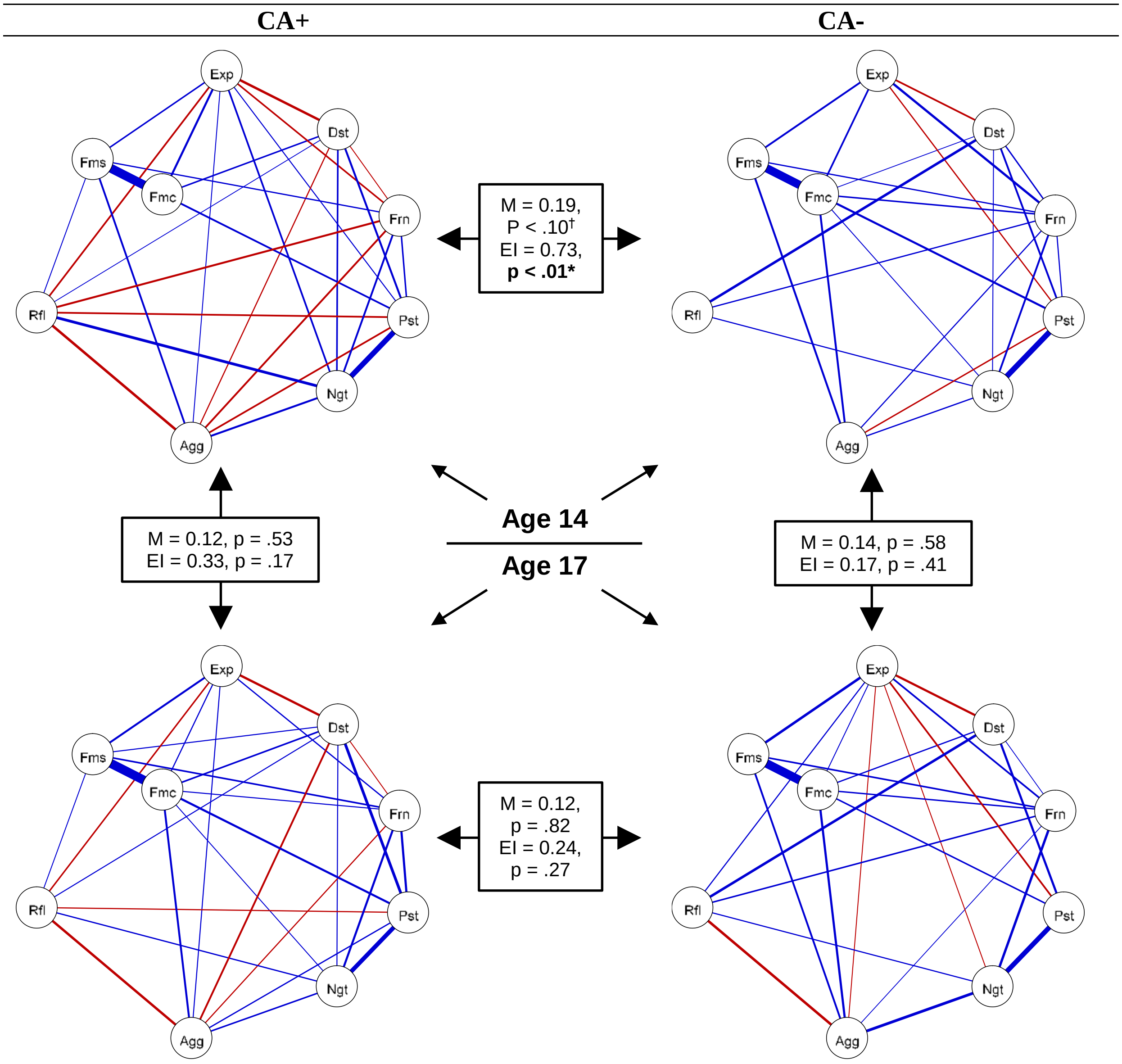

Figure 3. CA+ (n = 631) and CA- (n = 499) resilience factor networks for age 14 (upper panel) and age 17 (lower panel) without the brooding variable and corrected for the general distress variable. Width of the lines = association strength. Positive interrelations = blue, negative interrelations = red. **Legend:** Frn = friend support, fms = family support, fmc = family cohesion, ngt = negative self-esteem, pst = positive self-esteem, rfl = reflection, dst = distress tolerance, agg = aggression, exp = expressive suppression. The boxes depict the maximal interrelation difference between the respective two networks (M), the difference in global network expected influence (EI) between the respective two networks (EI), and the corresponding p-values (5000 comparison samples).
