# Supplementary material for: Unravelling the complex nature of resilience factors and their changes between early and later adolescence
Source: BMC Med. 2019 Nov 14;17:203. doi: 10.1186/s12916-019-1430-6 (PMC6854636; doi:10.1186/s12916-019-1430-6)
Supplement: Supplementary file 3 — Additional file 3. Overview of used R packages, including their version number and reference. [file 12916_2019_1430_MOESM3_ESM.pdf]

Additional file III

We conducted all analyses in R version 3.5.1, and used the below packages (see Table 1), and further dependencies these packages load.

Table 1

Used R packages, including their version number and reference

**Package (version number)**	**Reference**
mice (3.5.0)	van Buren, S. & Groothuis-Oudshoorn K. (2011)[4]
dplyr (0.7.7)	Wickham, H., François, R., Henry, L. & Müller (2018)[5]
pastecs (1.3.21)	Grosjean, P. & Ibanez, F (2018)[6]
coin (1.2-2)	Hothorn, T., Hornik, K., van de Wiel, M. A. & Zeileis, A (2008)[7]
reshape (0.8.8)	Wickham, H (2007)[8]
sjPlot (2.6.2)	Lüdecke, D (2018)[9]
lavaan (0.6-4)	Rosseel, Y (2012)[10]
semTools (0.5-1.933)	Jorgensen, T. D., Pornprasertmanit, S., Schoemann, A. M. & Rosseel, Y (2018)[11]
ggplot2 (3.1.0)	Wickham, H (2016)[12]
qgraph (1.5)	Epskamp, S., Cramer, A. O. J., Waldorp, L. J., Schmittmann, V. D. & Borsboom, D (2012)[13]
bootnet (1.1.0)	Epskamp, S., Borsboom, D. & Fried, E. I (2018)[14]
NetworkComparisonTest (2.0.1)	van Borkulo, C. D (2018)[15]
