# Supplementary material for: Unravelling the complex nature of resilience factors and their changes between early and later adolescence
Source: BMC Med. 2019 Nov 14;17:203. doi: 10.1186/s12916-019-1430-6 (PMC6854636; doi:10.1186/s12916-019-1430-6)
Supplement: Supplementary file 4 — Additional file 4. Missing data patterns and missingness predictors. [file 12916_2019_1430_MOESM4_ESM.pdf]

Additional file IV

We evaluated differences between those participants who had full data and participants who had missingness, either due to attrition or incidental missingness, as stipulated in Table 2. All RFs and the general distress variable had a small number of participants with entire missingness at both time points (50 to 78 participants). A minor subset of participants did not provide data at occasion1, but at occasion 2 for some of the scales (10 to 30 participants per scale). There was more attrition for people who provided data at occasion 1 but not at occasion 2 (123 to 294 per scale). There was a range of participants with incidentally missing items, which differed largely per scale (from 4 for aggression to 152 for general distress).

We also investigated whether the missingness was predictable (see Table 3). We identified that missingness on all RFs and general distress could be explained by CA. Moreover, for seven RFs and distress missingness was also explained by being male and by having a low mood (MFQ levels). A psychiatric history prior to the age of 14 explained additionally missingness in six RFs and general distress. Overall, we excluded 50 participants who had more than 85% of missing items across the scales, which resulted in 1188 remaining participants. Among those 1188 all had less than 59% missing items. On average the items had 9% missingness.

Table 2

Missingness patterns (N= 1238)

Variable	No data	Full data	Missing T2	Missing T1	Incidental M
FRN	051	941	192	030	024
FMS	054	915	190	028	051
FMC	054	917	190	028	049
PST	050	955	180	027	026
NGT	050	961	180	027	020
BRD	050	1004	123	029	032
RFL	050	1000	123	030	035
DST	073	849	294	010	012
AGG	050	975	180	029	004
EXP	078	854	292	014	000

GD	050	830	179	027	152

Note. Incidental M. = incidental missingness, FRN = friendship support, FMS = family support, FMC = family cohesion, PST = positive self-esteem, NGT = negative self-esteem, BRD = brooding, RFL = reflection, DST = distress tolerance, AGG = aggression, EXP = expressive suppression, GD = general distress.

Table 3

Missingness predictors

		U/ χ^2 (df)	*p*	Cross tabs<br>(1 = no missing; 2 = missing)
GD	CA	7.0557 (1)	<0.01**	1 2<br>CA- 374 127<br>CA+ 429 209
	Gender	5.9034 (1)	<0.05*	1 2<br>female 458 216<br>male 345 219
	MFQ	123280	<0.001***	1 M: 15.3300<br>2 M: 17.8711485
	Age-14 prior psychiatric history (PP; yes = PP+; no = PP-)	5.6529 (1)	<0.05*	1 2<br>PP- 623 286<br>PP+ 180 116
FRN	CA	11.361 (1)	<0.001***	1 2<br>CA- 423 78<br>CA+ 486 152
	Gender	10.95 (1)	<0.001***	1 2<br>female 521 153<br>male 388 176
	MFQ	103140	<0.05*	1 M = 15.7665<br>2 M = 17.3571429
	Age-14 PP	3.9534 (1)	<0.05*	1 2<br>PP- 699 210<br>PP+ 210 86
FMS	CA	20.05 (1)	<0.001***	1 2<br>CA- 421 80<br>CA+ 464 174
	Gender	3.9295 (1)	<0.05*	1 2<br>female 498 176<br>male 387 177
	MFQ	109500	<0.001***	1 M: 15.5386<br>2 M: 17.8088737
	Age-14 PP	5.8008 (1)	<0.05*	1 2<br>PP- 684 225<br>PP+ 201 95
FMC	CA	19.544 (1)	<0.001***	1 2<br>CA- 421 80<br>CA+ 465 173
	Gender	4.7008 (1)	<0.05*	1 2<br>female 500 174<br>male 386 178
	MFQ	109270	<0.001***	1 M: 15.5271<br>2 M: 17.843003
	Age-14 PP	5.9935 (1)	<0.05*	1 2<br>PP- 685 224<br>PP+ 201 95
PST	CA	7.9443 (1)	<0.01**	1 2<br>CA- 425 76<br>CA+ 498 140
	Gender	12.51 (1)	<0.001***	1 2

				female 530 144<br>male 393 171
	MFQ	102010	0.094	1 M: 15.8959<br>2 M: 16.9495798
	Age-14 PP	3.736 (1)	0.053	1 2<br>PP- 709 200<br>PP+ 214 82
NGT	CA	10.722 (1)	<0.01**	1 2<br>CA- 430 71<br>CA+ 498 140
	Gender	10.222(1)	<0.01**	1 2<br>female 530 144<br>male 398 166
	MFQ	99465	0.062	1 M: 15.8263<br>2 M: 17.2489270
	Age-14 PP	1.8092 (1)	0.18	1 2<br>PP- 709 200<br>PP+ 219 77
AGG	CA	11.568 (1)	<0.001***	1 2<br>CA- 436 65<br>CA+ 505 133
	Gender	18.327 (1)	<0.001***	1 2<br>female 562 112<br>male 413 151
	MFQ	84244	0.13	1 M: 15.9066<br>2 M: 17.1881720
	Age-14 PP	2.3499 (1)	0.13	1 2<br>PP- 745 164<br>PP+ 230 66
BRD	CA	9.5907 (1)	<0.01**	1 2<br>CA- 446 55<br>CA+ 525 113
	Gender	4.2525 (1)	<0.05*	1 2<br>female 544 130<br>male 427 137
	MFQ	83354	<0.05*	1 M: 15.8412<br>2 M: 17.4947368
	Age-14 PP	1.4102 (1)	0.24	1 2<br>PP- 740 169<br>PP+ 231 65
RFL	CA	7.2547 (1)	<0.01**	1 2<br>CA- 442 59<br>CA+ 525 113
	Gender	3.7544 (1)	0.053	1 2<br>female 541 133<br>male 426 138
	MFQ	83594	<0.05*	1 M: 15.8002<br>2 M: 17.6649485
	Age-14 PP	1.8251 (1)	0.18	1 2<br>PP- 738 171<br>PP+ 229 67
DST	CA	13.384 (1)	<0.001***	1 2<br>CA- 399 102<br>CA+ 446 192
	Gender	0.29893 (1)	0.59	1 2<br>female 465 209<br>male 380 184
	MFQ	112880	<0.001***	1 M: 15.2343<br>2 M: 18.3012048
	Age-14 PP	8.6094 (1)	<0.01**	1 2<br>PP- 658 251<br>PP+ 187 109

EXP	CA	20.024 (1)	<0.001***	1 2<br>CA- 407 94<br>CA+ 443 195
	Gender	0.47049 (1)	0.49	1 2<br>female 471 203<br>male 383 181
	MFQ	111640	<0.001***	1 M: 15.2452<br>2 M: 18.3487654
	Age-14 PP	9.8232 (1)	<0.01**	1 2<br>PP- 666 243<br>PP+ 188 108

Note. CA = childhood adversity, MFQ = Mood and Feelings Questionnaire, FRN = friendship support, FMS = family support, FMC = family cohesion, PST = positive self-esteem, NGT = negative self-esteem, BRD = brooding, RFL = reflection, DST = distress tolerance, AGG = aggression, EXP = expressive suppression, GD = general distress.
