# Supplementary material for: Unravelling the complex nature of resilience factors and their changes between early and later adolescence
Source: BMC Med. 2019 Nov 14;17:203. doi: 10.1186/s12916-019-1430-6 (PMC6854636; doi:10.1186/s12916-019-1430-6)
Supplement: Supplementary file 5 — Additional file 5. Part A: Rationale for using factor scores, instead of sum scores. Part B: Model specifications and model fit for the three estimated invariance levels of the categorical longitudinal confirmatory factor analyses for the resilience factors and the distress index, as well as box-and-whisker plots with individual data points for the resulting factor scores. [file 12916_2019_1430_MOESM5_ESM.pdf]

Additional file V

Part A. We decided to use factor scores, instead of sum scores, for two reasons. Firstly, to remove as much measurement error as possible from the latent resilience factor (RF) variables. In most published network analysis manuscripts author have used item level data. Yet, here we were not interested in the individual items but in RF constructs which were derived from a previous systematic review. As all RFs (except for expressive suppression) consisted of more than 3 items we could apply factor analyses to effectively reduce measurement error. A similar method would have been to use latent network modelling, which does the same but estimates the factor scores and the network models in one step.[16] Upon closer inspection we concluded that latent network modelling is as yet only (or at least particularly) applicable to smaller models. The second reason for using factor scores was that when using sum scores one assumes that all items have the same importance and hence go with the same weight into the construct (i.e. tau equivalence). However, when using factor scores, the factor loadings enable every item to have a unique weight for the latent construct, which means that items can differ in importance, enhancing construct validity. We felt that this point was particularly important as many of our used (sub-)scales did not consist of a large number of items (with exception for the general distress factor).

For completeness, we additionally performed our analyses based on sum scores. We added the results for mean change analyses with sum scores to Supplement Vb and the results for network analyses with sum scores to Supplement XV. The sum score results were overall similar to the results for fully invariant factor scores.

Part B. As we aimed to compare two time points, we estimated longitudinal CFAs (LCFAs) separately for each RF and the general distress variable. Given that all of the RF items (as well as the general distress items) were assessed with three to six answer categories, we computed categorical LCFAs and treated the items as ordinal (i.e. ordered categorical) indicators.[17–20] Accordingly, we used the weighted least squares mean and variance adjusted (WLSMV) estimator. The categorical LCFAs were specified as shown in Figure 4 (which is modelled along examples of [17]). We identified the model as suggested by Wu and Estabrook[20], using the theta parametrization. We estimated, a configural (i.e. baseline) model, a strong invariance and a full invariance model. For the strong invariance LCFAs we equated item loadings and item thresholds across the two time points (i.e. age 14 and 17), fixed all item intercepts to 0, the item variance of the first time point to 1, the latent factor mean of the first time point to 0, and the latent factor variance of the first time point to 1 (item covariances and the latent factor covariance were freely estimated). For the full invariance LCFAs we again equated item loadings and item thresholds across the

two time points (i.e. age 14 and 17) and fixed all item intercepts to 0, this time however we fixed all item variances to 1, both latent factor means to 0, and both latent factor variances to 1 (item covariances and the latent factor covariance were again freely estimated). A model specification overview can be found in Table 4. Table 5 depicts the fit indices for all models. We only applied modification indices when they were theoretically justified. We intended to pool over the fit indices of the 10 result sets (i.e. one for each imputation data set). We however discovered that for many of our models the pooling of fit indices resulted in either a negative or a close to negative chi-square statistics. When the chi-square statistic is negative, it needs to be set to zero, as the pooled fit otherwise cannot be computed. Unfortunately, a zero chi-square results in an arbitrary model fit. Therefore, we decided to report the fit indices of the separate models, which we consider more informative in this case (see Table 5). We do additionally provide the pooled standardized root mean residual (pooled SRMR) as this fit measure does not rely on the chi-square statistic (i.e. it represents the standardized difference between the observed and the predicted correlation) and is therefore reliable for our models. All models seemed to fit acceptably. Factor scores derived from the aggression models were however so poorly distributed that we had to binarize those scores. Distribution plots (i.e. box-and-whisker plots with individual data points) for the RFs (except for expressive suppression and aggression) and the general distress variable are depicted in Figure 5.

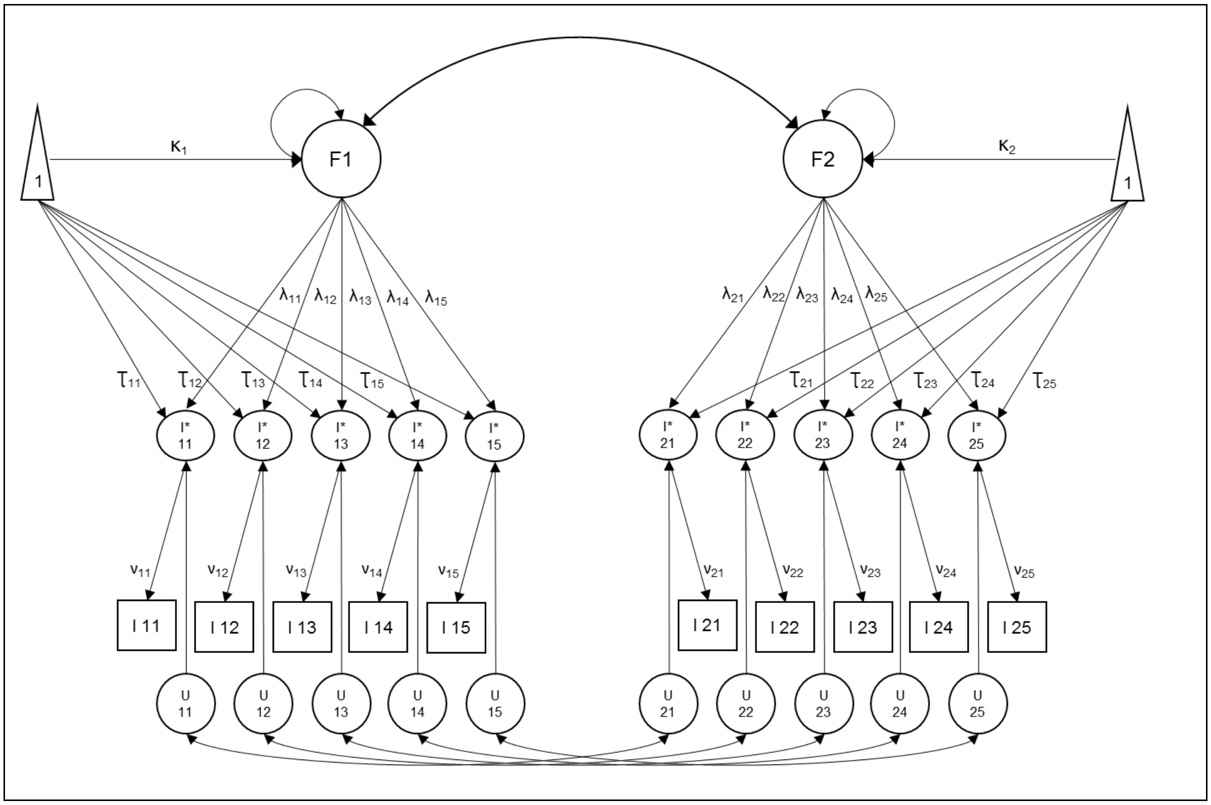

Figure 4. Longitudinal, categorical CFA model, with 5 categorical items assessed at two time points. The model is defined as follows: F = **common latent factor** (with the factor number indicating the corresponding time point); κ = **common latent factor means** (with the subscript number indicating the corresponding latent factor); I = **categorical observed items**; I* = **continuous latent item responses** inferred from the categorical observed items and described by the item thresholds; λ = **item loadings**; τ= **item intercepts**; ν = **item thresholds** (the number of thresholds is not depicted as every ordinal item has multiple thresholds, namely one less than the number of measured categories); U = **unique latent (item) factors;** for indicators with two numbers, the first number refers to the time point and the second to the item number; ↔ = two-sided arrows indicate (auto-co)variances. The Figure is modelled along examples of Liu, Millsap, West, Tein, Tanaka and Grimm [17]: https://doi.org/10.1037/met0000075.

Table 4

Model specifications for the three estimated invariance levels of the categorical LCFAs

(1) Configural Model

Estimated parameters:

1. λ = **factor loadings:** all freely estimated
2. ν = **items thresholds:** all freely estimated
3. auto-covar(U) = **unique latent (item) factor auto-covariances:** between the corresponding time 1 and time 2 unique latent (item) factors are all freely estimated

4. auto-covar(F) = **common latent factor auto-covariance:** between the corresponding time 1 and time 2 common latent factor is freely estimated

Parameters fixed for both time points:

1. τ = **item intercepts:** all fixed to zero
2. var(U) = **unique latent (item) factor variances**: are all fixed to one
3. κ = **common latent factor means:** all fixed to zero
4. var(F) = **common latent factors variances**: are all fixed to one

Parameters fixed for only the first but estimated for the second time point:

-

Equated parameters across time:

-

---

(2) Strong Invariance Model

Estimated parameters:

1. auto-covar(U) = **unique latent (item) factor auto-covariances:** between the corresponding time 1 and time 2 unique latent (item) factors are all freely estimated
2. auto-covar(F) = **common latent factor auto-covariance:** between the corresponding time 1 and time 2 common latent factor is freely estimated

Parameters fixed for both time points:

1. τ = **item intercepts:** all fixed to zero

Parameters fixed for only the first but estimated for the second time point:

1. var(U) = **unique latent (item) factor variances:** fixed to one only for the first, but not the second time point
2. κ = **common latent factor means:** fixed to zero only for the first, but not the second time point
3. var(F) = **common latent factor variances:** fixed to one only for the first, but not the second time point

Equated parameters across time:

1. λ = **factor loadings:** all equated across time
2. ν = **items thresholds:** all equated across time

---

(3) Full Invariance Model		
Estimated parameters:		
1.	auto-covar(U)	= **unique latent (item) factor auto-covariances:** between the corresponding time 1 and time 2 unique latent (item) factors are all freely estimated
2.	auto-covar(F)	= **common latent factor auto-covariance:** between the corresponding time 1 and time 2 common latent factor is freely estimated
Parameters fixed for both time points:		
1.	τ	= **item intercepts:** all fixed to zero
2.	var(U)	= **unique latent (item) factor variances:** all fixed to one
3.	κ	= **common latent factor means:** all fixed to zero
4.	var(F)	= **common latent factor variances:** all fixed to one
Parameters fixed for only the first but estimated for the second time points:		
-		
Equated parameters across time:		
1.	λ	= **factor loadings:** all equated across time
2.	ν	= **items thresholds:** all equated across time

Table 5

Longitudinal, Categorical Confirmatory Factor Analyses Conducted with the WLSMV Estimator

**Model**	**CFI**	**TLI**	**RMSEA**	**RMSEA 90% CI**	**RMSEA 90% CI**	**Chi²**
Friendship support [21], 5 items, 0 unique item covariances						
CM-1	0.976	0.962	0.079	0.070	0.089	244.996
CM-2	0.975	0.961	0.086	0.077	0.096	285.431
CM-3	0.982	0.973	0.083	0.074	0.092	264.909
CM-4	0.974	0.960	0.086	0.077	0.095	283.386
CM-5	0.978	0.965	0.085	0.076	0.094	275.672
CM-6	0.974	0.959	0.083	0.074	0.093	268.033
CM-7	0.979	0.967	0.076	0.067	0.085	227.553
CM-8	0.979	0.967	0.080	0.071	0.089	247.474
CM-9	0.975	0.960	0.084	0.075	0.093	270.736

CM-10	0.967	0.949	0.090	0.081	0.099	307.232
CM1-10	Pooled SRMR: 0.057					
SIM-1	0.975	0.974	0.066	0.058	0.073	270.276
SIM-2	0.971	0.971	0.075	0.067	0.082	335.870
SIM-3	0.980	0.979	0.072	0.065	0.079	313.837
SIM-4	0.972	0.971	0.073	0.065	0.080	319.411
SIM-5	0.977	0.976	0.070	0.063	0.078	301.303
SIM-6	0.973	0.972	0.069	0.061	0.076	290.286
SIM-7	0.976	0.975	0.066	0.058	0.073	268.221
SIM-8	0.973	0.972	0.073	0.065	0.080	319.566
SIM-9	0.973	0.972	0.071	0.063	0.078	303.678
SIM-10	0.966	0.965	0.074	0.067	0.082	331.894
SIM1-10	Pooled SRMR: 0.057					
FIM-1	0.975	0.978	0.061	0.054	0.068	275.396
FIM-2	0.968	0.972	0.073	0.066	0.080	374.584
FIM-3	0.970	0.974	0.081	0.074	0.088	448.215
FIM-4	0.969	0.972	0.071	0.064	0.078	358.451
FIM-5	0.974	0.977	0.068	0.061	0.075	333.487
FIM-6	0.974	0.977	0.063	0.056	0.070	278.475
FIM-7	0.978	0.981	0.058	0.051	0.065	255.501
FIM-8	0.975	0.978	0.065	0.058	0.072	304.653
FIM-9	0.971	0.974	0.067	0.061	0.075	326.333
FIM-10	0.968	0.971	0.067	0.060	0.074	323.691
FIM1-10	Pooled SRMR: 0.060					
Family support [22]*, 5 items, 0 unique item covariances*						
CM-1	0.990	0.985	0.057	0.047	0.066	138.928
CM-2	0.992	0.987	0.052	0.043	0.062	122.789
CM-3	0.994	0.99	0.046	0.036	0.056	100.896
CM-4	0.993	0.99	0.046	0.037	0.056	102.619
CM-5	0.991	0.987	0.054	0.044	0.063	128.006
CM-6	0.992	0.988	0.048	0.039	0.058	108.791
CM-7	0.990	0.985	0.056	0.047	0.066	138.048
CM-8	0.989	0.983	0.059	0.049	0.068	146.971
CM-9	0.991	0.986	0.052	0.043	0.062	123.619

CM-10	0.992	0.988	0.051	0.042	0.061	119.283
CM1-10	Pooled SRMR: 0.029					
SIM-1	0.985	0.983	0.06	0.052	0.068	212.679
SIM-2	0.99	0.989	0.049	0.041	0.057	153.589
SIM-3	0.989	0.988	0.051	0.043	0.059	162.287
SIM-4	0.989	0.988	0.051	0.043	0.059	161.544
SIM-5	0.989	0.987	0.052	0.044	0.061	169.785
SIM-6	0.99	0.989	0.047	0.039	0.055	144.249
SIM-7	0.987	0.985	0.055	0.047	0.063	185.314
SIM-8	0.987	0.986	0.054	0.046	0.063	180.133
SIM-9	0.988	0.987	0.051	0.043	0.060	165.77
SIM-10	0.991	0.990	0.048	0.040	0.056	148.522
SIM1-10	Pooled SRMR: 0.029					
FIM-1	0.981	0.981	0.063	0.056	0.071	269.238
FIM-2	0.984	0.985	0.056	0.049	0.063	221.745
FIM-3	0.981	0.981	0.063	0.056	0.071	271.247
FIM-4	0.984	0.984	0.057	0.05	0.064	227.918
FIM-5	0.985	0.986	0.055	0.048	0.063	218.267
FIM-6	0.989	0.989	0.046	0.039	0.054	166.872
FIM-7	0.983	0.984	0.057	0.05	0.065	230.402
FIM-8	0.985	0.986	0.054	0.046	0.061	207.083
FIM-9	0.988	0.989	0.048	0.040	0.055	174.514
FIM-10	0.982	0.983	0.061	0.054	0.069	257.422
FIM1-10	Pooled SRMR: 0.041					
Family cohesion [22]*, 7 items, 1 unique item covariance*						
CM-1	0.983	0.977	0.053	0.047	0.059	288.144
CM-2	0.97	0.959	0.066	0.06	0.072	414.681
CM-3	0.984	0.978	0.055	0.048	0.061	303.946
CM-4	0.97	0.959	0.071	0.065	0.077	470.339
CM-5	0.983	0.976	0.053	0.047	0.06	292.398
CM-6	0.98	0.973	0.056	0.05	0.062	314.174
CM-7	0.988	0.983	0.047	0.041	0.054	244.447
CM-8	0.98	0.973	0.056	0.05	0.063	318.451
CM-9	0.981	0.974	0.055	0.049	0.061	308.343

CM-10	0.985	0.979	0.052	0.046	0.058	280.724
CM1-10	Pooled SRMR: 0.043					
SIM-1	0.978	0.976	0.054	0.049	0.06	368.208
SIM-2	0.964	0.96	0.066	0.06	0.071	502.767
SIM-3	0.978	0.975	0.057	0.052	0.063	399.398
SIM-4	0.964	0.96	0.071	0.065	0.076	571.349
SIM-5	0.979	0.976	0.053	0.048	0.059	358.802
SIM-6	0.975	0.973	0.056	0.051	0.062	391.492
SIM-7	0.98	0.978	0.055	0.049	0.06	374.379
SIM-8	0.975	0.972	0.057	0.051	0.063	398.621
SIM-9	0.977	0.974	0.055	0.05	0.061	381.047
SIM-10	0.981	0.979	0.052	0.047	0.058	348.549
SIM1-10	Pooled SRMR: 0.044					
FIM-1	0.969	0.969	0.061	0.056	0.067	499.191
FIM-2	0.964	0.964	0.062	0.057	0.067	506.42
FIM-3	0.962	0.962	0.071	0.066	0.076	631.775
FIM-4	0.959	0.959	0.071	0.066	0.076	639.074
FIM-5	0.973	0.973	0.057	0.052	0.063	443.698
FIM-6	0.967	0.967	0.062	0.057	0.068	511.904
FIM-7	0.963	0.963	0.071	0.065	0.076	627.937
FIM-8	0.965	0.965	0.063	0.058	0.069	525.539
FIM-9	0.973	0.973	0.057	0.052	0.063	443.87
FIM-10	0.966	0.966	0.066	0.061	0.071	563.688
FIM1-10	Pooled SRMR: 0.055					
Positive self-esteem [23], 5 items, 0 unique item covariances						
CM-1	0.994	0.99	0.069	0.06	0.079	194.745
CM-2	0.995	0.993	0.059	0.05	0.068	148.122
CM-3	0.996	0.994	0.055	0.046	0.064	132.42
CM-4	0.996	0.994	0.056	0.047	0.066	137.011
CM-5	0.997	0.995	0.052	0.042	0.061	121.014
CM-6	0.996	0.993	0.057	0.048	0.067	141.289
CM-7	0.997	0.995	0.05	0.04	0.059	113.795
CM-8	0.997	0.995	0.051	0.042	0.061	118.093
CM-9	0.996	0.994	0.055	0.046	0.065	134.281

CM-10	0.996	0.994	0.055	0.046	0.065	133.806
CM1-10	Pooled SRMR: 0.022					
SIM-1	0.993	0.992	0.062	0.055	0.07	234.615
SIM-2	0.994	0.994	0.054	0.047	0.062	189.538
SIM-3	0.995	0.995	0.05	0.043	0.059	168.888
SIM-4	0.995	0.995	0.053	0.045	0.061	182.032
SIM-5	0.996	0.995	0.05	0.042	0.058	167.425
SIM-6	0.996	0.995	0.049	0.041	0.057	159.632
SIM-7	0.996	0.996	0.044	0.036	0.053	140.022
SIM-8	0.996	0.995	0.047	0.039	0.055	152.146
SIM-9	0.996	0.996	0.047	0.039	0.055	150.135
SIM-10	0.995	0.995	0.051	0.043	0.059	171.582
SIM1-10	Pooled SRMR: 0.022					
FIM-1	0.993	0.994	0.054	0.047	0.062	219.431
FIM-2	0.994	0.994	0.053	0.045	0.06	210.452
FIM-3	0.994	0.995	0.053	0.046	0.06	212.1
FIM-4	0.993	0.994	0.058	0.051	0.065	243.325
FIM-5	0.993	0.993	0.06	0.053	0.068	260.99
FIM-6	0.995	0.995	0.048	0.041	0.055	182.094
FIM-7	0.994	0.994	0.054	0.047	0.062	219.7
FIM-8	0.995	0.996	0.045	0.037	0.052	165.949
FIM-9	0.996	0.996	0.044	0.037	0.052	162.66
FIM-10	0.994	0.994	0.054	0.047	0.061	216.742
FIM1-10	Pooled SRMR: 0.029					
Negative self-esteem [23]*, 5 items, 0 unique item covariances*						
CM-1	0.999	0.999	0.025	0.013	0.036	50.578
CM-2	0.999	0.999	0.025	0.013	0.036	50.643
CM-3	0.999	0.999	0.025	0.012	0.036	50.203
CM-4	1	0.999	0.023	0.01	0.035	47.206
CM-5	1	0.999	0.02	0	0.032	42.357
CM-6	1	0.999	0.02	0.003	0.032	43.016
CM-7	1	1	0.015	0	0.028	36.877
CM-8	0.999	0.999	0.024	0.012	0.036	49.542
CM-9	0.999	0.999	0.027	0.015	0.038	54.188

CM-10	1	1	0.018	0	0.03	40.01
CM1-10	Pooled SRMR: 0.023					
SIM-1	0.999	0.999	0.031	0.022	0.04	89.463
SIM-2	0.999	0.999	0.031	0.022	0.04	88.917
SIM-3	0.999	0.999	0.028	0.018	0.037	80.439
SIM-4	0.999	0.999	0.024	0.013	0.033	70.227
SIM-5	0.999	0.999	0.023	0.012	0.033	68.249
SIM-6	0.999	0.999	0.024	0.014	0.033	70.689
SIM-7	0.999	0.999	0.024	0.013	0.033	69.754
SIM-8	0.999	0.999	0.03	0.021	0.039	87.168
SIM-9	0.999	0.999	0.028	0.019	0.037	80.832
SIM-10	0.999	0.999	0.021	0.009	0.031	63.684
SIM1-10	Pooled SRMR: 0.023					
FIM-1	0.989	0.989	0.084	0.078	0.092	463.987
FIM-2	0.988	0.989	0.088	0.081	0.095	503.638
FIM-3	0.985	0.986	0.091	0.084	0.098	527.52
FIM-4	0.986	0.987	0.094	0.087	0.101	559.567
FIM-5	0.986	0.987	0.094	0.088	0.102	567.829
FIM-6	0.986	0.987	0.09	0.084	0.098	525.236
FIM-7	0.989	0.989	0.089	0.082	0.096	506.841
FIM-8	0.988	0.989	0.086	0.079	0.093	474.214
FIM-9	0.984	0.985	0.104	0.097	0.111	678.34
FIM-10	0.986	0.987	0.094	0.087	0.101	557.688
FIM1-10	Pooled SRMR: 0.063					
Brooding old [3,24]*, 4 items, 0 unique item covariances*						
CM-1	0.977	0.956	0.067	0.054	0.08	93.868
CM-2	0.971	0.947	0.078	0.065	0.091	122.255
CM-3	0.977	0.957	0.068	0.055	0.081	96.623
CM-4	0.98	0.962	0.071	0.059	0.084	105.314
CM-5	0.977	0.956	0.076	0.063	0.089	117.05
CM-6	0.973	0.949	0.078	0.066	0.091	123.139
CM-7	0.977	0.957	0.069	0.056	0.082	98.864
CM-8	0.973	0.949	0.071	0.058	0.084	104.333
CM-9	0.969	0.942	0.077	0.065	0.09	121.504

CM-10	0.971	0.946	0.078	0.065	0.091	122.079
CM1-10	Pooled SRMR: 0.054					
SIM-1	0.936	0.929	0.085	0.075	0.095	238.995
SIM-2	0.927	0.918	0.096	0.086	0.106	298.739
SIM-3	0.936	0.928	0.088	0.078	0.098	252.866
SIM-4	0.937	0.93	0.097	0.087	0.107	303.33
SIM-5	0.935	0.927	0.098	0.088	0.107	307.155
SIM-6	0.932	0.924	0.096	0.086	0.106	296.329
SIM-7	0.936	0.928	0.089	0.08	0.099	261.496
SIM-8	0.93	0.922	0.088	0.078	0.098	254.419
SIM-9	0.938	0.93	0.085	0.075	0.095	237.518
SIM-10	0.928	0.919	0.095	0.085	0.105	291.417
SIM1-10	Pooled SRMR: 0.062					
FIM-1	0.942	0.948	0.073	0.064	0.082	225.822
FIM-2	0.934	0.94	0.082	0.074	0.091	279.996
FIM-3	0.946	0.952	0.072	0.063	0.081	221.084
FIM-4	0.941	0.947	0.084	0.076	0.093	292.813
FIM-5	0.939	0.945	0.085	0.076	0.094	296.591
FIM-6	0.937	0.943	0.082	0.074	0.091	280.363
FIM-7	0.941	0.947	0.077	0.068	0.086	247.179
FIM-8	0.939	0.945	0.074	0.065	0.083	231.274
FIM-9	0.942	0.948	0.073	0.064	0.082	227.791
FIM-10	0.937	0.943	0.08	0.071	0.089	265.313
FIM1-10	Pooled SRMR: 0.064					
Brooding new [3,24]*, 5 items, 0 unique item covariances*						
CM-1	0.992	0.988	0.048	0.038	0.057	106.843
CM-2	0.992	0.987	0.048	0.038	0.058	107.93
CM-3	0.991	0.986	0.05	0.04	0.059	113.783
CM-4	0.992	0.988	0.048	0.039	0.058	108.199
CM-5	0.991	0.986	0.05	0.041	0.06	115.891
CM-6	0.992	0.988	0.047	0.037	0.057	104.157
CM-7	0.993	0.99	0.043	0.033	0.053	92.914
CM-8	0.993	0.988	0.045	0.035	0.055	98.435
CM-9	0.992	0.988	0.046	0.036	0.056	101.432

CM-10	0.993	0.989	0.044	0.034	0.054	94.903
CM1-10	Pooled SRMR: 0.032					
SIM-1	0.99	0.989	0.045	0.037	0.053	142.215
SIM-2	0.99	0.99	0.043	0.035	0.051	133.586
SIM-3	0.988	0.987	0.047	0.039	0.056	153.827
SIM-4	0.99	0.99	0.044	0.036	0.052	138.354
SIM-5	0.988	0.987	0.048	0.04	0.056	156.958
SIM-6	0.991	0.99	0.043	0.035	0.051	133.694
SIM-7	0.99	0.99	0.043	0.035	0.052	135.477
SIM-8	0.991	0.99	0.042	0.034	0.05	129.786
SIM-9	0.99	0.989	0.043	0.035	0.052	135.846
SIM-10	0.99	0.99	0.043	0.035	0.051	132.763
SIM1-10	Pooled SRMR: 0.031					
FIM-1	0.986	0.987	0.049	0.041	0.056	186.199
FIM-2	0.981	0.983	0.055	0.048	0.063	227.768
FIM-3	0.981	0.983	0.055	0.048	0.062	224.08
FIM-4	0.984	0.985	0.052	0.045	0.059	205.898
FIM-5	0.981	0.983	0.056	0.049	0.064	233.487
FIM-6	0.983	0.984	0.054	0.047	0.061	217.252
FIM-7	0.983	0.985	0.053	0.046	0.06	211.592
FIM-8	0.982	0.983	0.054	0.047	0.061	218.171
FIM-9	0.983	0.984	0.053	0.046	0.06	210.824
FIM-10	0.983	0.984	0.053	0.045	0.06	209.935
FIM1-10	Pooled SRMR: 0.042					
Reflection [3,24]*, 5 items, 1 unique item covariance*						
CM-1	0.992	0.986	0.046	0.037	0.057	95.986
CM-2	0.991	0.985	0.048	0.038	0.058	101.052
CM-3	0.99	0.983	0.05	0.04	0.06	105.905
CM-4	0.992	0.987	0.046	0.036	0.057	95.777
CM-5	0.992	0.986	0.047	0.037	0.057	98.052
CM-6	0.992	0.987	0.045	0.036	0.056	93.022
CM-7	0.994	0.989	0.041	0.031	0.052	81.375
CM-8	0.992	0.986	0.046	0.036	0.056	94.02
CM-9	0.992	0.986	0.048	0.038	0.058	99.497

CM-10	0.991	0.986	0.047	0.038	0.058	98.857
CM1-10	Pooled SRMR: 0.045					
SIM-1	0.986	0.984	0.05	0.042	0.058	156.491
SIM-2	0.985	0.984	0.05	0.042	0.059	159.87
SIM-3	0.985	0.983	0.051	0.043	0.059	161.732
SIM-4	0.985	0.983	0.052	0.044	0.061	170.309
SIM-5	0.986	0.984	0.05	0.042	0.059	160.03
SIM-6	0.986	0.984	0.05	0.042	0.058	157.737
SIM-7	0.988	0.986	0.046	0.038	0.055	141.928
SIM-8	0.986	0.984	0.049	0.041	0.057	153.663
SIM-9	0.986	0.984	0.051	0.043	0.059	163.984
SIM-10	0.986	0.984	0.05	0.042	0.058	158.201
SIM1-10	Pooled SRMR: 0.045					
FIM-1	0.966	0.968	0.071	0.064	0.079	330.033
FIM-2	0.968	0.969	0.069	0.062	0.076	313.059
FIM-3	0.963	0.965	0.073	0.065	0.08	340.539
FIM-4	0.967	0.968	0.072	0.065	0.079	336.733
FIM-5	0.968	0.969	0.07	0.063	0.077	319.366
FIM-6	0.97	0.971	0.067	0.06	0.074	298.213
FIM-7	0.969	0.97	0.069	0.062	0.076	311.317
FIM-8	0.967	0.968	0.069	0.062	0.077	315.391
FIM-9	0.967	0.969	0.071	0.064	0.078	327.705
FIM-10	0.965	0.966	0.072	0.065	0.079	336.78
FIM1-10	Pooled SRMR: 0.063					
Distress tolerance [25]*, 5 items, 1 unique item covariance*						
CM-1	0.968	0.947	0.101	0.092	0.111	356.552
CM-2	0.969	0.948	0.105	0.096	0.114	379.225
CM-3	0.967	0.946	0.105	0.095	0.114	377.534
CM-4	0.968	0.947	0.103	0.094	0.113	369.052
CM-5	0.97	0.95	0.102	0.092	0.111	358.546
CM-6	0.971	0.951	0.101	0.092	0.111	354.518
CM-7	0.97	0.95	0.1	0.091	0.109	346.656
CM-8	0.972	0.954	0.097	0.088	0.107	329.16
CM-9	0.972	0.953	0.102	0.093	0.111	360.153

CM-10	0.97	0.95	0.101	0.092	0.11	353.137
CM1-10	Pooled SRMR: 0.059					
SIM-1	0.968	0.968	0.079	0.072	0.086	376.783
SIM-2	0.97	0.97	0.079	0.072	0.086	378.301
SIM-3	0.966	0.966	0.083	0.076	0.09	411.1
SIM-4	0.969	0.969	0.079	0.072	0.087	379.576
SIM-5	0.97	0.97	0.079	0.072	0.086	377.378
SIM-6	0.97	0.97	0.079	0.072	0.086	377.037
SIM-7	0.97	0.97	0.077	0.07	0.085	364.221
SIM-8	0.973	0.973	0.074	0.067	0.082	337.75
SIM-9	0.971	0.971	0.079	0.072	0.087	382.255
SIM-10	0.969	0.969	0.08	0.072	0.087	383.474
SIM1-10	Pooled SRMR: 0.060					
FIM-1	0.970	0.974	0.071	0.064	0.078	361.965
FIM-2	0.975	0.978	0.068	0.061	0.075	338.295
FIM-3	0.969	0.973	0.073	0.066	0.08	381.179
FIM-4	0.973	0.977	0.068	0.061	0.075	338.4
FIM-5	0.973	0.977	0.07	0.063	0.077	351.802
FIM-6	0.974	0.977	0.069	0.062	0.076	344.05
FIM-7	0.971	0.975	0.07	0.064	0.077	358.149
FIM-8	0.978	0.981	0.063	0.056	0.07	295.438
FIM-9	0.974	0.978	0.07	0.063	0.077	355.542
FIM-10	0.975	0.978	0.067	0.06	0.074	328.527
FIM1-10	Pooled SRMR: 0.064					
Aggression [26]*, 4 items, 0 unique item covariances*						
CM-1	0.999	0.997	0.036	0.022	0.051	38.412
CM-2	0.998	0.995	0.052	0.039	0.065	62.624
CM-3	0.999	0.998	0.035	0.021	0.05	36.937
CM-4	0.997	0.994	0.057	0.044	0.07	72.567
CM-5	0.996	0.992	0.051	0.038	0.064	61.034
CM-6	0.999	0.998	0.032	0.017	0.047	33.314
CM-7	0.998	0.997	0.055	0.042	0.069	69.342
CM-8	0.998	0.996	0.046	0.033	0.06	53.348
CM-9	0.998	0.996	0.045	0.031	0.058	50.375

CM-10	0.999	0.997	0.04	0.026	0.054	43.08
CM1-10	Pooled SRMR: 0.052					
SIM-1	0.993	0.992	0.063	0.053	0.073	136.864
SIM-2	0.993	0.991	0.071	0.061	0.081	167.668
SIM-3	0.994	0.993	0.058	0.048	0.068	119.667
SIM-4	0.992	0.991	0.071	0.061	0.081	167.166
SIM-5	0.989	0.988	0.065	0.055	0.075	144.604
SIM-6	0.994	0.993	0.059	0.049	0.069	122.171
SIM-7	0.995	0.994	0.074	0.064	0.084	179.659
SIM-8	0.994	0.993	0.063	0.053	0.073	136.06
SIM-9	0.992	0.991	0.068	0.058	0.079	157.416
SIM-10	0.994	0.993	0.062	0.052	0.073	134.713
SIM1-10	Pooled SRMR: 0.076					
FIM-1	0.993	0.993	0.059	0.05	0.069	155.276
FIM-2	0.993	0.994	0.061	0.052	0.07	161.385
FIM-3	0.993	0.993	0.059	0.05	0.068	154.017
FIM-4	0.993	0.993	0.061	0.052	0.07	162.482
FIM-5	0.99	0.99	0.058	0.049	0.067	148.267
FIM-6	0.992	0.992	0.06	0.051	0.07	159.709
FIM-7	0.994	0.994	0.073	0.064	0.083	221.604
FIM-8	0.994	0.994	0.058	0.049	0.067	148.019
FIM-9	0.992	0.993	0.062	0.053	0.071	166.672
FIM-10	0.993	0.993	0.063	0.054	0.073	172.324
FIM1-10	Pooled SRMR: 0.119					
General distress [27,28]*, 41 items, 2 unique item covariances*						
CM-1*	0.989	0.988	0.026	0.025	0.027	5752.268
CM-2*	0.989	0.988	0.026	0.025	0.027	5784.608
CM-3*	0.987	0.987	0.026	0.025	0.027	5707.489
CM-4*	0.989	0.989	0.026	0.025	0.027	5728.317
CM-5*	0.989	0.988	0.026	0.025	0.027	5795.843
CM-6*	0.987	0.987	0.027	0.026	0.028	5900.122
CM-7*	0.990	0.989	0.026	0.025	0.027	5787.842
CM-8*	0.989	0.989	0.026	0.025	0.027	5723.574
CM-9*	0.988	0.987	0.027	0.026	0.028	5892.805

CM-10*	0.987	0.987	0.027	0.026	0.028	5981.058
CM1-10	Pooled SRMR: 0.044					
SIM-1	0.987	0.987	0.027	0.026	0.028	6211.303
SIM-2	0.987	0.987	0.027	0.026	0.028	6249.019
SIM-3	0.986	0.986	0.027	0.026	0.028	6137.497
SIM-4	0.988	0.988	0.027	0.026	0.028	6173.344
SIM-5	0.987	0.987	0.027	0.026	0.028	6217.918
SIM-6	0.986	0.986	0.028	0.027	0.029	6323.823
SIM-7	0.988	0.988	0.027	0.026	0.028	6273.396
SIM-8	0.988	0.988	0.027	0.026	0.028	6148.299
SIM-9	0.986	0.986	0.028	0.027	0.029	6341.883
SIM-10	0.986	0.986	0.028	0.027	0.029	6432.838
SIM1-10	Pooled SRMR: 0.044					
FIM-1	0.953	0.953	0.052	0.051	0.053	14055.219
FIM-2	0.953	0.954	0.052	0.051	0.053	14248.743
FIM-3	0.949	0.949	0.051	0.050	0.052	13603.930
FIM-4	0.953	0.953	0.053	0.052	0.054	14707.235
FIM-5	0.952	0.953	0.053	0.052	0.053	14350.666
FIM-6	0.952	0.953	0.050	0.050	0.051	13508.845
FIM-7	0.953	0.953	0.055	0.054	0.056	15307.465
FIM-8	0.955	0.955	0.052	0.051	0.053	14073.361
FIM-9	0.951	0.952	0.052	0.051	0.053	14046.556
FIM-10	0.951	0.951	0.052	0.051	0.053	14007.992
FIM1-10	Pooled SRMR: 0.107					

Note. WLSMV = weighted least squares estimator with mean- and variance corrected test statistics and robust standard errors. CFI = Comparative fit index, TLI = Tucker-Lewis index, RMSEA = Root mean square error of approximation, CI = Confidence interval, CM = configural model, SIM = strong invariance model, FIM = full invariance model. *For the configural model of the general distress factor, we had to enforce the loadings to be positive to ensure that they would not switch negative. We had to do this, as for some of the imputation data sets, the loadings switched negative and when pooling over the coefficients the positive and negative loadings would have averaged each other out.

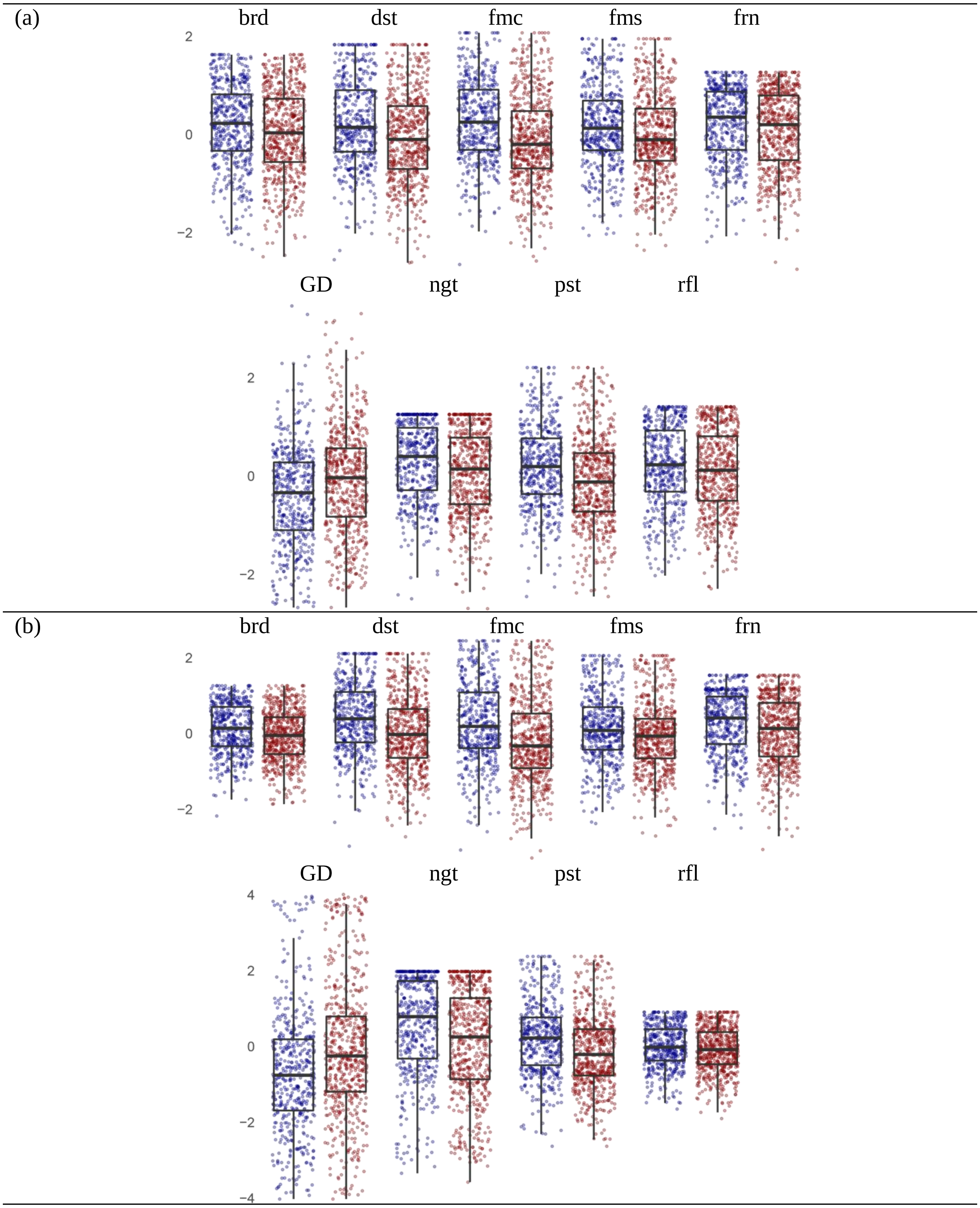
(a)
brd
dst
fmc
fms
frn
2
0
−2
GD
ngt
pst
rfl
2
0
−2
(b)
brd
dst
fmc
fms
frn
2
0
−2
GD
ngt
pst
rfl
4
2
0
−2
−4

(c)

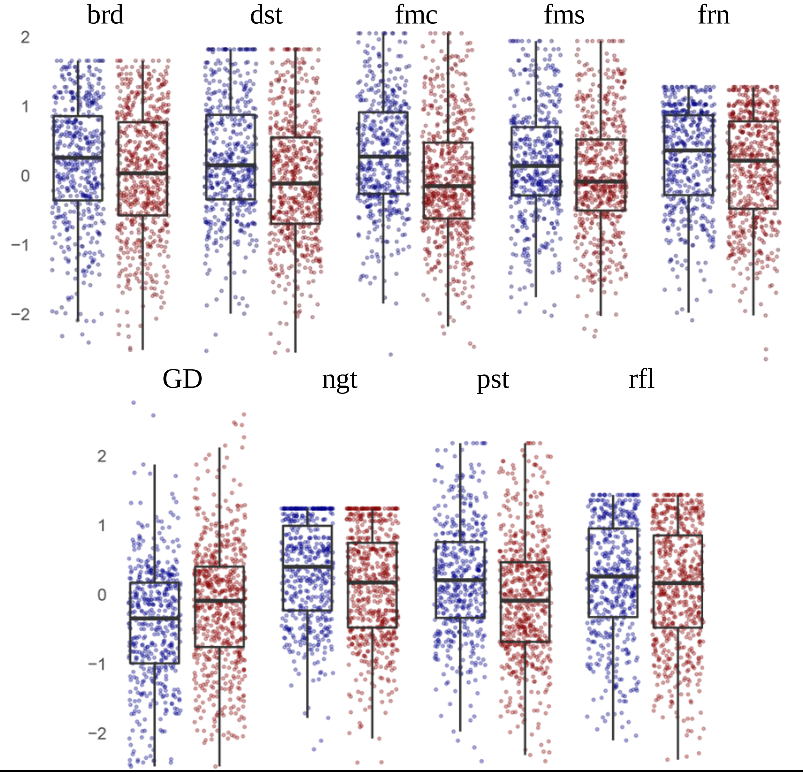

(d)

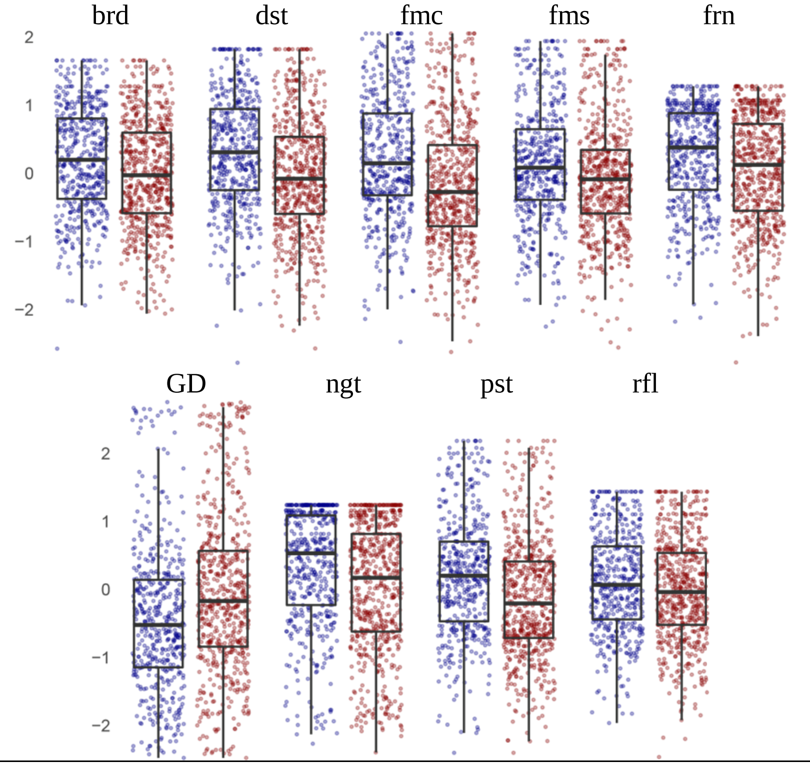

Figure 5. Box-and-whisker plots with individual data points for the RFs (except expressive suppression and aggression) and the general distress variable, separately for CA+ (n = 631) and CA- (n = 499). Panel (a) depicts the distributions for the strongly invariant scores for age 14 and panel (b) for age 17. Panel (c) depicts the distributions for the fully invariant scores for age 14 and panel (d) for age 17. CA- group = blue data points, CA+ group = red data points. Center line = median (50% quantile); lower box

limit =25% quantile; upper box limit = 75% quantile; lower whisker = smallest observation greater than or equal to the lower box limit - 1.5 x Inter Quartile Range (IQR); upper whisker = largest observation less than or equal to upper box limit + 1.5 x IQR; outliers = data points beyond the end of the whiskers. **Legend:** Brd = brooding, dst = distress tolerance, fmc = family cohesion, fms = family support, frn = friend support, ngt = negative self-esteem, GD = general distress, pst = positive self-esteem, rfl = reflective rumination.
