# Supplementary material for: Unravelling the complex nature of resilience factors and their changes between early and later adolescence
Source: BMC Med. 2019 Nov 14;17:203. doi: 10.1186/s12916-019-1430-6 (PMC6854636; doi:10.1186/s12916-019-1430-6)
Supplement: Supplementary file 6 — Additional file 6. Mean change analyses with (a) fully invariant factor scores and (b) sum scores. [file 12916_2019_1430_MOESM6_ESM.pdf]

Additional file VI

We compared the RF and general distress mean levels between age 14 and age 17, separately in the CA+ and CA- groups, based on strongly invariant factor scores, fully invariant factor scores, and sum scores. Results for the strongly invariant factor scores can be found in the main manuscript (see also Figure 1 in the main manuscript). We believe that the strongly invariant model is the most adequate model for latent mean comparisons, as it is the least constrained one of the sufficiently constrained models.

Fully invariant factor scores. For the fully invariant factor scores, the results looked somewhat different. This time, change was particularly notable in the CA+ group. In the CA- group only reflection and distress tolerance changed. While reflection deceased over time, distress tolerance increased over time, as for the strongly invariant scores. In the CA+ group instead of two this time six RFs changed between age 14 and 17. All inter-personal RFs (i.e. friendship support, family support and family cohesion) and three intra-personal RFs (negative self-esteem, positive self-esteem, and reflection) decreased between age 14 and 17 (see Figure 6a). Of those six decreasing RFs in the CA+ group, only two reached a p-values below 0.025 (namely family cohesion and reflection). Importantly, none of the RFs changed significantly different in the CA+ and the CA- group (as tested with interaction effects), which indicates that the detected changes in the CA+ group, which were non-significant in the CA- group, were so minor that they did not differ significantly between the two groups. Of note, the binarized aggression and expressive suppression RFs are the same variables as reported in the main manuscript.

Sum scores. For the sum scores, the results looked again somewhat different from the strongly invariant factor scores. However, the change patterns were the same as for the fully invariant factor scores (see Figure 6b). Importantly, once more none of the RFs changed significantly different in the CA+ and the CA- group (as tested with interaction effects). Hence, the same conclusion seems to hold as for fully invariant factor scores, namely that the detected changes in the CA+ group, which were non-significant in the CA- group, were so minor that they did not differ significantly between the two groups. Hence, overall we conclude that when investigating fully invariant factor scores and sum scores there seemed to be more variability in the CA+ group between age 14 and 17, yet, those changes were so minor that they did not differ between the two groups.

A. Fully invariant factor scores

CA+	CA-

No RF change

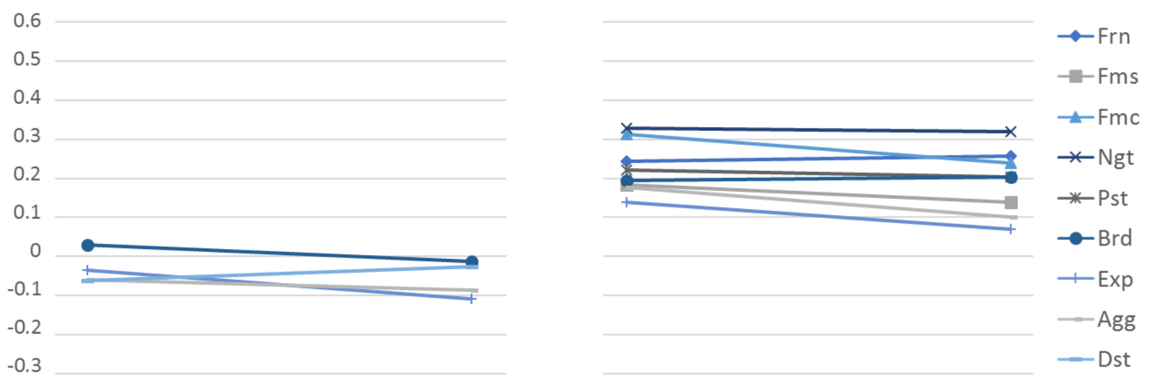

Increasing RFs

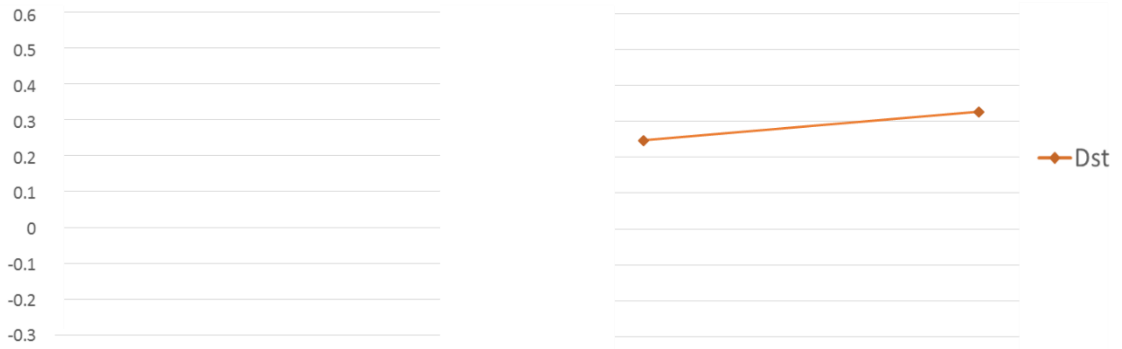

Decreasing RFs

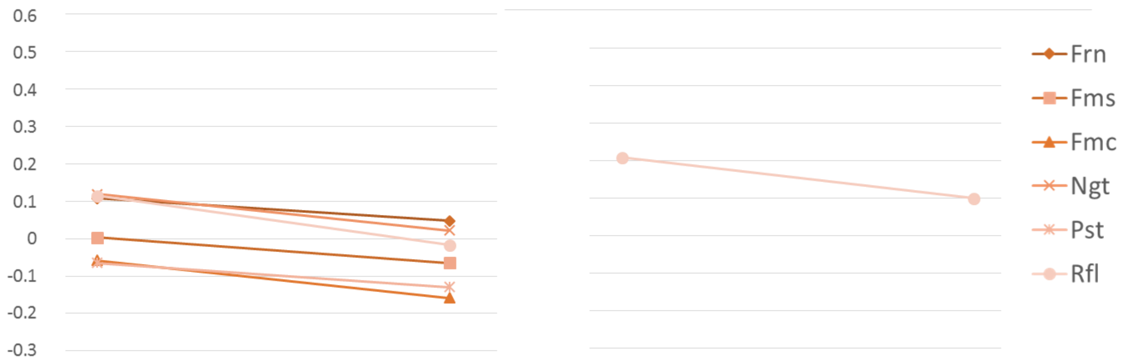

B. Sum scores

CA+	CA-

No RF change

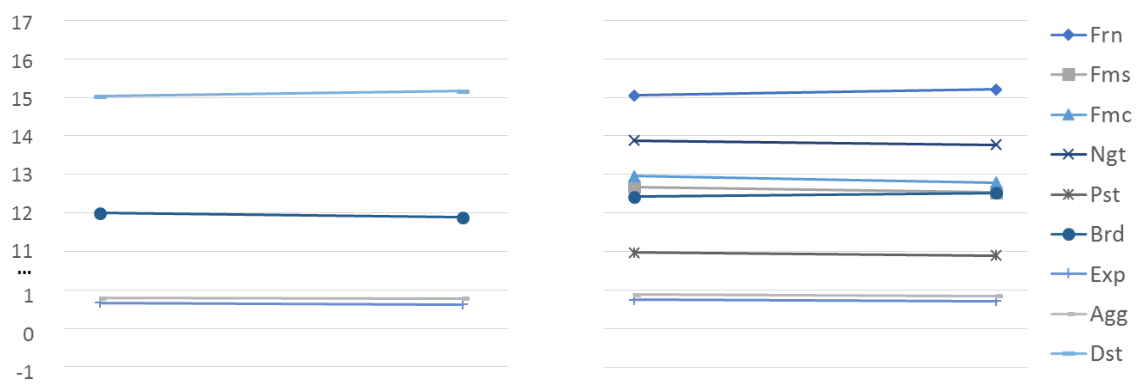

Increasing RFs

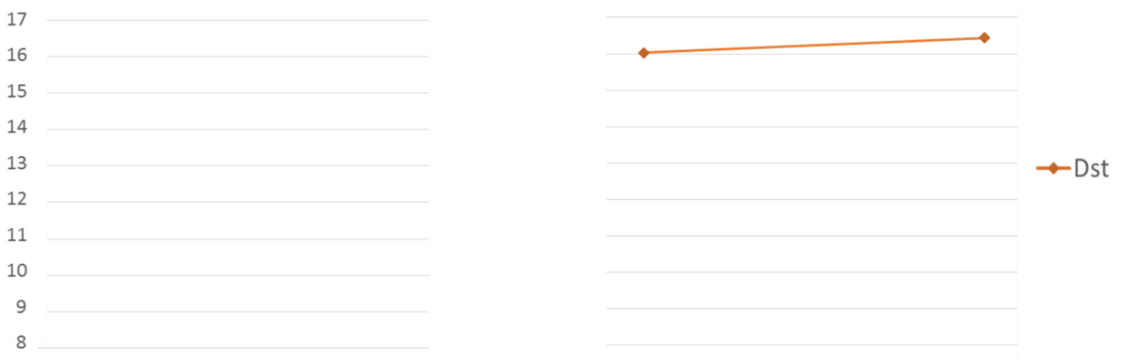

Decreasing RFs

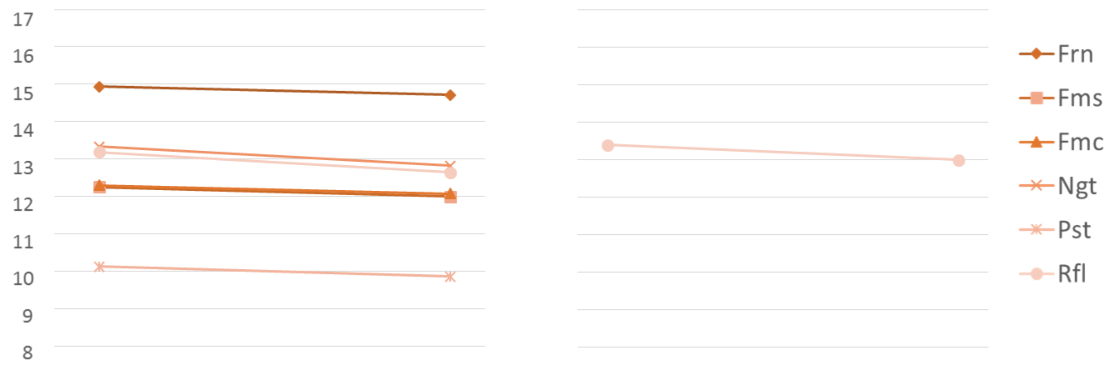

Figure 6. RF and general distress mean level comparisons: age 14 versus age 17. CA = childhood adversity. Panel A scores are derived from fully invariant confirmatory factor analyses; panel B scores are sum scores. All RFs are scored in such a way that high values are protective (e.g. high levels of high friendship support or high levels of low negative self-esteem) and low values are harmful (e.g. low levels of high friendship support or low levels of low negative self-esteem). **Legend:** Frn = friend support,

fms = family support, fmc = family cohesion, ngt = negative self-esteem, pst = positive self-esteem, rfl = reflection, brd = brooding, dst = distress tolerance, agg = aggression, exp = expressive suppression.
