# Supplementary material for: Unravelling the complex nature of resilience factors and their changes between early and later adolescence
Source: BMC Med. 2019 Nov 14;17:203. doi: 10.1186/s12916-019-1430-6 (PMC6854636; doi:10.1186/s12916-019-1430-6)
Supplement: Supplementary file 7 — Additional file 7. RF network results without the general distress variable as well as RF network results with the general distress variable. [file 12916_2019_1430_MOESM7_ESM.pdf]

**Additional file VII**

**RF networks without the general distress variable.** At age 14, the network invariance test was not significant for the RF networks without the general distress variable (M = .15, p = .30; see Figure 7), but the global network expected influence (EI) differed between the CA+ and the CA- RF networks ($EI_{CA+}$ = 3.21, $EI_{CA-}$ = 3.53, EI = 0.31, p = .03). More specifically, the global network expected influence was higher in the CA- than in the CA+ network. Those findings were only partially similar in the RF networks for age 17, as neither of the two tests revealed significant differences between the CA+ and the CA- group (M = .12, p = .56; $EI_{CA+}$ = 3.54, $EI_{CA-}$ = 3.35, EI = 0.19, p = .16). At age 14 six interrelations differed between the CA+ and the CA- networks: namely friendship support and brooding (CA+: less positive; CA-:more positive), friendship support and expressive suppression (CA+: negative; CA-: positive), positive self-esteem and brooding (CA+: null; CA-: positive), positive self-esteem and expressive suppression (CA+: null; CA-: negative), brooding and aggression (CA+: null; CA-: positive), as well as reflection and distress tolerance (CA+: null; CA-: positive). At age 17, the interrelation between friendship support and positive self-esteem (CA+: positive, CA-: null) differed between the CA+ and the CA- network.

Interestingly, when we compared the RF networks for age 14 and age 17, we did find a global network structure differences for adolescents with (M = .22, p = .003; $EI_{14}$ = 3.21, $EI_{17}$ = 3.54, EI = 0.33, p = .001), but not for adolescents without a history of adversity (M = .17, p = .24; $EI_{14}$ = 3.53, $EI_{17}$ = 3.35, EI = 0.18, p = .22). In the CA+ network five RF interrelations changed from age 14 to age 17, namely the interrelation between friendship support and expressive suppression (from negative to positive), positive and negative self-esteem (from more to less positive), positive self-esteem and aggression (from null to positive), negative self-esteem and brooding (from more to less positive), and the interrelation between negative self-esteem and aggression (from less to more positive). In the CA- network, three RF interrelations changed from age 14 to age 17, namely the interrelation between friendship support and negative self-esteem (from positive to more positive), negative self-esteem and aggression (from positive to more positive), and the interrelation between brooding and reflection (from more to less positive).

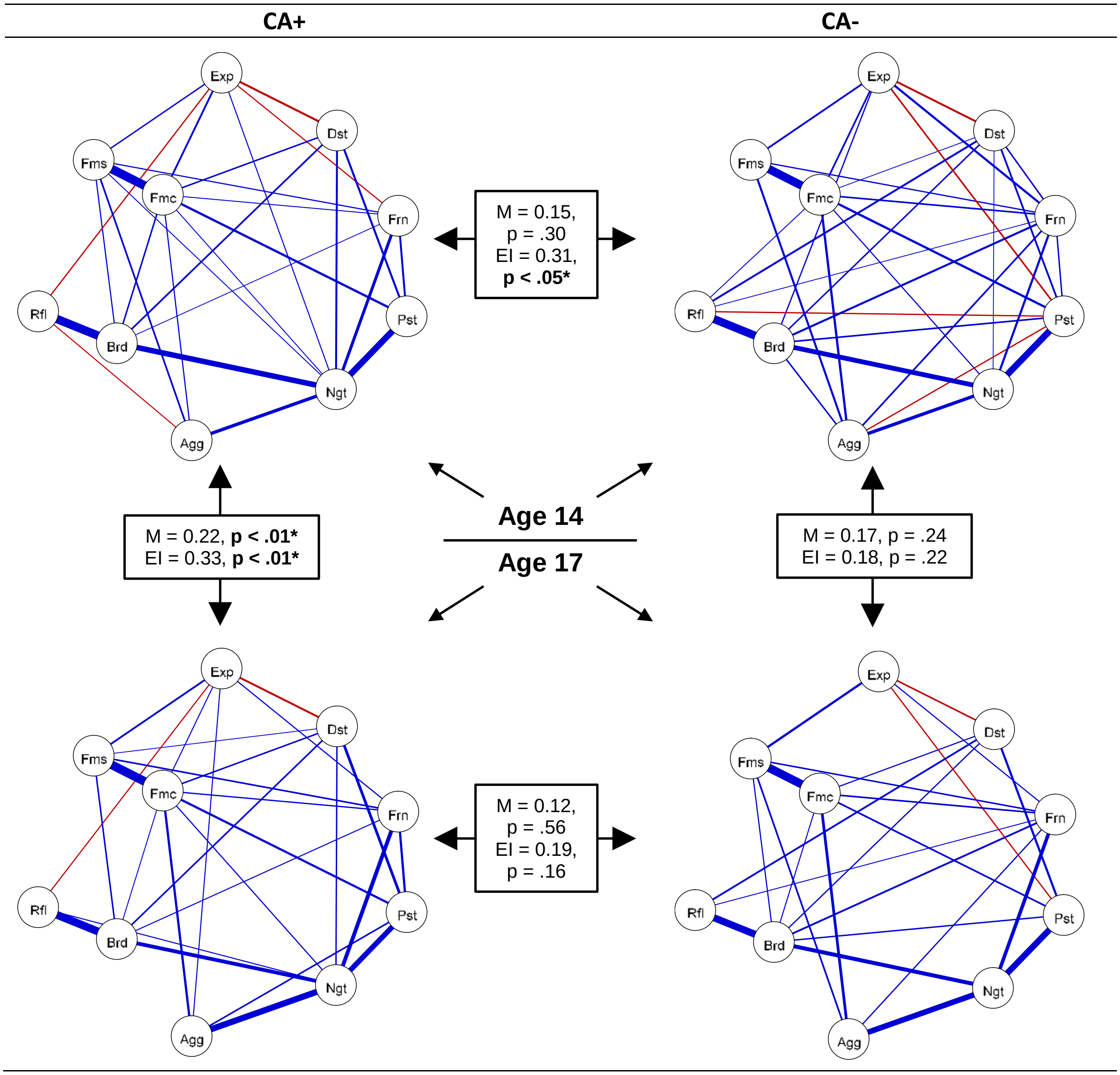


*Figure 7*. CA+ (*n* = 631) and CA- (*n* = 499) resilience factor networks for age 14 (upper panel) and age 17 (lower panel) without the general distress variable. Width of the lines = association strength. Positive interrelations = blue, negative interrelations = red. **Legend:** Frn = friend support, fms = family support, fmc = family cohesion, ngt = negative self-esteem, pst = positive self-esteem, rfl = reflection, brd = brooding, dst = distress tolerance, agg = aggression, exp = expressive suppression. The boxes depict the maximal interrelation difference between the respective two networks (M), the difference in global network expected influence (EI) between the respective two networks (EI), and the corresponding p-values (5000 comparison samples). The above networks with faded interrelations can be found in Supplement XIII.

**RF networks with the general distress variable**. At age 14, the network invariance test was not significant for the RF networks with the general distress variable (M = .14, p = .47; see Figure 8). However, the network expected influence was significantly higher in the CA- than in the CA+ network ($EI_{CA+}$ = 0.87, $EI_{CA-}$ = 1.43, EI = 0.56, p = .04). Those findings were only partially similar in the networks for age 17, as neither of the two tests revealed significant differences between the CA+ and the CA- group at age 17 (M = .16, p = .34; $EI_{CA+}$ = 0.97, $EI_{CA-}$ = 1.16, EI = 0.19, p = .54). At age 14, four interrelation differed between the CA+ and the CA- network, namely the interrelations between friendship support and brooding (CA+: null, CA-: positive), friendship support and expressive suppression (CA+: negative, CA-: positive), positive self-esteem and expressive suppression (CA+: null, CA-: negative), as well as between reflection and distress tolerance (CA+: null, CA-: positive). At age 17 two interrelations differed between the CA+ and the CA- network, namely friendship support and positive self-esteem (CA+: positive, CA-: null), as well as aggression and general distress (CA+: more negative, CA-: less negative).

When we compared those networks for age 14 and age 17, the network invariance test was still significant (M = .20, p = .01), but the global network expected influence did no longer differ ($EI_{14}$ = 0.87, $EI_{17}$ = 0.97, EI = 0.11, p = .71) in the CA+ group. In the CA- group, neither of the two tests was significant (M = .12, p = .79; $EI_{14}$ = 1.43, $EI_{17}$ = 1.16, EI = 0.26, p = .36). In the CA+ network five individual RF interrelations changed from age 14 to age 17, namely the interrelations between friendship support and expressive suppression (from negative to positive), family cohesion and general distress (from more to less negative), negative self-esteem and brooding (from more to less positive), brooding and general distress (from more to less negative), as well as between aggression and general distress (from less to more negative). In contrast in the CA- network, only two RF interrelation changed from age 14 to age 17, namely the interrelation between negative self-esteem and general distress (from less to more negative), as well as the interrelation between brooding and general distress (from more to less negative).

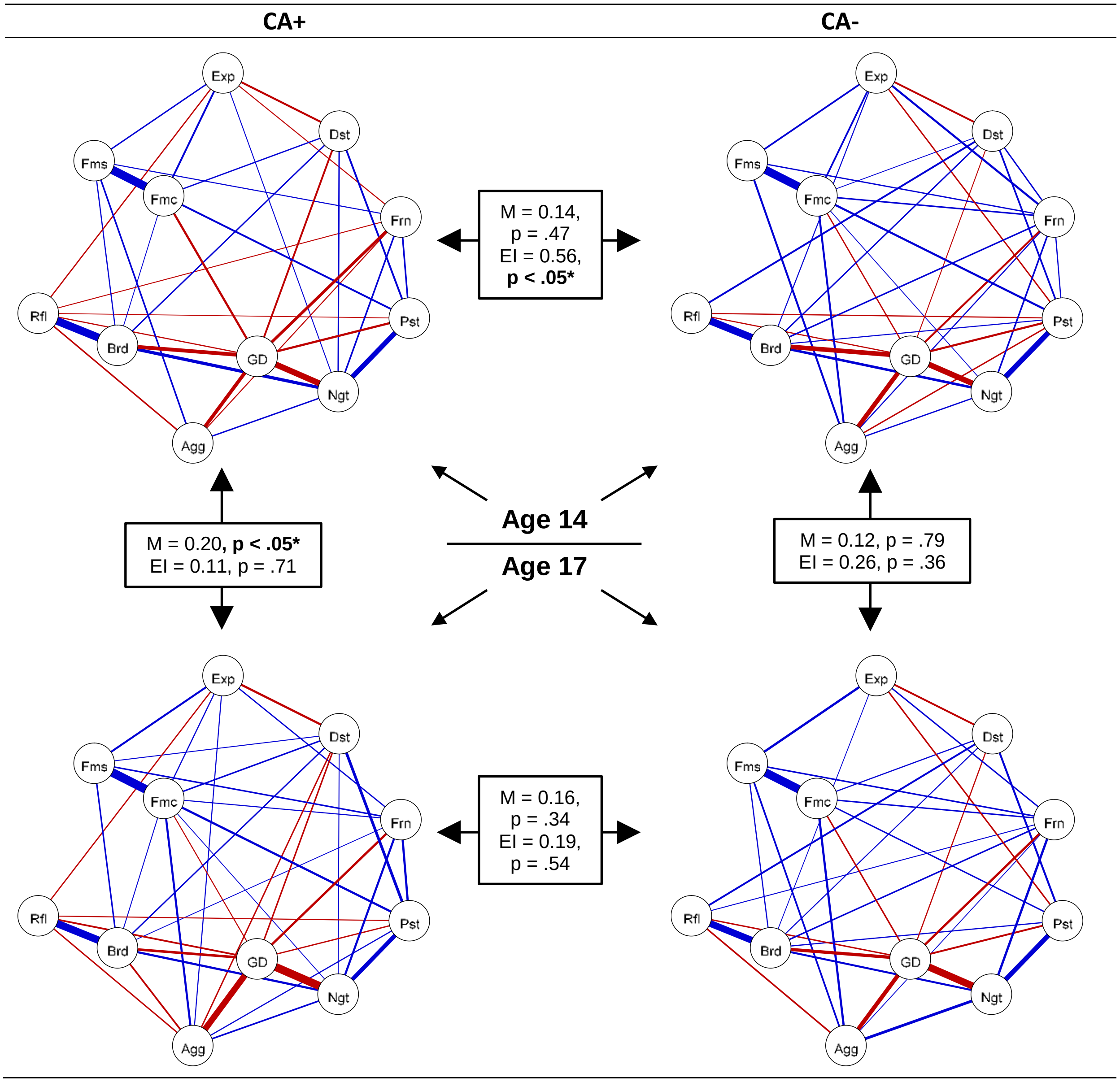


*Figure 8.* CA+ (*n* = 631) and CA- (*n* = 499) resilience factor networks for age 14 (upper panel) and age 17 (lower panel) with the general distress variable. Width of the lines = association strength. Positive interrelations = blue, negative interrelations = red. **Legend:** Frn = friend support, fms = family support, fmc = family cohesion, ngt = negative self-esteem, pst = positive self-esteem, rfl = reflection, brd = brooding, dst = distress tolerance, agg = aggression, exp = expressive suppression, GD = general distress. The boxes depict the maximal interrelation difference between the respective two networks (M), the difference in global network expected influence (EI) between the respective two networks (EI), and the corresponding p-values (5000 comparison samples). The above networks with faded interrelations can be found in Supplement XIII.
