# Supplementary material for: Unravelling the complex nature of resilience factors and their changes between early and later adolescence
Source: BMC Med. 2019 Nov 14;17:203. doi: 10.1186/s12916-019-1430-6 (PMC6854636; doi:10.1186/s12916-019-1430-6)
Supplement: Supplementary file 8 — Additional file 8. Correlations and regularized partial correlations between the RFs and the general distress factor. [file 12916_2019_1430_MOESM8_ESM.pdf]

Additional file VIII

As can be seen in Table 6, all RFs were negatively correlated with general distress, except for expressive suppression for CA+ adolescents at age 14 and CA- adolescents at age 17, as it then was positively correlated with general distress. Regularized partial correlations of the RFs, which we used for the network models, were also mostly negative for the relationships between the RFs and general distress. Yet, expressive suppression and family support were in both groups no longer related with general distress, neither at age 14 nor at 17. Thus, the overall results pattern was still similar, showing that even after the correction for all other RFs, most RFs were clearly negatively associated with general distress. As can be seen in Figure 9, negative self-esteem, positive self-esteem, brooding and aggression seemed to be most strongly correlated with general distress. However, in terms of partial correlations, positive self-esteem no longer appeared to be among those factors with the highest interrelations with general distress.

Table 6

Correlations and Regularized Partial Correlations between the RFs and the General Distress Variable

**CA**	**frn**	**fms**	**fmc**	**pst**	**ngt**	**brd**	**rfl**	**dst**	**agg**	**exp**
Correlations										
yes: age 14	-0.43	-0.35	-0.44	-0.59	-0.78	-0.68	-0.49	-0.36	-0.44	0.02
yes: age 17	-0.53	-0.38	-0.46	-0.61	-0.87	-0.55	-0.41	-0.30	-0.74	-0.05
no: age 14	-0.41	-0.31	-0.38	-0.54	-0.74	-0.68	-0.48	-0.23	-0.54	-0.05
no: age 17	-0.49	-0.35	-0.43	-0.57	-0.83	-0.60	-0.39	-0.25	-0.64	0.02
Regularized Partical Correlations										
yes: age 14	-0.17	0.00	-0.12	-0.11	-0.42	-0.25	-0.03	-0.08	-0.22	0.00
yes: age 17	-0.13	0.00	-0.03	-0.04	-0.58	-0.14	-0.08	-0.06	-0.42	0.00
no: age 14	-0.10	0.00	-0.04	-0.11	-0.39	-0.30	-0.03	-0.02	-0.29	0.00
no: age 17	-0.12	0.00	-0.06	-0.09	-0.51	-0.20	-0.05	-0.04	-0.26	0.00

Note. CA = Childhood adversity (yes: *n* = 631, no: *n* = 499). Frn = friend support, fms = family support, fmc = family cohesion, pst = positive self-esteem, ngt = negative self-esteem, brd = brooding, rfl = reflection, dst = distress tolerance, agg = aggression, exp = expressive suppression.

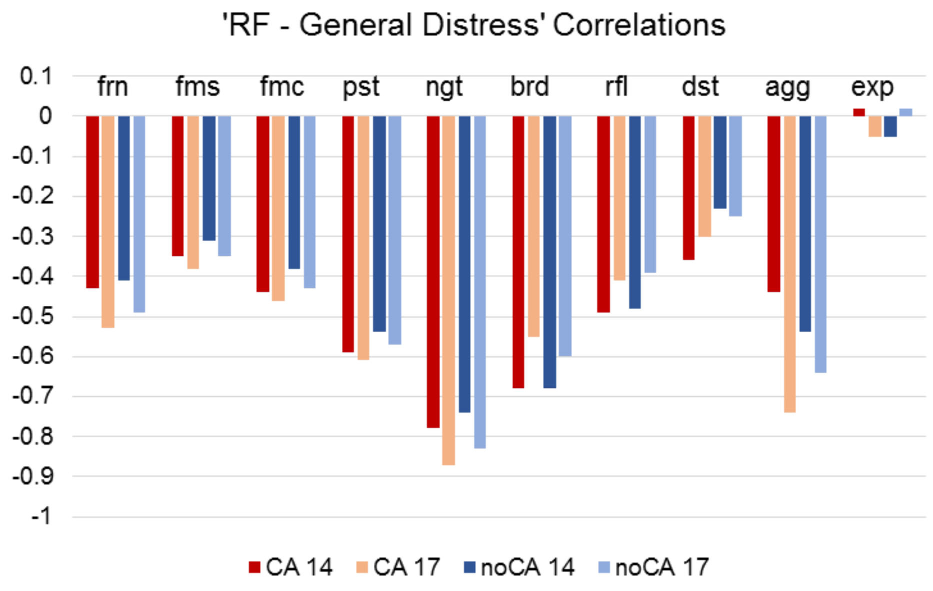

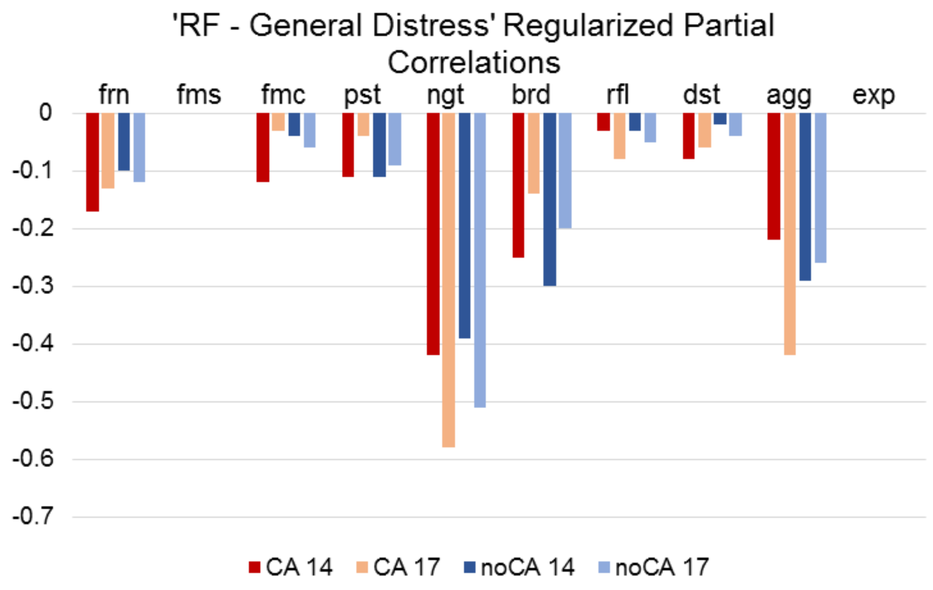

Figure 9. Visualizing the correlations and regularized partial correlations between RFs and general distress, for CA+ (n = 631) and CA- (n = 499) adolescents at age 14 and age 17.
