# Supplementary material for: Unravelling the complex nature of resilience factors and their changes between early and later adolescence
Source: BMC Med. 2019 Nov 14;17:203. doi: 10.1186/s12916-019-1430-6 (PMC6854636; doi:10.1186/s12916-019-1430-6)
Supplement: Supplementary file 9 — Additional file 9. The stability of the expected influence (EI) coefficients and the accuracy of the ‘RF-RF’ and ‘RF-general distress’ interrelations. [file 12916_2019_1430_MOESM9_ESM.pdf]

Additional file IX

To test the stability of the *expected influence (EI)* coefficients we applied a subset bootstrap (2000 bootstraps) to identify the maximum sample percentage that can be dropped to reveal (with a 95% chance) a relationship of ≥0.7 between the subset and the original EI coefficients. The analyses showed that at both age 14 and 17 EI coefficients were sufficiently stable, as more than 50 percent of the sample could be dropped (see Table 7).

To test the accuracy of the network models we bootstrapped the 'RF-RF' and 'RF-general distress' interrelations (2000 bootstraps) and investigated the bootstrap confidence intervals (CIs). Overall, the CIs had an acceptable width, which indicates that the estimated models have an appropriate interrelation accuracy. At age 14, family cohesion and family support were most strongly interrelated, followed next by the brooding and reflective rumination interrelation, and then by the positive and negative self-esteem interrelation, for both the CA+ and the CA- group (see Figure 10 and Figure 11). In the models without general distress, negative self-esteem and aggression were additionally very highly interrelated, at age 17 (see Figure 10). In the models with general distress, negative self-esteem and general distress were also strongly interrelated (see Figure 11).

Table 7

Expected Influence (EI) Stability

**CA**	**Age**	**MDP_{EI}**	**Case range for MDP_{EI}**	**MDP_{EI}**	**Case range for MDP_{EI}**
		Without general distress		*With general distress*	
Yes	14	0.750	(caseMin = 0.721, caseMax = 1)	0.750	(caseMin = 0.721, caseMax = 1)
No	14	0.749	(caseMin = 0.721, caseMax = 1)	0.749	(caseMin = 0.721, caseMax = 1)
Yes	17	0.750	(caseMin = 0.721, caseMax = 1)	0.750	(caseMin = 0.721, caseMax = 1)
No	17	0.749	(caseMin = 0.721, caseMax = 1)	0.749	(caseMin = 0.721, caseMax = 1)

Note. CA = childhood adversity. MDP = Maximum drop proportion.

CA+	CA-

Age 14, without general distress

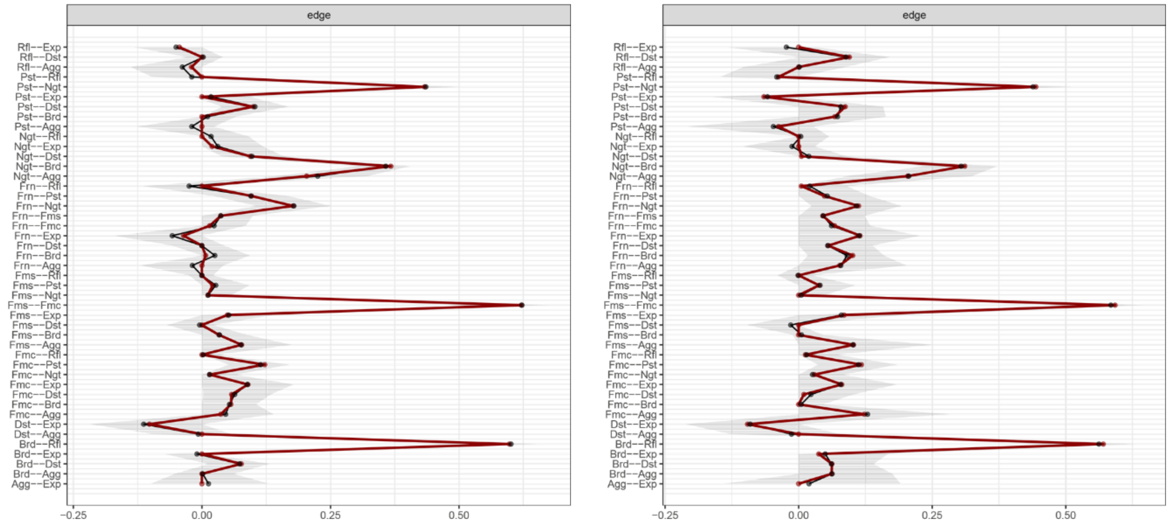

Age 17, without general distress

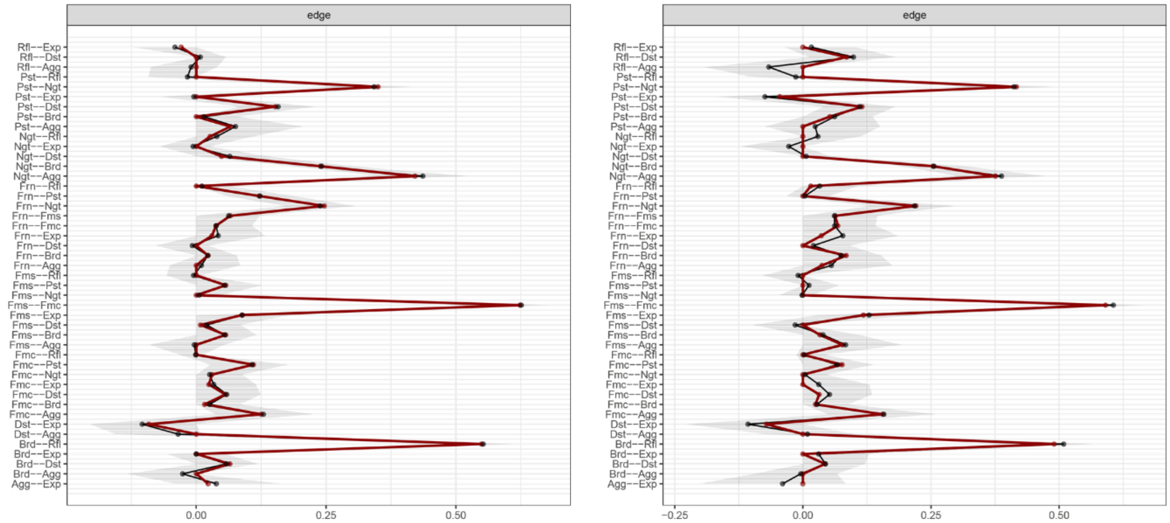

Figure 10. Interrelation accuracy plots for CA+ (*n* = 631) and CA- (*n* = 499) networks without the general distress variable, for both age 14 and age 17. The plots depict the sample RF interrelations (i.e. edge weights) which are represented by the red dots, the means of the bootstrapped RF interrelations (i.e. edge weights) which are represented by the black dots, and the belonging bootstrap confidence intervals (CIs) which indicate the RF interrelation accuracy.

CA+	CA-

Age 14, with general distress

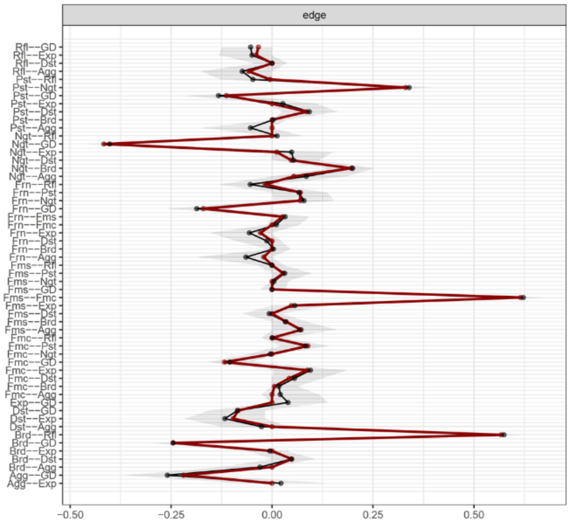

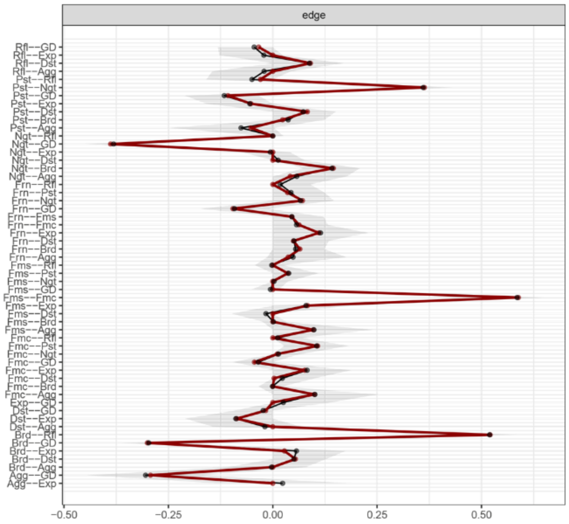

Age 17, with general distress

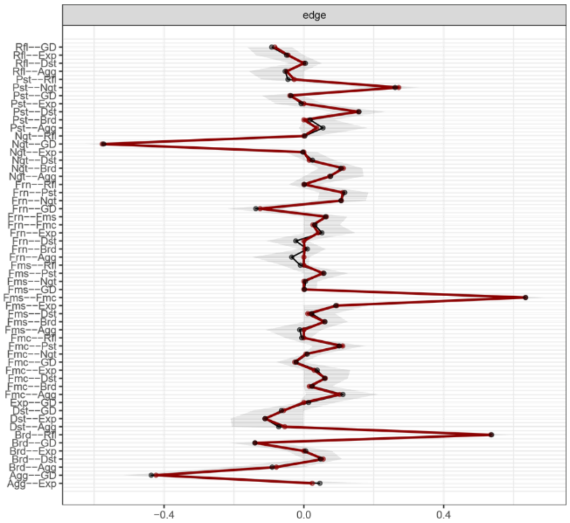

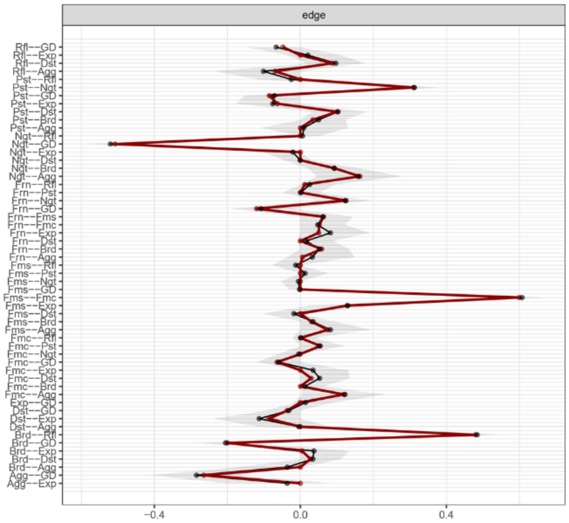

Figure 11. Interrelation accuracy plots for CA+ (*n* = 631) and CA- (*n* = 499) networks with the general distress variable, for both age 14 and age 17. The plots depict the sample 'RF-RF' and 'RF-general distress' interrelations (i.e. edge weights) which are represented by the red dots, the means of the bootstrapped interrelations (i.e. edge weights) which are represented by the black dots, and the belonging bootstrap confidence intervals (CIs) which indicate the 'RF-RF' and 'RF-general distress' interrelation accuracy.
