# Supplementary material for: Unravelling the complex nature of resilience factors and their changes between early and later adolescence
Source: BMC Med. 2019 Nov 14;17:203. doi: 10.1186/s12916-019-1430-6 (PMC6854636; doi:10.1186/s12916-019-1430-6)
Supplement: Supplementary file 10 — Additional file 10. Expected influence (EI) for RFs in networks corrected for general distress. [file 12916_2019_1430_MOESM10_ESM.pdf]

Additional file X

The connectivity analyses for expected influence (EI) coefficients showed that the family, ruminative brooding and negative self-esteem RFs had the highest coefficients, in both groups and at both time points (see Table 8). For both group, expressive suppression had the lowest EI coefficient, at both time points. We did not detect any particular age or group patterns (see Figure 12).

Table 8

Expected Influence (EI) for Networks Corrected for General Distress

**CA**	**frn**	**fms**	**fmc**	**pst**	**ngt**	**brd**	**rfl**	**dst**	**agg**	**exp**
yes: age 14	0.10	0.83	0.84	0.60	0.71	0.85	0.45	0.13	0.05	-0.02
yes: age 17	0.35	0.92	0.99	0.71	0.59	0.70	0.41	0.13	0.06	0.03
no: age 14	0.48	0.85	0.95	0.51	0.63	0.84	0.58	0.19	0.22	0.16
no: age 17	0.37	0.90	0.86	0.44	0.69	0.73	0.51	0.16	0.29	0.03

Note. CA = Childhood adversity (yes: *n* = 631, no: *n* = 499). Frn = friend support, fms = family support, fmc = family cohesion, pst = positive self-esteem, ngt = negative self-esteem, brd = brooding, rfl = reflection, dst = distress tolerance, agg = aggression, exp = expressive suppression.

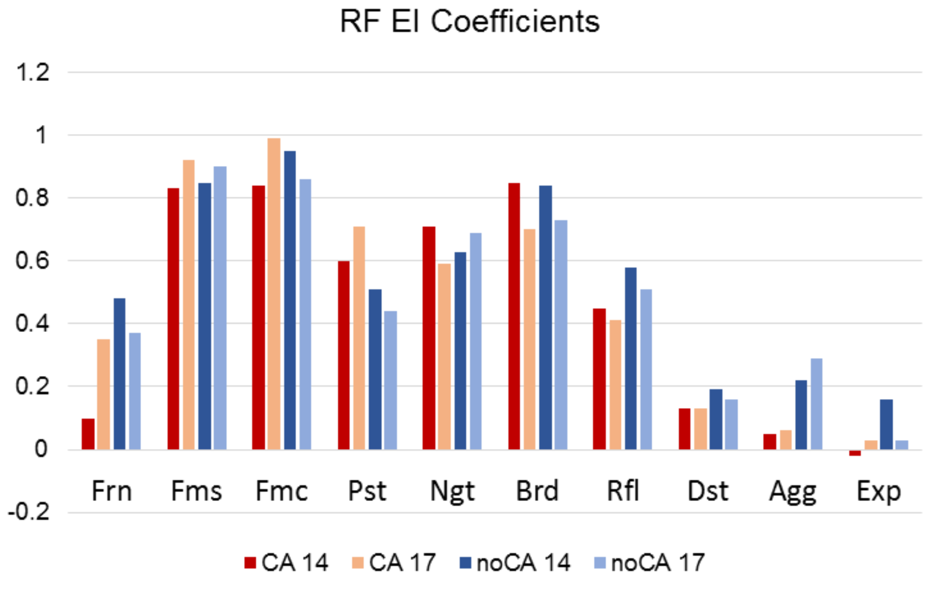

Figure 12. Visualizing expected influence (EI) coefficients for CA+ (*n* = 631) and CA- (*n* = 499) networks corrected for the general distress variable, for both age 14 and age 17. Frn = friend support, fms = family support, fmc = family cohesion, pst = positive self-esteem, ngt = negative self-esteem, brd = brooding, rfl = reflection, dst = distress tolerance, agg = aggression, exp = expressive suppression.
