# Supplementary material for: Unravelling the complex nature of resilience factors and their changes between early and later adolescence
Source: BMC Med. 2019 Nov 14;17:203. doi: 10.1186/s12916-019-1430-6 (PMC6854636; doi:10.1186/s12916-019-1430-6)
Supplement: Supplementary file 12 — Additional file 12. Network analysis results conducted with sum scores. [file 12916_2019_1430_MOESM12_ESM.pdf]

Additional file XII

The following three figures depict CA+ and CA- networks with sum scores. The models were estimated separately for age 14 and 17, as well as (1) once without the general distress variable, (2) once with the general distress variable, and (3) once corrected for the general distress variable. At age 14, the network invariance test was not significant for the networks without the general distress variable (M = .12, p = .58; see Figure 16), and the global network expected influence differed marginally between the CA+ and the CA- RF networks (EI_{CA+} = 3.01, EI_{CA-} = 3.31, EI = 0.31, p = .08). More specifically, the RFs in the CA+ network were less positively interrelated. Those findings were only partially similar in the networks for age 17, as neither of the two tests revealed significant differences between the CA+ and the CA- group (M = .13, p = .48; EI_{CA+} = 3.43, EI_{CA-} = 3.33, EI = 0.10, p = .49). When we compared the RF networks for age 14 and age 17, the two CA+ network were not invariant over time, in other words, they did vary over time (M = .24, p = .001). Moreover, the RFs in the age 14 network were less positively interrelated than in the age 17 network (EI_{14} = 3.01, EI_{17} = 3.43, EI = 0.42, p < .001). The two CA- network were not invariant over time (M = .22, p = .03), but did not differ in expected influence (EI_{14} = 3.31, EI_{17} = 3.33, EI = 0.02, p = .93).

For the networks with the general distress variable, the network invariance test (M = .13, p = .35; see Figure 17) was not significant at age 14. However, the global network expected influence differed between the CA+ and the CA- networks (EI_{CA+} = 0.67, EI_{CA-} = 1.20, EI = 0.53, p = .05). Those findings were only partially similar in the networks for age 17, as neither of the two tests revealed significant differences between the CA+ and the CA- group (M = .13, p = .60; EI_{CA+} = 1.22, EI_{CA-} = 1.27, EI = 0.05, p = .84). When we compared the CA+ networks for age 14 and age 17, the network invariance test was significant (M = .19, p = .02), and the RFs in the age 14 network were less positively interrelated than in the age 17 network (EI_{14} = 0.67, EI_{17} = 1.22, EI = 0.55, p = .04). The age 14 and age 17 CA- networks were again not invariant (M = .25, p = 0.009), but did not differ with regard to the expected influence (EI_{14} = 1.20, EI_{17} = 1.27, EI = 0.07, p = .80).

For the networks corrected for the general distress variable, the network invariance test was not significant at age 14 (M = .13, p = .31; see Figure 18), but the global network expected influence differed between the CA+ and the CA- networks (EI_{CA+} = 2.09, EI_{CA-} = 2.49, EI = 0.41, p = .04). Those findings were only partially similar in the networks for age 17, as neither of the two tests revealed significant differences between the CA+ and the CA- group (M = .13, p = .59; EI_{CA+} = 2.51, EI_{CA-} = 2.53, EI = 0.02, p = .90). When we compared the CA+ networks for age 14

and age 17, the network invariance test was not significant (M = .15, p = .12), but the RFs in the age 14 network were less positively interrelated than in the age 17 network (EI_{14} = 2.09, EI_{17} = 2.51, EI = 0.42, p = .02). The age 14 and age 17 CA- networks were invariant (M = .10, p = 0.90), and did not differ with regard to the expected influence (EI_{14} = 2.49, EI_{17} = 2.53, EI = 0.04, p = .85).

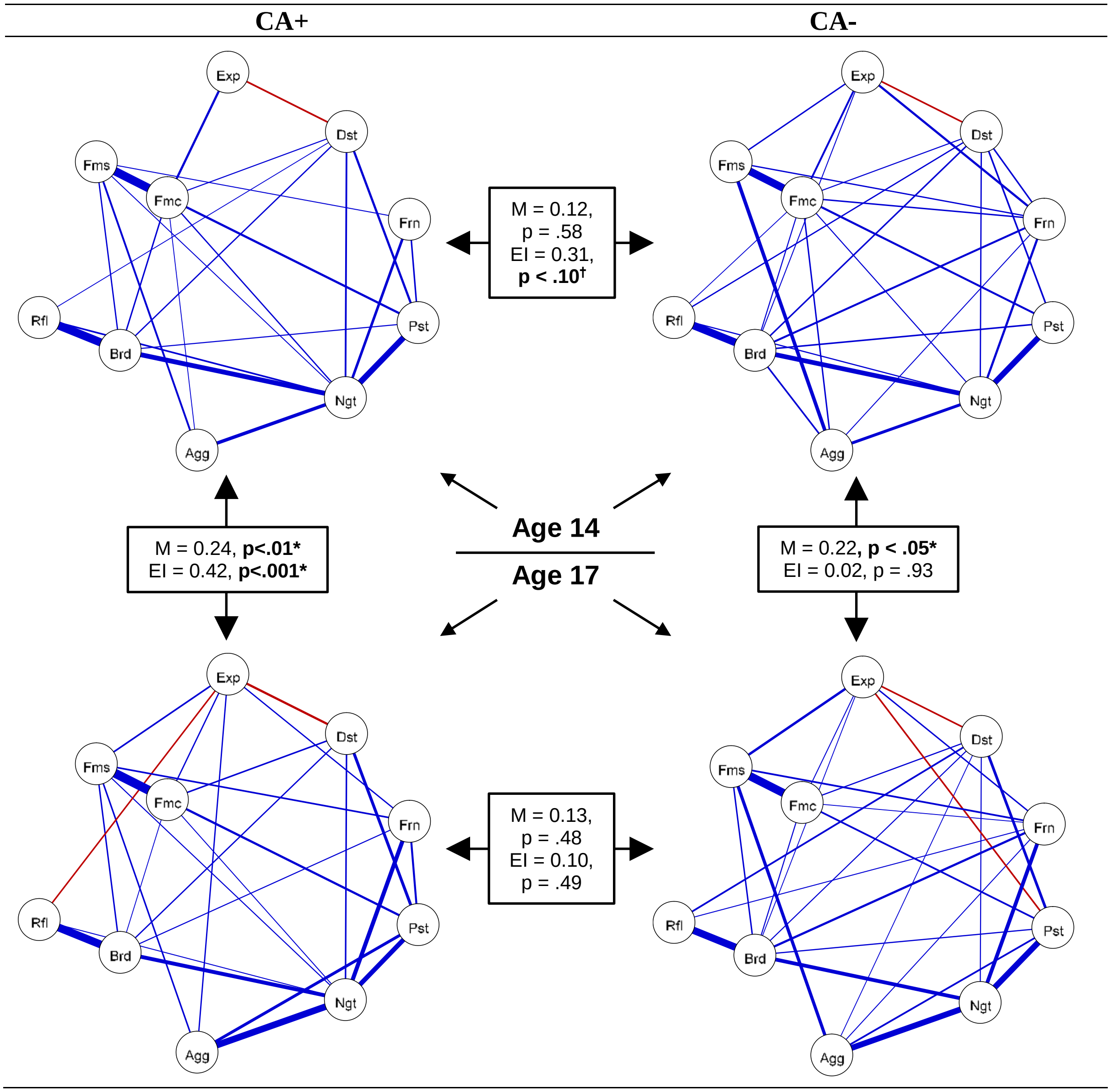

Figure 16. CA+ (*n* = 631) and CA- (*n* = 499) resilience factor networks with sum scores for age 14 (upper panel) and age 17 (lower panel) without the general distress variable. Width of the lines = association strength. Positive interrelations = blue, negative interrelations = red. **Legend:** Frn = friend support, fms = family support, fmc = family cohesion, ngt = negative self-esteem, pst = positive self-esteem, rfl = reflection, brd = brooding, dst = distress tolerance, agg = aggression, exp = expressive suppression. The boxes depict the maximal interrelation difference between the respective two networks (M), the difference in global network expected influence (EI) between the respective two networks (EI), and the corresponding p-values (5000 comparison samples).

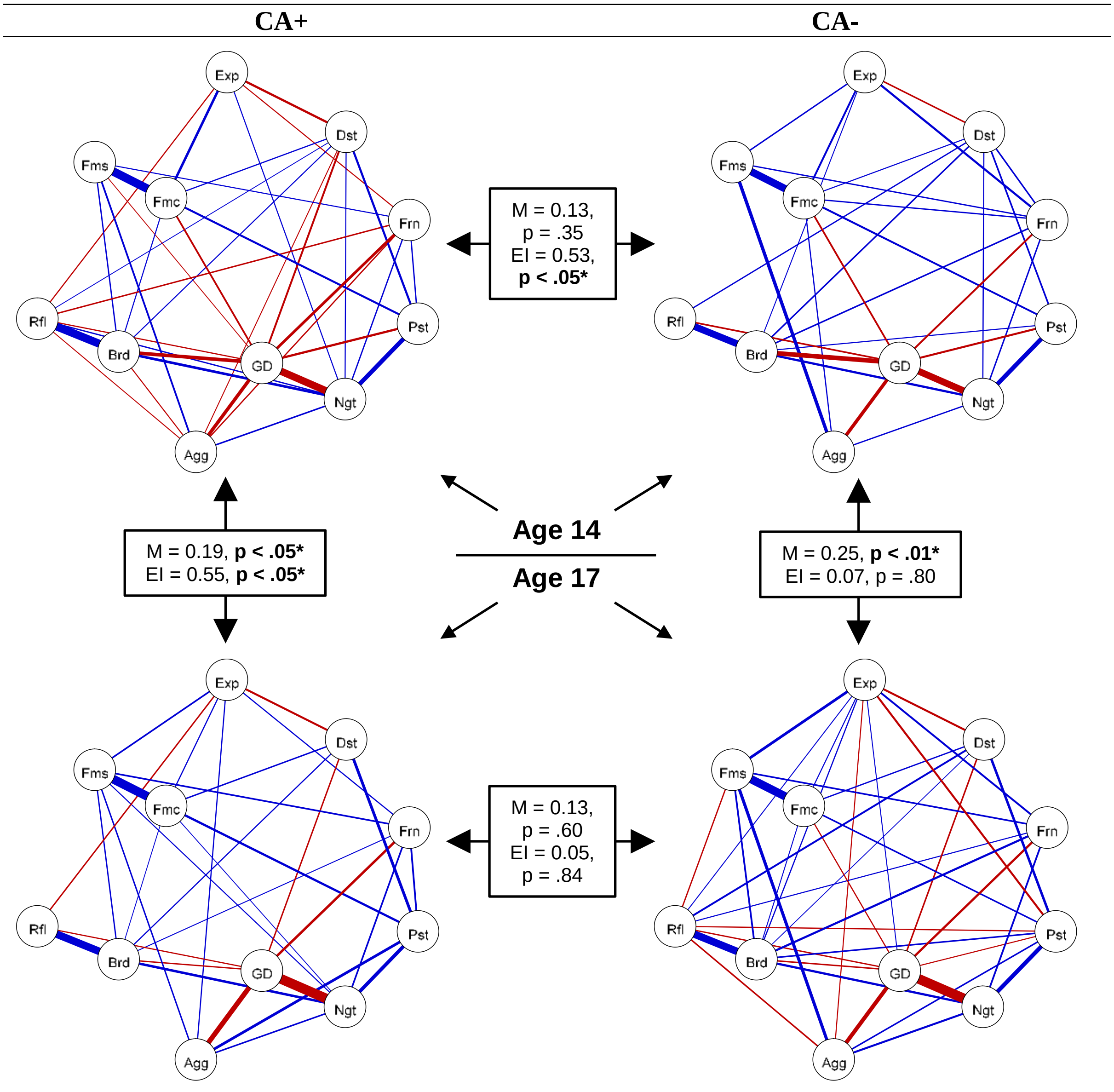

Figure 17. CA+ (*n* = 631) and CA- (*n* = 499) resilience factor networks with sum scores for age 14 (upper panel) and age 17 (lower panel) with the general distress variable. Width of the lines = association strength. Positive interrelations = blue, negative interrelations = red. **Legend:** Frn = friend support, fms = family support, fmc = family cohesion, ngt = negative self-esteem, pst = positive self-esteem, rfl = reflection, brd = brooding, dst = distress tolerance, agg = aggression, exp = expressive suppression, GD = general distress. The boxes depict the maximal interrelation difference between the respective two networks (M), the difference in global network expected influence (EI) between the respective two networks (EI), and the corresponding p-values (5000 comparison samples).

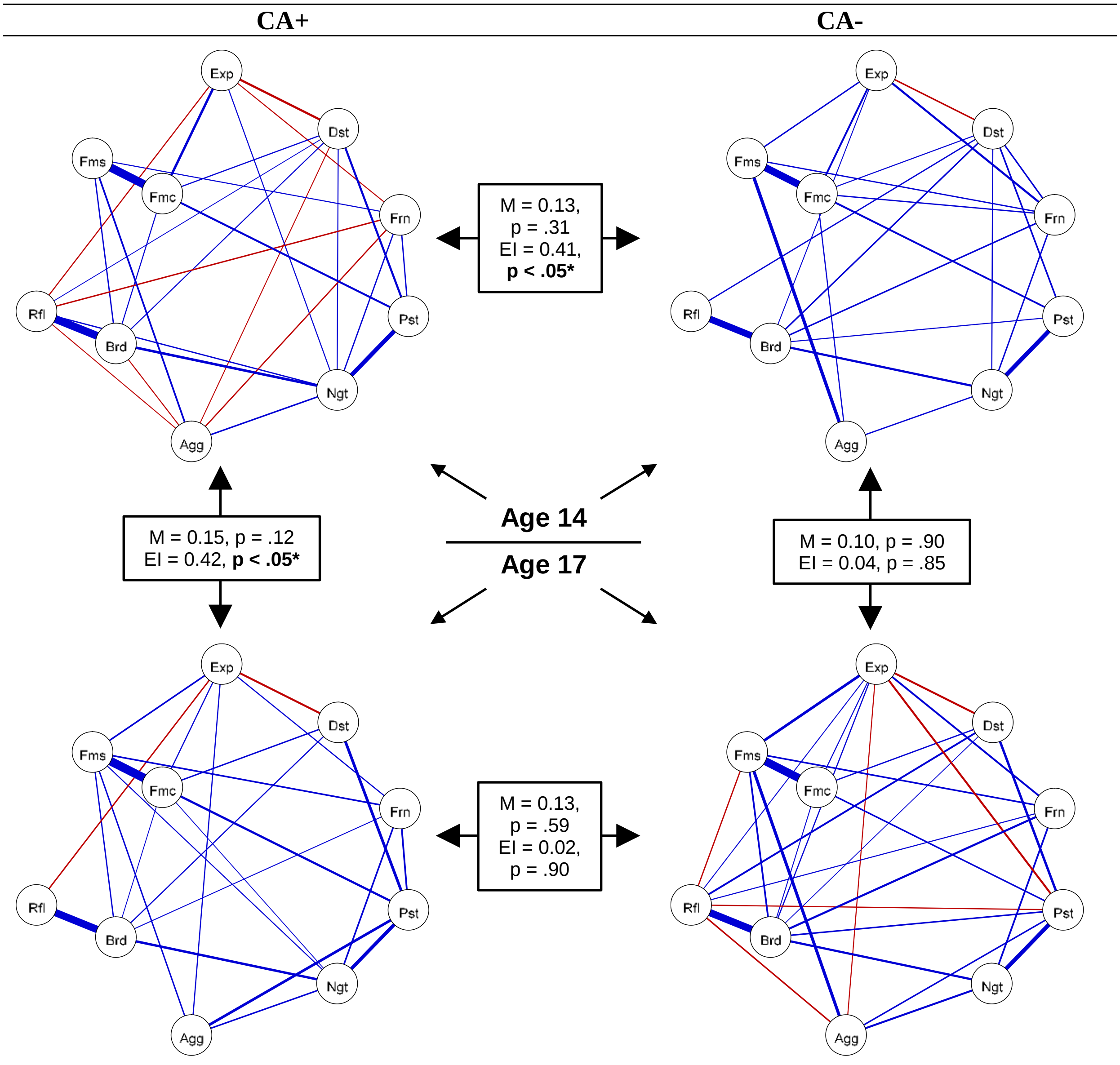

Figure 18. CA+ (*n* = 631) and CA- (*n* = 499) resilience factor networks for sum scores for age 14 (upper panel) and age 17 (lower panel) corrected for the general distress variable. Width of the lines = association strength. Positive interrelations = blue, negative interrelations = red. **Legend:** Frn = friend support, fms = family support, fmc = family cohesion, ngt = negative self-esteem, pst = positive self-esteem, rfl = reflection, brd = brooding, dst = distress tolerance, agg = aggression, exp = expressive suppression. The boxes depict the maximal interrelation difference between the respective two networks (M), the difference in global network expected influence (EI) between the respective two networks (EI), and the corresponding p-values (5000 comparison samples).
