# Supplementary material for: Unravelling the complex nature of resilience factors and their changes between early and later adolescence
Source: BMC Med. 2019 Nov 14;17:203. doi: 10.1186/s12916-019-1430-6 (PMC6854636; doi:10.1186/s12916-019-1430-6)
Supplement: Supplementary file 13 — Additional file 13. Network models presented in the main manuscript and in Additional file 7 with faded interrelations. [file 12916_2019_1430_MOESM13_ESM.pdf]

Additional file XIII

The following three figures depict CA+ and CA- networks with faded interrelations, for both age 14 and age 17, for (1) the networks without the general distress variable, (2) the networks with the general distress variable, and (3) the networks corrected for the general distress variable.

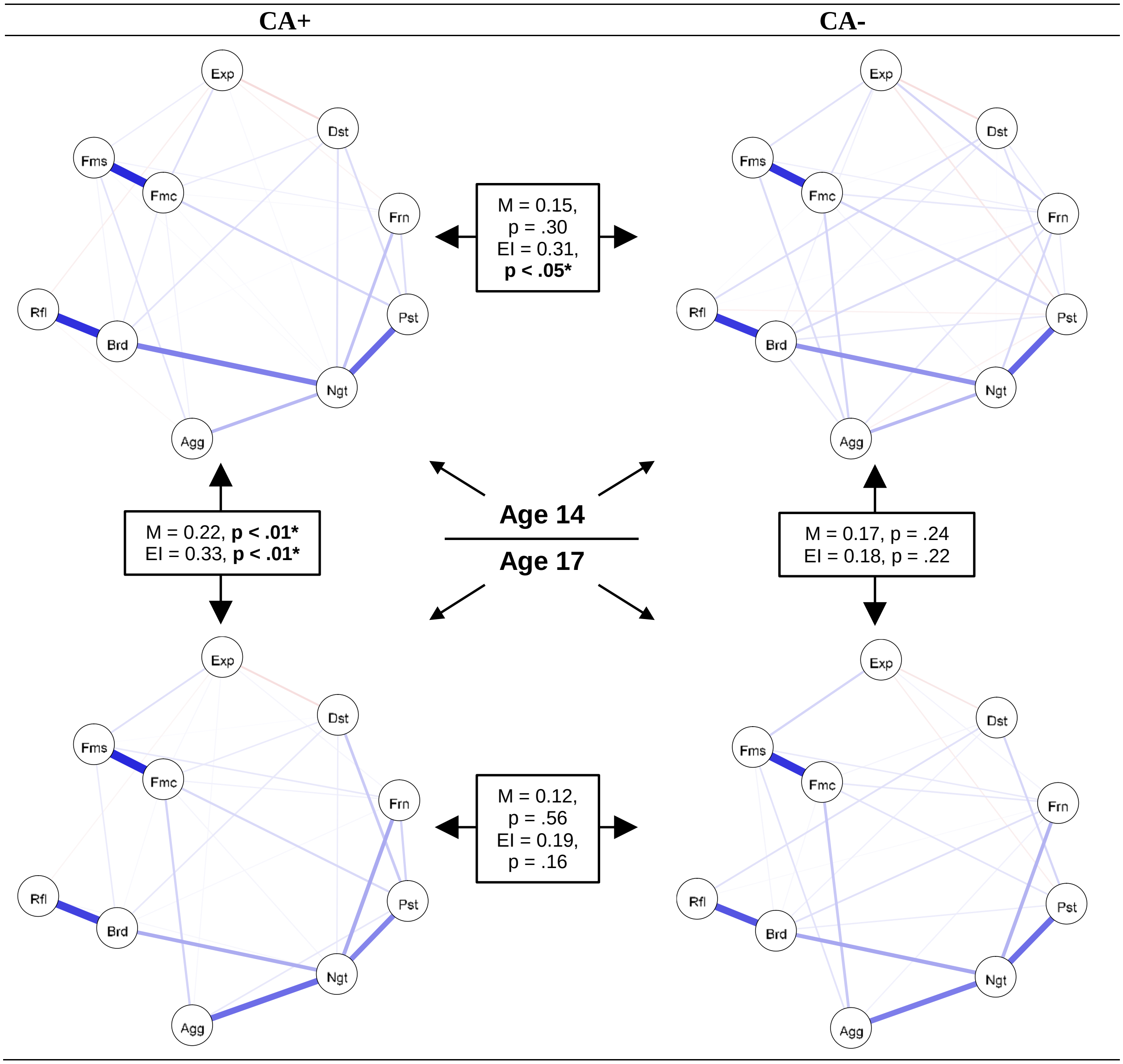

Figure 19. CA+ (*n* = 631) and CA- (*n* = 499) resilience factor networks with faded interrelations for age 14 (upper panel) and age 17 (lower panel) without the general distress variable. Width of the lines = association strength. Positive interrelations = blue, negative interrelations = red. **Legend:** Frn = friend support, fms = family support, fmc = family cohesion, ngt = negative self-esteem, pst = positive self-esteem, rfl = reflection, brd = brooding, dst = distress tolerance, agg = aggression, exp = expressive suppression. The boxes depict the maximal interrelation difference between the respective two networks (M), the difference in

global network expected influence (EI) between the respective two networks (EI), and the corresponding p-values (5000 comparison samples).

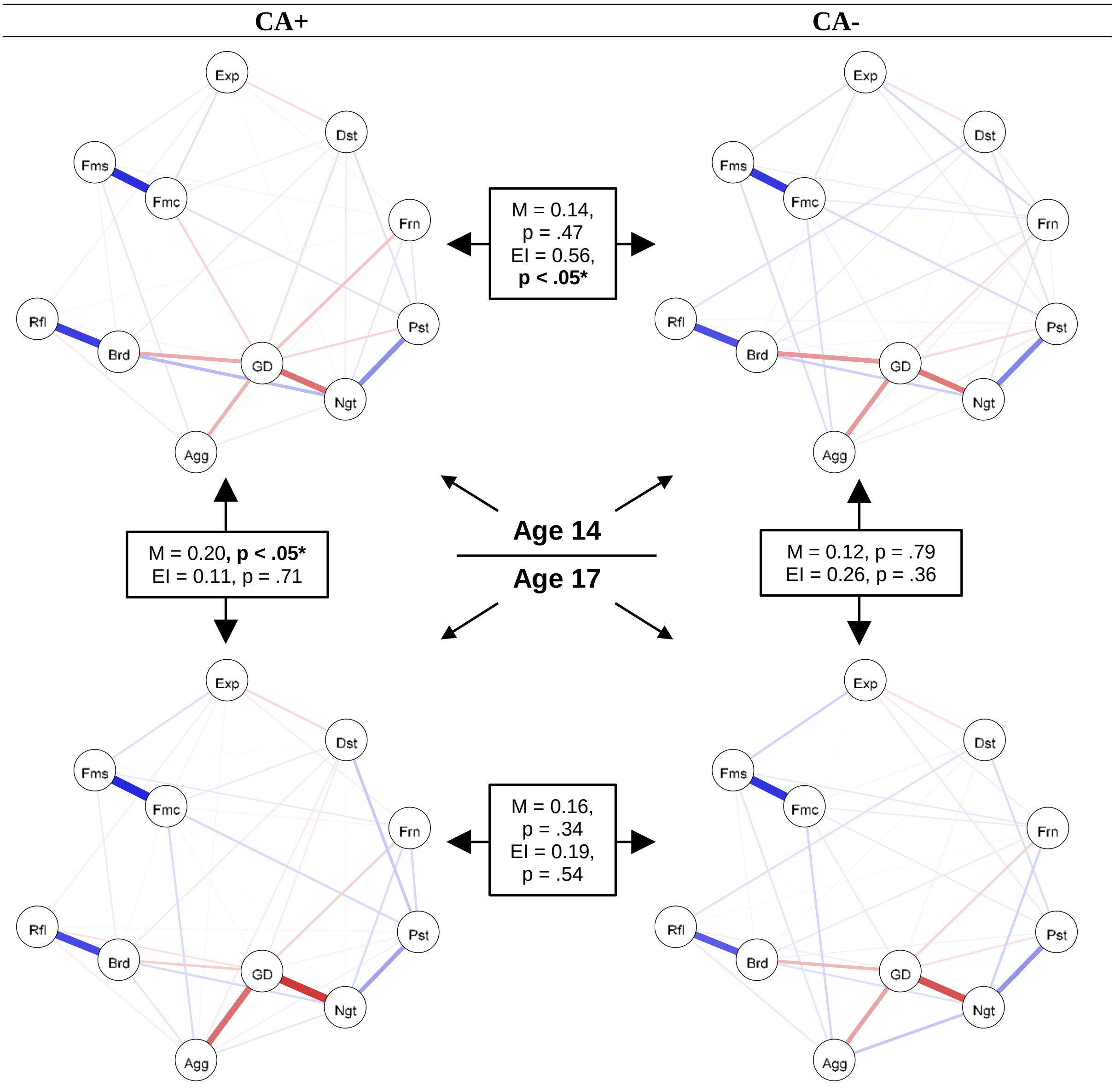

Figure 20. CA+ (*n* = 631) and CA- (*n* = 499) resilience factor networks with faded interrelations for age 14 (upper panel) and age 17 (lower panel) with the general distress variable. Width of the lines = association strength. Positive interrelations = blue, negative interrelations = red. **Legend:** Frn = friend support, fms = family support, fmc = family cohesion, ngt = negative self-esteem, pst = positive self-esteem, rfl = reflection, brd = brooding, dst = distress tolerance, agg = aggression, exp = expressive suppression, GD = general distress. The boxes depict the maximal interrelation difference between the respective two networks (M), the difference in global network expected influence (EI) between the respective two networks (EI), and the corresponding p-values (5000 comparison samples).

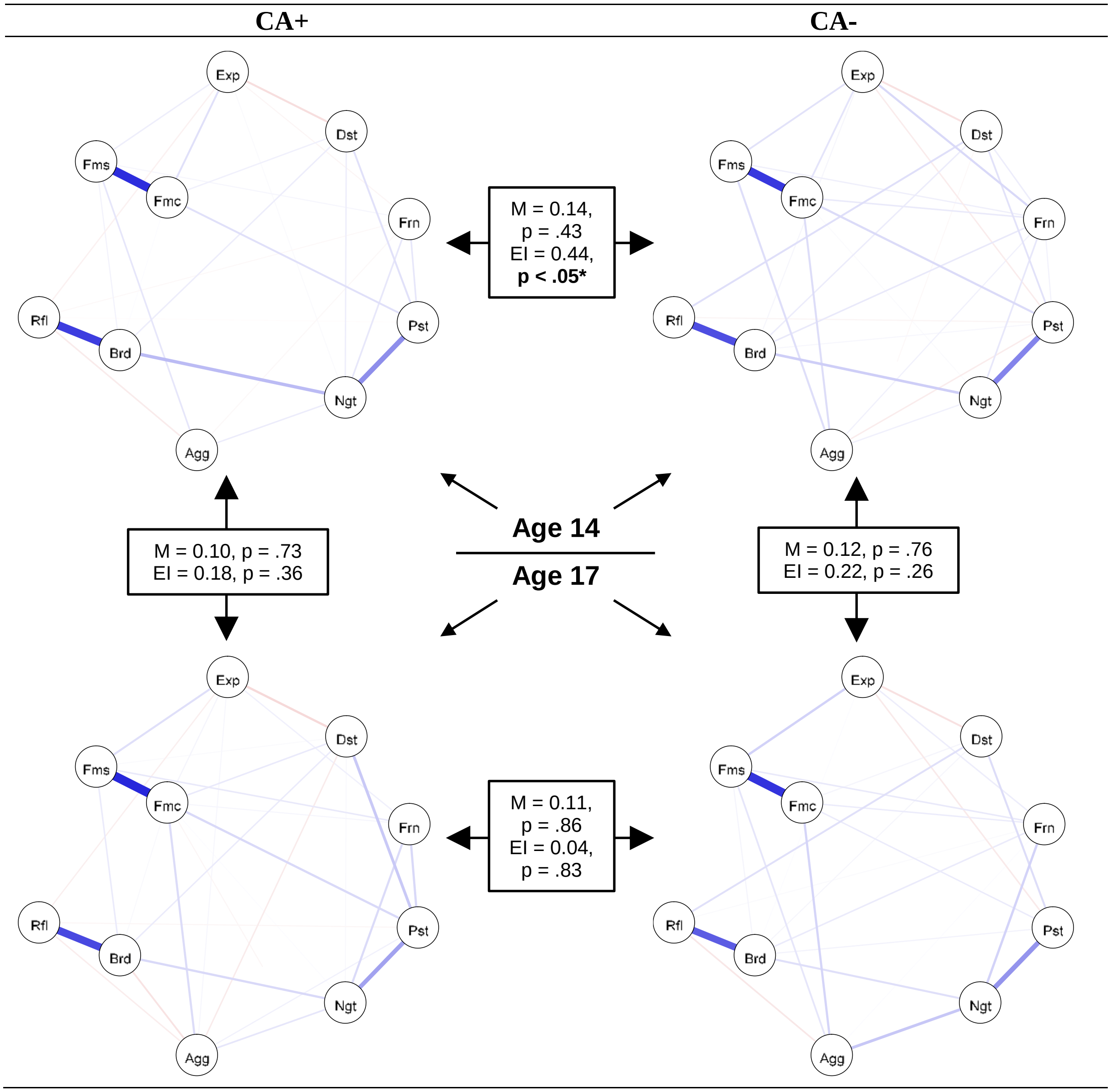

Figure 21. CA+ (*n* = 631) and CA- (*n* = 499) resilience factor networks with faded interrelations for age 14 (upper panel) and age 17 (lower panel) corrected for the general distress variable. Width of the lines = association strength. Positive interrelations = blue, negative interrelations = red. **Legend:** Frn = friend support, fms = family support, fmc = family cohesion, ngt = negative self-esteem, pst = positive self-esteem, rfl = reflection, brd = brooding, dst = distress tolerance, agg = aggression, exp = expressive suppression, GD = general distress. The boxes depict the maximal interrelation difference between the respective two networks (M), the difference in global network expected influence (EI) between the respective two networks (EI), and the corresponding p-values (5000 comparison samples).
