# Supplementary material for: Unravelling the complex nature of resilience factors and their changes between early and later adolescence
Source: BMC Med. 2019 Nov 14;17:203. doi: 10.1186/s12916-019-1430-6 (PMC6854636; doi:10.1186/s12916-019-1430-6)
Supplement: Supplementary file 14 — Additional file 14. Similarity and differences to Figures in a previous report on this sample. [file 12916_2019_1430_MOESM14_ESM.pdf]

Additional file XIV

Several network figures for age 14 (i.e. Figure 2 and 3 in the main manuscript, and figures in Supplement II, V, VII, IX, XI, XII, XIII) are similar to figures in a previous report on this sample Fritz et al. (2018; Scientific Reports; can be retrieved from https://doi.org/10.1038/s41598-018-34130-2)[1]. In the original article, the figures were published under the Creative Commons Attribution 4.0 International License. Information about this license can be found in the article itself[1] or at http://creativecommons.org/licenses/by/4.0/. The figures here are however only partially similar to the figures in our previous report, for the following reasons: (1) a slightly different sample was used as we could impute the missing data, (2) the general distress variable was not the same for reasons described in Supplement I, (3) the brooding variable was not the same for reasons described in Supplement I, (4) the scores were not derived from one-factor CFAs, but from longitudinal categorical CFAs with two factors, one for each time point, and (5) due to computing different CFA models, some CFAs did not need the modification we had to apply for the CFAs in our previous report.
