# Supplementary material for: Unravelling the complex nature of resilience factors and their changes between early and later adolescence
Source: BMC Med. 2019 Nov 14;17:203. doi: 10.1186/s12916-019-1430-6 (PMC6854636; doi:10.1186/s12916-019-1430-6)
Supplement: Supplementary file 15 — Additional file 15. Significant RF-RF interrelation differences (a) between the CA+ (n = 631) and the CA- (n = 499) networks, as well as (b) between age 14 and age 17 networks. [file 12916_2019_1430_MOESM15_ESM.pdf]

Additional file XV

Table 9

Significant RF-RF Interrelation Differences between the CA+ (n = 631) and the CA- (n = 499) Networks

RF1	RF2	interrelation sign in the CA+ network	interrelation sign in the CA- network	E	*p*
			Age 14		
friendship support	brooding	null	positive	0.07	.014
friendship support	expressive suppression	negative	positive	0.14	.007
positive self-esteem	expressive suppression	null	negative	0.05	.046
reflection	distress tolerance	null	positive	0.09	.015
			Age 17		
friendship support	positive self-esteem	positive	null	0.11	.031

Note. RF = Resilience factor. CA = childhood adversity. E = RF-RF interrelation difference (i.e. edge difference).

Table 10

Significant RF-RF Interrelation Differences between Age 14 and Age 17 Networks

RF1	RF2	interrelation sign in the age 14 network	interrelation sign in the age 17 network	E	*p*
			CA+ Networks		
friendship support	expressive suppression	negative	positive	0.07	.031
negative self-esteem	brooding	more positive	less positive	0.08	.019
			CA- Networks		
-	-	-	-	-	-

Note. RF = Resilience factor. CA = childhood adversity. E = RF-RF interrelation difference (i.e. edge difference).
