# Supplementary material for: Unravelling the complex nature of resilience factors and their changes between early and later adolescence
Source: BMC Med. 2019 Nov 14;17:203. doi: 10.1186/s12916-019-1430-6 (PMC6854636; doi:10.1186/s12916-019-1430-6)
Supplement: Supplementary file 16 — Additional file 16. Debate: Are resilience and risk factors opposing sides of the same coin? [file 12916_2019_1430_MOESM16_ESM.pdf]

Additional file XVI

Are resilience and risk factors opposing sides of the same coin?

The question whether resilience and risk factors lie on one continuum, representing respectively the opposite ends, has been widely discussed in the resilience literature. Yet, the answer to this question is probably not done justice with a simple yes or no. For our study the resilience factors (RFs) were derived from our systematic review[29] and were defined as follows (p. 2): "*RFs have a promotive impact on the adjustment process following CA and thus help individuals to adapt and recover from the sequelae of CA (Rutter, 1985, 2013; Zolkoski & Bullock, 2012).*[30–32] *Statistically, RFs operate as a moderator (Fergus & Zimmerman, 2005; Rutter, 1985)*[30,33]*, and/ or as a positive mediator (Masten, 2001; van Harmelen et al., 2016)*[34,35] *for the relationship between CA and psychopathology.*" We further specified as inclusion criteria that an RF "*belongs either to the individual-, family-, or community-level category, […] belongs to the cognitive, behavioural, social, and/ or emotional functioning domain, [… and should be] amenable"* (p. 3 in [29]). We specified as exclusion criteria that the RF should not be "*defined (a) as financial advantage, (b) as no re-victimization, (c) as inverse of CA, [and] (d) as inverse of psychopathology*" (p. 3 in [29]). In other words, RFs are amenable factors that operate as ameliorating or modifying variables in the relationship between adversity and mental health problems, and should neither be equivalent to CA nor to mental health problems. CA was defined as "*traumatic and/or severely stressful events, [leading to]…a higher risk of developing mental health problems*" (p. 2 in [29]). We additionally specified in detail which events would qualify as CA, in the attempt to keep the definitions of RFs and CA as separate as possible. Yet, based on our definitions, adversity is not equivalent to risk factors, as risk factors do not need to be traumatic and/or severely stressful events but still lead to a higher risk of developing mental health problems (e.g. low maternal education). With regard to our study, the quick, but insufficient answer is probably that many (or most) of the investigated RFs are indeed the flip side of risk factors. For example, self-esteem (or a positive self-concept) is commonly defined as RF and has been discussed as such by many of the seminal resilience researchers, including Michael Rutter, Emmy Werner, Ann Masten, and Michael Ungar (for a review see e.g. [36]). Yet, at the same time a low level of self-esteem or self-worth is part of the DSM V criteria for depression ("Feelings of worthlessness"; American Psychiatric Association[37]). Hence, whereas a high level of self-esteem may protect against low mood levels, low self-esteem is assumed to contribute to or reflect low mood.

That said, some RFs have been suggested not to lie on the same continuum with their supposingly opposing risk factor. For example, Carretta and colleagues[38] showed that hope and hopelessness are highly negatively correlated, but not as high as would be expected for opposing poles of the same construct.[39] Others have proposed that not hopelessness and hope, but hopelessness and the absence of hopelessness may be bipolar.[39,40] Hence, here the risk factor may be hopelessness and the RF hope, which may however not lie on the exact same continuum. For other factors the liaison between risk and protection is even more complicated, as it depends on external factors. For instance, a low level of expressive suppression, which means that someone can effectively expresses and communicate his/her emotions, may well be advantageous in safe environments. Yet, in hazardous environments, as for example a violent home environment, emotional expression may not always be advantageous. Similarly, Luthar[41] found in adolescents from underprivileged environments that high intelligence functions as risk factor, rather than, as commonly found, as RF. Hence some RFs may be protective in one context or environment but may be harmful in another.[36] Moreover, some RFs may be particularly protective during early development and others during adulthood. For example, some researchers argue that parental support is particularly protective during childhood, but less so during adolescence.[42]

Sometimes researchers differentiate between RFs and risk factors through defining risk factors as direct effects, and RFs as moderators or mediators (e.g. see [43]). Such attempts are limited in our opinion, as both mediating and moderating RFs statistically also require a direct effect between the RF and the mental health outcome. Hence, according to such a definition one would suggest that all factors that qualify as direct effect and as mediator and/or moderator should be clustered into the RF category, whereas all factors that only qualify as direct effect should be clustered into the risk factor category. One crucial consideration that limits this definition is the present lack of replicability of RFs and risk factors. For example, Dubow and colleagues[44] found that positive parenting moderates the relationship between CA and mental health problems, while Cui and Conger[45] did not find convincing support for a moderation effect. Thus, now one would be stuck with deciding on whether high positive parenting should be considered as RF or whether low positive parenting should be considered as risk factor. Other resilience researchers have argued that both direct effects and mediating and moderating effects qualify as RFs. For example, Garmezy and colleagues[46] refer to RFs with a direct effect on mental health as “compensatory” factors and to RFs with an interaction effect on mental health as “protective” factors.

In sum, we cautiously conclude that on the group level (particularly when a rather homogeneous group is studied) many RFs (such as those included in our study) operate on a continuum with risk factors. Yet, on an individual level, the relationship between resilience and risk factors is likely to additionally depend on biological predispositions, type of adversity experienced, the specific environmental circumstances, and the developmental stage.

While neither our RF definition nor our analyses allow us to clearly demarcate the conceptualisation of resilience vs risk factors, we believe that our work expands the RF literature on another aspect. Shaik and Kauppi (p. 162-163 in [43]) state that "*[o]ne of the major shortcomings is the tendency to view factors as mono-directional influences as opposed to bi-directional influences (Glantz & Sloboda, 1999)*[47]*. These models fail to delineate how all factors can be the influences, mediators and outcomes tied in varying degrees to the entire system of variables. Despite the large number of empirical studies […], there are not sufficient details available about how and why the protective or compensatory factors directly or indirectly influence the outcomes (Lepore & Revenson, 2006)*[48]." We believe that the strength of our manuscript lies in shedding light onto the bi-directional system of RFs that are associated with a lower risk of mental distress during early and later adolescence. Regardless of whether resilience and risk factors operate on the same continuum or are inversely correlated but not identical, understanding the nature of RFs seems to have universal appeal as it focuses on what promotes good mental health rather than on what increases mental health problems. Knowledge on the promotion of good mental health in adolescents may not only be of clinical, but also of policy interest, as good mental health in today's youth may result in less mental health problems in tomorrow's adults. Or to put it into Garmezy's words (p. 171 in [49]): "*Government, by providing protective factors, enables some who would otherwise be lost to a fruitful life to move above the threshold of competence needed to survive in an increasingly complex, technological society.*"
