# Supplementary material for: Unravelling the complex nature of resilience factors and their changes between early and later adolescence
Source: BMC Med. 2019 Nov 14;17:203. doi: 10.1186/s12916-019-1430-6 (PMC6854636; doi:10.1186/s12916-019-1430-6)
Supplement: Supplementary file 17 — Additional file 17. References for the additional files. [file 12916_2019_1430_MOESM17_ESM.pdf]

Additional file XVII

References for the additional files

1. Fritz, J., Fried, E. I., Goodyer, I. M., Wilkinson, P. O. & van Harmelen, A.-L. A Network Model of Resilience Factors for Adolescents with and without Exposure to Childhood Adversity. *Sci. Rep.* **8,** 15774 (2018).
2. Brodbeck, J., Abbott, R. A., Goodyer, I. M. & Croudace, T. J. General and specific components of depression and anxiety in an adolescent population. *BMC Psychiatry* **11,** 191 (2011).
3. Burwell, R. A. & Shirk, S. R. Subtypes of rumination in adolescence: Associations between brooding, reflection, depressive symptoms, and coping. *J. Clin. Child Adolesc. Psychol.* **36,** 56–65 (2007).
4. van Buren, S. & Groothuis-Oudshoorn, K. mice: Multivariate Imputation by Chained Equations in R. *J. Stat. Softw.* **45,** 1–67 (2011).
5. Wickham, H., François, R., Henry, L. & Müller, K. dplyr: A Grammar of Data Manipulation. R package version 0.7.7. (2018). Available at: https://cran.r-project.org/package=dplyr.
6. Grosjean, P. & Ibanez, F. pastecs: Package for Analysis of Space-Time Ecological Series. R package version 1.3.21. (2018). Available at: https://cran.r-project.org/package=pastecs.
7. Hothorn, T., Hornik, K., van de Wiel, M. A. & Zeileis, A. Implementing a Class of Permutation Tests: The coin Package. *J. Stat. Softw.* **28,** 1–23 (2008).
8. Wickham, H. Reshaping data with the reshape package. *J. Stat. Softw.* **21,** 2007 (2007).
9. Lüdecke, D. sjPlot: Data Visualization for Statistics in Social Science. R package version 2.6.2. (2018).
10. Rosseel, Y. lavaan: An R package for structural equation modeling. *J. Stat. Softw.* **48,** 1–36. Retrieved from http://www.jstatsoft.org/v48/ (2012).
11. Jorgensen, T. D., Pornprasertmanit, S., Schoemann, A. M. & Rosseel, Y. semTools: Useful tools for structural equation modeling. R package version 0.5-1.905. (2018). Available at: https://cran.r-project.org/package=semTools.
12. Wickham, H. *ggplot2: Elegant Graphics for Data Analysis.* (Springer-Verlag, 2016).
13. Epskamp, S., Cramer, A. O. J., Waldorp, L. J., Schmittmann, V. D. & Borsboom, D. qgraph: Network Visualizations of Relationships in Psychometric Data. *J. Stat. Softw.* **48,** 1–18 (2012).
14. Epskamp, S., Borsboom, D. & Fried, E. I. Estimating Psychological Networks and their Accuracy : A Tutorial Paper. *Behav. Res. Methods* **50,** 195–212 (2018).
15. van Borkulo, C. D. *Comparing network structures on three aspects: A permutation test (PhD Thesis*

Chapter 5). (University of Groningen, 2018).

16. Epskamp, S., Rhemtulla, M. & Borsboom, D. Generalized Network Psychometrics: Combining Network and Latent Variable Models. *Psychometrika* **82,** 904–927 (2017).

17. Liu, Y. *et al.* Testing measurement invariance in longitudinal data with ordered-categorical measures. *Psychol. Methods* **22,** 486–506 (2017).

18. Muthen, B. & Asparouhov, T. Latent Variable Analysis With Categorical Outcomes: Multiple-Group And Growth Modeling In Mplus. *Mplus Web Notes No. 4* Retrieved from https://www.statmodel.com/download/ (2002).

19. Sass, D. A. Testing measurement invariance and comparing latent factor means within a confirmatory factor analysis framework. *J. Psychoeduc. Assess.* **29,** 347–363 (2011).

20. Wu, H. & Estabrook, R. Identification of Confirmatory Factor Analysis Models of Different Levels of Invariance for Ordered Categorical Outcomes. *Psychometrika* **81,** 1014–1045 (2016).

21. Goodyer, I. M., Wright, C. & Altham, P. M. E. Recent friendships in anxious and depressed school age children. *Psychol. Med.* **19,** 165–174 (1989).

22. Epstein, N. B., Baldwin, L. M. & Bishop, D. S. The McMaster Family Assessment Device. *J. Marital Fam. Ther.* **9,** 171–180 (1983).

23. Rosenberg, M. *Society and the Adolescent Self-Image*. (Princeton, NJ: Princeton University Press, 1965).

24. Treynor, W., Gonzalez, R. & Nolen-Hoeksema, S. Rumination reconsidered: A psychometric analysis. *Cognit. Ther. Res.* **27,** 247–259 (2003).

25. Bould, H., Joinson, C., Sterne, J. & Araya, R. The Emotionality Activity Sociability Temperament Survey: Factor analysis and temporal stability in a longitudinal cohort. *Pers. Individ. Dif.* **54,** 628–633 (2013).

26. Goodyer, I. M. *et al.* Improving mood with psychoanalytic and cognitive therapies (IMPACT): a pragmatic effectiveness superiority trial to investigate whether specialised psychological treatment reduces the risk for relapse in adolescents with moderate to severe unipolar dep... *Trials* **12,** 175 (2011).

27. Messer, S. C., Angold, A. & Costello, E. J. Development of a Short Questionnaire for Use in Epidemiological Studies of Depression in Children and Adolescents: Factor Composition and Structure across Development. *Int. J. Methods Psychiatr. Res.* **5,** 251–262 (1995).

28. Reynolds, C. R. & Richmond, B. O. What I Think and Feel: A Revised Measure of Children's Manifest

Anxiety. *J. Abnorm. Child Psychol.* **6,** 271–280 (1978).

29. Fritz, J., de Graaff, A. M., Caisley, H., van Harmelen, A.-L. & Wilkinson, P. O. A Systematic Review of Amenable Resilience Factors that Moderate and/or Mediate the Relationship between Childhood Adversity and Mental Health in Young People. *Front. Psychiatry* **9,** 230 (2018).
30. Rutter, M. Resilience in the Face of Adversity: Protective Factors and Resistance to Psychiatric Disoder. *Br. J. Psychiatry* **147,** 598–611 (1985).
31. Rutter, M. Annual Research Review: Resilience – clinical implications. *J. Child Psychol. Psychiatry* **54,** 474–487 (2013).
32. Zolkoski, S. M. & Bullock, L. M. Resilience in children and youth: A review. *Child. Youth Serv. Rev.* **34,** 2295–2303 (2012).
33. Fergus, S. & Zimmerman, M. A. Adolescent Resilience: A Framework for Understanding Healthy Development in the Face of Risk. *Annu. Rev. Public Heal.* **26,** 399–419 (2005).
34. Masten, A. S. Ordinary Magic: Resilience Processes in Development. *Am. Psychol.* **56,** 227–238 (2001).
35. van Harmelen, A.-L. *et al.* Friendships and Family Support Reduce Subsequent Depressive Symptoms in At-Risk Adolescents. *PLoS One* **11,** e0153715 (2016).
36. VicHealth. *Current theories relating to resilience and young people: A literature review*. *Victorian Health Promotion Foundation: Melbourne, Australia* (2015).
37. American Psychiatric Association. *Diagnostic and statistical manual of mental disorders (5th ed.).* (Washington, DC: Author, 2013).
38. Carretta, C. M., Ridner, S. H. & Dietrich, M. S. Hope, hopelessness, and anxiety: A pilot instrument comparison study. *Arch. Psychiatr. Nurs.* **28,** 230–234 (2014).
39. Cheavens, J. S., Cukrowicz, K. C., Hansen, R. & Mitchell, S. M. Incorporating Resilience Factors Into the Interpersonal Theory of Suicide: The Role of Hope and Self-Forgiveness in an Older Adult Sample. *J. Clin. Psychol.* **72,** 58–69 (2016).
40. Grewal, P. K. & Porter, J. E. Hope theory: A framework for understanding suicidal action. *Death Stud.* **31,** 131–154 (2007).
41. Luthar, S. S. Vulnerability and Resilience: A Study of High-Risk Adolescents. *Child Dev.* **62,** 600–616 (1991).
42. Hostinar, C. E., Johnson, A. E. & Gunnar, M. R. Parent support is less effective in buffering cortisol stress reactivity for adolescents compared to children. *Dev. Sci.* **18,** 281–297 (2015).

43. Shaikh, A. & Kauppi, C. Deconstructing Resilience: Myriad Conceptualizations and Interpretations. *Int. J. Arts Sci.* **3,** 155–176 (2010).

44. Dubow, E. F. *et al.* Exposure to Political Conflict and Violence and Posttraumatic Stress in Middle East Youth: Protective Factors. *J. Clin. Child Adolesc. Psychol.* **41,** 402–416 (2012).

45. Cui, M. & Conger, R. D. Parenting Behavior as Mediator and Moderator of the Association Between Marital Problems and Adolescent Maladjustment. *J. Res. Adolesc.* **18,** 261–284 (2008).

46. Garmezy, N., Masten, A. S. & Tellegen, A. The Study of Stress and Competence in Children: A Building Block for Developmental Psychopathology. *Child Dev.* **55,** 97–111 (1984).

47. Glantz, M. D. & Sloboda, Z. Analysis and reconceptulization of resilience. in *Resilience and development: Positive life adaptations* (eds. Glantz, M. D. & Johnson, J. L.) 17–83 (New York: Kluwer Academic/Plenum Publishers., 1999).

48. Lepore, S. J. & Revenson, T. A. Reslience and posttraumatic growth: Recovery, resistance, and reconfiguration. in *Handbook of posttraumatic growth: research and practice* (eds. Calhoun, L. G. & Tedeschi, R. G.) 24–46 (New York: Routledge Taylor & Francis Group, 2006).

49. Garmezy, N. Stress, competence, and development: Continuities in the study of schizophrenic adults, children vulnerable to psychopathology, and the search for stress-resistant children. *Am. J. Orthopsychiatry* **57,** 159–174 (1987).
