# Supplementary material for: Unravelling the complex nature of resilience factors and their changes between early and later adolescence
Source: BMC Med. 2019 Nov 14;17:203. doi: 10.1186/s12916-019-1430-6 (PMC6854636; doi:10.1186/s12916-019-1430-6)
Supplement: Supplementary file 18 — Additional file 18. Supplementary materials: Analysis results based on imputed data. [file 12916_2019_1430_MOESM18_ESM.zip › 4_Manuscript_withoutImp_2019.08August.13.pdf]

Unravelling the Complex Nature of Resilience Factors and their Changes between Early and Later Adolescence

J. Fritz[1,*], J. Stochl[1,2], E. I. Fried[3], I. M. Goodyer[1], C. D. van Borkulo[4], P. O. Wilkinson[1,A], A.-L. van Harmelen[1,A]

* Correspondence: Jessica Fritz, jf585@cam.ac.uk

[1] University of Cambridge, Department of Psychiatry, United Kingdom

[2] Charles University, Department of Kinanthropology, Czech Republic

[3] Leiden University, Department of Clinical Psychology, the Netherlands

[4] University of Amsterdam, Department of Psychological Methods, the Netherlands

[A] shared last authorship

Childhood adversity (CA) is strongly associated with mental health problems. Resilience factors (RFs) reduce mental health problems following CA. Yet, knowledge on the nature of RFs is scarce. Therefore, we examined RF mean levels, RF interrelations, RF-distress pathways, and their changes between early (age 14) and later adolescence (age 17). Specifically, we studied 10 empirically-supported RFs in adolescents with (CA+; n=638) and without CA (CA-; n=501), using network psychometrics. All inter-personal RFs (e.g. friendships) showed stable mean levels between age 14 and 17, whereas five of seven intra-personal RFs (e.g. distress-tolerance) changed in a similar manner in both groups. The CA+ group had lower RFs and higher distress at both ages. Thus, CA does not seem to inhibit RF changes but seems to increase the risk of persistently lower RFs. At age 14, but not 17, the RF network of the CA+ group was less positively connected, suggesting that RFs are less likely to enhance each other than in the CA- group. Moreover, despite that the CA+ group had stronger negative RF-distress pathways, at age 14, those did not reduce distress to a similar mean level as in the CA- group. Those findings suggest that CA has a predominantly strong proximal effect. In contrast to RF mean levels, most RF interrelations and RF-distress pathways were stable between age 14 and 17, which may help explain why exposure to CA is frequently found to have a lasting impact on mental health. Thus, our findings not only shed light on the nature and changes of RFs between early and later adolescence, but also offer some accounts for why exposure to CA has strong proximal effects, and is often found to have a lasting impact on mental health.

Keywords: resilience factors, childhood adversity, mental health, adolescence

Abbreviations: RFs = Resilience factors; CA = Childhood adversity

Abbreviated title: Resilience Factor Changes between Early and Later Adolescence

Words: title page = 417 (abstract = 285), manuscript (including in-text references) = 6852, reference list = 1676, info sections = 219, tables = 1226

Unravelling the Complex Nature of Resilience Factors and their Changes between Early and Later Adolescence

Adolescents who have been exposed to adversity in childhood (CA), such as traumatic and/or severely stressful events, have a higher risk of developing mental health problems.[1–3] Moreover, approximately one in two children and adolescents worldwide experience adverse events before the age of 18.[1–4] Therefore, it is imperative that the deleterious mental health consequences following CA are addressed in research, therapy and mental health policy. This notion has not only been noticed in science,[3,5] but has also led to a discussion in public media questioning whether "…childhood trauma [should] be treated as a public health crisis?" (NPR: National Public Radio, 09 November 2018)[6] and whether "…people [can] be saved from a terrible childhood?" (The Guardian, 07 November 2018)[7]. One way to understand better how we can reduce the deleterious consequences of CA is to study the complex nature of resilience factors (RFs); i.e. factors that are empirically found to reduce the risk of mental health problems following CA.[8,9] To this end, we here aim to shed light on the nature of RFs during two time points, respectively marking early and late adolescence.

RFs operate on various intertwined functioning levels encompassing biological (e.g. genes or hormones), intra-personal (e.g. distress tolerance), and inter-personal levels (e.g. peer support).[8,10,11] We will focus on the latter two categories as those RFs can be targeted in psychosocial interventions and may therefore be particularly relevant in informing translational research and thus eventually prevention and therapy.

Despite the fact that RFs do not function in isolation, most studies have investigated single RFs.[8,12] Recently, researchers have argued that to improve our understanding of resilience mechanisms, it is necessary to move from relatively simple reductionist towards more holistic, complex models.[12–14] In several research fields, complex system models have been applied to describe risk and resilience processes, as for instance for financial markets or ecosystems.[13,15,16] Complex system models promise to fit the complexity of resilience research well, as they enable the exploration of multiple interconnected factors that are assumed to reinforce each other. Recently, we took the first step in bridging this gap for resilience research focussing on mental health in the face of adversity. We showed that RFs function as a complex interrelated network in both adolescents with and without CA, at age 14.[17] We found that the group of adolescents with CA had lower RF mean levels and the RFs were less positively interrelated, suggesting that the RFs may not enhance each other to the same extent as in adolescents without CA.[17]

Mental health levels can change over time, particularly during the process of dealing with adversity.[18–21] This suggests that RFs and/or their interrelations may also change over time. Individuals with CA often have

lower levels of RFs,[17,22] which are suggested to be transferred forward across development.[3,23] Hence, it is crucial to determine how RFs change over time in adolescents with and without CA, as this firstly unravels whether RFs change similarly or different in the two groups and secondly disentangles which RFs improve, deteriorate or stay stable during adolescence. Such RF changing patterns can inform translational research which in turn can shed light on the RFs that should be targeted and promoted to aid successful development after CA.[3,23] However, research on RF changes is surprisingly scarce, and results are mixed: Some intra- and inter-personal RFs are found to increase (e.g. ruminative worrying, prosocial involvement), whereas others have been reported to stay stable between early and later adolescence (e.g. family involvement, expressive suppression, dysfunctional rumination).[23–25] Here, we therefore examined whether RFs change between early (age 14) and later (age 17) adolescence, through investigating (a) RF mean levels, (b) RF interrelations, and (c) the way RFs are interrelated with distress (directly and/or indirectly via other RFs). Importantly, we specifically examined whether RFs change differentially in groups of adolescents with (CA+) and without CA (CA-).

Methods

Design

In 2005 and 2006, 1238 14-year-old adolescents were recruited from schools in Cambridgeshire to take part in the longitudinal Roots study. Follow-up took place around age 17.[26] Consent was provided by the adolescents and one parent.[26] Roots was conducted following Good Clinical Practice guidelines and the Declaration of Helsinki, and was approved by the Cambridgeshire Research Ethics Committee (03/302).[27]

Sample

In the current study we included 1139 of the 1238 participants, as they had data for potential CA experiences (CA+: n = 638; CA-: n = 501). There was on average 17.77% attrition (depending on the measure: 11.85 to 26.11%) between the two occasions.

Measures

Childhood adversity (CA). CA was assessed with the semi-structured Cambridge Early Experience Interview (CAMEEI) that mainly measures intra-family related adversity before the age of 14.[27] The interview was conducted with the primary caregiver, which was in 96% of the cases the biological mother. All interviews were performed when the adolescents were 14 years old. The CAMEEI was designed to measure adverse events in three time windows (0-5, 5-11, and 11-14 years), to support recall accuracy. Several types of adverse experiences were measured: loss of a family member, family separations (> 6 months), divorce, death, adoption, discord within the family, absence of maternal affection/involvement, aberrant parenting style, significant

medical illnesses within the family, psychopathology of family members, times of parental unemployment, financial hardship, physical abuse, sexual abuse, emotional abuse, criminality of family members, acute life events (e.g. environmental event with impact on the living situation), and chronic social hardship (e.g. demands of caring for extended family).[27] Based on this information Dunn and colleagues[27] performed a latent class analysis, which revealed four classes (no CA, moderate CA, severe CA, and aberrant parenting CA) for each of the three time windows. In line with previous reports[17] adolescents were assigned a '0' when they belonged for all three time windows to the 'no CA' category (CA-), and were assigned a '1' when they belonged for at least one time window to a category other than 'no CA' (CA+; see Table 1 for detailed numbers).

< Table 1 here>

General distress. To compile a general distress index, we used the 13-item short form of the Mood and Feelings Questionnaire (MFQ),[28] measuring a broad range of depression related symptoms, and the 28-item Revised Children's Manifest Anxiety Scale (RCMAS),[29] measuring a wide range of anxiety related symptoms. We used confirmatory factor analysis (CFA) based on polychoric correlations to estimate one underlying latent general distress factor for those 41 items. Brodbeck et al.[30], Stochl et al.[31] and St Clair et al.[32] used similar approaches and showed that latent general distress factors replicate well in adolescent samples. Please note the general distress factor does not match the one used in our previous report[17], for a detailed rationale see Supplement I.

Resilience factors (RFs). Based on findings of our preregistered systematic review,[8] we included 8 self-report (1-8 below) and 2 parent report RFs (9-10 below) that were assessed in our adolescent cohort. All RFs are scored in such a way that high values are protective, to which end five of the scales were reversed:

1. Friendship support was assessed with five items of the Cambridge Friendships Questionnaire.[33]
2. Family support was assessed with five items of the McMaster Family Assessment Device.[34]
3. Family cohesion was assessed with seven items of the McMaster Family Assessment Device.[34]
4. Positive self-esteem was assessed with five items of the Rosenberg self-esteem scale.[35]
5. Negative self-esteem was assessed with five items of the Rosenberg self-esteem scale.[35] We reversed the items so that high values of low negative self-esteem are protective.
6. Reflective rumination was assessed with five items of the Ruminative Response Scale (RRS).[36,37] We reversed the items so that high values of low reflective rumination are protective.

7. Ruminative brooding was assessed with five items of the RRS.[36,37] Please note the ruminative brooding factor does not match the one used in our previous report[17], for a detailed rationale see Supplement I. We reversed the items so that high values of low ruminative brooding are protective.
8. Aggression was assessed with four items of the Behaviour Checklist (11 questions based on the DSM-IV criteria for conduct problems).[38,39] We reversed the items so that high values of low aggression are protective.
9. Distress tolerance was assessed with five items of the Emotionality Activity Sociability Temperament Survey.[40]
10. Expressive suppression was assessed with one item of the Antisocial Process Screening Device.[41] We reversed the item so that high values of low expressive suppression are protective.

Information regarding the psychometric properties of the RF measures is reported in Fritz, Fried and colleagues[17] (i.e. in Supplement XIV).

Analysis

All analyses were conducted with R version 3.5.1.[42] All used packages and the belonging version numbers can be found in Supplement II.

Variable preparation. To estimate the best fitting latent RF and distress indices we used CFA models and extracted the resulting factor scores as RF and general distress variables. We decided to use factor scores instead of sum scores to reduce measurement error and to circumvent tau-equivalence (for a rational see Supplement III). As we aimed to compare two time points, we estimated longitudinal CFAs (LCFAs; separately for each RF and general distress). Given that all RF and general distress items were assessed with three to six answer categories, we computed categorical LCFAs,[43] treated the items as ordinal and used a weighted least square mean and variance adjusted (WLSMV) estimator (for details see Supplement III). Distribution plots for the RFs and general distress are in Supplement III.

Investigating RF mean level changes. To examine whether RFs (a) differ in their protective value between the CA+ and the CA- group, and (b) change in their protective value between age 14 and 17, we conducted RF mean comparison analyses. More specifically, we compared the RF and general distress mean levels (a) between the CA+ and the CA- group (i.e. separately for age 14 and 17), and (b) between age 14 and age 17 (i.e. separately in the CA+ and CA- groups). To ensure latent mean comparability across ages we estimated strongly invariant categorical LCFAs,[43] for which the exact LCFA parameter specifications and model identification details are outlined in Supplement III. All strongly invariant categorical LCFAs fitted

satisfactorily (Supplement III, Table 2). We did not compute an LCFA for the expressive suppression RF, as this RF was measured with only one item. We binarized the aggression and expressive suppression RFs, as they showed a restricted range. To circumvent slight deviations from normality we tested CA+ vs CA- mean level differences with independent sample Wilcoxon Rank Sum tests (with continuity correction). Moreover, we compared age 14 and age 17 mean levels with paired sample Wilcoxon Signed Rank tests (with continuity correction). As sensitivity analyses, we re-ran the mean change analyses for factor scores retrieved from the full invariance models, which can be found in Supplement IV. All mean comparisons were corrected for the false discovery rate.[44] Additionally, we explored whether CA moderates the relationship between age and RFs, to test whether the change patterns of the RFs differ between the two groups.

Investigating network structure changes. To examine (a) whether RFs interrelate differently in the CA+ and the CA- groups, and (b) whether those RF interrelations change between age 14 and 17, we computed RF network models. More specifically, we used RF factor scores to estimate regularized partial correlation network models.[45] Those models were computed separately for adolescents with and without CA, as well as for age 14 and age 17. We compared the resulting models with each other using permutation tests (i.e. network comparison tests (NCTs)).[46] To ensure that the exchangeability assumption of permutation tests was met (i.e. the joint distribution of the scores is invariant when permuting over time), we estimated fully invariant categorical LCFAs. The exact LCFA parameter specifications and details regarding the model identification can be found in Supplement III. All fully invariant categorical LCFAs fitted satisfactorily (see Supplement III, Table 2). As above, we did not compute an LCFA for expressive suppression, and we again binarized the aggression and expressive suppression RFs. We estimated (a) networks only containing the 10 RFs, (b) networks containing both the 10 RFs and the general distress variable, and (c) networks containing the 10 RFs corrected for general distress levels. In the results section we discuss the RF network models being corrected for general distress levels, as those enable the comparison of the CA+ and the CA- groups when taking the putatively confounding effect of psychopathology levels into account. To ensure conciseness, the other two models are discussed in Supplement VII.

For the comparisons of the four network models (i.e. CA+ vs CA- = independent sample permutation tests; and age 14 vs age 17 = paired sample permutation tests) we conducted three types of network comparison tests (two-tailed; we used an adjusted version of [46]). Firstly, we investigated whether the highest interrelation difference between the respective two networks differs from the highest interrelation differences of several (i.e. 5000 permutations) randomly permuted network model pairs, which indicates whether the two tested network

structures are invariant.[46] Secondly, we investigated whether the relative connectivity, which is the sum of the positive interrelations after subtracting the sum of the negative interrelations, differed between the two respective networks. This test is also called 'global network expected influence' comparison[17,47] and indicates to which degree RFs are concurrently positively associated. This test is of particular interest here, as it suggests to which degree RFs can concurrently enhance each other. Thirdly, we explored which individual RF interrelations and/or interrelations between RFs and general distress differed between the respective two networks of interest (for details see [46]). Hence, the first two tests examine *global* network structure differences, whereas the third test examines *local* network structure differences.

Investigating RF-general distress pathway changes. To examine the way RFs are interrelated with distress in the network models, we calculated two types of pathways between the RFs and general distress. First, we examined the direct pathways between the RFs and general distress, regardless of whether those pathways are the strongest or "quickest" ways to traverse the network from the RFs to general distress.[48] Second, we examined the shortest pathways (or 'shortest path lengths') between the RFs and general distress, regardless of whether the RFs have direct pathways with general distress. More specifically, we explored whether the shortest pathway to traverse the network from a given RF to the general distress variable is direct, or indirect via other RFs.[49] Moreover, we conducted permutation tests to compare the two types of pathways between the CA+ and the CA- group, for both age 14 and age 17. Lastly, we examined whether the two types of pathways changed between age 14 and 17 (i.e. separately for the CA+ and the CA- groups), again using permutation tests. Correlations and regularized partial correlations between the RFs and the general distress variable, for both CA+ and CA- as well as for age 14 and age 17, are discussed in Supplement IX.

Network stability, accuracy, and inference. To test the robustness of our network model parameters we estimated the stability of expected influence (EI) coefficients and the accuracy of all interrelations. We tested the stability of the EI coefficients by applying a subset bootstrap (2000 bootstraps) to identify the maximum sample percentage that can be dropped to reveal (with a 95% chance) a relationship of ≥ 0.7 between the subset and the original EI coefficients.[50] Moreover, we tested the accuracy of the network models by bootstrapping all interrelations (2000 bootstraps) and investigated their bootstrapped confidence intervals (CIs).[50] Those analyses are reported in Supplement X. We further explored the node expected influence coefficients for individual RFs (i.e. the sum of all positive interrelations of the respective RF, after subtracting the sum of the negative interrelations of that RF),[51,52] which are reported in Supplement XI.

Network sensitivity analyses. To establish whether our results would hold if the RFs were computed differently, we re-estimated the network models based on factor scores of the configural LCFAs, which do not constrain parameters across time points but estimate the best fitting time point specific latent factor. Results were overall comparable and are discussed in Supplement XIII.

Data Availability. Data for this specific paper has been uploaded to the Cambridge Data Repository https://doi.org/10.17863/CAM.36708 and is password protected. Our participants did not give informed consent for their measures to be made publicly available, and it is possible that they could be identified from this data set. Access to the data supporting the analyses presented in this paper will be made available to researchers with a reasonable request to openNSPN@medschl.cam.ac.uk.

Code Availability. Analysis code is available from http://jessica-fritz.com/.

Results

Sample

The CA+ (n = 638) and the CA- (n = 501) groups did not differ with regard to age or gender, but the CA+ group had a lower socio-economic status (see Table 2). In addition, adolescents in the CA+ group were more likely to have a psychiatric history, and had higher levels of depression and anxiety symptoms, at both age 14 and 17.

<Table 2 here>

RF Mean Level Changes

Group Comparisons. At both age 14 and 17, distress was significantly higher and six of the ten RFs (i.e. family support, family cohesion, negative self-esteem, positive self-esteem, ruminative brooding, and distress tolerance) were significantly lower in the CA+ group (please note, RFs are scored in such a way that high levels are protective; see Table 3). Moreover, the CA+ group had lower levels of expressive suppression and aggression at age 14, and lower levels of friendship support at age 17. The general pattern indicates that RFs are lower and distress is higher in the CA+ than in the CA- group, during both early and later adolescence.

<Table 3 here>

Temporal Comparisons. In both groups, three RFs had higher mean levels at age 17 than at age 14: negative self-esteem, distress tolerance, and aggression potential. In contrast, in both groups, two RFs had lower mean levels at age 17 than at age 14: ruminative brooding and reflection. Five did not change across time (see Figure 1). Importantly, age-CA interaction effects did neither predict the RFs, nor general distress (see Table 4). Therefore, all RFs that changed between age 14 and 17 did so in similar ways in the two groups.

<Table 4 here>

<Figure 1 here>

RF Interrelation Changes

Group Comparisons. Figure 2 depicts the RF networks that are corrected for general distress for the CA+ and the CA- group, as well as for age 14 and 17. For age 14, the CA+ and CA- networks were invariant (M = .16, p = .47). However, the global network expected influence, which indicates the degree to which RFs are positively interrelated, was significantly lower in the CA+ network (EI_{CA+} = 2.13, EI_{CA-} = 2.66, EI = 0.53, p < .05). This suggests that in the CA+ network RFs are less likely to enhance each other than in the CA- network. One individual RF interrelations differed between the CA+ and the CA- networks (see Table 4 in Supplement VI). For age 17, both the global network structure invariance and the expected influence comparison tests were not significant (M = .18, p = .70; EI_{CA+} = 2.18, EI_{CA-} = 2.32, EI = 0.14, p = .70). Moreover, two individual RF interrelation differed between the CA+ and the CA- networks (see Table 4 in Supplement VI).

Temporal Comparisons. When we compared the networks between age 14 and 17, the networks were invariant and did not differ in global network expected influence, in both the CA+ (M = .16, p = .50; EI_{14} = 2.14, EI_{17} = 2.18, EI = 0.04, p = .88) and the CA- group (M = .23, p = .20; EI_{14} = 2.66, EI_{17} = 2.32, EI = 0.34, p = .22). In the CA+ network two individual RF interrelations changed significantly between age 14 and 17, and one changed in the CA- network, see Table 5 in Supplement VI.

<Figure 2 here>

Changes in Pathways between RFs and General Distress

Group Comparisons. First, we explored the *direct pathways* between the RFs and general distress (Figure 3 upper panel). At age 14, most RFs had negative direct pathways, in both the CA+ and the CA- group, indicating that high RFs go together with low distress (or vice versa). Those *negative direct pathways* to distress were stronger in the CA+ than in the CA- group (DP_{CA+} = - 1.50, DP_{CA-} = -1.20, DP = 0.30, p = .05; i.e. a more negative DP value indicates a stronger (negative) direct pathway and a less negative DP value indicates a weaker (negative) direct pathway). At age 17, results were similar, but the strength of the *direct pathways* no longer differed significantly between the two groups (DP_{CA+} = -1.52, DP_{CA-} = -1.20, DP = 0.31, p = .11). Importantly, the *direct pathway* results do not consider that some RFs have stronger *indirect* than *direct* effects on distress, i.e. via other RFs. To this end, we next calculated *shortest pathways* between RFs and distress, which indicate the quickest way to traverse the network from the RF to distress (Figure 3 lower panel). At age 14, most RFs in the CA+ group had a *direct shortest pathway* with general distress, whereas most RFs in the

CA- group had an *indirect shortest pathway* with distress. However, the overall strength of the *shortest pathways* did not differ significantly between the two groups (SP_{CA+} = 67.21, SP_{CA-} = 77.12, SP = 9.90, p = .46; i.e. a lower SP value indicates a stronger (and thus shorter) shortest pathway and a higher SP value indicates a weaker (and thus longer) shortest pathway). At age 17, the two groups did again differ in the number of *negative shortest pathways* (five in the CA+ compared to four in the CA- group), but not in the strength of the *shortest pathways* (SP_{CA+} = 82.15, SP_{CA-} = 102.16, SP = 20.02, p = .20).

Temporal Comparisons. When comparing the *direct pathways* between the RFs and general distress between age 14 and age 17, no significant temporal differences were found in the CA+ (CA+: DP_{14} = -1.50, DP_{17} = -1.52, DP = 0.01, p = 0.95) and the CA- group (DP_{14} = -1.20, DP_{17} = -1.20, DP = 0.00, p = 0.99). Moreover, the *shortest pathways* did neither in the CA+ (SP_{14} = 67.21, SP_{17} = 82.15, SP = 14.94, p = 0.18) nor in the CA- group (SP_{14} = 77.12, SP_{17} = 102.16, SP = 25.05, p = 0.10) differ in strength between age 14 and age 17.

<Figure 3 here>

Discussion

We aimed to shed light on RF changes between age 14 and age 17, and investigated (a) RF mean levels, (b) RF interrelations, and (c) pathways from the RFs to general distress, in adolescents with and without CA. Regarding RF mean levels (a) we found that although inter-personal RFs (e.g. friendship support) seemed to stay stable, most intra-personal RFs (e.g. distress tolerance) changed between age 14 and 17. Interestingly, all RFs that in- or decreased between age 14 and 17 changed similarly in the two groups. Moreover, the CA+ group had lower RFs and higher distress at both ages. Regarding RF interrelations (b) we found that at age 14, but not at age 17, RFs were less positively interrelated. This suggests that the RFs are less likely to enhance each other in the CA+ compared to the CA- network, when the general distress level is taken into account. Regarding RF-distress pathways (c) our results suggest that at age 14 RFs had stronger negative *direct* pathways with distress in the CA+ than in the CA- group, while the CA- group reported lower mean levels of distress and higher mean levels of RFs. Despite that the same mean level pattern was observed at age 17, the *direct* RF-distress pathways were similarly strong in the CA+ and the CA- group at age 17. The strength of the *shortest* pathways did not differ between the CA+ and the CA- group, neither at age 14 nor at age 17. Below we will outline how our findings inform about the complex nature of RFs and will discuss tentative accounts for why CA+ not only has strong proximal effects, but is often found to have a lasting impact on mental health.

RF Mean Level Changes

All inter-personal RFs (i.e. friendship support, family support, and family cohesion) seemed to stay stable between age 14 and 17, showing that, in this cohort, adolescents perceive their social support environment to be similar during early and later adolescence. Importantly, we found that the mean levels of most intra-personal RFs changed between age 14 and 17 (i.e. distress tolerance, aggression, negative self-esteem, brooding, and reflection). Adolescents reported a higher level of distress tolerance and a lower aggression potential at age 17 than at age 14, which potentially may be explained by the improvement of executive functions and emotion regulation strategies. Previous literature has shown that executive functions, such as inhibitory control which facilitates the regulation of cognition and behaviour, develop and improve until adulthood.[53,54] Similarly, the use of emotion regulation strategies is found to be significantly lower in mid-adolescence (age 15) than in young adulthood (age 19).[25]

In the literature, findings regarding changes in rumination are mixed. For example, Zimmerman and Iwanski[25] did not find a significant difference in rumination between age 13 and 17, whereas Frydenberg and Lewis[24] showed that ruminative worrying is higher at age 16 than at age 14. In line with Frydenberg and Lewis,[24] our sample reported higher (more harmful) levels of reflective rumination and ruminative brooding at age 17 than at age 14. Despite the increase in rumination, our adolescents reported a decrease in negative self-esteem between age 14 and 17. Those results together suggest that although adolescents may worry and reflect more about their experiences and behaviours during later adolescence, they may not attach those negative thoughts and evaluations to their self-image. While further replication of our results is required, we suggest that between early and later adolescence mechanisms emerge that alter the perception of the self (e.g. negative self-esteem, rumination) and self-regulation (e.g. distress tolerance, aggression).[23–25,53,54]

Our results further showed that all changes in RF mean levels between early and later adolescence were similar in the CA+ and the CA- groups. Crucially, however, the CA+ group had lower RFs at both ages, which is in line with previous research.[22] Hence, CA does not seem to inhibit RF changes, but seems to increase the risk of persistently lower RFs. Those findings support the hypothesis that lower and therefore possibly disadvantageous RF levels after CA are transferred forward from early to later adolescence,[3,23] which underpins the importance of revealing which factors and processes lend themselves best to aid optimal development after CA.[3,23]

In sum, our findings show that individual RFs change differently between early and later adolescence, but that the change pattern is similar in groups of CA+ and CA- adolescents. Based on those results we cautiously suggest implications for future research, while reminding the reader that our findings only allow for

group level not individual level conclusions. The main questions that arise from our mean level findings are threefold. Firstly, one could ask whether RFs that seem to increase naturally during adolescence (i.e. distress tolerance, self-esteem, low aggression potential) are particularly amenable and therefore more efficient intervention targets for reducing distress. Similarly, one may wonder whether it may be as advantageous to intervene on worsening RFs (i.e. ruminative reflection and brooding), to reduce or prevent such a decline. Regarding RFs that stay stable (i.e. friendships, family support and family cohesion), the arising question seems different. Stable RF levels may be advantageous for adolescents with a high level of those RFs, but may be disadvantageous for adolescents with a persistently low level of those RFs. Speculatively, stable RFs may function as a "vulnerability marker" when being persistently low, and an early detection may be beneficial. Replication studies and translational research are crucially needed to answer these important questions, as such knowledge may eventually shed light on which RFs should be targeted in order to aid successful mental health development in adolescents with and without CA.

RF Interrelation Changes

Despite the fact that the RF levels differed between the CA+ and the CA- group at both age 14 and 17, RF interrelations differed between the two groups only at age 14, not at age 17. This suggests that CA may have a more pronounced effect at age 14, as it then goes together with both differential RF levels and differential RF interrelations. One account could be proximity of CA, as CA was measured up to the age of 14. This would be in line with previous work suggesting that although CA has deleterious effects on mental health across the life course, it has a particularly strong effect on a shorter term and accordingly a decreasing effect on affective and behaviour disorders from childhood to young adulthood.[2,55]

Interestingly, on a *global* network structure level, taking the overall pattern of RF interrelations into account, both the CA+ and the CA- network were invariant between early and later adolescence. Moreover, neither the CA+ nor the CA- network changed in the degree to which RFs are expected to enhance each other (i.e. expected influence) between early and later adolescence. We believe that the lack of temporal changes on the *global* network level is unlikely to be explained by power, as we did detect a difference in expected influence between the CA+ and the CA- networks at age 14. Moreover, on the *local* network structure level, we also identified only minor changes between early and later adolescence. In the CA+ network one out of 45 possible RF interrelations turned more positive and one turned less positive between age 14 and 17 (see Table 5 in Supplement VI), which may have cancelled each other out and may thus explain why there was hardly any change in the expected influence of the CA+ network. In the CA- network one RF interrelation became

significantly more negative between age 14 and 17 (see Table 5 in Supplement VI), which may have contributed to the small and non-significant decline in the expected influence of the CA- network. Hence, those findings indicate a general stability of RF interrelations between early and later adolescence, in both the CA+ and the CA- network. If this would generalize to other cohorts, it may offer one account for the finding that CA often has lasting effects on mental health.[1,56]

For both the CA+ and the CA- network, at both age 14 and age 17, the family, brooding and negative self-esteem RFs were most positively connected with the other RFs (for more details see Supplement XI). Hence, those RFs are potentially important in driving the positive connectivity of the RF networks and in underpinning the degree to which RFs can enhance each other. Interestingly, in terms of mean levels the family RFs stayed stable, the brooding RF decreased and the negative self-esteem RF increased between age 14 and age 17. This suggests that (changes in) mean levels of RFs may not, or at least not directly, impact the degree to which the RFs can enhance other RFs. Thus, our RF mean level and RF network model analyses provide independent but complementary insights. To further improve knowledge about the clinical relevance of those indicators, future research needs to examine whether RF mean levels or RF interrelations characteristics (such as *expected influence* coefficients) are better predictors for subsequent mental health. Such knowledge needs to be obtained before our network findings can inform clinical research, as knowledge on the prediction magnitude is essential for picking promising RF targets for translational studies.

Changes in Pathways between RFs and General Distress

Our findings showed that most RFs had direct negative pathways with distress, in both the CA+ and the CA- group, indicating that high RFs decrease distress, high distress decreases RFs, or both mutually influence each other. As all investigated RFs have empirically been shown to significantly decrease subsequent distress,[8] it seems plausible that RF-distress pathways may not only over time, but also concurrently operate as protective pathways. In the same vein it is however also plausible that high distress reduces the protective effects of RFs (concurrently and/or over time). Such mutualistic coupling effects[57] need to be examined in future research. At age 14, *direct* RF-distress pathways appeared to be stronger in the CA+ than in the CA- group. We did not detect differences between age 14 and 17, suggesting that *direct* RF-distress pathways seem stable between age 14 and 17. With the additional *shortest* pathway analyses we aimed to detect the quickest way to traverse the network from an RF to distress regardless of whether the pathway is *direct* or *indirect* (i.e. via other RFs). At age 14, six of the ten RFs had a *direct* negative shortest pathway in the CA+ group and four out of 10 RFs had a *direct* negative shortest pathways with distress in the CA- group. Yet, the strength of the shortest pathways did

not differ between the two groups. Moreover, we did not detect significant changes in the strength of the shortest pathways between age 14 and 17.

On the first glance, the *direct* pathway analyses for age 14 seem to suggest that *direct* pathways between RFs and distress may be more protective in the CA+ than in the CA- group. Importantly however, when taking our mean level findings into account ─ i.e. that the CA+ group had lower RFs and higher distress than the CA- group ─ another interpretation emerges. That is, despite the fact that at age 14 the RFs appear to reduce distress more in the CA+ group, they did not succeed in reducing distress to a similar mean level as in the CA- group. Therefore, the lower RF levels in the CA+ group could be seen as insufficient to decrease distress to a similar mean level as in the CA- group. Moreover, there was no difference in strength of the *shortest* pathways between the CA+ and the CA- group, neither at age 14 nor at age 17, which additionally underpins that overall RF-distress pathways may be similarly protective in the two groups. In sum, as lower RFs, higher distress, the RF interrelation pattern, and the strength of RF-distress pathways seemed to be persistent from early to later adolescence, this may help explain why exposure to CA is frequently found to not only have a short-term, but also a longer lasting impact on mental health.[1,56]

The three RFs that were most strongly interrelated with distress, in both the direct and the shortest pathway models, were negative self-esteem, brooding and aggression. Interestingly, negative self-esteem and brooding were also among those RFs that were most positively connected with the other RFs, in both groups and at both ages. Hence, if replication of our findings would hold, the negative self-esteem and brooding RFs may be of particular interest for future prediction studies, as they not only seem to have the highest potential of increasing other RFs, but also seem to have the highest potential in reducing distress, and therefore may also have a high potential in reducing subsequent mental health problems.

Limitations

Our research has several limitations. First, CA was assessed with retrospective caregiver report, which may be inaccurate due to for example limited recall, limited knowledge, or embarrassment. To enhance recall caregivers were encouraged to use assisting material (e.g. photo albums),[27] and an event timeline (with the following time windows: 0-5, 5-11, 11-14) was established. Second, the family support and family cohesion RFs were derived from one questionnaire, which may have resulted in more similar response patterns in those RFs. The same argument goes for rumination (reflection and brooding) and self-esteem (high positive and low negative self-esteem) RFs. Third, to enable RF comparisons over time, we had to equate multiple LCFA parameters between age 14 and age 17. This may disadvantage the model fit and therefore potentially increase

bias in the resulting factor scores. To circumvent this limitation as best we could, we used the least restricted models possible to still meet the assumptions of the respective network and mean change analyses. However, this meant that we could not use the exact same factor scores for the network and the mean change analyses. For completeness, we re-ran the mean change analyses with factor scores derived from the LCFAs that we used for the network analyses, which revealed similar findings (see Supplement IV). Fourth, we interpret negative interrelations between RFs in networks that take general distress into account as disadvantageous. However, as our models are undirected, we cannot disentangle whether the general distress variable behaved as intended as a confounder, or against our expectation as a collider,[58] falsely inducing or enhancing these interrelations (for a detailed discussion see Supplement XII in [17]). Fifth, we performed the network models with regularized partial correlations, which currently is the default method. However, recently, other approaches have been suggested such as non-regularized methods.[59] Future research will need to show which methods tend to be most suitable for psychometric network models. Sixth, as our study contains two time points, we cannot draw conclusions with regard to tipping points or specifically sensitive periods. Likewise, we cannot examine how RFs change from prior to post CA, as we did not assess the RFs prior to CA. Seventh, we only included information for participants who took part at both time points, which reduced the available information of the baseline sample by 17.77 percent (range = 11.85 to 26.11%). Considering the size of the cohort (N = 1139) and its longitudinal nature we believe that this amount of information is still highly insightful, but that does not preclude a potential increase in selection bias. Eighth, it would have been valuable to explore gender effects (e.g. as in [60]), however, for many of the analyses we would not have had enough power to split the sample additionally with regard to gender. Ninth, the Roots participants had on average a slightly higher SES than the average UK population and generalizations may therefore be most valid for above average SES populations.[26]

Regarding the question whether resilience and risk factors are opposing sides of the same coin, the quick, but insufficient answer for our study is probably that many (or most) of the investigated RFs are indeed the flip side of risk factors. For example, self-esteem (or a positive self-concept) is commonly defined as RF and has been discussed as such by many of the seminal resilience researchers, including Michael Rutter, Emmy Werner, Ann Masten, and Michael Ungar (for a review see e.g.[61]). Yet, at the same time a low level of self-esteem or self-worth is part of the DSM V criteria for depression (“Feelings of worthlessness”; American Psychiatric Association[62]). Hence, whereas a high level of self-esteem may protect against low mood levels, low self-esteem is assumed to contribute to or reflect low mood. As doing this question fully justice is out of the scope of this discussion, we added a more detailed debate on the question to Supplement XIV. Importantly

however, regardless of whether resilience and risk factors operate on the same continuum or are inversely correlated but not identical, understanding the nature of RFs seems to have universal appeal as it focuses on what promotes good mental health rather than on what increases mental health problems.

Conclusion

Our results support several prior conjectures regarding changes in RF mean levels, for example that lower and therefore disadvantageous levels of RFs are likely to be carried forward over time in adolescents with prior exposure to CA. Our findings also contribute novel hypotheses: for example, they suggest that RF changes are similar in adolescents with and without CA, and that inter-personal mean levels may stay stable, whereas many intra-personal RFs change between early and later adolescence. On a network level CA seemed to have a stronger proximal effect, as RF interrelations differed between the two groups at age 14, but not at age 17. Despite RFs seeming to have stronger direct protective effects in the CA+ group, at least at age 14, the CA+ group reported more distress than the CA- group at both age 14 and 17. We cautiously suggest that lower RFs in the CA+ group are insufficient to decrease distress to a similar mean level as in the CA- group. Moreover, after considering that RFs also can have indirect pathways with distress, we no longer detected a difference in the potentially protective pathways between the two groups. As lower RFs, higher distress, RF-RF interrelations, and RF-distress pathways seemed to be carried forward from early to later adolescence, our findings may help explain why exposure to CA is frequently found to have a lasting impact on mental health. To pinpoint the clinical relevance of our findings, we commend future research to examine whether (a) RF mean levels, (b) RF interrelations coefficients, or (c) RFs that score high on both indicators offer the best prediction for subsequent mental health and thus lend themselves best for formulating translational hypotheses. In sum, our study not only sheds light on the complex nature and changes of ten empirically supported RFs between early and later adolescence, but also offers tentative accounts for why CA+ has strong proximal effects, and is often found to have a lasting impact on mental health.

References

1. Greif Green, J. *et al.* Childhood adversities and adult psychopathology in the National Comorbidity Survey Replication (NCS-R) I: Associations with first onset of DSM-IV disorders. *Arch. Gen. Psychiatry* **67,** 113–133 (2010).

2. Kessler, R. C. *et al.* Childhood adversities and adult psychopathology in the WHO World Mental Health Surveys. *Br. J. Psychiatry* **197,** 378–385 (2010).

3. McLaughlin, K. A. Future Directions in Childhood Adversity and Youth Psychopathology. *J. Clin. Child Adolesc. Psychol.* **45,** 361–382 (2016).

4. Kessler, R. C., Davis, C. G. & Kendler, K. S. Childhood adversity and adult psychiatric disorder in the US National Comorbidity Survey. *Psychol. Med.* **27,** 1101–1119 (1997).

5. Afifi, T. O. *et al.* Individual- and Relationship-Level Factors Related to Better Mental Health Outcomes following Child Abuse: Results from a Nationally Representative Canadian Sample. *Can. J. Psychiatry* **61,** 776–788 (2016).

6. Blakemore, E. Should Childhood Trauma Be Treated As A Public Health Crisis? (2018). Available at: https://www.npr.org/sections/health-shots/2018/11/09/666143092/should-childhood-trauma-be-treated-as-a-public-health-crisis. (Accessed: 9th November 2018)

7. Zanolli, L. Can people be saved from a terrible childhood? (2018). Available at: https://www.theguardian.com/world/commentisfree/2018/nov/07/ace-adverse-childhood-experience-trauma. (Accessed: 7th November 2018)

8. Fritz, J., de Graaff, A. M., Caisley, H., van Harmelen, A.-L. & Wilkinson, P. O. A Systematic Review of Amenable Resilience Factors that Moderate and/or Mediate the Relationship between Childhood Adversity and Mental Health in Young People. *Front. Psychiatry* **9,** 230 (2018).

9. Zimmerman, M. A. *et al.* Adolescent Resilience: Promotive Factors That Inform Prevention. *Child Dev. Perspect.* **7,** 215–220 (2013).

10. Ioannidis, K., Askelund, A. D. & van Harmelen, A.-L. The complex neurobiology of resilient functioning after child maltreatment. *Open Sci. Framew.* Retrieved from https://osf.io/3vfqb/ (2018).

11. van Harmelen, A.-L. *et al.* Friendships and Family Support Reduce Subsequent Depressive Symptoms

in At-Risk Adolescents. *PLoS One* **11,** e0153715 (2016).

12. Diehl, M., Hay, E. L. & Chui, H. Personal Risk and Resilience Factors in the Context of Daily Stress. *Annu Rev Gerontol Geriatr.* **32,** 251–274 (2012).

13. Scheffer, M. *et al.* Quantifying resilience of humans and other animals. *PNAS* **115,** 11883–11890 (2018).

14. Kalisch, R. *et al. Deconstructing and reconstructing resilience: a dynamic network approach*. (Manuscript submitted for publication, 2018).

15. Scheffer, M. *et al.* Creating a safe operating space for iconic ecosystems. *Science (80-.).* **347,** 1317–1318 (2015).

16. Battiston, S. *et al.* Complexity theory and financial regulation. *Science (80-.).* **351,** 818–820 (2016).

17. Fritz, J., Fried, E. I., Goodyer, I. M., Wilkinson, P. O. & van Harmelen, A.-L. A Network Model of Resilience Factors for Adolescents with and without Exposure to Childhood Adversity. *Sci. Rep.* **8,** 15774 (2018).

18. Costello, E. J., Copeland, W. & Angold, A. Trends in psychopathology across the adolescent years: What changes when children become adolescents, and when adolescents become adults? *J. Child Psychol. Psychiatry Allied Discip.* **52,** 1015–1025 (2011).

19. Kalisch, R. *et al.* The resilience framework as a strategy to combat stress-related disorders. *Nat. Hum. Behav.* (2017). doi:10.1038/s41562-017-0200-8

20. Rutter, M. Resilience in the face of adversity: Protective factors and resistance to psychiatric disorder. *Br. J. Psychiatry* **147,** 598–611 (1985).

21. Masten, A. S. Resilience in children threatened by extreme adversity: Frameworks for research, practice, and translational synergy. *Dev. Psychopathol.* **23,** 493–506 (2011).

22. Almquist, Y. B. *et al.* Prevailing over adversity: Factors counteracting the long-term negative health influences of social and material disadvantages in youth. *Int. J. Environ. Res. Public Health* **15,** (2018).

23. Kim, B. K. E., Oesterle, S., Catalano, R. F. & Hawkins, J. D. Change in protective factors across adolescent development. *J. Appl. Dev. Psychol.* **40,** 26–37 (2015).

24. Frydenberg, E. & Lewis, R. Teaching Coping to Adolescents: When and to Whom? *Am. Educ. Res. J.* **37,** 727–745 (2000).

25. Zimmermann, P. & Iwanski, A. Emotion regulation from early adolescence to emerging adulthood and middle adulthood: Age differences, gender differences, and emotion-specific developmental variations. *Int. J. Behav. Dev.* **38,** 182–194 (2014).

26. Goodyer, I. M., Croudace, T., Dunn, V., Herbert, J. & Jones, P. B. Cohort Profile: Risk patterns and processes for psychopathology emerging during adolescence: the ROOTS project. *Int. J. Epidemiol.* **39,** 361–369 (2010).

27. Dunn, V. J. *et al.* Profiles of family-focused adverse experiences through childhood and early adolescence: The ROOTS project a community investigation of adolescent mental health. *BMC Psychiatry* **11,** 109 (2011).

28. Messer, S. C., Angold, A. & Costello, E. J. Development of a Short Questionnaire for Use in Epidemiological Studies of Depression in Children and Adolescents: Factor Composition and Structure across Development. *Int. J. Methods Psychiatr. Res.* **5,** 251–262 (1995).

29. Reynolds, C. R. & Richmond, B. O. What I Think and Feel: A Revised Measure of Children's Manifest Anxiety. *J. Abnorm. Child Psychol.* **6,** 271–280 (1978).

30. Brodbeck, J., Abbott, R. A., Goodyer, I. M. & Croudace, T. J. General and specific components of depression and anxiety in an adolescent population. *BMC Psychiatry* **11,** 191 (2011).

31. Stochl, J. *et al.* Mood, anxiety and psychotic phenomena measure a common psychopathological factor. *Psychol. Med.* **45,** 1483–1493 (2015).

32. St Clair, M. C. *et al.* Characterising the latent structure and organisation of self-reported thoughts, feelings and behaviours in adolescents and young adults. *PLoS One* **12,** e0175381 (2017).

33. Goodyer, I. M., Wright, C. & Altham, P. M. E. Recent friendships in anxious and depressed school age children. *Psychol. Med.* **19,** 165–174 (1989).

34. Epstein, N. B., Baldwin, L. M. & Bishop, D. S. The McMaster Family Assessment Device. *J. Marital Fam. Ther.* **9,** 171–180 (1983).

35. Rosenberg, M. *Society and the Adolescent Self-Image*. (Princeton, NJ: Princeton University Press,

1965).

36. Treynor, W., Gonzalez, R. & Nolen-Hoeksema, S. Rumination reconsidered: A psychometric analysis. *Cognit. Ther. Res.* **27,** 247–259 (2003).

37. Burwell, R. A. & Shirk, S. R. Subtypes of rumination in adolescence: Associations between brooding, reflection, depressive symptoms, and coping. *J. Clin. Child Adolesc. Psychol.* **36,** 56–65 (2007).

38. Goodyer, I. M. *et al.* Improving mood with psychoanalytic and cognitive therapies (IMPACT): a pragmatic effectiveness superiority trial to investigate whether specialised psychological treatment reduces the risk for relapse in adolescents with moderate to severe unipolar dep... *Trials* **12,** 175 (2011).

39. American Psychiatric Association. *Diagnostic and statistical manual of mental disorders (4th ed., text rev.)*. (Washington, DC: Author, 2000).

40. Bould, H., Joinson, C., Sterne, J. & Araya, R. The Emotionality Activity Sociability Temperament Survey: Factor analysis and temporal stability in a longitudinal cohort. *Pers. Individ. Dif.* **54,** 628–633 (2013).

41. Poythress, N. G. *et al.* Internal Consistency Reliability of the Self-Report Antisocial Process Screening Device. *Assessment* **13,** 107–113 (2006).

42. R Core Team. R: A language and environment for statistical computing. (2018).

43. Wu, H. & Estabrook, R. Identification of Confirmatory Factor Analysis Models of Different Levels of Invariance for Ordered Categorical Outcomes. *Psychometrika* **81,** 1014–1045 (2016).

44. Benjamini, Y. & Hochberg, Y. Controlling the false discovery rate: a practical and powerful approach to multiple testing. *J. R. Stat. Soc. Ser. B* **57,** 289–300 (1995).

45. Epskamp, S. & Fried, E. I. A Tutorial on Regularized Partial Correlation Networks. *Psychol. Methods* **23,** 617–634 (2018).

46. van Borkulo, C. D. *Comparing network structures on three aspects: A permutation test (PhD Thesis Chapter 5)*. (University of Groningen, 2018).

47. Elliott, H., Jones, P. J. & Schmidt, U. Central Symptoms Predict Post-Treatment Outcomes and Clinical Impairment in Anorexia Nervosa: A Network Analysis. *PsyArXiv* (2018).

48. Isvoranu, A.-M. *et al.* Toward incorporating genetic risk scores into symptom networks of psychosis. *Psychol. Med.* 1–8. https:// org/10.1017/S003329171900045X (2019).

49. Isvoranu, A.-M., Borsboom, D., van Os, J. & Guloksuz, S. A Network Approach to Environmental Impact in Psychotic Disorder: Brief Theoretical Framework. *Schizophr. Bull.* **42,** 870–873 (2016).

50. Epskamp, S., Borsboom, D. & Fried, E. I. Estimating Psychological Networks and their Accuracy : A Tutorial Paper. *Behav. Res. Methods* **50,** 195–212 (2018).

51. Costantini, G. *et al.* State of the aRt personality research: A tutorial on network analysis of personality data in R. *J. Res. Pers.* **54,** 13–29 (2015).

52. McNally, R. J. Can network analysis transform psychopathology? *Behav. Res. Ther.* **86,** 95–104 (2016).

53. Friedman, N. P. *et al.* Stability and change in executive function abilities from late adolescence to early adulthood: A longitudinal twin study. *Dev. Psychol.* **52,** 326–340 (2016).

54. Dimond, A. Executive Functions. *Annu. Rev. Psychol.* **64,** 135–168 (2013).

55. Shanahan, L., Copeland, W. E., Costello, E. J. & Angold, A. Child-, adolescent-and young adult-onset depressions: Differential risk factors in development? *Psychol. Med.* **41,** 2265–2274 (2011).

56. Raposo, S. M., Mackenzie, C. S., Henriksen, C. A. & Afifi, T. O. Time Does Not Heal All Wounds: Older Adults Who Experienced Childhood Adversities Have Higher Odds of Mood, Anxiety, and Personality Disorders. *Am. J. Geriatr. Psychiatry* **22,** 1241–50 (2014).

57. Kievit, R. A. *et al.* Mutualistic Coupling Between Vocabulary and Reasoning Supports Cognitive Development During Late Adolescence and Early Adulthood. *Psychol. Sci.* **28,** 1419–1431 (2017).

58. Elwert, F. & Winship, C. Endogenous Selection Bias: The Problem of Conditioning on a Collider Variable. *Annu. Rev. Sociol.* **40,** 31–53 (2014).

59. Williams, D. R., Rhemtulla, M., Wysocki, A. & Rast, P. *On Non-Regularized Estimation of Psychological Networks*. *PsyArXiv* (Retrieved from: https://doi.org/10.31234/osf.io/xr2vf/, 2018).

60. Stochl, J. *et al.* Identifying key targets for interventions to improve psychological wellbeing: Replicable results from four UK cohorts. *Psychol. Med.* 1–8 (2018). doi:10.1017/S0033291718003288

61. VicHealth. *Current theories relating to resilience and young people: A literature review. Victorian*

Health Promotion Foundation: Melbourne, Australia (2015).

62. American Psychiatric Association. *Diagnostic and statistical manual of mental disorders (5th ed.).* (Washington, DC: Author, 2013).

63. Hothorn, T., Hornik, K., van de Wiel, M. A. & Zeileis, A. Implementing a Class of Permutation Tests: The coin Package. *J. Stat. Softw.* **28,** 1–23 (2008).

64. Morgan, M. & Chinn, S. ACORN group, social class, and child health. *J. Epidemiol. Community Health* **37,** 196–203 (1983).

65. Kaufman, J. *et al.* Schedule for Affective Disorders and Schizophrenia for School-Age Children-Present and Lifetime Version (K-SADS-PL): Initial Reliability and Validity Data. *J. Am. Acad. Child Adolesc. Psychiatry* **36,** 980–988 (1997).

Acknowledgements

JS received support from the NIHR Collaboration for Leadership in Applied Health Research and Care (CLAHRC) East of England (EoE) at the Cambridgeshire and Peterborough NHS Foundation Trust. IMG is funded by a Wellcome Trust Strategic Award and declares consulting to Lundbeck. CDvB is funded by the ERC Consolidator Grant (647209). POW is funded by the University of Cambridge. ALvH is funded by the Royal Society (DH15017 & RGF\EA\180029 & RFG/RI/180064), and MQ (MQBFC/2). JF is funded by the Medical Research Council Doctoral Training/Sackler Fund and the Pinsent Darwin Fund. The views expressed are those of the authors and not necessarily those of the NHS, the NIHR or the Department of Health and Social Care. Funders of the authors played no role in the study conduction, analysis performance, or the reporting of the study.

Author Contributions

IMG was responsible for the data collection. JF formulated the research proposal in collaboration with JS, IMG, ALvH, and PoW. JF performed the analyses and the write up in collaboration with JS, EIF, IMG, CDvB, POW and ALvH. All authors approved the final manuscript. POW and ALvH are joint last authors of this manuscript.

Competing Interests

The authors declare no competing interests.

Correspondence

Jessica Fritz, University of Cambridge, Department of Psychiatry, Douglas House, 18B Trumpington Road, phone: +44 (0)1223 465253, email: jf585@cam.ac.uk

Table 1

Numbers CA exposure

0 to 5 years	5 to 11 years	11 to 14 years	CA variable	Cumulative number of participants with CA		
CA+ = 355	CA+ = 463	CA+ = 406	CA+ = 638	1 time window	2 time windows	3 time windows
CA- = 784	CA- = 676	CA- = 733	CA- = 501	n = 262	n = 166	n = 210

Table 2

Sample Comparisons: CA+ (n = 638) versus CA- (n = 501) Groups

	CA+	CA-	$t^{*1}/z^{*2}/X^{2*3}$ (*DF*)	95% CI[*4]	*p*
gender	*n* girls = 358	*n* girls = 262	1.50(1)		.22
	n boys = 280	*n* boys = 239			
SES[*5]	*n* hard pressed = 77	*n* hard pressed = 30	5.45		<.001
	n moderate means = 36	*n* moderate means = 11			
	n comfortably off = 170	*n* comfortably off = 105			
	n urban prosperity = 37	*n* urban prosperity = 41			
	n wealthy achievers = 318	*n* wealthy achievers = 314			
Age 14					
age	*M* = 14.49, *SD* = 0.28	*M* = 14.48, *SD* = 0.28	-0.43(1049.3)	-.04 - .03	.67
psychiatric history	*n* PH = 201	*n* PH = 74	42(1)		<.001
(PH)[*6]	*n* no-PH = 437	*n* no-PH = 427			
depression symptoms	*M* = 17.42, *SD* = 11.61	*M* = 14.03, *SD* = 10.46	-5.10(1088.5)	-4.69 - -2.09	<.001
anxiety symptoms	*M* = 16.92, *SD* = 12.61	*M* = 13.92, *SD* = 11.28	-4.17(1089.2)	-4.42 - -1.59	<.001
Age 17					
age	*M* = 17.49, *SD* = 0.34	*M* = 17.48, *SD* = 0.32	-0.56(1017.5)	-.05 - .03	.58
PH[*6]	*n* PH = 268	*n* PH = 122	48.48(1)		<.001
	n no-PH = 297	*n* no-PH = 345			
depression symptoms	*M* = 16.36, *SD* = 12.27	*M* = 12.38, *SD* = 10.19	-5.51(967.61)	-5.39 - -2.56	<.001
anxiety symptoms	*M* = 15.02, *SD* = 12.72	*M* = 11.53, *SD* = 10.96	-4.58(967.76)	-4.98 - -1.99	<.001

Note. CA = childhood adversity. SES = socio-economic status. [*1]We applied Welsh's two-tailed independent sample t-test to account for potentially unequal variances across groups. [*2]As SES was split in five ordered categories, we applied the two-tailed Asymptotic Cochran-Armitage test.[63] [*3]We applied two-tailed Pearson's chi-square tests. [*4]The confidence interval (CI) for the difference in location estimates, corresponding to the alternative hypothesis. [*5]SES was assessed with the ACORN classification system (http://www.caci.co.uk).[64] [*6]Psychiatric history was assessed with the Schedule for Affective Disorders and Schizophrenia for School-Age Children (Present and Lifetime Version), at age 14 additionally including learning disabilities, clinical sub-threshold diagnoses and deliberate self-harm, and at age 17 additionally incuding clinical sub-threshold diagnoses and deliberate self-harm.[65]

Table 3

RF and General Distress Comparisons: CA+ (n = 638) versus CA- (n = 501) Groups

	Age	CA+	CA-	*W* / χ^2(*df*)	95% *CI*[*1]	*p*[*2]
Friendship support (high)	14	0.08	0.20	110590	.01 - .22	.053
	17	0.08	0.24	113470	.06 - .27	<.01
Family support (high)	14	-0.02	0.16	109880	.07 - .29	<.01
	17	-0.05	0.14	109600	.06 - .32	<.01
Family cohesion (high)	14	-0.12	0.28	122750	.28 - .51	<.001
	17	-0.14	0.30	120380	.29 - .59	<.001
Negative self-esteem (low)	14	0.03	0.27	124670	.14 - .35	<.001
	17	0.12	0.42	126140	.18 - .42	<.001
Positive self-esteem (high)	14	-0.11	0.20	126480	.19 - .42	<.001
	17	-0.11	0.23	128500	.22 - .46	<.001
Ruminative brooding (low)	14	0.01	0.19	131070	.07 - .29	<.01
	17	-0.06	0.13	134770	.10 - .28	<.001
Reflective rumination (low)	14	0.10	0.19	123720	-.01 - .20	.075
	17	-0.08	0.01	124640	.01 - .16	.057
Distress tolerance (high)	14	-0.04	0.19	101290	.12 - .36	<.01
	17	0.06	0.38	105060	.19 - .46	<.001
Aggression (low)	14	low: 418 (s=1)	low: 391 (s=1)	8.69 (1)		<.01
		high: 87 (s=0)	high: 45 (s=0)			
	17	low: 460 (s=1)	low: 408 (s=1)	1.69 (1)		.193
		high: 45 (s=0)	high: 28 (s=0)			
Expressive suppression (low)	14	low: 293 (s=1)	low: 303 (s=1)	6.60 (1)		<.05
		high: 150 (s=0)	high: 104 (s=0)			
	17	low: 287 (s=1)	low: 290 (s=1)	3.78 (1)		.057
		high: 156 (s=0)	high: 117 (s=0)			
General Distress	14	-0.07	-0.39	72380	-.46 - -.17	<.001
	17	-0.11	-0.50	70110	-.57 - -.25	<.001

Note. CA = childhood adversity. All RFs are scored in such a way that high values are protective (e.g. high levels of high friendship support or high levels of low negative self-esteem) and low values are harmful (e.g. low levels of high friendship support or low levels of low negative self-esteem). The continuous general distress variable is scored in such a way that the higher the value the higher the level of general distress. [*1]The confidence interval (CI) for the difference in location estimates, corresponding to the alternative hypothesis. [*2]Please note the p-values are corrected for the false discovery rate, which is why the CIs do not have to contain 0 for the p-value to be nonsignificant.

Table 4

RF and General Distress Comparisons: Age 14 versus Age 17

	CA	Age 14	Age 17	*V*	*95% CI*[*1]	*p*[*2]	age$_x$CA[*3]	age$_x$CA *p*
Friendship support (high)	yes	0.08	0.08	59525	-.06 - .07	.94	-.05	.54
	no	0.20	0.24	42166	-.11 - .02	.35		
Family support (high)	yes	-0.02	-0.05	55752	-.05 - .09	.83	-.01	.94
	no	0.16	0.14	45375	-.05 - .08	.70		
Family cohesion (high)	yes	-0.12	-0.14	54995	-.06 - .10	.94	-.05	.64
	no	0.28	0.30	42117	-.10 - .06	.39		
Negative self-esteem (low)	yes	0.03	0.12	52153	-.17 - -.02	<.01	-.06	.51
	no	0.27	0.42	33490	-.22 - -.08	<.001		
Positive self-esteem (high)	yes	-0.11	-0.11	61899	-.06 - .07	.94	-.04	.65
	no	0.20	0.23	41857	-.10 - .04	.28		
Ruminative brooding (low)	yes	0.01	-0.06	77743	.01 - .13	<.05	-.01	.87
	no	0.19	0.13	55982	-.00 - .12	<.05		
Reflective rumination (low)	yes	0.10	-0.08	89421	.12 - .23	<.001	.01	.83
	no	0.19	0.01	64508	.12 - .25	<.001		
Distress tolerance (high)	yes	-0.04	0.06	37812	-.15 - -.06	<.001	-.09	.34
	no	0.19	0.38	21567	-.23 - -.15	<.001		
Aggression (low)	yes	low: 418 (=1)	low: 460 (=1)	1092		<.001	1.27	.46
		high: 87 (=0)	high: 45 (=0)					
	no	low: 391 (=1)	low: 408 (=1)	532		<.05		
		high: 45 (=0)	high: 28 (=0)					
Expressive suppression (low)	yes	low: 293 (=1)	low: 287 (=1)	4725		.83	1.11	.63
		high: 150 (=0)	high: 156 (=0)					
	no	low: 303 (=1)	low: 290 (=1)	3016		.28		
		high: 104 (=0)	high: 117 (=0)					
General Distress	yes	-0.07	-0.11	54349	-.03 - .15	.41	.07	.51
	no	-0.39	-0.50	42248	.01 - .21	<.05		

Note. CA = childhood adversity. All RFs are scored in such a way that high values are protective (e.g. high levels of high friendship support or high levels of low negative self-esteem) and low values are harmful (e.g. low levels of high friendship support or low levels of low negative self-esteem). The continuous general distress variable is scored in such a way that the higher the value the higher the level of general distress. [*1]The confidence interval (CI) for the difference in location estimates, corresponding to the alternative hypothesis. [*2]Please note the p-values are corrected for the false discovery rate, which is why the CIs do not have to contain 0 for the p-value to be nonsignificant. [*3]For linear models the interaction is reported as *b*-value and for binomial logit models as odds ratio.

Figure Legends

Figure 1. RF mean level comparisons. CA = childhood adversity. All scores are derived from strongly invariant confirmatory factor analyses. All RFs are scored in such a way that high values are protective (e.g. high levels of high friendship support or high levels of low negative self-esteem) and low values are harmful (e.g. low levels of high friendship support or low levels of low negative self-esteem). **Legend:** pos. = positive, exp. = expressive, dist. = distress, neg. = negative.

Figure 2. CA+ (*n* = 638) and CA- (*n* = 501) resilience factor networks for age 14 (upper panel) and age 17 (lower panel) corrected for the general distress variable. Width of the lines = association strength. Positive interrelations = blue, negative interrelations = red. **Legend:** Frn = friend support, fms = family support, fmc = family cohesion, ngt = negative self-esteem, pst = positive self-esteem, rfl = reflection, brd = brooding, dst = distress tolerance, agg = aggression, exp = expressive suppression, GD = general distress. The boxes depict the maximal interrelation difference between the respective two networks (M), the difference in global network expected influence (EI) between the respective two networks (EI), and the corresponding p-values (5000 comparison samples). The above networks with faded interrelations can be found in Supplement VIII. Please note, the upper panel of the Figure is similar to a Figure in a previous report on this sample (see [17] in Scientific Reports; can be retrieved from https://doi.org/10.1038/s41598-018-34130-2; information regarding the publishing license of the original Figure, and information regarding differences with the above Figure can be found in Supplement V).

Figure 3. Direct (DP) and shortest pathways (SP) between the resilience factors (RFs) and the general distress variable, for the CA+ (n = 638) and the CA- (n = 501) group. The upper panel depicts direct and the lower panel the shortest pathways between the RFs and general distress. Within the panels the upper part depicts the networks for age 14 and the lower part the networks for age 17. Non-transparent lines = direct/shortest pathway of interest. Transparent/dotted lines = all remaining partial regularized correlation relationships. Positive interrelations = blue, negative interrelations = red. **Legend:** Frn = friend support, fms = family support, fmc = family cohesion, ngt = negative self-esteem, pst = positive self-esteem, rfl = reflection, brd = brooding, dst = distress tolerance, agg = aggression, exp = expressive suppression. Please note, the upper part of the lower panel is similar to a Figure in a previous report on this sample (see [17] Scientific Reports; can be retrieved from https://doi.org/10.1038/s41598-018-34130-2; information regarding the publishing license of the original Figure, and information regarding differences with the above Figure can be found in Supplement V).

CA+	CA-

No RF change

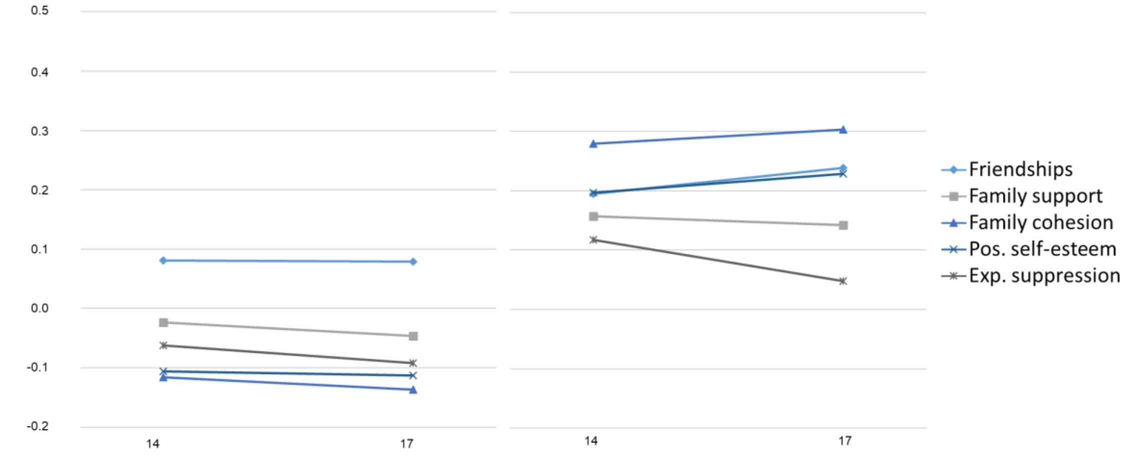

Increasing RFs

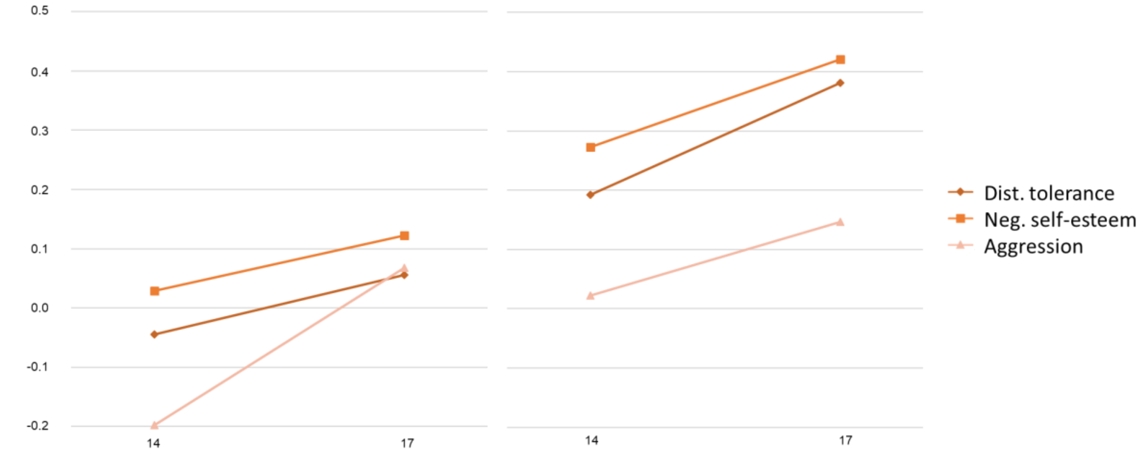

Decreasing RFs

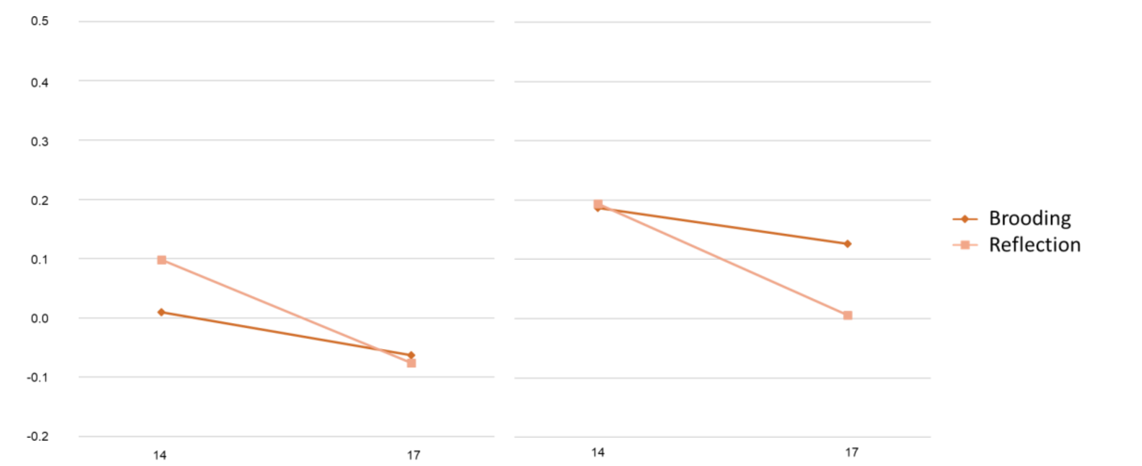

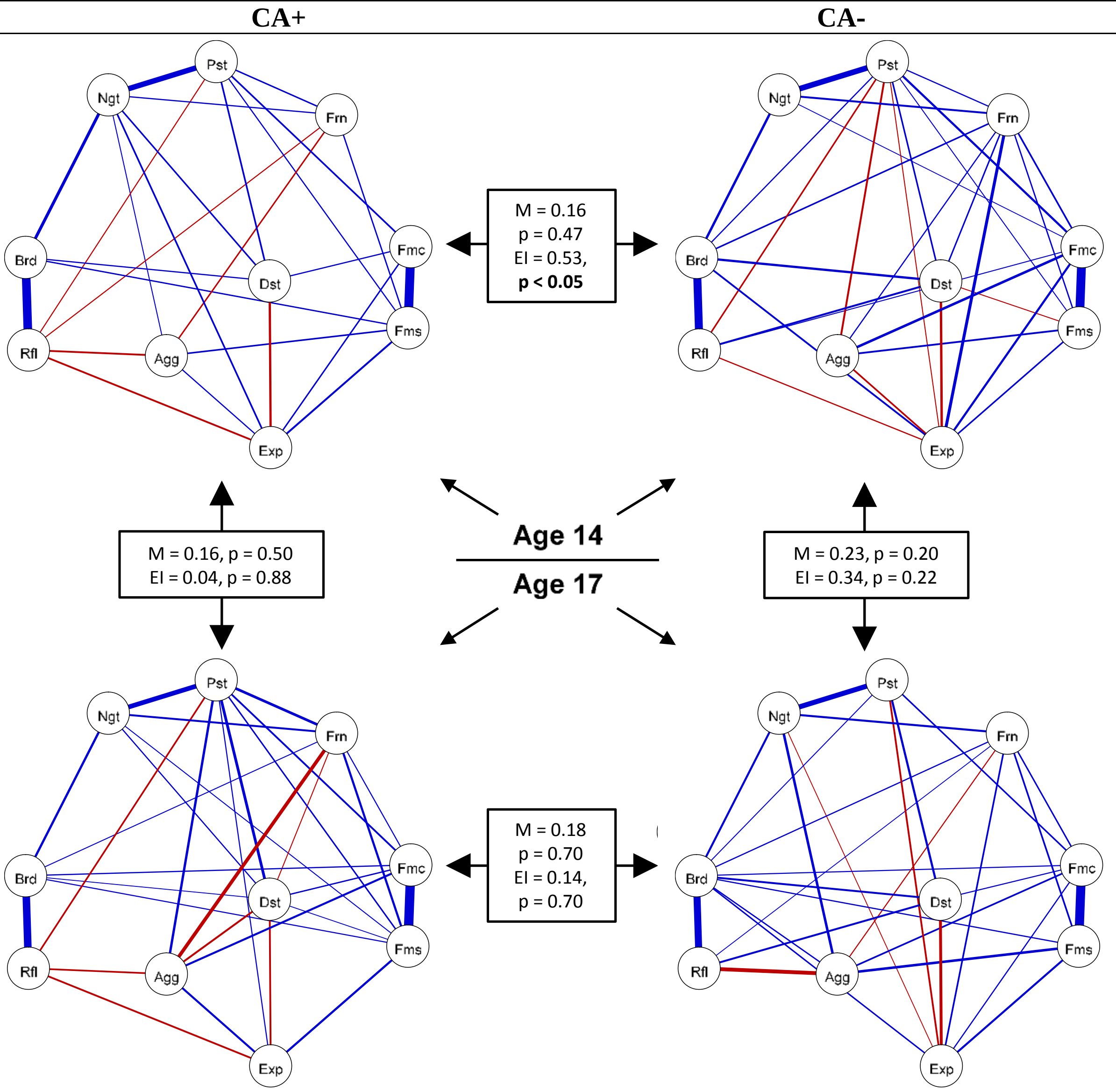

CA+
CA-
Pst
Ngt
Frn
Brd
Dst
Fmc
Fms
Rfl
Agg
Exp
M = 0.16
p = 0.47
EI = 0.53,
p < 0.05
Age 14
Age 17
M = 0.16, p = 0.50
EI = 0.04, p = 0.88
M = 0.23, p = 0.20
EI = 0.34, p = 0.22
M = 0.18
p = 0.70
EI = 0.14,
p = 0.70

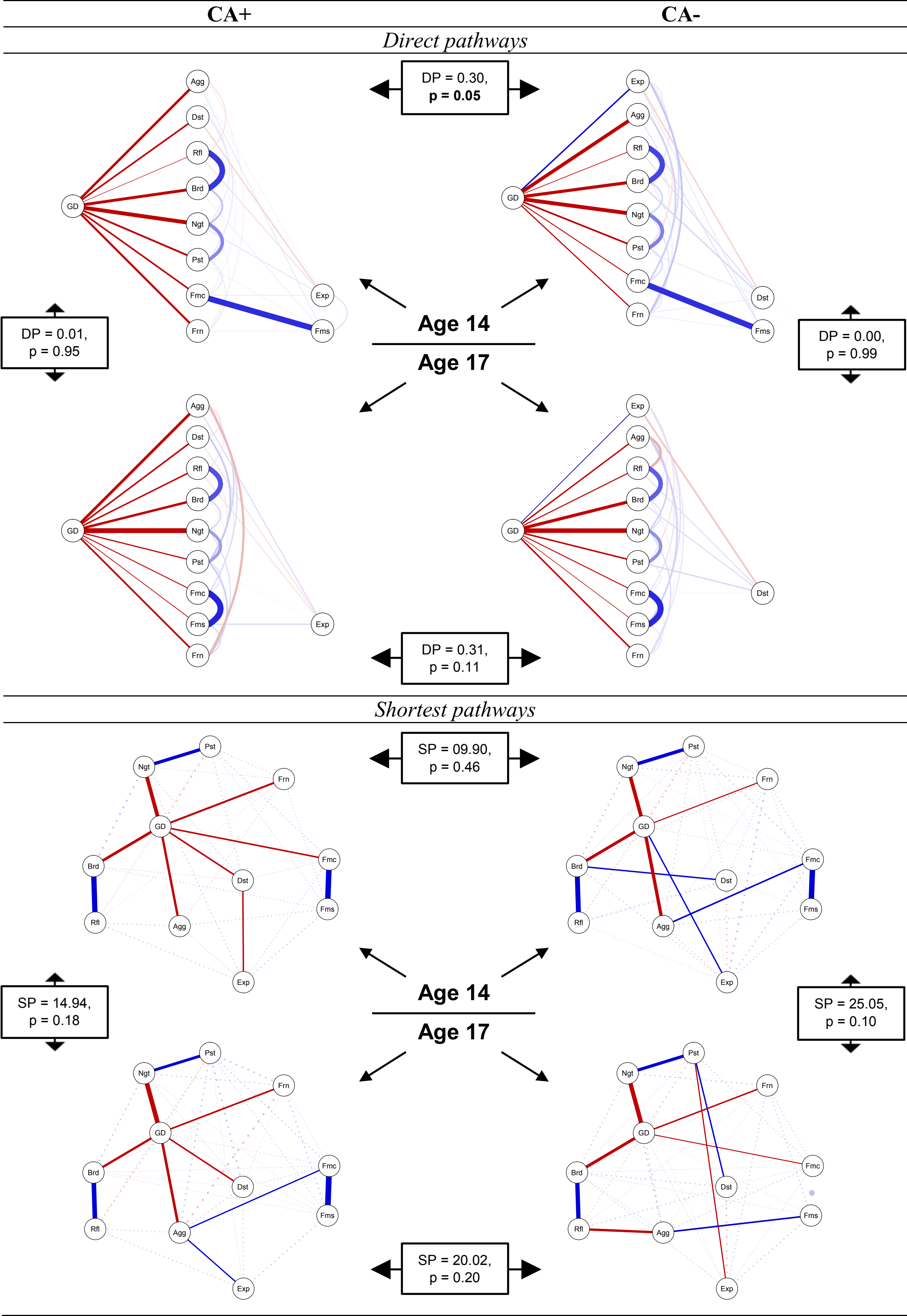

CA+
CA-
Direct pathways
DP = 0.30,
p = 0.05
DP = 0.01,
p = 0.95
DP = 0.00,
p = 0.99
Age 14
Age 17
DP = 0.31,
p = 0.11
Shortest pathways
SP = 09.90,
p = 0.46
SP = 14.94,
p = 0.18
SP = 25.05,
p = 0.10
Age 14
Age 17
SP = 20.02,
p = 0.20
GD
Agg
Dst
Rfl
Brd
Ngt
Pst
Fmc
Frn
Exp
Fms
