# Supplementary material for: Unravelling the complex nature of resilience factors and their changes between early and later adolescence
Source: BMC Med. 2019 Nov 14;17:203. doi: 10.1186/s12916-019-1430-6 (PMC6854636; doi:10.1186/s12916-019-1430-6)
Supplement: Supplementary file 18 — Additional file 18. Supplementary materials: Analysis results based on imputed data. [file 12916_2019_1430_MOESM18_ESM.zip › 5_Supplements_withoutImp_2019.08August.13.pdf]

Supplements for the Manuscript:

Unravelling the Complex Nature of Resilience Factors and their Changes between Early and Later Adolescence

J. Fritz*, J. Stochl, E. I. Fried, I. M. Goodyer, C. D. van Borkulo, P. O. Wilkinson[A], A.-L. van Harmelen[A]

* Correspondence: Jessica Fritz, jf585@cam.ac.uk

[A] shared last authorship

Supplement I

In our previous report[1] we used a distress index computed by Brodbeck and colleagues (2011)[2]. This index was however only computed for age 14. As we here wanted to compare two occasions, we decided to compute a separate general distress factor including information for both occasions so that we could apply invariance constraints over the two time points. This was for example necessary to compare the latent means. Moreover, in comparison to Brodbeck and colleagues (2011)[2] we simplified the index (e.g. through only using the short form rather than the complete version of the used depression scale), to ensure feasibility of the computation for the invariance models.

As the rumination questionnaire underwent revision between our two occasions, different versions of the questionnaire were used at the two occasions. The ruminative reflection factor stayed the same for both time points, however, we had to use another ruminative brooding factor than in Fritz, Fried et al. [1] as only two of the five originally used items were available for both occasions. Here we report results based on a ruminative brooding approach that has been established by Burwell and Shirk (6 items)[3] for a version of the rumination questionnaire that matches the one used at our second occasion, as five of the six identified items of that approach were available for both of our occasions. We also analysed the network models excluding the brooding variable, which revealed similar results, see Supplement XII.

Supplement II

We conducted all analyses in R version 3.5.1, and used the below packages (see Table 1), and further dependencies these packages load.

Table 1

Used R packages, including their version number and reference

**Package (version number)**	**Reference**
mice (3.5.0)	van Buren, S. & Groothuis-Oudshoorn (2011)[4]
dplyr (0.7.7)	Wickham, H., François, R., Henry, L. & Müller (2018)[5]
pastecs (1.3.21**)**	Grosjean, P. & Ibanez, F (2018)[6]
coin (1.2-2)	Hothorn, T., Hornik, K., van de Wiel, M. A. & Zeileis, A (2008)[7]
reshape (0.8.8)	Wickham, H (2007)[8]
sjPlot (2.6.2)	Lüdecke, D (2018)[9]
lavaan (0.6-4)	Rosseel, Y (2012)[10]
semTools (0.5-1.933)	Jorgensen, T. D., Pornprasertmanit, S., Schoemann, A. M. & Rosseel, Y (2018)[11]
ggplot2 (3.1.0)	Wickham, H (2016)[12]
qgraph (1.5)	Epskamp, S., Cramer, A. O. J., Waldorp, L. J., Schmittmann, V. D. & Borsboom, D (2012)[13]
bootnet (1.1.0)	Epskamp, S., Borsboom, D. & Fried, E. I (2018)[14]
NetworkComparisonTest (2.0.1)	van Borkulo, C. D (2018)[15]

Supplement III

We decided to use factor scores, instead of sum scores, for two reasons. Firstly, to remove as much measurement error as possible from the latent resilience factor (RF) variables. In most published network analysis manuscripts author have used item level data. Yet, here we were not interested in the individual items but in RF constructs which were derived from a previous systematic review. As all RFs (except for expressive suppression) consisted of more than 3 items we could apply factor analyses to effectively reduce measurement error. A similar method would have been to use latent network modelling, which does the same but estimates the factor scores and the network models in one step.[16] Upon closer inspection we concluded that latent network modelling is as yet only (or at least particularly) applicable to smaller models. The second reason for using factor scores was that when using sum scores one assumes that all items have the same importance and hence go with the same weight into the construct (i.e. tau equivalence). However, when using factor scores, the factor loadings enable every item to have a unique weight for the latent construct, which means that items can differ in importance, enhancing construct validity. We felt that this point was particularly important as many of our used (sub-)scales did not consist of a large number of items (with exception for the general distress factor).

As we aimed to compare two time points, we estimated longitudinal CFAs (LCFAs) separately for each RF and the general distress variable. Given that all of the RF items (as well as the general distress items) were assessed with three to six answer categories, we computed categorical LCFAs and treated the items as ordinal (i.e. ordered categorical) indicators.[17–20] Accordingly, we used the weighted least squares mean and variance adjusted (WLSMV) estimator. The categorical LCFAs were specified as shown in Figure 1 (which is modelled along examples of [17]). We identified the model as suggested by Wu and Estabrook,[20] using the theta parametrization. We estimated, a configural (i.e. baseline) model, a strong invariance and a full invariance model. For the strong invariance LCFAs we equated item loadings and item thresholds across the two time points (i.e. age 14 and 17), fixed all item intercepts to 0, the item variance of the first time point to 1, the latent factor mean of the first time point to 0, and the latent factor variance of the first time point to 1 (item covariances and the latent factor covariance were freely estimated). For the full invariance LCFAs we again equated item loadings and item thresholds across the two time points (i.e. age 14 and 17) and fixed all item intercepts to 0, this time however we fixed all item variances to 1, both latent factor means to 0, and both latent factor variances to 1 (item covariances and the latent factor covariance were again freely estimated). A model specification overview can be found in Table 2. Table 3 depicts the fit indices for all models. We only applied modification indices when they were theoretically justified. All models seemed to fit acceptably. Factor scores

derived from the aggression models were however so poorly distributed that we had to binarize those scores. Distribution plots (i.e. box-and-whisker plots with individual data points) for the RFs (except for expressive suppression and aggression) and the general distress variable are depicted in Figure 2.

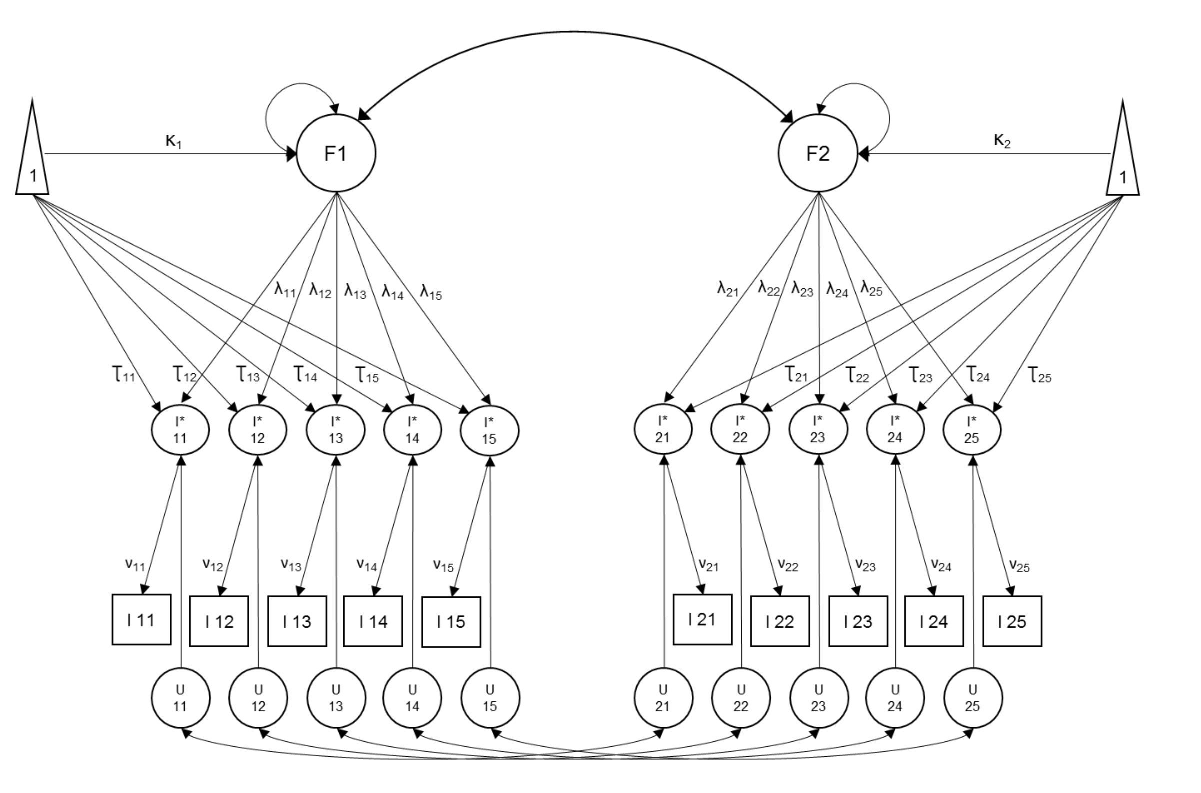

Figure 1. Longitudinal, categorical CFA model, with 5 categorical items assessed at two time points. The model is defined as follows: F = **common latent factor** (with the factor number indicating the corresponding time point); κ = **common latent factor means** (with the subscript number indicating the corresponding latent factor); I = **categorical observed items**; I* = **continuous latent item responses** inferred from the categorical observed items and described by the item thresholds; λ = **item loadings**; τ= **item intercepts**; ν = **item thresholds** (the number of thresholds is not depicted as every ordinal item has multiple thresholds, namely one less than the number of measured categories); U = **unique latent (item) factors;** for indicators with two numbers, the first number refers to the time point and the second to the item number; ↔ = two-sided arrows indicate (auto-co)variances. The Figure is modelled along examples of Liu, Millsap, West, Tein, Tanaka and Grimm (2017)[17]: https://doi.org/10.1037/met0000075.

Table 2

Model specifications for the three estimated invariance levels of the categorical LCFAs

(1) Configural Model

Estimated parameters:

1. λ = **factor loadings:** all freely estimated
2. ν = **items thresholds:** all freely estimated
3. auto-covar(U) = **unique latent (item) factor auto-covariances:** between the corresponding time 1 and time 2 unique latent (item) factors are all freely estimated
4. auto-covar(F) = **common latent factor auto-covariance:** between the corresponding time 1 and time 2 common latent factor is freely estimated

Parameters fixed for both time points:

1. τ = **item intercepts:** all fixed to zero
2. var(U) = **unique latent (item) factor variances**: are all fixed to one
3. κ = **common latent factor means:** all fixed to zero
4. var(F) = **common latent factors variances**: are all fixed to one

Parameters fixed for only the first but estimated for the second time point:

-

Equated parameters across time:

-

(2) Strong Invariance Model

Estimated parameters:

1. auto-covar(U) = **unique latent (item) factor auto-covariances:** between the corresponding time 1 and time 2 unique latent (item) factors are all freely estimated
2. auto-covar(F) = **common latent factor auto-covariance:** between the corresponding time 1 and time 2 common latent factor is freely estimated

Parameters fixed for both time points:

1. τ = **item intercepts:** all fixed to zero

Parameters fixed for only the first but estimated for the second time point:

1. var(U) = **unique latent (item) factor variances:** fixed to one only for the first, but not the second time point
2. κ = **common latent factor means:** fixed to zero only for the first, but not the second time point

3. var(F) = **common latent factor variances:** fixed to one only for the first, but not the second time point

Equated parameters across time:

1. λ = **factor loadings:** all equated across time
2. ν = **items thresholds:** all equated across time

(3) Full Invariance Model

Estimated parameters:

1. auto-covar(U) = **unique latent (item) factor auto-covariances:** between the corresponding time 1 and time 2 unique latent (item) factors are all freely estimated
2. auto-covar(F) = **common latent factor auto-covariance:** between the corresponding time 1 and time 2 common latent factor is freely estimated

Parameters fixed for both time points:

1. τ = **item intercepts:** all fixed to zero
2. var(U) = **unique latent (item) factor variances:** all fixed to one
3. κ = **common latent factor means:** all fixed to zero
4. var(F) = **common latent factor variances:** all fixed to one

Parameters fixed for only the first but estimated for the second time points:

-

Equated parameters across time:

1. λ = **factor loadings:** all equated across time
2. ν = **items thresholds:** all equated across time

Table 3

Longitudinal, Categorical Confirmatory Factor Analyses Conducted with the WLSMV Estimator

**Model**	**Scaled CFI**	**Scaled TLI**	**Scaled RMSEA**	**RMSEA 90% CI**	**Chi^2**
Friendship support,[21] 5 items, 0 unique item covariances, n = 941					
CM	0.98	0.96	0.08	0.07 – 0.09	140
SIM	0.98	0.97	0.06	0.06 – 0.07	165
FIM	0.98	0.98	0.06	0.05 – 0.07	211
Family support, [22] 5 items, 0 unique item covariances, n = 915					
CM	0.99	0.99	0.05	0.04 – 0.06	52
SIM	0.99	0.99	0.04	0.03 – 0.05	72

FIM	0.99	0.99	0.05	0.04 – 0.06	153
Family cohesion, [22] *7 items, 1 unique item covariance, n = 917*					
CM	0.99	0.98	0.05	0.04 – 0.06	171
SIM	0.98	0.98	0.05	0.04 – 0.06	210
FIM	0.98	0.98	0.06	0.05 – 0.06	385
Positive self-esteem,[23] *5 items, 0 unique item covariances, n = 955*					
CM	0.996	0.99	0.06	0.05 – 0.07	58
SIM	0.996	0.996	0.05	0.04 – 0.06	70
FIM	0.997	0.997	0.04	0.03 – 0.05	115
Negative self-esteem, [23] *5 items, 0 unique item covariances, n = 961*					
CM	0.999	0.998	0.02	0.01 – 0.04	24
SIM	0.997	0.996	0.03	0.02 – 0.04	45
FIM	0.99	0.99	0.04	0.03 – 0.05	136
Brooding,[3,24] *7 items, 0 unique item covariances, n = 1004*					
CM	0.99	0.99	0.05	0.04 – 0.06	56
SIM	0.99	0.99	0.04	0.03 – 0.05	75
FIM	0.98	0.98	0.05	0.05 – 0.06	182
Reflection,[3,24] *5 items, 1 unique item covariance, n = 1000*					
CM	0.99	0.98	0.06	0.04 – 0.07	84
SIM	0.98	0.98	0.06	0.05 – 0.07	135
FIM	0.97	0.97	0.07	0.07 – 0.08	307
Distress tolerance,[25] *5 items, 1 unique item covariance, n = 849*					
CM	0.97	0.95	0.11	0.10 – 0.12	172
SIM	0.97	0.97	0.08	0.07 – 0.09	184
FIM	0.97	0.98	0.08	0.07 – 0.08	268
Aggression, [26] *4 items, 0 unique item covariances, n = 975*					
CM	0.99	0.99	0.02	0.00 – 0.04	12
SIM	0.997	0.997	0.01	0.00 – 0.03	17
FIM	0.97	0.97	0.03	0.02 – 0.04	53
General distress,[27,28] *41 items, 2 unique item covariances, n = 865*					
CM	0.97	0.96	0.03	0.03 – 0.03	6537
SIM	0.96	0.96	0.03	0.03 – 0.03	7031
FIM	0.97	0.97	0.03	0.03 – 0.03	8895

Note. WLSMV = weighted least squares estimator with mean- and variance corrected test statistics and robust standard errors. CFI = Comparative fit index, TLI = Tucker-Lewis index, RMSEA = Root mean square error of approximation, CI = Confidence interval, CM = configural model, SIM = strong invariance model, FIM = full invariance model.

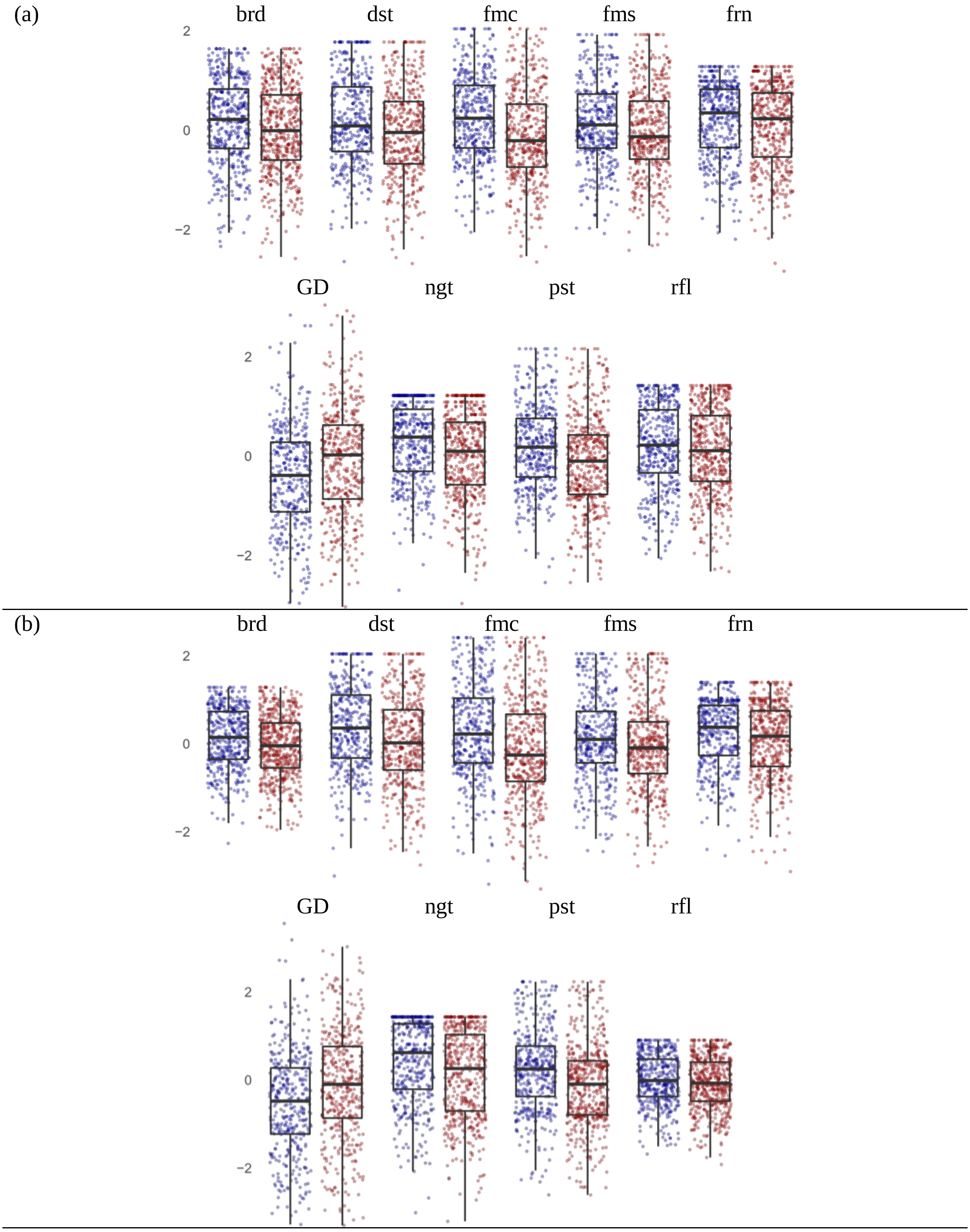
(a)
brd
dst
fmc
fms
frn
GD
ngt
pst
rfl
2
0
−2
(b)
brd
dst
fmc
fms
frn
GD
ngt
pst
rfl
2
0
−2

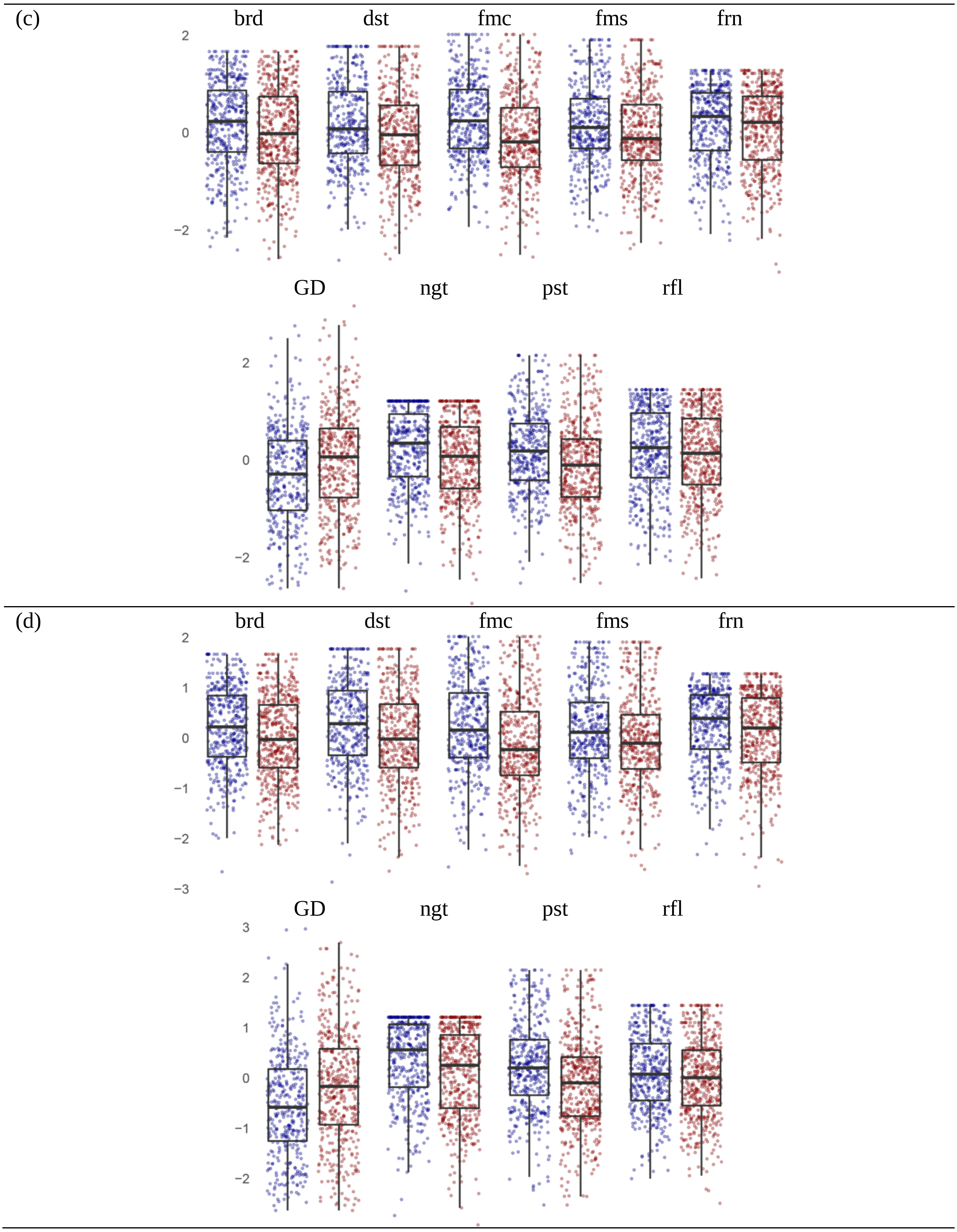

Figure 2. Box-and-whisker plots with individual data points for the RFs (except expressive suppression and aggression) and the general distress variable, separately for CA+ (n = 638) and CA- (n = 501). Panel (a) depicts the distributions for the strongly invariant scores for age 14 and panel (b) for age 17. Panel (c) depicts the distributions for the fully invariant scores for age 14 and panel (d) for age 17. CA- group = blue data points, CA+ group = red data points. Center line = median (50% quantile); lower box limit =25% quantile; upper box limit = 75% quantile; lower whisker = smallest observation greater than or equal to the lower box limit - 1.5 x Inter Quartile Range (IQR); upper whisker = largest observation less than or equal to upper box limit + 1.5 x IQR; outliers = data points beyond the end of the whiskers. **Legend:** Brd = brooding, dst = distress tolerance, fmc = family cohesion, fms = family support, frn = friend support, ngt = negative self-esteem, GD = general distress, pst = positive self-esteem, rfl = reflective rumination.

Supplement IV

We compared the RF and general distress mean levels between age 14 and age 17, separately in the CA+ and CA- groups, for the strong invariance and the full invariance model. Results for the strong invariance model can be found in the main manuscript (see also Figure 1 in the main manuscript). For the full invariance factor scores, the same two RFs as for the strongly invariant factor scores had for both CA+ and CA- adolescents higher mean levels at age 17 than at age 14 (see Figure 3): i.e. low negative self-esteem and high distress tolerance. As for the strongly invariant factor scores, reflection had for both adolescents with and without CA lower mean levels at age 17 than at age 14. Five RFs did not significantly change from age 14 to age 17 (i.e. friendship support, family support, family cohesion, positive self-esteem, and ruminative brooding). The binarized aggression and expressive suppression RFs are the same variables as reported in the main manuscript. Hence, aggression increased from age 14 to age 17 in the CA+ group and expressive suppression did not change. Moreover, for both adolescents with and without CA the general distress level was significantly higher at age 14 than at age 17. In sum, the results for the fully invariant factor scores were mainly comparable to the findings for the strongly invariant factor scores, only brooding did no longer decrease in mean level, and general distress did decrease in mean level in both groups rather than only in the CA- group.

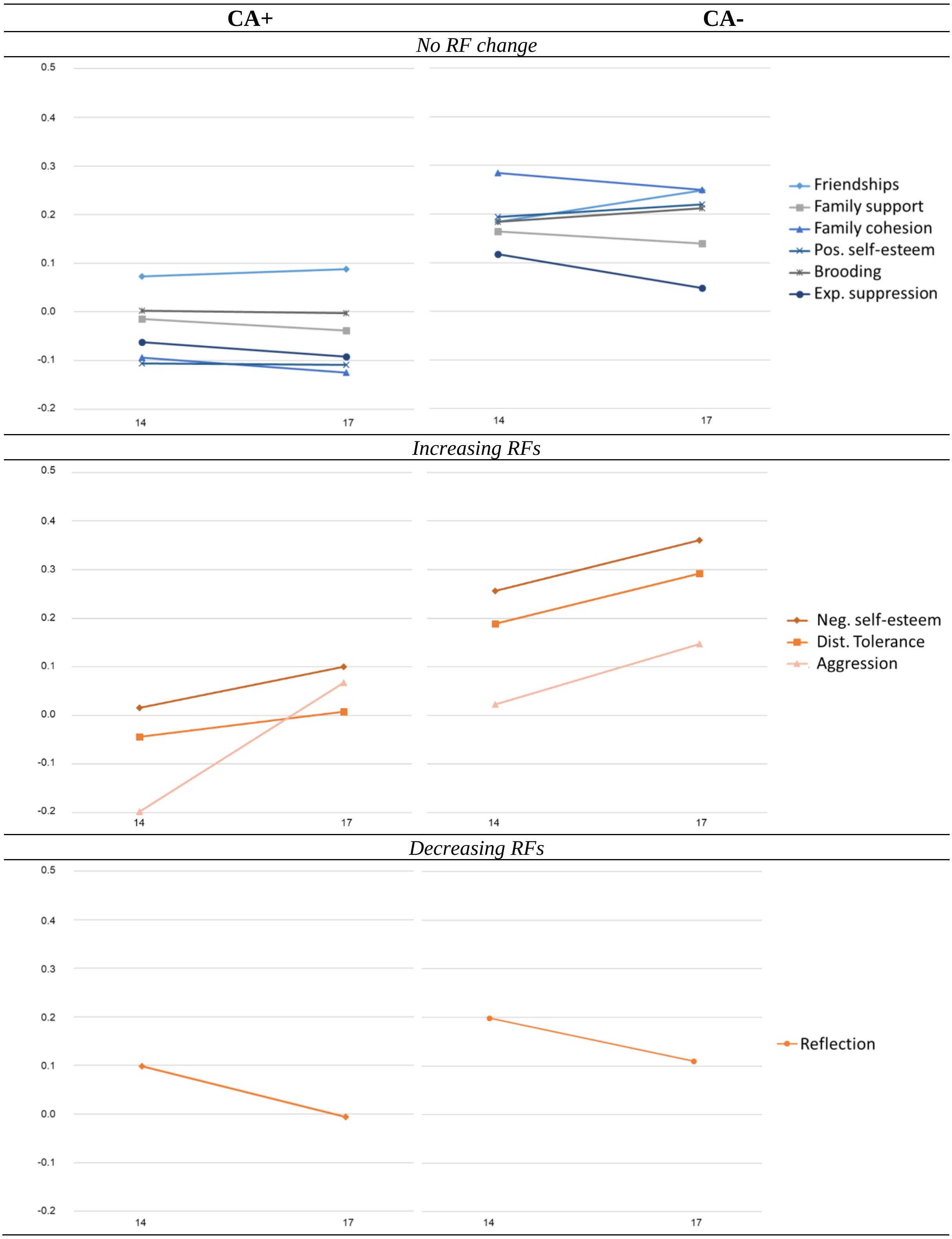

Figure 3. RF and general distress mean level comparisons: age 14 versus age 17. CA = childhood adversity. All scores are derived from fully invariant confirmatory factor analyses. All RFs are scored in such a way that high values are protective (e.g. high levels of high friendship support or high levels of low negative self-esteem) and low values are harmful (e.g. low levels of high friendship support or low levels of low negative self-esteem). **Legend:** pos. = positive, exp. = expressive, neg. = negative, dist. = distress.

Supplement V

Several network figures for age 14 (i.e. Figure 2 and 3 in the main manuscript, and figures in Supplement III, VII, VIII, XII, XIII) are similar to figures in a previous report on this sample Fritz et al. (Scientific Reports; can be retrieved from https://doi.org/10.1038/s41598-018-34130-2).[1] In the original article, the figures were published under the Creative Commons Attribution 4.0 International License. Information about this license can be found in the article itself[1] or at http://creativecommons.org/licenses/by/4.0/. The figures here are however only partially similar to the figures in our previous report, for the following reasons: (1) a smaller sample was used due to attrition at age 17, (2) the general distress variable was not the same for reasons described in Supplement I, (3) the brooding variable was not the same for reasons described in Supplement I, (4) the scores were not derived from one-factor CFAs, but from longitudinal categorical CFAs with two factors, one for each time point, (5) due to computing different CFA models, some CFAs did not need the modification we had to apply for the CFAs in our previous report.

Supplement VI

Table 4

Significant RF-RF Interrelation Differences between the CA+ (n = 638) and the CA- (n = 501) Networks

RF1	RF2	interrelation sign in the CA+ network	interrelation sign in the CA- network	E	*p*
		Age 14			
friendship support	expressive suppression	null	positive	0.16	.014
		Age 17			
friendship support	positive self-esteem	positive	null	0.14	.012
positive self-esteem	expressive suppression	positive	negative	0.11	.043

Note. RF = Resilience factor. CA = childhood adversity. E = RF-RF interrelation difference (i.e. edge difference).

Table 5

Significant RF-RF Interrelation Differences between Age 14 and Age 17 Networks

RF1	RF2	interrelation sign in the age 14 network	interrelation sign in the age 17 network	E	*p*
		CA+ Networks			
positive self-esteem	aggression	null	positive	0.12	.049
brooding	reflection	more positive	less positive	0.08	.041
		CA- Networks			
reflection	aggression	null	negative	0.23	.008

Note. RF = Resilience factor. CA = childhood adversity. E = RF-RF interrelation difference (i.e. edge difference).

Supplement VII

RF networks without the general distress variable. At age 14, the network invariance test was not significant for the RF networks without the general distress variable (M = .13, p = .80; see Figure 4), and the global network expected influence (EI) did not differ between the CA+ and the CA- RF networks (EI_{CA+} = 3.22, EI_{CA-} = 3.30, EI = 0.08, p = .68). Those findings were similar in the RF networks for age 17, as neither of the two tests revealed significant differences between the CA+ and the CA- group (M = .18, p = .69; EI_{CA+} = 3.30, EI_{CA-} = 3.00, EI = 0.30, p = .22). At age 14 two interrelations differed between the CA+ and the CA- networks: namely friendship support and expressive suppression (CA+: null; CA-: positive), and negative self-esteem and distress tolerance (CA+: positive; CA-: null). At age 17, the interrelation between friendship support and positive self-esteem (CA+: positive, CA-: null), and positive self-esteem and expressive suppression (CA+: positive; CA-: negative) differed between the CA+ and the CA- network.

Interestingly, when we compared the RF networks for age 14 and age 17, we did not find any significant global network structure differences; neither for adolescents with (M = .15, p = .49; EI_{14} = 3.22, EI_{17} = 3.30, EI = 0.08, p = .64) nor for adolescents without a history of adversity (M = .18, p = .45; EI_{14} = 3.30, EI_{17} = 3.00, EI = 0.30, p = .17). In the CA+ network two RF interrelations changed from age 14 to age 17, namely the interrelation between positive and negative self-esteem (from more to less positive), and the interrelation between positive self-esteem and aggression (from null to positive). In the CA- network, one RF interrelations changed from age 14 to age 17, namely the interrelation between reflection and aggression (from null to negative).

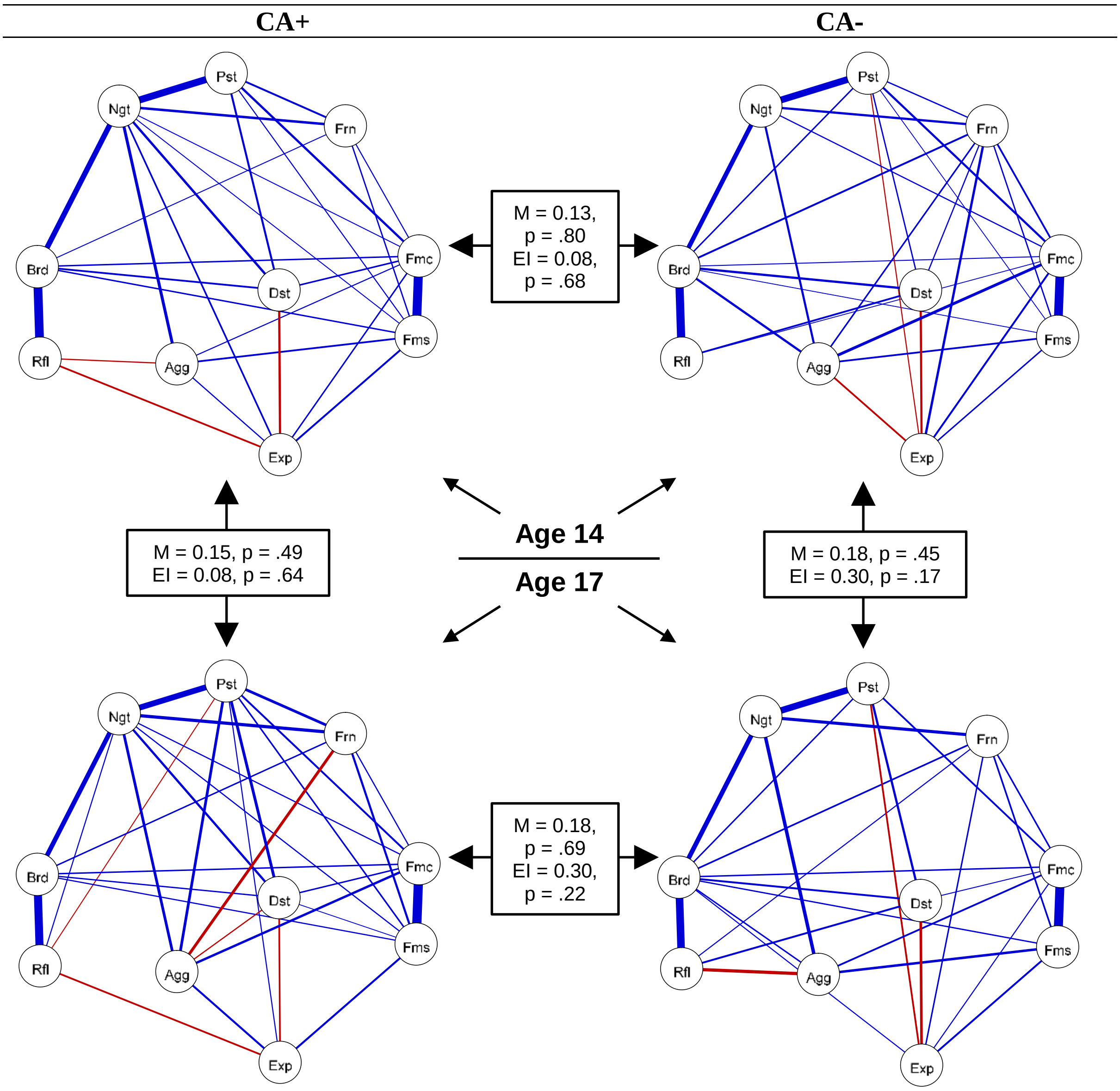

Figure 4. CA+ (*n* = 638) and CA- (*n* = 501) resilience factor networks for age 14 (upper panel) and age 17 (lower panel) without the general distress variable. Width of the lines = association strength. Positive interrelations = blue, negative interrelations = red. **Legend:** Frn = friend support, fms = family support, fmc = family cohesion, ngt = negative self-esteem, pst = positive self-esteem, rfl = reflection, brd = brooding, dst = distress tolerance, agg = aggression, exp = expressive suppression. The boxes depict the maximal interrelation difference between the respective two networks (M), the difference in global network expected influence (EI) between the respective two networks (EI), and the corresponding p-values (5000 comparison samples). The above networks with faded interrelations can be found in Supplement VIII.

RF networks with the general distress variable. At age 14, the network invariance test was also not significant for the RF networks with the general distress variable (M = .16, p = .53; see Figure 5). However, the network expected influence was significantly higher in the CA- than in the CA+ network (EI_{CA+} = 0.63, EI_{CA-} = 1.46, EI = 0.83, p = .03). Those findings were only partially similar in the networks for age 17, as neither of the two tests revealed significant differences between the CA+ and the CA- group at age 17 (M = .18, p = .76; EI_{CA+} = 0.66, EI_{CA-} = 1.12, EI = 0.45, p = .38). At age 14, three interrelation differed between the CA+ and the CA- network, namely the interrelations between friendship support and expressive suppression (CA+: null, CA-: positive), positive self-esteem and brooding (CA+: null, CA-: positive), as well as between distress tolerance and general distress (CA+: negative, CA-: null). At age 17 two interrelation differed between the CA+ and the CA- network, namely the interrelation between friendship support and positive self-esteem (CA+: positive, CA-: null), as well as the interrelation between positive self-esteem and expressive suppression (CA+: positive, CA-: negative).

When we compared those networks for age 14 and age 17, we again did not find any significant global network structure differences, neither for adolescents with (M = .16, p = .55; EI_{14} = 0.63, EI_{17} = 0.66, EI = 0.03, p = .94) nor for adolescents without a history of adversity (M = .25, p = .17; EI_{14} = 1.46, EI_{17} = 1.12, EI = 0.34, p = .38). In the CA+ network two individual RF interrelations changed from age 14 to age 17, namely the interrelations between negative self-esteem and general distress (from less to more negative), as well as the interrelation between brooding and reflection (from more to less positive). In the CA- network, also two interrelation changed from age 14 to age 17, namely the interrelation between reflection and aggression (from null to negative), as well as the interrelation between aggression and general distress (from more to less negative).

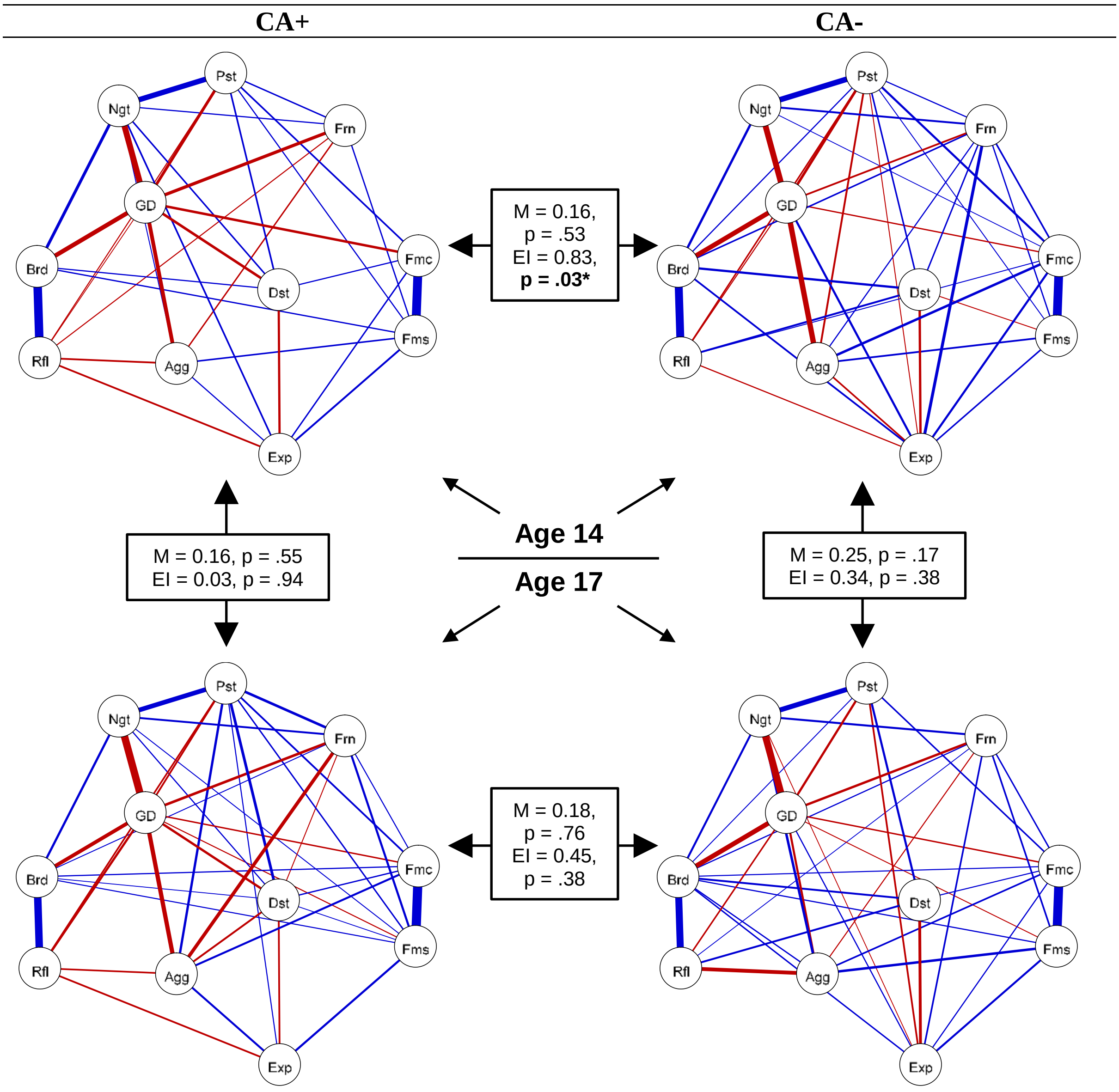

Figure 5. CA+ (*n* = 638) and CA- (*n* = 501) resilience factor networks for age 14 (upper panel) and age 17 (lower panel) with the general distress variable. Width of the lines = association strength. Positive interrelations = blue, negative interrelations = red. **Legend:** Frn = friend support, fms = family support, fmc = family cohesion, ngt = negative self-esteem, pst = positive self-esteem, rfl = reflection, brd = brooding, dst = distress tolerance, agg = aggression, exp = expressive suppression, GD = general distress. The boxes depict the maximal interrelation difference between the respective two networks (M), the difference in global network expected influence (EI) between the respective two networks (EI), and the corresponding p-values (5000 comparison samples). The above networks with faded interrelations can be found in Supplement VIII.

Supplement VIII

The following three figures depict CA+ and CA- networks with faded interrelations, for both age 14 and age 17, for (1) the networks without the general distress variable, (2) the networks with the general distress variable, and (3) the networks corrected for the general distress variable.

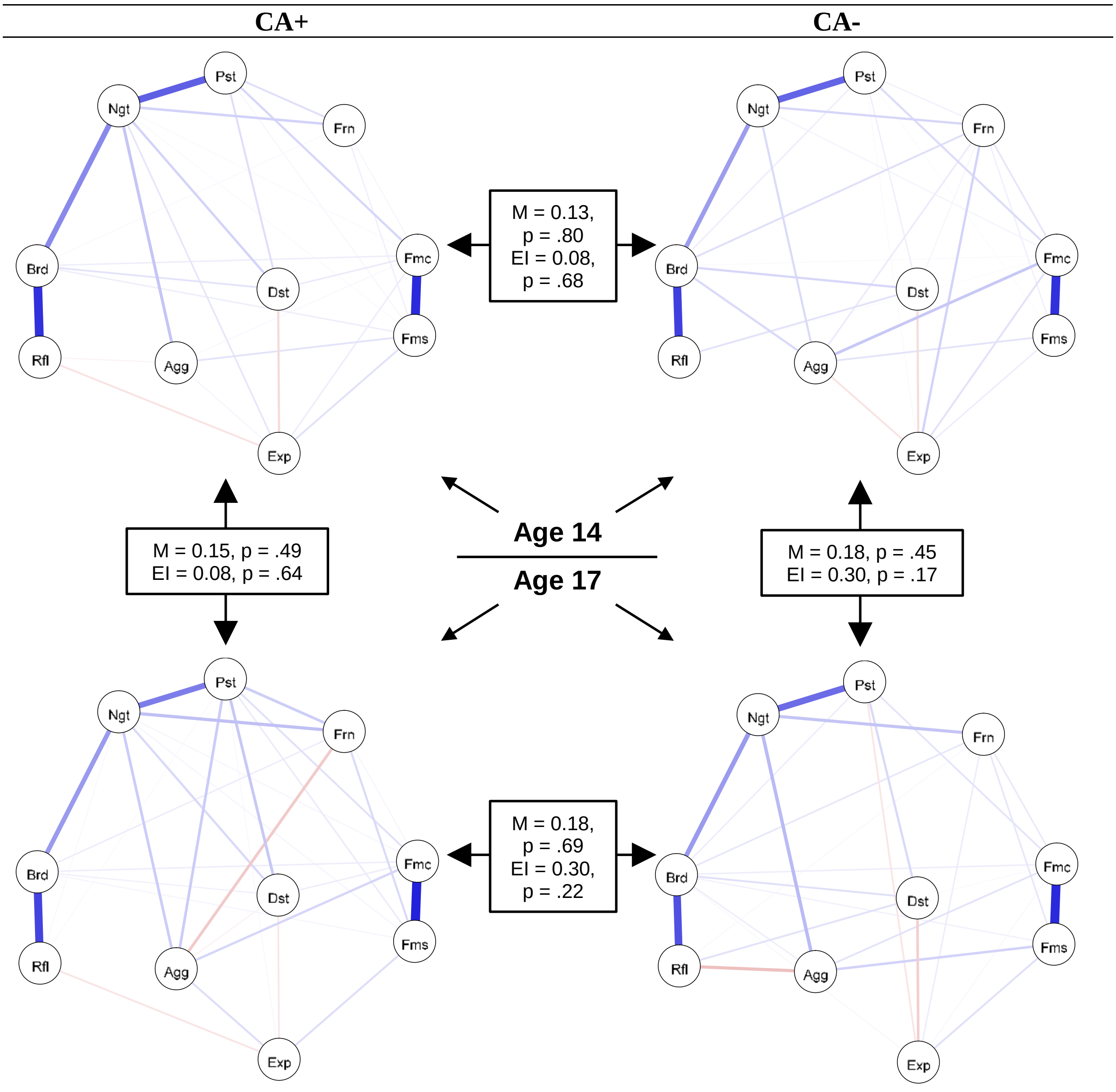

Figure 6. CA+ (*n* = 638) and CA- (*n* = 501) resilience factor networks with faded interrelations for age 14 (upper panel) and age 17 (lower panel) without the general distress variable. Width of the lines = association strength. Positive interrelations = blue, negative interrelations = red. **Legend:** Frn = friend support, fms = family support, fmc = family cohesion, ngt = negative self-esteem, pst = positive self-esteem, rfl = reflection, brd = brooding, dst = distress tolerance, agg = aggression, exp = expressive suppression. The boxes depict the maximal interrelation difference between the respective two networks (M), the difference in global network expected influence (EI) between the respective two networks (EI), and the corresponding p-values (5000 comparison samples).

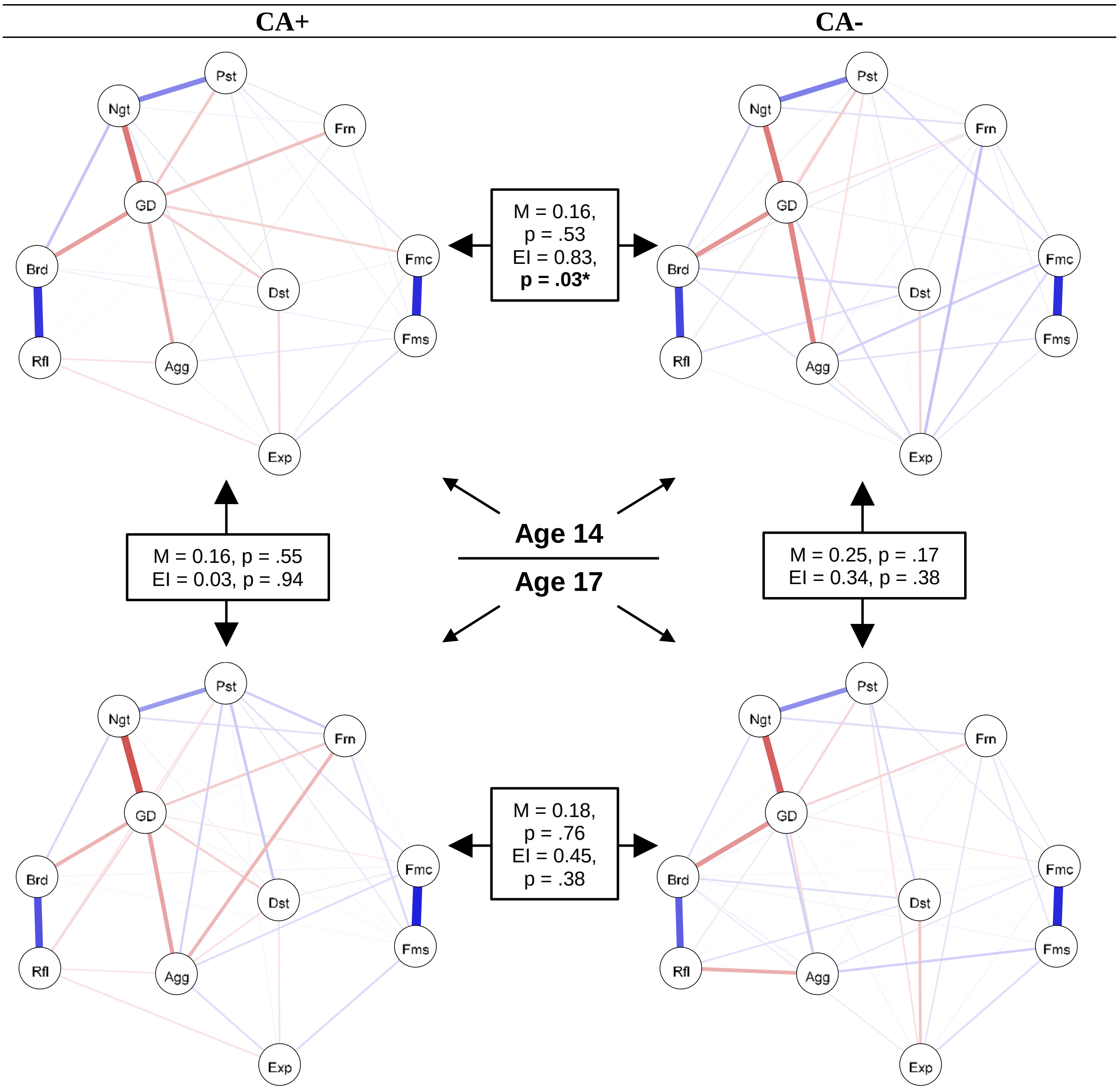

Figure 7. CA+ (*n* = 638) and CA- (*n* = 501) resilience factor networks with faded interrelations for age 14 (upper panel) and age 17 (lower panel) with the general distress variable. Width of the lines = association strength. Positive interrelations = blue, negative interrelations = red. **Legend:** Frn = friend support, fms = family support, fmc = family cohesion, ngt = negative self-esteem, pst = positive self-esteem, rfl = reflection, brd = brooding, dst = distress tolerance, agg = aggression, exp = expressive suppression, GD = general distress. The boxes depict the maximal interrelation difference between the respective two networks (M), the difference in global network expected influence (EI) between the respective two networks (EI), and the corresponding p-values (5000 comparison samples).

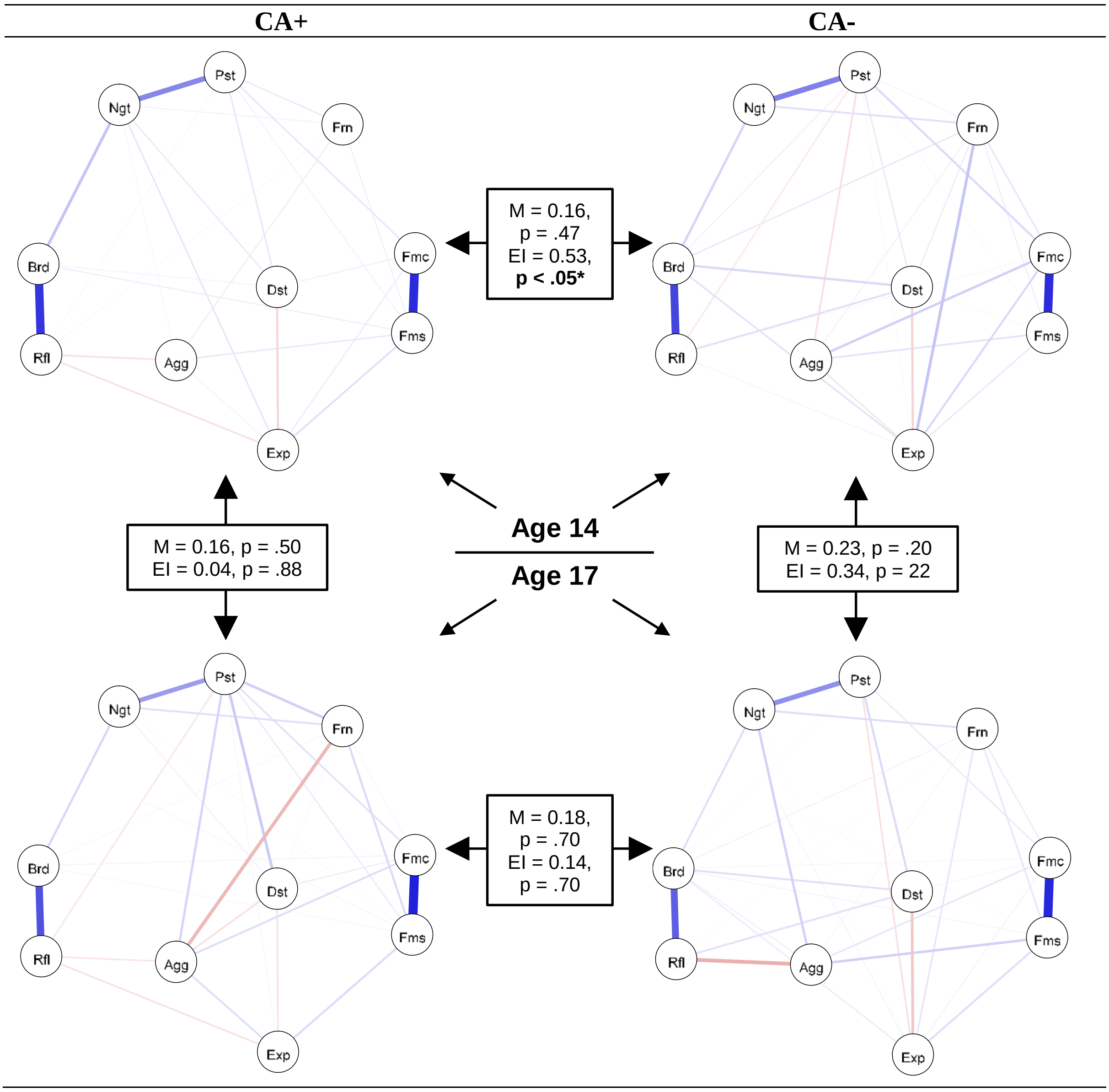

Figure 8. CA+ (*n* = 638) and CA- (*n* = 501) resilience factor networks with faded interrelations for age 14 (upper panel) and age 17 (lower panel) corrected for the general distress variable. Width of the lines = association strength. Positive interrelations = blue, negative interrelations = red. **Legend:** Frn = friend support, fms = family support, fmc = family cohesion, ngt = negative self-esteem, pst = positive self-esteem, rfl = reflection, brd = brooding, dst = distress tolerance, agg = aggression, exp = expressive suppression. The boxes depict the maximal interrelation difference between the respective two networks (M), the difference in global network expected influence (EI) between the respective two networks (EI), and the corresponding p-values (5000 comparison samples).

Supplement IX

As can be seen in Table 6, all RFs were negatively correlated with general distress, except for expressive suppression for CA- adolescents, which was at both age 14 and 17 positively correlated with general distress. Regularized partial correlations of the RFs, which we used for the network models, were also mostly negative for the relationships between the RFs and general distress. Yet, in the CA+ group expressive suppression was not related with general distress, and in the CA- group expressive suppression was positively related with general distress, at both age 14 and 17. Moreover, at age 14, family support was no longer related with general distress, in both the CA+ and the CA- group. Furthermore, distress tolerance was no longer associated with general distress in the CA- group, at both age 14 and 17. However, the overall results pattern is still similar, showing that even after the correction for all other RFs, most RFs are clearly negatively associated with general distress. As can be seen in Figure 9, negative self-esteem, positive self-esteem and brooding seemed to be most strongly correlated with general distress. However, in terms of partial correlations, negative self-esteem, brooding and aggression appeared to have the highest interrelations with general distress.

Table 6

Correlations and Regularized Partial Correlations between the RFs and the General Distress Variable

**CA**	**frn**	**fms**	**fmc**	**pst**	**ngt**	**brd**	**rfl**	**dst**	**agg**	**exp**
					Correlations					
yes: age 14	-0.40	-0.34	-0.46	-0.62	-0.77	-0.67	-0.44	-0.41	-0.40	-0.03
yes: age 17	-0.41	-0.39	-0.42	-0.56	-0.78	-0.61	-0.44	-0.35	-0.42	-0.02
no: age 14	-0.35	-0.27	-0.36	-0.53	-0.71	-0.65	-0.45	-0.20	-0.55	0.09
no: age 17	-0.37	-0.30	-0.33	-0.51	-0.74	-0.63	-0.40	-0.19	-0.31	0.05
					Regularized Partical Correlations					
yes: age 14	-0.18	0.00	-0.13	-0.16	-0.39	-0.28	-0.003	-0.14	-0.23	0.00
yes: age 17	-0.14	-0.03	-0.05	-0.08	-0.50	-0.22	-0.11	-0.13	-0.26	0.00
no: age 14	-0.07	0.00	-0.05	-0.14	-0.38	-0.30	-0.02	0.00	-0.35	0.11
no: age 17	-0.12	-0.01	-0.06	-0.12	-0.46	-0.30	-0.06	0.00	-0.10	0.03

Note. CA = Childhood adversity (yes: n = 638, no: n = 501). Frn = friend support, fms = family support, fmc = family cohesion, pst = positive self-esteem, ngt = negative self-esteem, brd = brooding, rfl = reflection, dst = distress tolerance, agg = aggression, exp = expressive suppression.

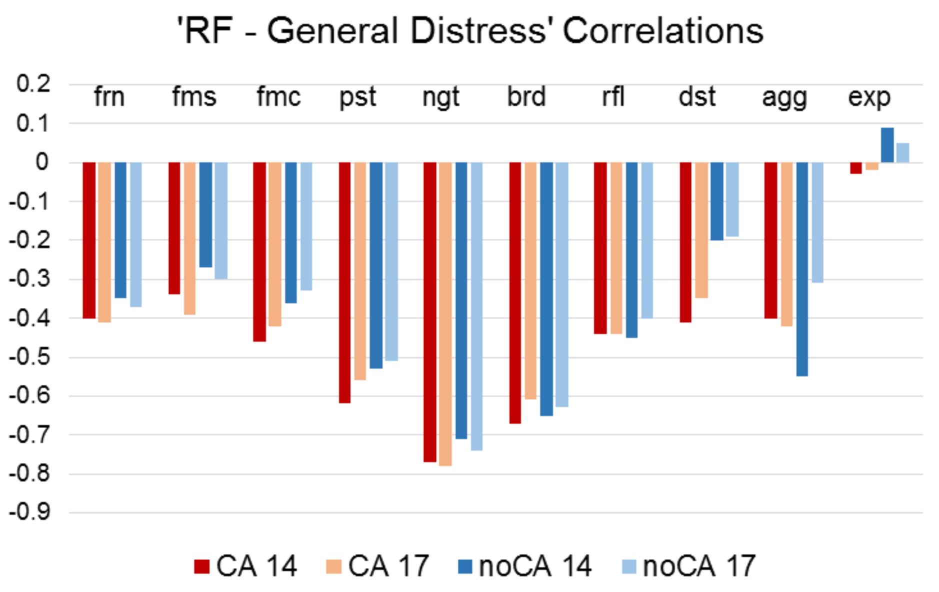

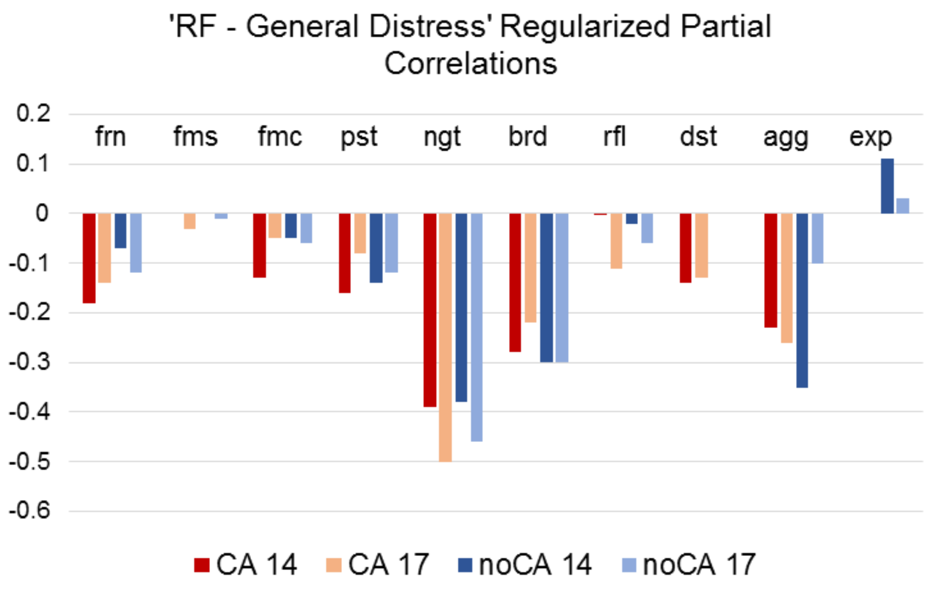

Figure 9. Visualizing the correlations and regularized partial correlations between RFs and general distress, for CA+ (n = 638) and CA- (n = 501) adolescents at age 14 and age 17. Frn = friend support, fms = family support, fmc = family cohesion, pst = positive self-esteem, ngt = negative self-esteem, brd = brooding, rfl = reflection, dst = distress tolerance, agg = aggression, exp = expressive suppression.

Supplement X

To test the stability of the *expected influence (EI)* coefficients we applied a subset bootstrap (2000 bootstraps) to identify the maximum sample percentage that can be dropped to reveal (with a 95% chance) a relationship of ≥0.7 between the subset and the original EI coefficients. The analyses showed that at both age 14 and 17 EI coefficients were sufficiently stable, as more than 50 percent of the sample could be dropped (see Table 7).

To test the accuracy of the network models we bootstrapped the 'RF-RF' and 'RF-general distress' interrelations (2000 bootstraps) and investigated the bootstrap confidence intervals (CIs). Overall, the CIs had an acceptable width, which indicates that the estimated models have an appropriate interrelation accuracy. At age 14 and age 17, family cohesion and family support were most strongly interrelated, followed next by the brooding and reflective rumination interrelation, and then by the positive and negative self-esteem interrelation, for both the CA+ and the CA- group (see Figure 10 and Figure 11). In the networks with general distress (Figure 11), negative self-esteem was also highly negatively interrelated with general distress, at both age 14 and 17.

Table 7

Expected Influence (EI) Stability

**CA**	**Age**	**MDP_{EI}**	**Case range for MDP_{EI}**	**MDP_{EI}**	**Case range for MDP_{EI}**
		Without general distress		*With general distress*	
Yes	14	0.75	(caseMin = 0.72, caseMax = 1)	0.75	(caseMin = 0.72, caseMax = 1)
No	14	0.75	(caseMin = 0.72, caseMax = 1)	0.75	(caseMin = 0.72, caseMax = 1)
Yes	17	0.75	(caseMin = 0.72, caseMax = 1)	0.75	(caseMin = 0.72, caseMax = 1)
No	17	0.72	(caseMin = 0.69, caseMax = 0.75)	0.72	(caseMin = 0.69, caseMax = 0.75)

Note. CA = childhood adversity. MDP = Maximum drop proportion.

CA+	CA-

Age 14, without general distress

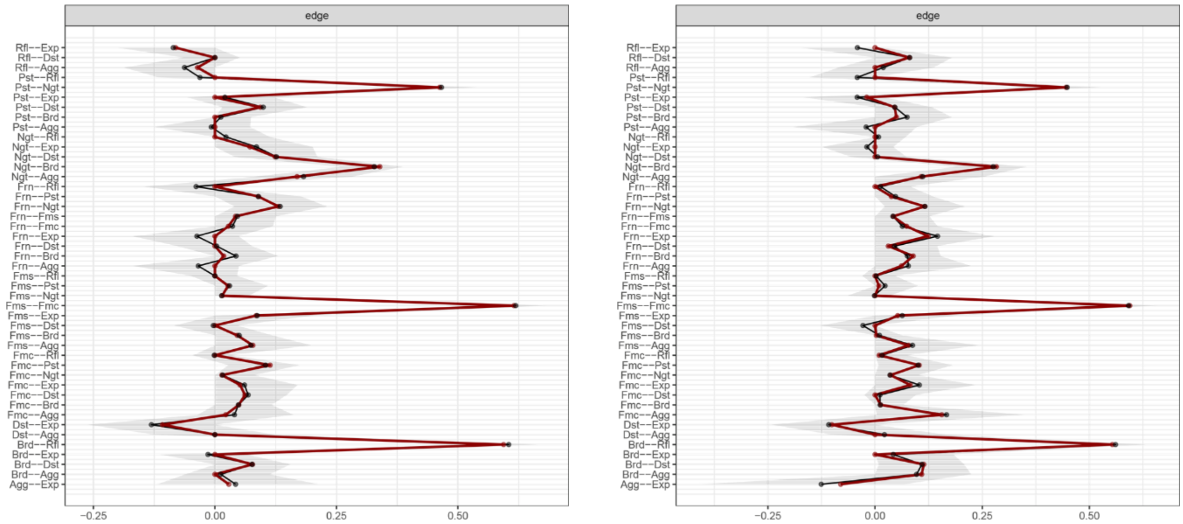

Age 17, without general distress

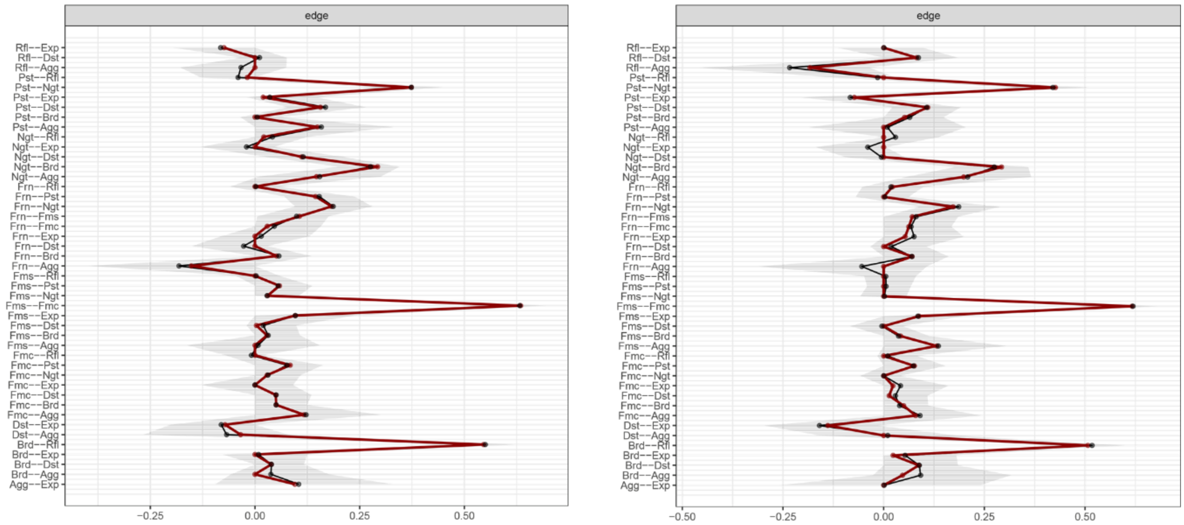

Figure 10. Interrelation accuracy plots for CA+ (*n* = 638) and CA- (*n* = 501) networks without the general distress variable, for both age 14 and age 17. The plots depict the sample RF interrelations (i.e. edge weights) which are represented by the red dots, the means of the bootstrapped RF interrelations (i.e. edge weights) which are represented by the black dots, and the belonging bootstrap confidence intervals (CIs) which indicate the RF interrelation accuracy. Frn = friend support, fms = family support, fmc = family cohesion, pst = positive self-esteem, ngt = negative self-esteem, brd = brooding, rfl = reflection, dst = distress tolerance, agg = aggression, exp = expressive suppression.

**CA+**	**CA-**

Age 14, with general distress

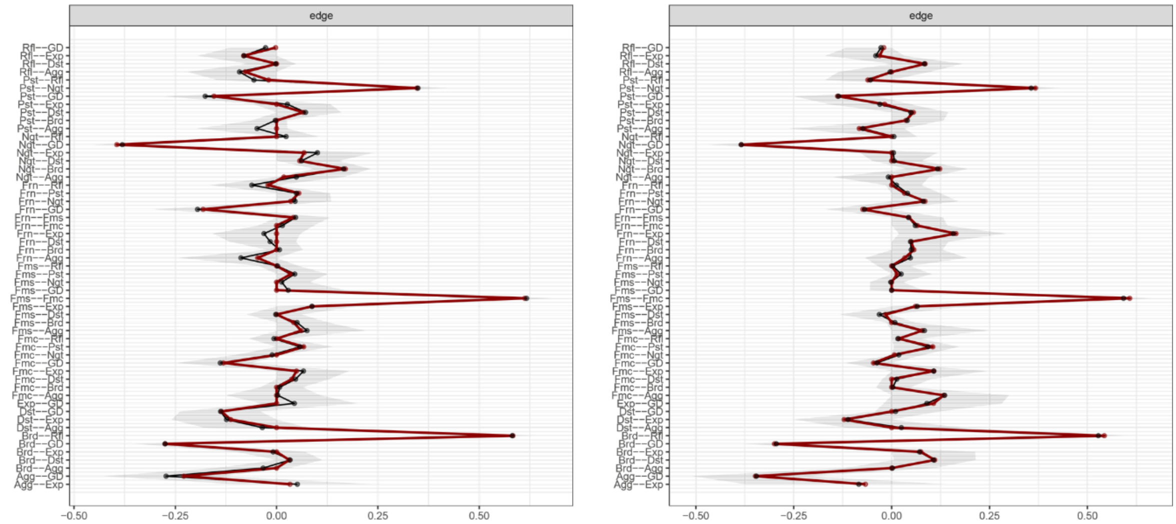

Age 17, with general distress

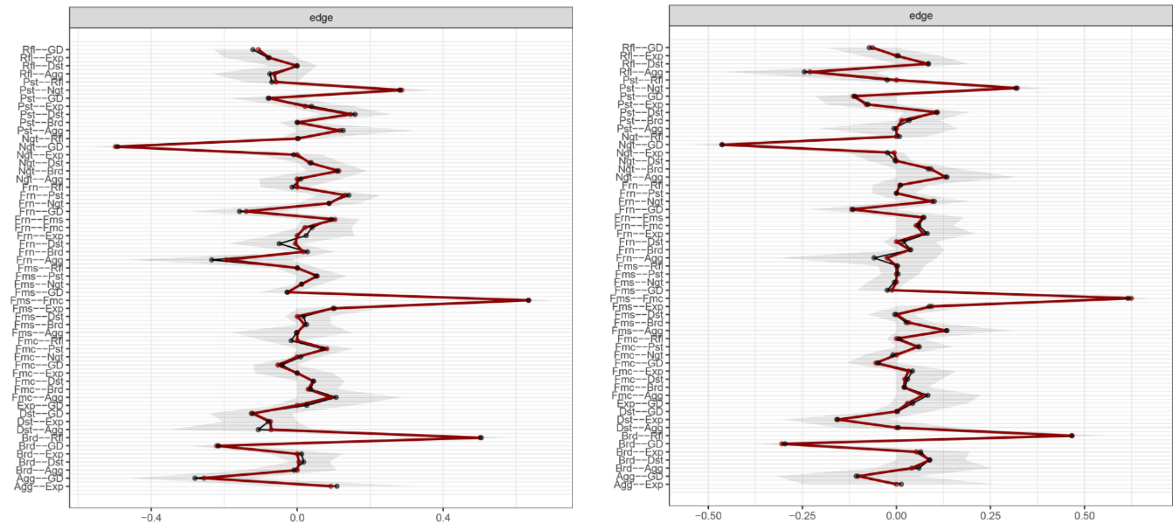

Figure 11. Interrelation accuracy plots for CA+ (*n* = 638) and CA- (*n* = 501) networks with the general distress variable, for both age 14 and age 17. The plots depict the sample 'RF-RF' and 'RF-general distress' interrelations (i.e. edge weights) which are represented by the red dots, the means of the bootstrapped interrelations (i.e. edge weights) which are represented by the black dots, and the belonging bootstrap confidence intervals (CIs) which indicate the 'RF-RF' and 'RF-general distress' interrelation accuracy. Frn = friend support, fms = family support, fmc = family cohesion, pst = positive self-esteem, ngt = negative self-esteem, brd = brooding, rfl = reflection, dst = distress tolerance, agg = aggression, exp = expressive suppression, GD = general distress.

Supplement XI

The connectivity analyses for expected influence (EI) coefficients showed that the family, ruminative brooding and self-esteem RFs had the highest coefficients, in both groups and at both time points (see Table 8). For the CA+ group, aggression had for both time points the lowest EI coefficient. For the CA- group, aggression had the lowest EI coefficient for age 14 and expressive suppression had the lowest EI coefficient for age 17. We did not detect any particular age or group patterns (see Figure 12).

Table 8

Expected Influence (EI) for Networks Corrected for General Distress

**CA**	**frn**	**fms**	**fmc**	**pst**	**ngt**	**brd**	**rfl**	**dst**	**agg**	**exp**
yes: age 14	0.06	0.88	0.77	0.55	0.69	0.83	0.38	0.08	-0.01	0.04
yes: age 17	0.14	0.93	0.91	0.79	0.54	0.69	0.29	0.06	-0.05	0.06
no: age 14	0.53	0.79	1.04	0.45	0.58	0.94	0.56	0.17	0.10	0.17
no: age 17	0.31	0.95	0.88	0.42	0.63	0.84	0.33	0.15	0.12	0.01

Note. CA = Childhood adversity (yes: *n* = 638, no: *n* = 501). Frn = friend support, fms = family support, fmc = family cohesion, pst = positive self-esteem, ngt = negative self-esteem, brd = brooding, rfl = reflection, dst = distress tolerance, agg = aggression, exp = expressive suppression.

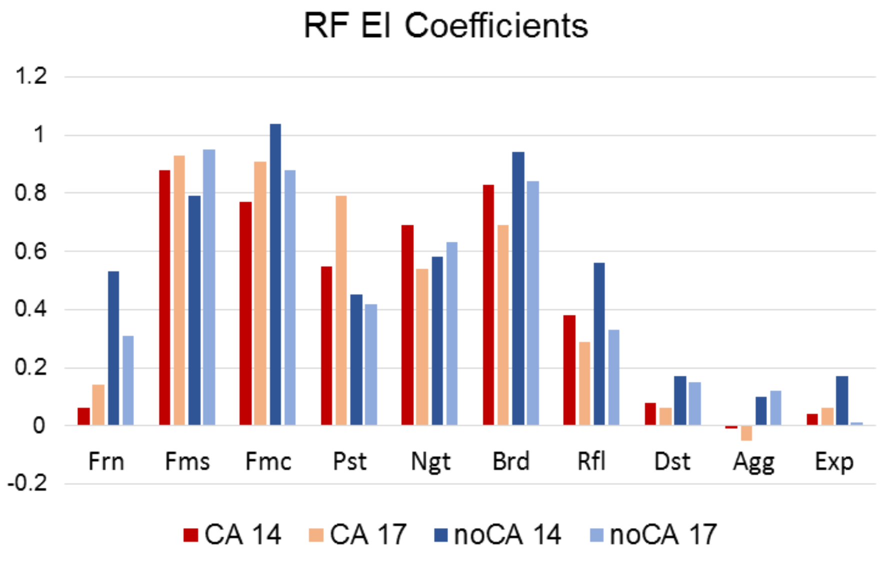

Figure 12. Visualizing expected influence (EI) coefficients for CA+ (*n* = 638) and CA- (*n* = 501) networks corrected for the general distress variable, for both age 14 and age 17. Frn = friend support, fms = family support, fmc = family cohesion, pst = positive self-esteem, ngt = negative self-esteem, brd = brooding, rfl = reflection, dst = distress tolerance, agg = aggression, exp = expressive suppression.

Supplement XII

The following three figures depict CA+ and CA- networks as presented in the main manuscript, however this time excluding the brooding variable. The models were estimated separately for age 14 and 17, as well as (1) once without the general distress variable, (2) once with the general distress variable, and (3) once corrected for the general distress variable. At age 14, the network invariance test was not significant for the networks without the general distress variable (M = .15, p = .68; see Figure 13), and the global network expected influence did not differ between the CA+ and the CA- RF networks (EI_{CA+} = 2.58, EI_{CA-} = 2.75, EI = 0.17, p = .41). Those findings were similar in the networks for age 17, as neither of the two tests revealed significant differences between the CA+ and the CA- group (M = .17, p = .83; EI_{CA+} = 2.68, EI_{CA+} = 2.36, EI = 0.31, p = .22). When we compared the RF networks for age 14 and age 17, we did not find any significant global network structure differences; neither for adolescents with (M = .17, p = .41; EI_{14} = 2.58, EI_{17} = 2.68, EI = 0.10, p = .55) nor for adolescents without a history of adversity (M = .24, p = .17; EI_{14} = 2.75, EI_{17} = 2.36, EI = 0.38, p = .11).

For the networks with the general distress variable, the network invariance test (M = .19, p = .41; see Figure 14) was not significant at age 14. However, the global network expected influence differed significantly between the CA+ and the CA- networks (EI_{CA+} = -0.12, EI_{CA-} = 0.80, EI = 0.92, $p < .05$). Those findings were only partially similar in the networks for age 17, as neither of the two tests revealed significant differences between the CA+ and the CA- group (M = .26, p = .34; EI_{CA+} = -0.18, EI_{CA-} = 0.39, EI = 0.57, p = .30). When we compared the networks for age 14 and age 17, we again did not find any significant global network structure differences; neither for adolescents with (M = .21, p = .25; EI_{14} = -0.12, EI_{17} = -0.18, EI = 0.06, p = .91) nor for adolescents without a history of adversity (M = .26, p = .15; EI_{14} = 0.80, EI_{17} = 0.39, EI = 0.40, p = .32).

For the networks corrected for the general distress variable, the network invariance test (M = .19, p = .36; see Figure 15) did not differ between the CA+ and the CA- group, at age 14. In contrast, the global network expected influence differed between the CA+ and the CA- networks (EI_{CA+} = 1.44, EI_{CA-} = 2.07, EI = 0.62, $p < .05$). Those findings were again only partially similar in the networks for age 17, as neither of the two tests revealed significant differences between the CA+ and the CA- group (M = .26, p = .30; EI_{CA+} = 1.44, EI_{CA-} = 1.63, EI = 0.20, p = .60). When we compared the networks for age 14 and age 17, we once more did not find any significant global network structure differences; neither for adolescents with (M = .21, p = .21; EI_{14} = 1.44, EI_{17} = 1.44, EI = 0.01, p = .99) nor for adolescents without a history of adversity (M = .19, p = .44; EI_{14} = 2.07, EI_{17} = 1.63, EI = 0.43, p = .14).

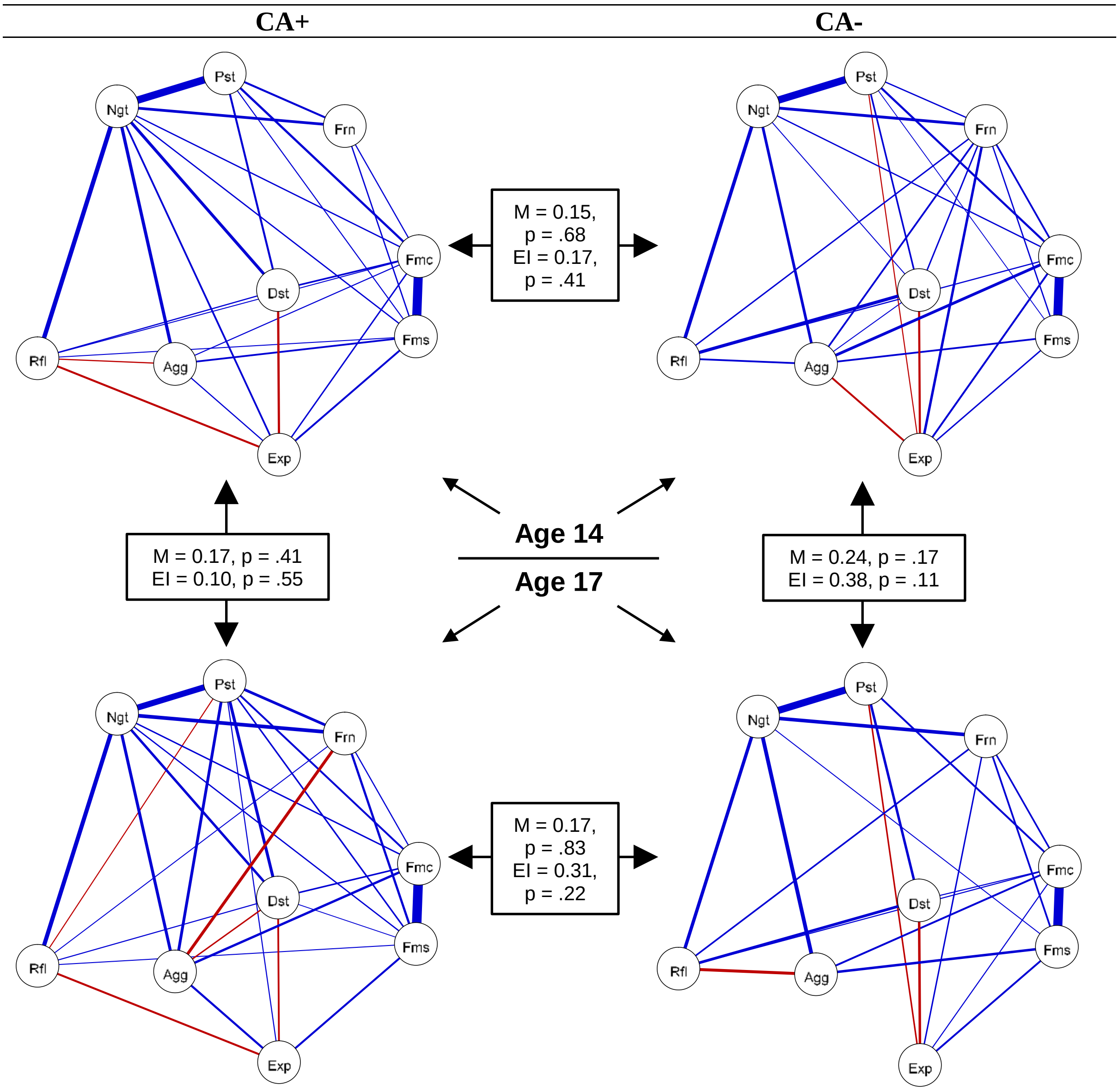

Figure 13. CA+ (*n* = 638) and CA- (*n* = 501) resilience factor networks for age 14 (upper panel) and age 17 (lower panel) without the brooding and the general distress variable. Width of the lines = association strength. Positive interrelations = blue, negative interrelations = red. **Legend:** Frn = friend support, fms = family support, fmc = family cohesion, ngt = negative self-esteem, pst = positive self-esteem, rfl = reflection, brd = brooding, dst = distress tolerance, agg = aggression, exp = expressive suppression. The boxes depict the maximal interrelation difference between the respective two networks (M), the difference in global network expected influence (EI) between the respective two networks (EI), and the corresponding p-values (5000 comparison samples).

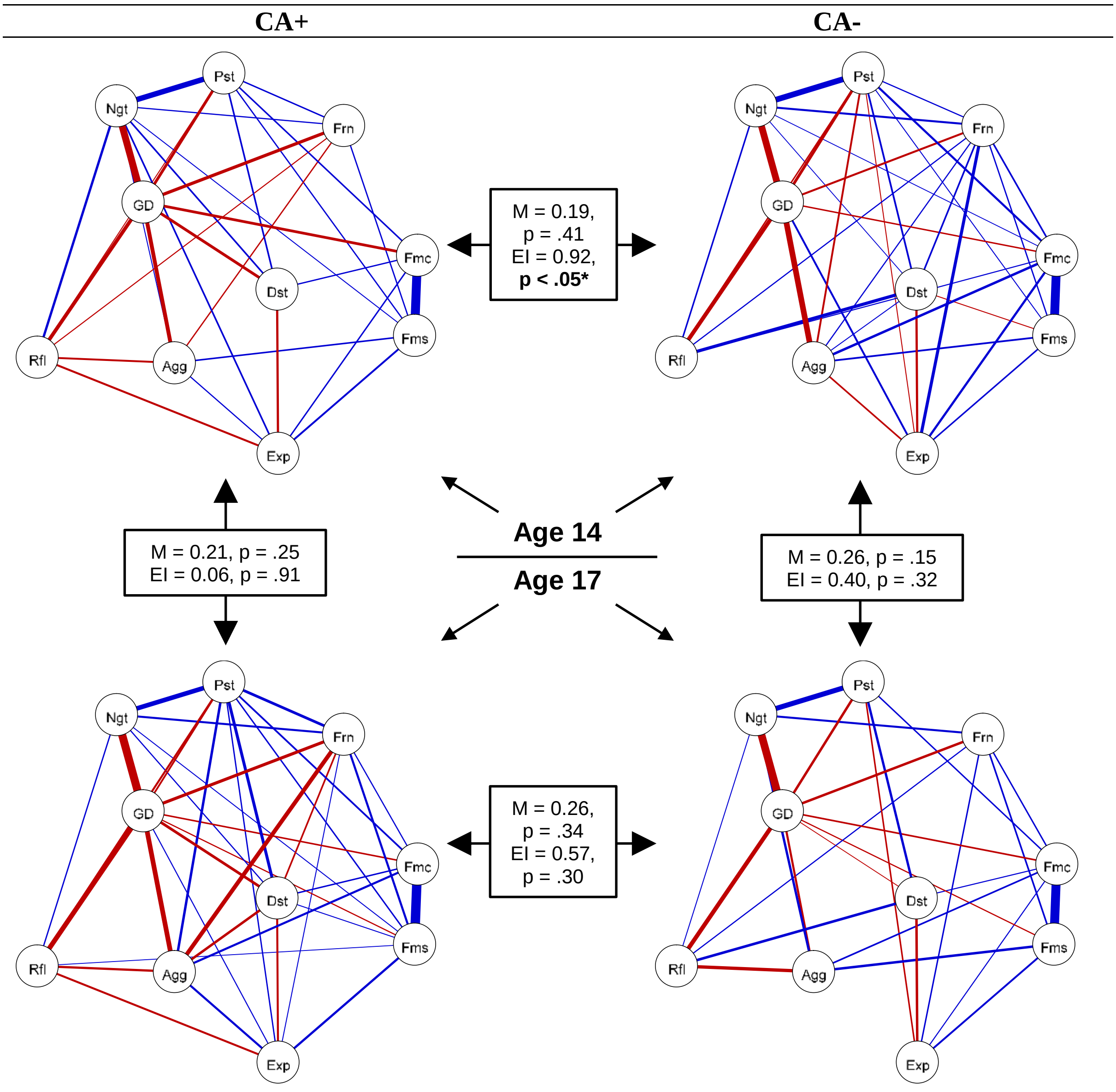

Figure 14. CA+ (*n* = 638) and CA- (*n* = 501) resilience factor networks for age 14 (upper panel) and age 17 (lower panel) without the brooding variable, but with the general distress variable. Width of the lines = association strength. Positive interrelations = blue, negative interrelations = red. **Legend:** Frn = friend support, fms = family support, fmc = family cohesion, ngt = negative self-esteem, pst = positive self-esteem, rfl = reflection, brd = brooding, dst = distress tolerance, agg = aggression, exp = expressive suppression, GD = general distress. The boxes depict the maximal interrelation difference between the respective two networks (M), the difference in global network expected influence (EI) between the respective two networks (EI), and the corresponding p-values (5000 comparison samples).

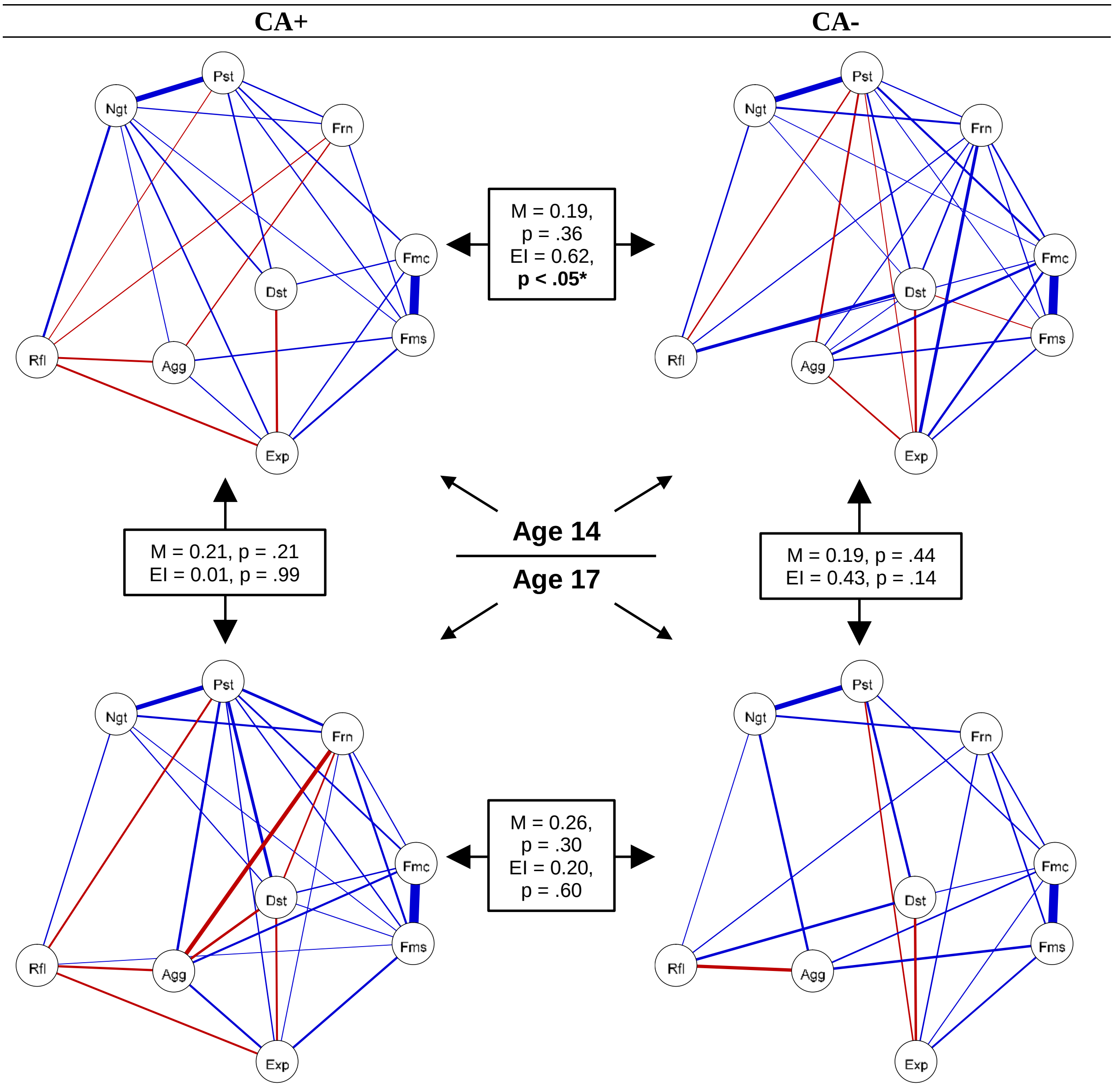

Figure 15. CA+ (*n* = 638) and CA- (*n* = 501) resilience factor networks for age 14 (upper panel) and age 17 (lower panel) without the brooding variable and corrected for the general distress variable. Width of the lines = association strength. Positive interrelations = blue, negative interrelations = red. **Legend:** Frn = friend support, fms = family support, fmc = family cohesion, ngt = negative self-esteem, pst = positive self-esteem, rfl = reflection, brd = brooding, dst = distress tolerance, agg = aggression, exp = expressive suppression, GD = general distress. The boxes depict the maximal interrelation difference between the respective two networks (M), the difference in global network expected influence (EI) between the respective two networks (EI), and the corresponding p-values (5000 comparison samples).

Supplement XIII

The following three figures depict CA+ and CA- networks with factor scores derived from the configurable CFA models. The models were estimated separately for age 14 and 17, as well as (1) once without the general distress variable, (2) once with the general distress variable, and (3) once corrected for the general distress variable. At age 14, the network invariance test was not significant for the networks without the general distress variable (M = .13, p = .83; see Figure 16) and the global network expected influence did not differ between the CA+ and the CA- RF networks (EI_{CA+} = 3.23, EI_{CA-} = 3.29, EI = 0.07, p = .72). Those findings were similar in the networks for age 17, as neither of the two tests revealed significant differences between the CA+ and the CA- group (M = .24, p = .29; EI_{CA+} = 3.30, EI_{CA-} = 3.07, EI = 0.23, p = .35). When we compared the networks for age 14 and age 17, we did not find any significant global network structure differences; neither for adolescents with (M = .17, p = .31; EI_{14} = 3.23, EI_{17} = 3.30, EI = 0.07, p = .67) nor for adolescents without a history of adversity (M = .24, p = .14; EI_{14} = 3.29, EI_{17} = 3.07, EI = 0.22, p = .29).

For the networks with the general distress variable, the network invariance test (M = .18, p = .42; see Figure 17) was not significant at age 14. However, the global network expected influence differed between the CA+ and the CA- networks (EI_{CA+} = 0.63, EI_{CA-} = 1.45, EI = 0.83, p = .03). Those findings were only partially similar in the networks for age 17, as neither of the two tests revealed significant differences between the CA+ and the CA- group (M = .18, p = .74; EI_{CA+} = 0.64, EI_{CA-} = 1.09, EI = 0.45, p = .39). When we compared the networks for age 14 and age 17, we again did not find any significant global network structure differences; neither for adolescents with (M = .18, p = .39; EI_{14} = 0.63, EI_{17} = 0.64, EI = 0.01, p = .98) nor for adolescents without a history of adversity (M = .24, p = .19; EI_{14} = 1.45, EI_{17} = 1.09, EI = 0.36, p = .35).

For the networks corrected for the general distress variable, the network invariance test (M = .18, p = .37; see Figure 18) was again not significant, at age 14, but the global network expected influence differed between the CA+ and the CA- networks (EI_{CA+} = 2.13, EI_{CA-} = 2.66, EI = 0.53, p < .05). Those findings were again only partially similar in the networks for age 17, as neither of the two tests revealed significant differences between the CA+ and the CA- group (M = .18, p = .68; EI_{CA+} = 2.16, EI_{CA-} = 2.30, EI = 0.14, p = .71). When we compared the networks for age 14 and age 17, we once more did not find any significant global network structure differences; neither for adolescents with (M = .18, p = .36; EI_{14} = 2.13, EI_{17} = 2.16, EI = 0.03, p = .92) nor for adolescents without a history of adversity (M = .24, p = .17; EI_{14} = 2.66, EI_{17} = 2.30, EI = 0.36, p = .19).

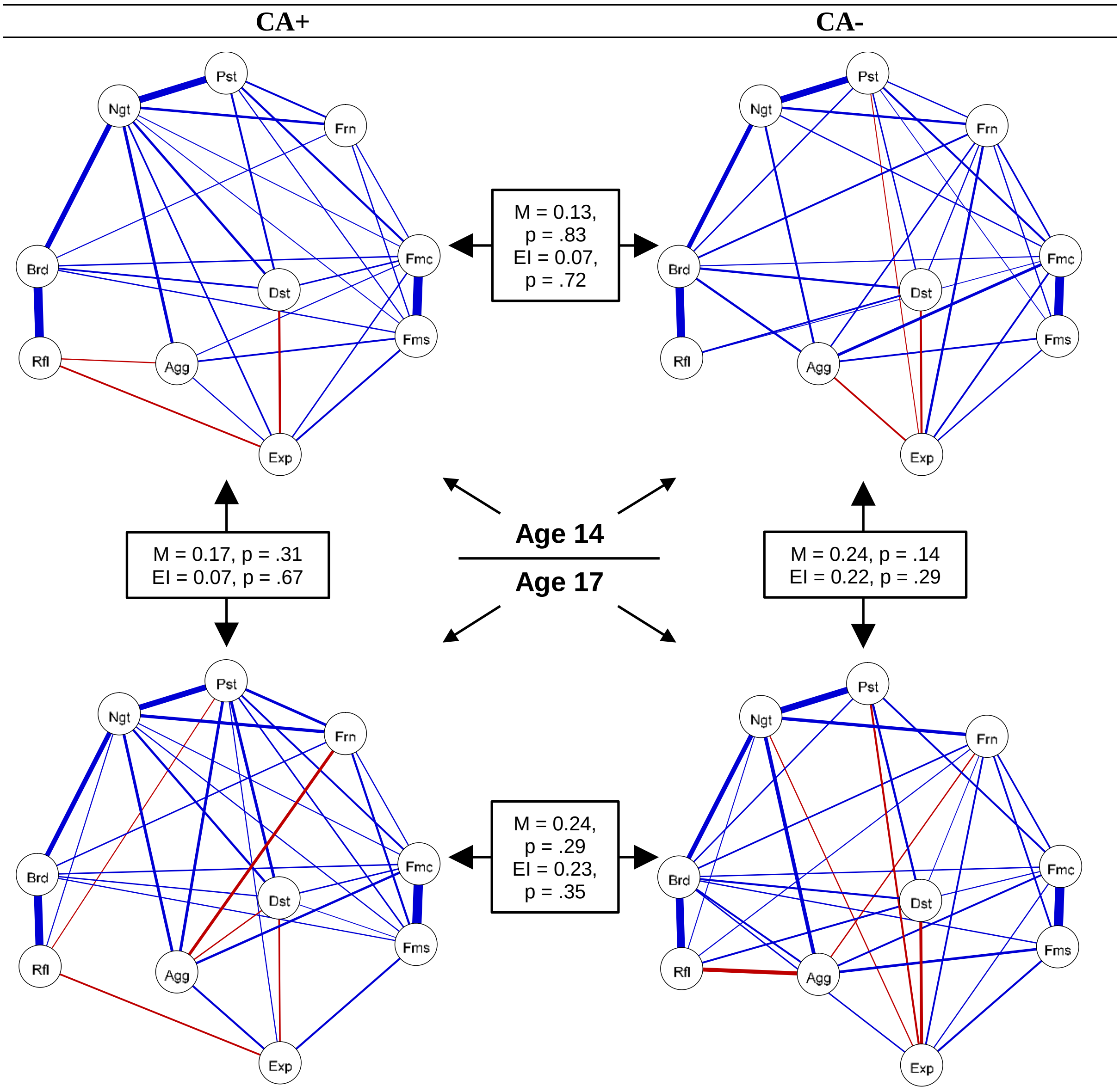

Figure 16. CA+ (*n* = 638) and CA- (*n* = 501) resilience factor networks with configural factor scores for age 14 (upper panel) and age 17 (lower panel) without the general distress variable. Width of the lines = association strength. Positive interrelations = blue, negative interrelations = red. **Legend:** Frn = friend support, fms = family support, fmc = family cohesion, ngt = negative self-esteem, pst = positive self-esteem, rfl = reflection, brd = brooding, dst = distress tolerance, agg = aggression, exp = expressive suppression. The boxes depict the maximal interrelation difference between the respective two networks (M), the difference in global network expected influence (EI) between the respective two networks (EI), and the corresponding p-values (5000 comparison samples).

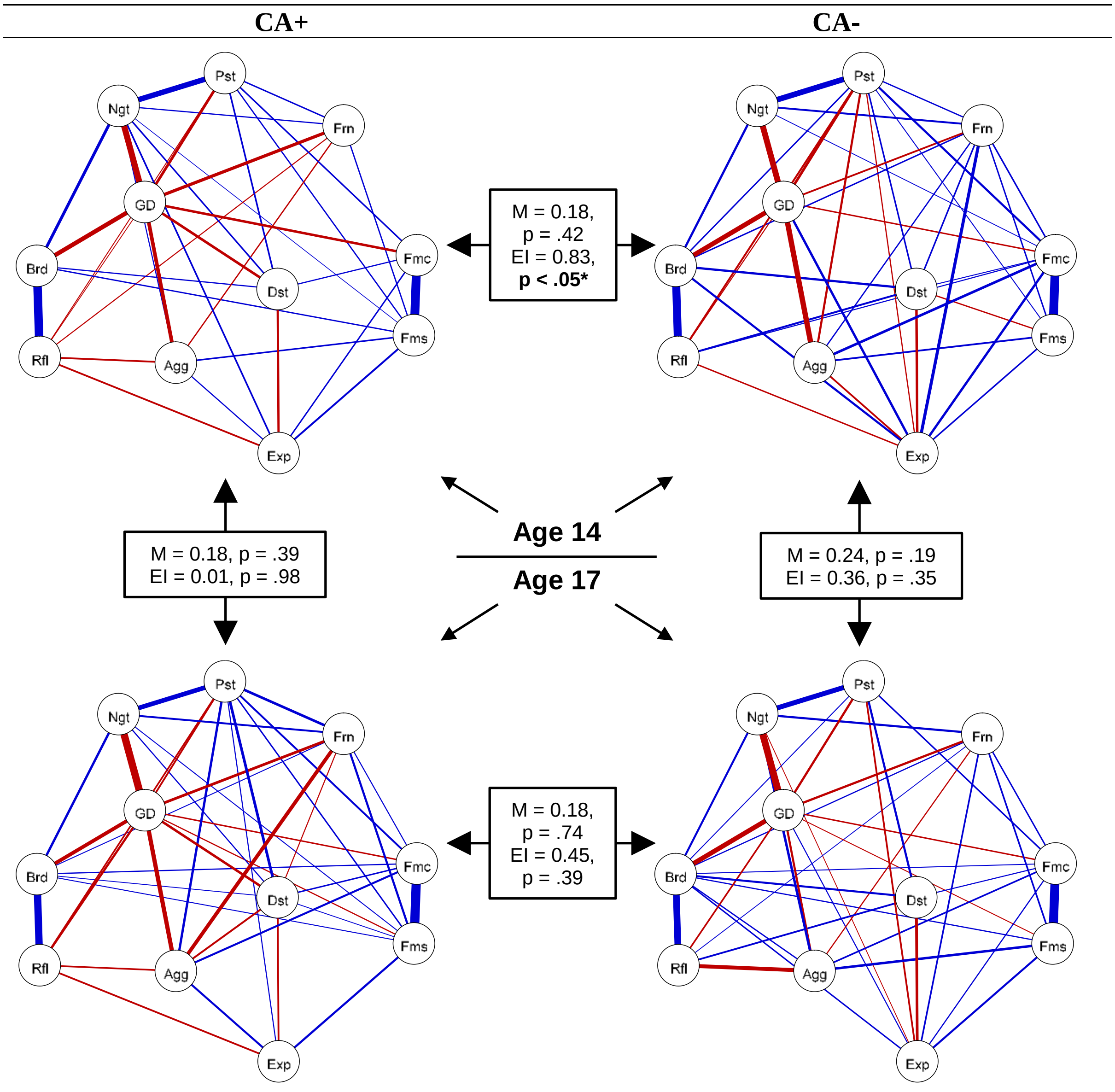

Figure 17. CA+ (*n* = 638) and CA- (*n* = 501) resilience factor networks with configural factor scores for age 14 (upper panel) and age 17 (lower panel) with the general distress variable. Width of the lines = association strength. Positive interrelations = blue, negative interrelations = red. **Legend:** Frn = friend support, fms = family support, fmc = family cohesion, ngt = negative self-esteem, pst = positive self-esteem, rfl = reflection, brd = brooding, dst = distress tolerance, agg = aggression, exp = expressive suppression, GD = general distress. The boxes depict the maximal interrelation difference between the respective two networks (M), the difference in global network expected influence (EI) between the respective two networks (EI), and the corresponding p-values (5000 comparison samples).

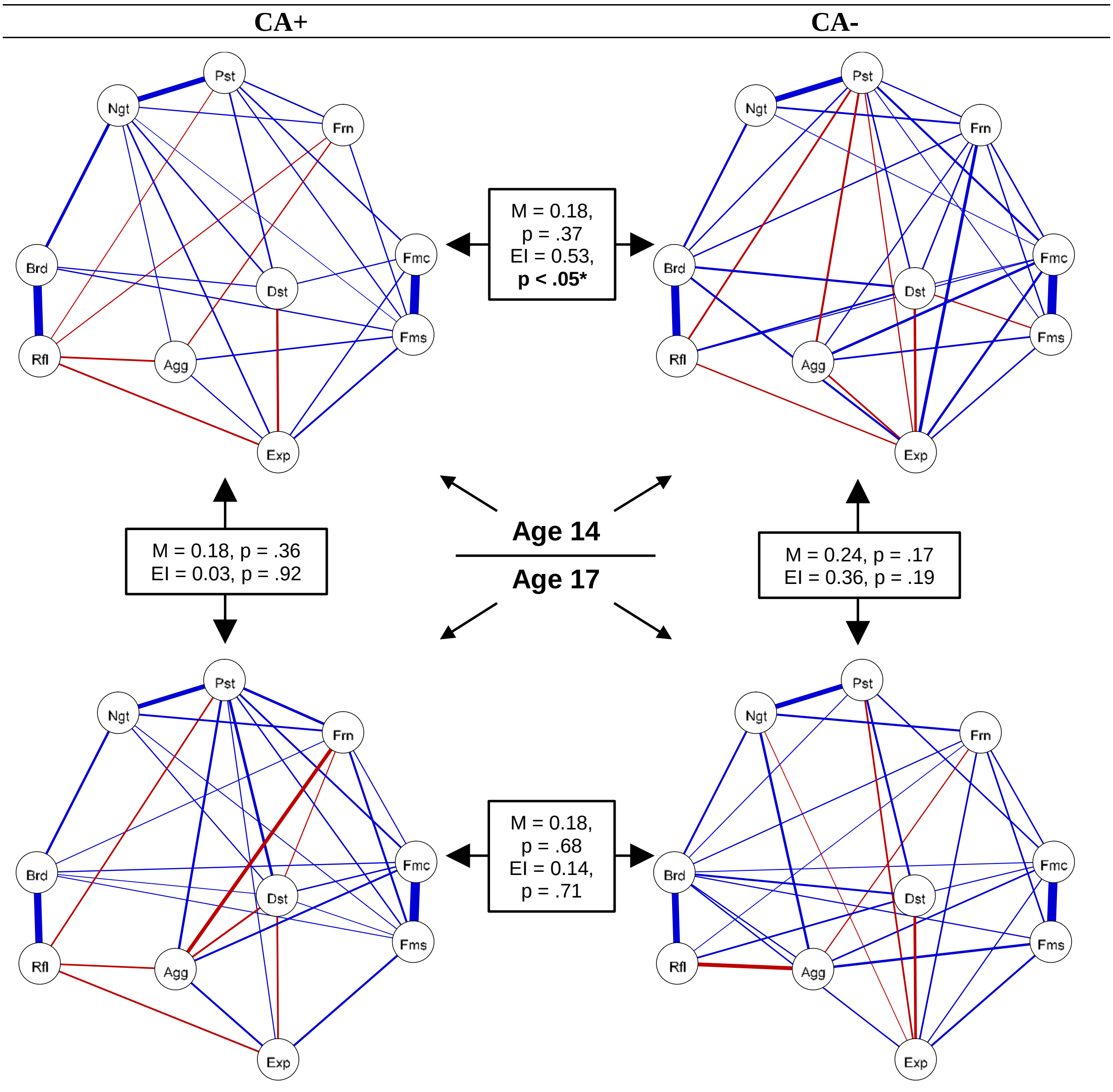

Figure 18. CA+ (*n* = 638) and CA- (*n* = 501) resilience factor networks for configural factor scores for age 14 (upper panel) and age 17 (lower panel) corrected for the general distress variable. Width of the lines = association strength. Positive interrelations = blue, negative interrelations = red. **Legend:** Frn = friend support, fms = family support, fmc = family cohesion, ngt = negative self-esteem, pst = positive self-esteem, rfl = reflection, brd = brooding, dst = distress tolerance, agg = aggression, exp = expressive suppression, GD = general distress. The boxes depict the maximal interrelation difference between the respective two networks (M), the difference in global network expected influence (EI) between the respective two networks (EI), and the corresponding p-values (5000 comparison samples).

Supplement XIV

Are resilience and risk factors opposing sides of the same coin?

The question whether resilience and risk factors lie on one continuum, representing respectively the opposite ends, has been widely discussed in the resilience literature. Yet, the answer to this question is probably not done justice with a simple yes or no. For our study the resilience factors (RFs) were derived from our systematic review[29] and were defined as follows (p. 2): "*RFs have a promotive impact on the adjustment process following CA and thus help individuals to adapt and recover from the sequelae of CA (Rutter, 1985, 2013; Zolkoski & Bullock, 2012).*[30–32] *Statistically, RFs operate as a moderator (Fergus & Zimmerman, 2005; Rutter, 1985)*[30,33]*, and/ or as a positive mediator (Masten, 2001; van Harmelen et al., 2016)*[34,35] *for the relationship between CA and psychopathology.*" We further specified as inclusion criteria that an RF "*belongs either to the individual-, family-, or community-level category, […] belongs to the cognitive, behavioural, social, and/ or emotional functioning domain, [… and should be] amenable"* (p. 3 in [29]). We specified as exclusion criteria that the RF should not be "*defined (a) as financial advantage, (b) as no re-victimization, (c) as inverse of CA, [and] (d) as inverse of psychopathology*" (p. 3 in [29]). In other words, RFs are amenable factors that operate as ameliorating or modifying variables in the relationship between adversity and mental health problems, and should neither be equivalent to CA nor to mental health problems. CA was defined as "*traumatic and/or severely stressful events, [leading to]…a higher risk of developing mental health problems*" (p. 2 in [29]). We additionally specified in detail which events would qualify as CA, in the attempt to keep the definitions of RFs and CA as separate as possible. Yet, based on our definitions, adversity is not equivalent to risk factors, as risk factors do not need to be traumatic and/or severely stressful events but still lead to a higher risk of developing mental health problems (e.g. low maternal education). With regard to our study, the quick, but insufficient answer is probably that many (or most) of the investigated RFs are indeed the flip side of risk factors. For example, self-esteem (or a positive self-concept) is commonly defined as RF and has been discussed as such by many of the seminal resilience researchers, including Michael Rutter, Emmy Werner, Ann Masten, and Michael Ungar (for a review see e.g. [36]). Yet, at the same time a low level of self-esteem or self-worth is part of the DSM V criteria for depression ("Feelings of worthlessness"; American Psychiatric Association[37]). Hence, whereas a high level of self-esteem may protect against low mood levels, low self-esteem is assumed to contribute to or reflect low mood.

That said, some RFs have been suggested not to lie on the same continuum with their supposingly opposing risk factor. For example, Carretta and colleagues[38] showed that hope and hopelessness are highly negatively correlated, but not as high as would be expected for opposing poles of the same construct.[39] Others

have proposed that not hopelessness and hope, but hopelessness and the absence of hopelessness may be bipolar.[39,40] Hence, here the risk factor may be hopelessness and the RF hope, which may however not lie on the exact same continuum. For other factors the liaison between risk and protection is even more complicated, as it depends on external factors. For instance, a low level of expressive suppression, which means that someone can effectively expresses and communicate his/her emotions, may well be advantageous in safe environments. Yet, in hazardous environments, as for example a violent home environment, emotional expression may not always be advantageous. Similarly, Luthar[41] found in adolescents from underprivileged environments that high intelligence functions as risk factor, rather than, as commonly found, as RF. Hence some RFs may be protective in one context or environment but may be harmful in another.[36] Moreover, some RFs may be particularly protective during early development and others during adulthood. For example, some researchers argue that parental support is particularly protective during childhood, but less so during adolescence.[42]

Sometimes researchers differentiate between RFs and risk factors though defining risk factors as direct effects, and RFs as moderators or mediators (e.g. see [43]). Such attempts are limited in our opinion, as both mediating and moderating RFs statistically also require a direct effect between the RF and the mental health outcome. Hence, according to such a definition one would suggest that all factors that qualify as direct effect and as mediator and/or moderator should be clustered into the RF category, whereas all factors that only qualify as direct effect should be clustered into the risk factor category. One crucial consideration that limits this definition is the present lack of replicability of RFs and risk factors. For example, Dubow and colleagues[44] found that positive parenting moderates the relationship between CA and mental health problems, while Cui and Conger[45] did not find convincing support for a moderation effect. Thus, now one would be stuck with deciding on whether high positive parenting should be considered as RF or whether low positive parenting should be considered as risk factor. Other resilience researchers have argued that both direct effects and mediating and moderating effects qualify as RFs. For example, Garmezy and colleagues[46] refer to RFs with a direct effect on mental health as “compensatory” factors and to RFs with an interaction effect on mental health as “protective” factors.

In sum, we cautiously conclude that on the group level (particularly when a rather homogeneous group is studied) many RFs (such as those included in our study) operate on a continuum with risk factors. Yet, on an individual level, the relationship between resilience and risk factors is likely to additionally depend on biological predispositions, type of adversity experienced, the specific environmental circumstances, and the developmental stage.

While neither our RF definition nor our analyses allow us to clearly demarcate the conceptualisation of resilience vs risk factors, we believe that our work expands the RF literature on another aspect. Shaik and Kauppi (p. 162-163 in [43]) state that "*[o]ne of the major shortcomings is the tendency to view factors as mono-directional influences as opposed to bi-directional influences (Glantz & Sloboda, 1999)*[47]*. These models fail to delineate how all factors can be the influences, mediators and outcomes tied in varying degrees to the entire system of variables. Despite the large number of empirical studies […], there are not sufficient details available about how and why the protective or compensatory factors directly or indirectly influence the outcomes (Lepore & Revenson, 2006)*[48]." We believe that the strength of our manuscript lies in shedding light onto the bi-directional system of RFs that are associated with a lower risk of mental distress during early and later adolescence. Regardless of whether resilience and risk factors operate on the same continuum or are inversely correlated but not identical, understanding the nature of RFs seems to have universal appeal as it focuses on what promotes good mental health rather than on what increases mental health problems. Knowledge on the promotion of good mental health in adolescents may not only be of clinical, but also of policy interest, as good mental health in today's youth may result in less mental health problems in tomorrow's adults. Or to put it into Garmezy's words (p. 171 in [49]): "*Government, by providing protective factors, enables some who would otherwise be lost to a fruitful life to move above the threshold of competence needed to survive in an increasingly complex, technological society.*"

References

1. Fritz, J., Fried, E. I., Goodyer, I. M., Wilkinson, P. O. & van Harmelen, A.-L. A Network Model of Resilience Factors for Adolescents with and without Exposure to Childhood Adversity. *Sci. Rep.* **8,** 15774 (2018).
2. Brodbeck, J., Abbott, R. A., Goodyer, I. M. & Croudace, T. J. General and specific components of depression and anxiety in an adolescent population. *BMC Psychiatry* **11,** 191 (2011).
3. Burwell, R. A. & Shirk, S. R. Subtypes of rumination in adolescence: Associations between brooding, reflection, depressive symptoms, and coping. *J. Clin. Child Adolesc. Psychol.* **36,** 56–65 (2007).
4. van Buren, S. & Groothuis-Oudshoorn, K. mice: Multivariate Imputation by Chained Equations in R. *J. Stat. Softw.* **45,** 1–67 (2011).
5. Wickham, H., François, R., Henry, L. & Müller, K. dplyr: A Grammar of Data Manipulation. R package version 0.7.7. (2018). Available at: https://cran.r-project.org/package=dplyr.
6. Grosjean, P. & Ibanez, F. pastecs: Package for Analysis of Space-Time Ecological Series. R package version 1.3.21. (2018). Available at: https://cran.r-project.org/package=pastecs.
7. Hothorn, T., Hornik, K., van de Wiel, M. A. & Zeileis, A. Implementing a Class of Permutation Tests: The coin Package. *J. Stat. Softw.* **28,** 1–23 (2008).
8. Wickham, H. Reshaping data with the reshape package. *J. Stat. Softw.* **21,** 2007 (2007).
9. Lüdecke, D. sjPlot: Data Visualization for Statistics in Social Science. R package version 2.6.2. (2018).
10. Rosseel, Y. lavaan: An R package for structural equation modeling. *J. Stat. Softw.* **48,** 1–36. Retrieved from http://www.jstatsoft.org/v48/ (2012).
11. Jorgensen, T. D., Pornprasertmanit, S., Schoemann, A. M. & Rosseel, Y. semTools: Useful tools for structural equation modeling. R package version 0.5-1.905. (2018). Available at: https://cran.r-project.org/package=semTools.
12. Wickham, H. *ggplot2: Elegant Graphics for Data Analysis.* (Springer-Verlag, 2016).
13. Epskamp, S., Cramer, A. O. J., Waldorp, L. J., Schmittmann, V. D. & Borsboom, D. qgraph: Network Visualizations of Relationships in Psychometric Data. *J. Stat. Softw.* **48,** 1–18 (2012).
14. Epskamp, S., Borsboom, D. & Fried, E. I. Estimating Psychological Networks and their Accuracy : A Tutorial Paper. *Behav. Res. Methods* **50,** 195–212 (2018).
15. van Borkulo, C. D. *Comparing network structures on three aspects: A permutation test (PhD Thesis*

Chapter 5). (University of Groningen, 2018).

16. Epskamp, S., Rhemtulla, M. & Borsboom, D. Generalized Network Psychometrics: Combining Network and Latent Variable Models. *Psychometrika* **82,** 904–927 (2017).

17. Liu, Y. *et al.* Testing measurement invariance in longitudinal data with ordered-categorical measures. *Psychol. Methods* **22,** 486–506 (2017).

18. Muthen, B. & Asparouhov, T. Latent Variable Analysis With Categorical Outcomes: Multiple-Group And Growth Modeling In Mplus. *Mplus Web Notes No. 4* Retrieved from https://www.statmodel.com/download/ (2002).

19. Sass, D. A. Testing measurement invariance and comparing latent factor means within a confirmatory factor analysis framework. *J. Psychoeduc. Assess.* **29,** 347–363 (2011).

20. Wu, H. & Estabrook, R. Identification of Confirmatory Factor Analysis Models of Different Levels of Invariance for Ordered Categorical Outcomes. *Psychometrika* **81,** 1014–1045 (2016).

21. Goodyer, I. M., Wright, C. & Altham, P. M. E. Recent friendships in anxious and depressed school age children. *Psychol. Med.* **19,** 165–174 (1989).

22. Epstein, N. B., Baldwin, L. M. & Bishop, D. S. The McMaster Family Assessment Device. *J. Marital Fam. Ther.* **9,** 171–180 (1983).

23. Rosenberg, M. *Society and the Adolescent Self-Image*. (Princeton, NJ: Princeton University Press, 1965).

24. Treynor, W., Gonzalez, R. & Nolen-Hoeksema, S. Rumination reconsidered: A psychometric analysis. *Cognit. Ther. Res.* **27,** 247–259 (2003).

25. Bould, H., Joinson, C., Sterne, J. & Araya, R. The Emotionality Activity Sociability Temperament Survey: Factor analysis and temporal stability in a longitudinal cohort. *Pers. Individ. Dif.* **54,** 628–633 (2013).

26. Goodyer, I. M. *et al.* Improving mood with psychoanalytic and cognitive therapies (IMPACT): a pragmatic effectiveness superiority trial to investigate whether specialised psychological treatment reduces the risk for relapse in adolescents with moderate to severe unipolar dep... *Trials* **12,** 175 (2011).

27. Messer, S. C., Angold, A. & Costello, E. J. Development of a Short Questionnaire for Use in Epidemiological Studies of Depression in Children and Adolescents: Factor Composition and Structure across Development. *Int. J. Methods Psychiatr. Res.* **5,** 251–262 (1995).

28. Reynolds, C. R. & Richmond, B. O. What I Think and Feel: A Revised Measure of Children's Manifest

Anxiety. *J. Abnorm. Child Psychol.* **6,** 271–280 (1978).

29. Fritz, J., de Graaff, A. M., Caisley, H., van Harmelen, A.-L. & Wilkinson, P. O. A Systematic Review of Amenable Resilience Factors that Moderate and/or Mediate the Relationship between Childhood Adversity and Mental Health in Young People. *Front. Psychiatry* **9,** 230 (2018).

30. Rutter, M. Resilience in the Face of Adversity: Protective Factors and Resistance to Psychiatric Disoder. *Br. J. Psychiatry* **147,** 598–611 (1985).

31. Rutter, M. Annual Research Review: Resilience – clinical implications. *J. Child Psychol. Psychiatry* **54,** 474–487 (2013).

32. Zolkoski, S. M. & Bullock, L. M. Resilience in children and youth: A review. *Child. Youth Serv. Rev.* **34,** 2295–2303 (2012).

33. Fergus, S. & Zimmerman, M. A. Adolescent Resilience: A Framework for Understanding Healthy Development in the Face of Risk. *Annu. Rev. Public Heal.* **26,** 399–419 (2005).

34. Masten, A. S. Ordinary Magic: Resilience Processes in Development. *Am. Psychol.* **56,** 227–238 (2001).

35. van Harmelen, A.-L. *et al.* Friendships and Family Support Reduce Subsequent Depressive Symptoms in At-Risk Adolescents. *PLoS One* **11,** e0153715 (2016).

36. VicHealth. *Current theories relating to resilience and young people: A literature review*. *Victorian Health Promotion Foundation: Melbourne, Australia* (2015).

37. American Psychiatric Association. *Diagnostic and statistical manual of mental disorders (5th ed.).* (Washington, DC: Author, 2013).

38. Carretta, C. M., Ridner, S. H. & Dietrich, M. S. Hope, hopelessness, and anxiety: A pilot instrument comparison study. *Arch. Psychiatr. Nurs.* **28,** 230–234 (2014).

39. Cheavens, J. S., Cukrowicz, K. C., Hansen, R. & Mitchell, S. M. Incorporating Resilience Factors Into the Interpersonal Theory of Suicide: The Role of Hope and Self-Forgiveness in an Older Adult Sample. *J. Clin. Psychol.* **72,** 58–69 (2016).

40. Grewal, P. K. & Porter, J. E. Hope theory: A framework for understanding suicidal action. *Death Stud.* **31,** 131–154 (2007).

41. Luthar, S. S. Vulnerability and Resilience: A Study of High-Risk Adolescents. *Child Dev.* **62,** 600–616 (1991).

42. Hostinar, C. E., Johnson, A. E. & Gunnar, M. R. Parent support is less effective in buffering cortisol stress reactivity for adolescents compared to children. *Dev. Sci.* **18,** 281–297 (2015).

43. Shaikh, A. & Kauppi, C. Deconstructing Resilience: Myriad Conceptualizations and Interpretations. *Int. J. Arts Sci.* **3,** 155–176 (2010).

44. Dubow, E. F. *et al.* Exposure to Political Conflict and Violence and Posttraumatic Stress in Middle East Youth: Protective Factors. *J. Clin. Child Adolesc. Psychol.* **41,** 402–416 (2012).

45. Cui, M. & Conger, R. D. Parenting Behavior as Mediator and Moderator of the Association Between Marital Problems and Adolescent Maladjustment. *J. Res. Adolesc.* **18,** 261–284 (2008).

46. Garmezy, N., Masten, A. S. & Tellegen, A. The Study of Stress and Competence in Children: A Building Block for Developmental Psychopathology. *Child Dev.* **55,** 97–111 (1984).

47. Glantz, M. D. & Sloboda, Z. Analysis and reconceptulization of resilience. in *Resilience and development: Positive life adaptations* (eds. Glantz, M. D. & Johnson, J. L.) 17–83 (New York: Kluwer Academic/Plenum Publishers., 1999).

48. Lepore, S. J. & Revenson, T. A. Reslience and posttraumatic growth: Recovery, resistance, and reconfiguration. in *Handbook of posttraumatic growth: research and practice* (eds. Calhoun, L. G. & Tedeschi, R. G.) 24–46 (New York: Routledge Taylor & Francis Group, 2006).

49. Garmezy, N. Stress, competence, and development: Continuities in the study of schizophrenic adults, children vulnerable to psychopathology, and the search for stress-resistant children. *Am. J. Orthopsychiatry* **57,** 159–174 (1987).
